# Within-microenvironment exposure to particulate matter and health effects in children with asthma: a pilot study utilizing real-time personal monitoring with GPS interface

**DOI:** 10.1186/s12940-016-0181-5

**Published:** 2016-10-10

**Authors:** Nathan Rabinovitch, Colby D. Adams, Matthew Strand, Kirsten Koehler, John Volckens

**Affiliations:** 1 Department of Pediatrics, National Jewish Health, Denver, CO USA; 2 Department of Environmental and Radiological Health Sciences, Colorado State University, Fort Collins, CO USA; 3 Division of Biostatistics and Bioinformatics, National Jewish Health, Denver, CO USA; 4 Department of Environmental Health Sciences, Johns Hopkins University, Baltimore, MA USA; 5 Department of Mechanical Engineering, Colorado State University, 1374 Campus Delivery, Fort Collins, CO 80523 USA

**Keywords:** Spatio-temporal, Aerosol, Air pollution, Inflammation, Epidemiology

## Abstract

**Background:**

Most particulate matter (PM) and health studies in children with asthma use exposures averaged over the course of a day and do not take into account spatial/temporal variability that presumably occurs as children move from home, into transit and then school microenvironments. The objectives of this work were to identify increases in morning PM exposure occurring within home, transit and school microenvironments and determine their associations with asthma-related inflammation and rescue medication use.

**Methods:**

In 2007–2008, thirty Denver-area schoolchildren with asthma performed personal PM exposure monitoring using a real-time sensor integrated with a geographic information system (GIS) to apportion exposures to home, transit and school microenvironments. Concurrently, daily monitoring of the airway inflammatory biomarker urinary leukotriene E4 (uLTE_4_) and albuterol usage was performed.

**Results:**

Mean PM exposures each morning were relatively well correlated between microenvironments for subject samples (0.3 < r < 0.8), thus limiting use of this exposure metric to attribute health effects to PM exposure in specific microenvironments. Within-microenvironment increases in exposure, such as would be characterized by one or a series of transient spikes or a sustained increase in concentration (*exposure event*), however, were not strongly correlated between microenvironments (|r| < 0.25). On days when children were exposed to a ≥ 5μg/m^3^ *exposure event* during transit, they demonstrated a 24.0 % increase in uLTE_4_ (95 % CI: 1.5 %, 51.5 %) and a 9.7 % (−5.9 %, 27.9 %) increase in albuterol usage compared to days without transit *exposure events*. Associations between *exposure events* and health outcomes in home and school microenvironments tended to be positive as well, but weaker than for transit.

**Conclusions:**

School children with asthma moving across morning microenvironments experience spatially heterogeneous PM exposures with potentially varying health effects.

**Electronic supplementary material:**

The online version of this article (doi:10.1186/s12940-016-0181-5) contains supplementary material, which is available to authorized users.

## Background

Time-series studies have reported associations between ambient air pollution concentrations and acute asthma exacerbations in adults and children with asthma [1]. These studies often utilize fixed-site monitors that measure ambient particulate matter (PM) thought to be primarily products of combustion from energy consumption and transportation sources in the urban setting. However, these fixed-site monitors are unable to fully capture the spatial and temporal heterogeneity of an individual’s personal exposure due to a combination of personal behaviors and microenvironmental sources. As a result, individual exposure estimates derived from ambient monitoring data are subject to measurement error [2, 3]. Few studies have assessed asthma health effects in highly mobile populations (such as children) as a function of exposure by microenvironment.

To determine the potential role of differing microenvironments on PM exposures and their effects on asthma, the present study utilized a novel personal monitoring system that integrated a real-time sensor package with a geographic information system (GIS) [4] to apportion exposures to the home, transit and school microenvironments. This approach allowed for identification of spatially and temporally heterogeneous PM exposures that may vary by microenvironment. We characterized these microenvironmental exposures using both traditional (mean, maximum) and novel (increase, exposure event) exposure metrics. We then determined if these exposure metrics were associated with increases in airway inflammation as measured by the biomarker urinary leukotriene E4 (uLTE_4_) and with as–needed albuterol usage in schoolchildren with asthma.

## Methods

### Study subjects

Thirty elementary schoolchildren with physician-diagnosed asthma, who attended the Kunsberg (now Morgridge) School at National Jewish Health (NJH, Denver, CO), were studied over a 5-month period (December 2007- April 2008). Ethical and scientific approval was obtained from the NJH Institutional Review Board.

### Personal PM exposures by microenvironment

**Fig. 1:**
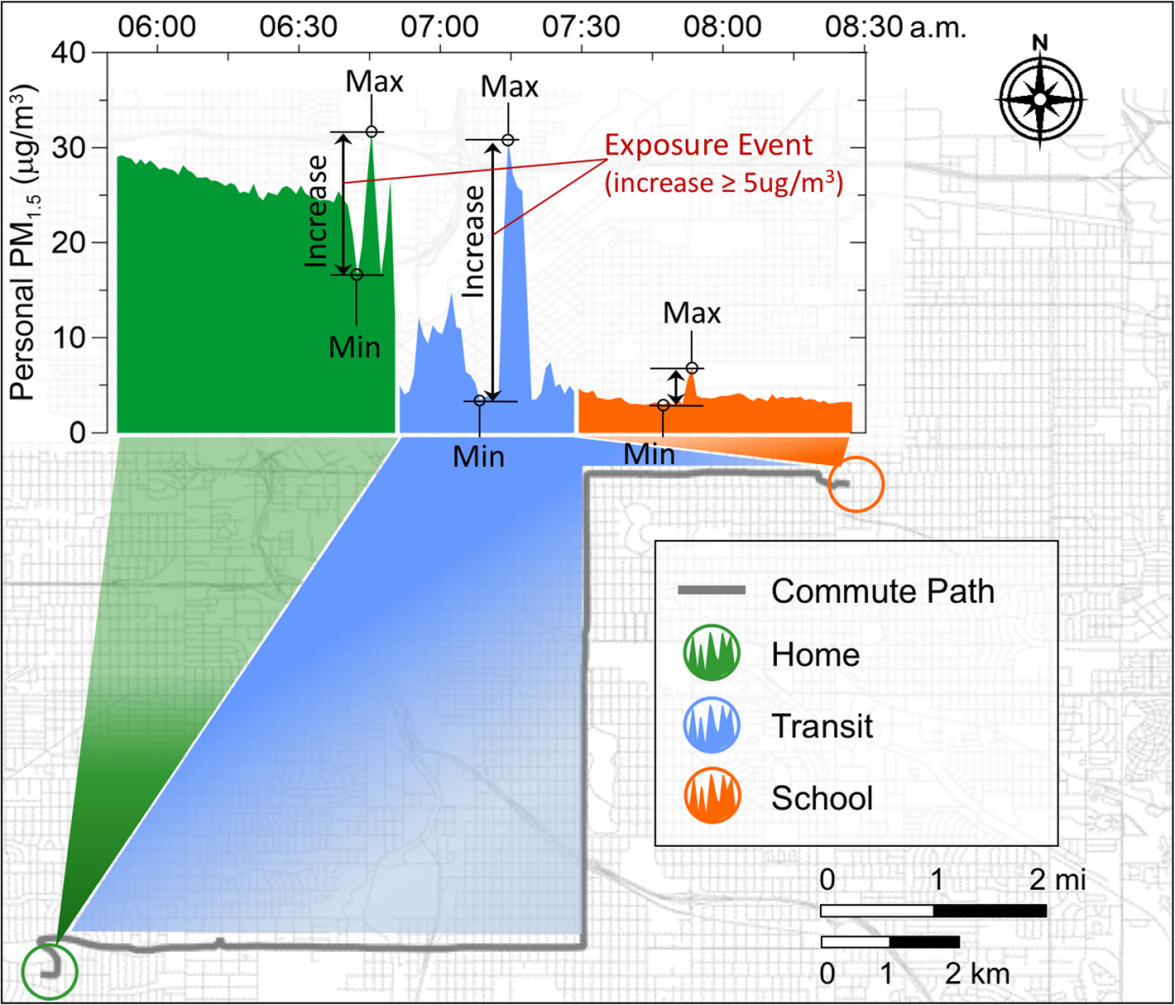
Example profile of one child’s personal exposure to PM_1.5_ across a morning commute from home to school in Denver, CO. Exposure and time-location data are collected on children in real-time and apportioned into distinct microenvironments: at home, in transit, and at school. Also shown are three observations of the *increase* variable, as they would be calculated for each microenvironment

Personal PM exposures were measured continuously on a daily basis using a recently developed method for personal, spatiotemporal exposure assessment (see [4] and on line-supplement for methodology). Each child’s air pollution exposure was monitored for up to 4 consecutive school days (mid-day Monday through mid-day Friday) across two non-consecutive weeks (8 days total). Predetermined microenvironment classifications (at home, during transit to/from school, and at school) were made with time-, location-, and sensor-based algorithms (Fig. 1; see [4] and on-line supplement for methodology).

Subjects wore sampling backpacks containing an aerosol nephelometer to measure fine PM concentrations, a global positioning system (GPS) receiver (GPSMap 60Cx, Garmin Inc. Olathe KS) to record geographic position data, and a temperature sensor to record ambient temperature (see Additional file 1: Figure S1). Each monitor recorded data at 10-s intervals. Personal PM levels were actively sampled with a Personal DataRAM 1200, or pDR, (Thermo Fisher Scientific Inc., Waltham, MA) in conjunction with a pump (6.8 L/min flow, Omni Personal Pump, BGI Inc., Waltham MA) and cyclone (1.5 μm size cut, Model GK2.05, BGI Inc., Waltham MA). The 1.5 μm aerodynamic size cut resulted from the volumetric flow necessary to meet method quantification limits for gravimetric analysis of a filter sampler (Teflo 37mm, Pall Inc. East Hills, NY) located immediately downstream of the pDR. The pDR has been used extensively to assess personal PM exposure [5–7] and has been found to be precise and in good agreement with other continuous monitors [8]. To correct for instrument bias due to PM light scattering properties, data from the pDR were normalized to mass on the corresponding personal filter [9] during data processing. The normalization factor was calculated from the ratio of the average, daily personal filter gravimetric measurement to the corresponding pDR average; this correction was specific to each child’s daily exposure. Daily meteorology measurements (temperature, relative humidity, barometric pressure) used in analyses were obtained from the Colorado Department of Public Health and Environment.

The microenvironment classification algorithm has been described in detail previously [4]. Briefly, geographical areas, or buffer regions, were developed to define an area surrounding the location of each child’s home and the school using geographical information system software (ArcGIS 9.1, ESRI Inc.). The size and shape of the buffer regions were optimized to minimize misclassification error, especially during times of transit between home and school. Using the customized home and school buffer regions in conjunction with time-based rules, a spatiotemporal algorithm was applied to apportion exposure time series into pre-determined microenvironments: at home, at school, and morning transit (i.e., commuting from home to school). Accuracy of the classification for home and school using this method during a pilot study were greater than 98 % [4]. Additional quality assurance of the processed data was performed to ensure that the child was carrying the backpack during the sampling period. Conditions for eliminating the daily sample data included when geospatial information indicated the backpack was left at school overnight, or if the backpack was left in a car overnight (as determined from temperature data).

### Health outcome measurements

An electronic monitor (Doser) recorded school-time albuterol use as the total number of activations in a 24-h period. Urine was collected between 8:00 a.m. and 11:30 a.m. (80 % between 9:30 a.m. and 10:30 a.m.) on school days (Monday – Friday). Samples were batch assayed for uLTE_4_, cotinine (a marker of environmental tobacco smoke exposure), and creatinine (to normalize levels for urinary volume) levels as previously described [10]. Daily surveys included questions determining presence or absence of upper respiratory infection (URI) symptoms and mode of transit (e.g. car, bus, van, walk to school).

### Pollution exposure concentration variables

For purposes of the health effect analysis, personal PM exposure variables were defined for the following microenvironment times: one hour before commuting to school (home), time spent commuting to school (transit), and the first hour spent within the school (school). Traditional variables used in our exposure analyses were the mean and maximum, defined as: *mean* = concentration averaged over all measurements within a given microenvironment time; *1-minute maximum* = the maximum value of 1-min averaged concentration readings within the microenvironment time. Both mean and 1-min maximum concentration variables were computed and analyzed on the natural log scale. We also constructed novel exposure variables (“*increase”* and “*exposure event”*) specifically to reduce correlation between measurements in adjacent microenvironments. We defined *increase* as the maximum concentration in the current microenvironment time minus the minimum concentration among observations that preceded the maximum, starting with the last observation in the previous microenvironment time; and *exposure event* as a binary indicator of whether the *increase* exceeded 5 or 10 μg/m^3^. Minimum and maximum concentrations used to calculate *increase* were determined based on geometric means of 1-min data (i.e., 1-min average of logged concentrations, exponentiated). Examples of *increase* and *exposure event* variables are illustrated in Fig. 1; exposure profiles for all 125 monitoring days are included in the Additional file 1.

### Statistical analyses

Health outcomes were modeled as a function of personal PM concentration variables using linear mixed models (for uLTE_4_) or generalized linear models employing generalized estimating equations (for albuterol usage counts). Daily within-subject serial correlation was modeled using a spatial power structure in the linear mixed models and a working first-order autoregressive structure in the generalized linear models. For the latter, intermittent daily measurements were accounted for by including ‘missing records’, as necessary. The linear mixed models also included random intercepts for subjects to account for general differences in uLTE_4_ levels between subjects. For albuterol models, the log link was used – i.e., Poisson regression, and for uLTE_4_ models, the outcome was logged before analysis; thus, exponentiated slope estimates from these models have relative change interpretations (after subtracting 1). For more detail, see the Additional file 1.

Based on results from our previous studies [11, 12], only the same-day and 1-day lag between pollutant and health outcome were examined (plus the 2-day average or composite). Covariates in models included upper-respiratory infection (URI) indicator for uLTE_4_, a non-exercise day indicator for albuterol (distinguishing exercise and non-exercise days at school), and ambient temperature in both models; other meteorological variables were tested but dropped due to insignificance. Reported p-values are based on 2-sided tests. Effect estimates were standardized by interquartile range (i.e. 75th percentile minus the 25th percentile for the pollutant), except for the *exposure event* dichotomized variables. All statistical analyses were conducted using SAS, PROC MIXED (version 9.4).

## Results

Table 1 contains a summary of the demographics and physiological characteristics of the panel. Children were predominately African American (43 %), followed by multiracial (36 %), Hispanic (16 %) and White (5 %). One-third had been admitted into an intensive care unit for asthma at least once in their lifetime and over 60 % had experienced an asthma exacerbation requiring a prednisone burst during the previous year. Based on the frequency of nighttime symptoms, most subjects were classified as having moderate asthma by National Asthma Education and Prevention Program guidelines [13].

**Table 1:** Demographics, asthma severity, and albuterol and uLTE_4_ levels

Subject variable	Data
Panel size	30
Mild asthma^a^	11 (36.7 %)
Moderate asthma^a^	13 (43.3 %)
Severe asthma^a^	6 (20.0 %)
African American	13 (43.3 %)
Children with at least one ICU admission for asthma	10 (33.3 %)
Children with at least one exacerbation within the past year^b^	19 (63.3 %)
Children using daily inhaled steroids	26 (86.7 %)
Urinary LTE_4_ (pg per mg creatinine)	87.44 (5.3, 839)
Albuterol (puffs per day)	1.40 (0, 3)
Age	10 (7, 13)

*Definition of abbreviation*: ICU - intensive care unit

Entries are number (percentage) of children or mean (minimum, maximum) unless otherwise indicated

^a^Daily asthma severity categories were defined by National Asthma Education and Prevention Program criteria (reference 13)

^b^Exacerbations were defined as episodes requiring hospitalization, visits to emergency or urgent care departments, or prednisone bursts

Tables 2 and 3 and Additional file 1: Table S1 summarize personal PM_1.5_ concentrations measured at home, transit and school microenvironments (for varying time scales during the morning interval) and for approximately 21 h (2:30 pm to 11:30 am) averaged concentrations (as monitors were processed and batteries replaced over a 3-h period at school). Personal exposures tended to be highest at home and lowest at school. Transit to school for most (84 %) children occurred by motor vehicle (bus, 3 %; vanpool 64 %; or car, 17 %) while 16 % of children walked to school. The majority of vehicular routes were on major city streets with traffic flow managed by stoplights. Transit to school typically began at approximately 7:00 a.m. lasting 32 min, on average (standard deviation = 15, range 9–85 min).

**Table 2:** Average PM_1.5_ concentrations by micronenvironment, μg/m^3^ ^a^

Microenvironment or time frame	Mean	Min value	25th quantile	Median	75th quantile	Max value
Home	12.7	0.2	2.5	5.2	14.3	109.5
Transit	9.2	0.1	2.4	5.3	12.2	61.6
School	6.4	0.1	1.9	3.7	8.1	44.9
Complete sample^b^	9.5	0.1	2.2	4.7	11.8	109.5

^a^Statistics were computed at the subject-day level, not distinguishing between versus within-subject data. Statistics were computed by first determining means within samples (subject, day and microenvironment), then computing statistics across the 125 samples. PM_1.5_ is particulate matter less than 1.5 μm in aerodynamic diameter. Home, Transit and School microenvironment times are summarized in the text

^b^Approximately 2:30pm through 11:30am the following day

**Table 3:** Statistics for within-sample PM_1.5_ increases (see text for formal definition of the ‘increase’ variable)^a^

Microenvironment	Mean	Min value	25th Quantile	Median	75th Quantile	Max value	% at least 5 μg/m^3^	% at least 10 μg/m^3^
Home	24.0	0.0	1.6	7.4	19.4	282.1	56.8	40.8
Transit	17.6	0.0	1.0	5.3	14.5	269.9	50.4	38.4
School	10.1	0.0	1.3	3.2	11.4	137.3	40.8	27.2

^a^Statistics were computed by exponentiating 1-min averaged logged concentrations, determining the increase amount within subject samples (subject, day and microenvironment), then computing composite statistics across the 125 samples. PM concentrations (micrograms per cubic meter) have been corrected by gravimetric filter mass collected concurrently with each daily pDR sample

Figure 1 illustrates an example of one child’s microenvironmental exposure profile. In this example, the transit and home microenvironments contain an *exposure event* (i.e. an *increase* of at least 5 ug/m^3^) but the school microenvironment does not (all profiles are included in the Additional file 1: Figure S4). Using these parameters, *exposure events* of at least 5 μg/m^3^ were detected in 56 % (*n* = 71) of home, 50 % (*n* = 63) of transit and 41 % (*n* = 51) of school profiles.

Table 4 summarizes Pearson correlations between and within microenvironments for the different exposure metrics. Mean values for personal microenvironmental exposure were correlated especially well between transit and school (*r* = 0.77); less so between home/transit (*r* = 0.42) and home/school (*r* = 0.33). In contrast to *mean* exposures, exposure *increases* in one microenvironment were only weakly correlated with *increases* in subsequent microenvironments (*r* = 0.24 for home/transit, *r* = 0.13 for transit/school, *r* = −0.09 for home/school). Mean PM exposures were moderately correlated with *increases* in the same microenvironment (*r* = 0.59 for home, *r* = 0.58 for transit, *r* = 0.42 for school).

**Table 4:**
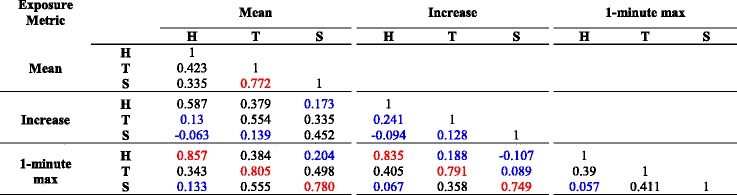
Pearson correlations within and between microenvironments for PM exposure metrics (125 records)

See text for more detail on pollution variable definitions. H = Home, T = Transit, S = School microenvironments. Pollution variables were considered on the natural log scale (before taking the relevant summary statistic). Correlations exceeding 0.7 are shown in red, and those with |r| < 0.3 are in blue

Comparisons among model goodness-of-fit statistics (using Akaike Information Criterion [AIC] for LTE_4_ and Quasi-likelihood under the independence model criterion u [QICu] for albuterol use) associating *increase, event, mean, and max* exposure metrics with health effects are provided in Additional file 1: Table S3. These goodness of fit models demonstrate that in general metrics using the *event* variables fit the data better than either the *mean* or 1-min *max* variables and that utilizing the *increase* as a continuous variable generally did not provide additional information in predicting health outcomes beyond the 5 or 10 μg/m^3^ exposure event variables. These results and the fact that increase and exposure event variables were less correlated between microenvironments, suggest that the *event* variable is the more informative metric for this study design than the more commonly used *mean* and *max* variables. In this particular dataset, we would consider the 5 μg/m^3^ cut-off as providing the most informative associations with health as it provided the best fit most often among all metrics.

**Fig. 2:**
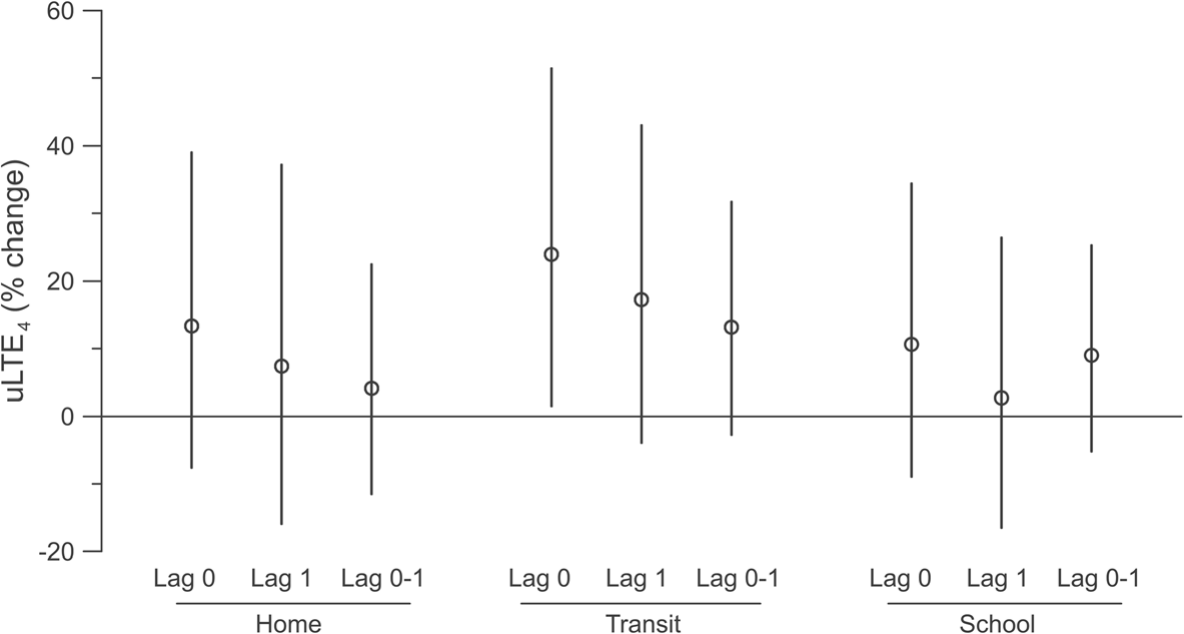
Associations between 5 μg/m^3^ (or more) exposure events and percent change in uLTE_4_ within each microenvironment for current day (lag 0), previous day (lag 1) or either day (0–1). Mean estimates are circles and error bars represent 95 % confidence intervals

**Fig. 3:**
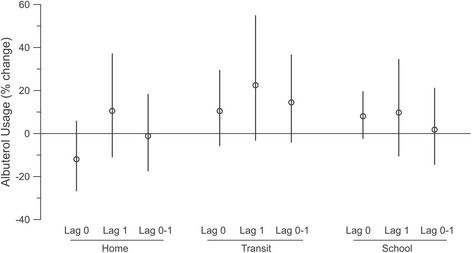
Associations between 5 μg/m^3^ (or more) exposure events and percent change school time albuterol usage within each microenvironment for current day (lag 0), previous day (lag 1) or either day (0–1). Mean estimates are circles and error bars represent 95 % confidence intervals

Health effect estimates for 5 μg/m^3^ exposure events occurring on the same day (lag 0), previous day (lag 1), or on either day (0–1) are illustrated for uLTE_4_ and albuterol usage in Figs. 2 and 3, respectively, and summarized in Table 5. In health outcome models, a 5 μg/m^3^ exposure event in the transit microenvironment was associated with a 24.0 % increase in uLTE_4_ (1.5 %, 51.5 %) and a 9.7 % (−5.9 %, 27.9 %) increase in albuterol usage on the same day. For the home and school microenvironments, estimates tended to also be positive, but somewhat weaker, as shown in Figs. 2 and 3. Lag 0, 1 and 2-day (i.e., 0–1) effect estimates based on exposure events of at least 10 μg/m^3^ yielded similar results, although not quite as pronounced as when using the 5 μg/m^3^ cut point (Table 5).

**Table 5:** Associations between health outcomes (uLTE_4_ and albuterol usage) and PM_1.5_ *exposure events* (*increase* of at least 5 or 10 μg/m^3^ within microenvironment) as measured by the personal monitor

Micro-environment	*Exposure event increase*	Lag, days	% Increase for *exposure event* (95 % CI)	
			Albuterol usage	LTE_4_
Home	5μg/m^3^	0	−1.7 (−17.6, 17.2)	13.3 (−7.6,39.1)
		1	11.0 (−10.1, 37.1)	7.4 (−16.0,37.2)
		0-1	−1.2 (−17.3, 18.0)	4.1 (−11.5,22.5)
	10μg/m^3^	0	10.3 (−6.5, 30.3)	17.7 (−4.8,45.6)
		1	13.4 (−8.3, 40.2)	−3.4 (−23.4,21.8)
		0-1	7.6 (−7.6, 25.1)	9.5 (−8.5,31.2)
Transit	5μg/m^3^	0	9.7 (−5.9, 27.9)	24.0 (1.5,51.5)
		1	17.6 (−6.9, 48.6)	17.2 (−3.9,43.1)
		0-1	13.7 (−4.3, 35.2)	13.2 (−2.8,31.7)
	10μg/m^3^	0	10.8 (−4.2, 28.2)	10.9 (−10.4,37.2)
		1	6.3 (−15.5, 33.6)	8.7 (−12.7,35.2)
		0-1	7.3 (−9.5, 27.2)	4.7 (−10.5,22.4)
School	5μg/m^3^	0	13.3 (−5.7, 36.0)	10.6 (−9.0,34.4)
		1	5.9 (−13.2, 29.1)	2.7 (−16.5,26.4)
		0-1	1.7 (−14.6, 21.1)	9.0 (−5.2,25.4)
	10μg/m^3^	0	12.4 (−5.2, 33.2)	19.0 (−5.3,49.6)
		1	3.1 (−19.3, 31.6)	0.6 (−19.8,26.3)
		0-1	4.8 (−11.6, 24.3)	9.1 (−6.0,26.6)

For 0–1 day lag, the indicator was ‘1’ if an event occurred on current or previous day, and ‘0’ otherwise; observations were weighted by the number of records used in the 0–1 variable (1 or 2 days). For Albuterol usage models, temperature and Friday indicator covariates were used; for uLTE_4_ models, temperature and cold indicator were used (the lag for cold was set as the same for the air pollution variable). 30 subjects were available for analysis; number of records used for model fits were: 114, 80 and 143 for 0, 1 and 0–1 day estimates for albuterol usage, respectively, and 80, 58 and 111 for uLTE_4_. For more detail, see the text

The microenvironment specific health-effect estimates could be confounded by the elapsed time between exposure and when the health outcome was measured, since school exposures were closest in time to outcome measurement, followed by transit, and then home exposures. Because we recorded daily urine collection times, we were able to examine this potential confounder by evaluating the timing of the relationship between personal PM exposures and uLTE_4_ measurement (i.e., urine collection). Specifically, *hr0* was constructed as the mean log PM_1.5_ concentration in the 60 min preceding urine collection, *hr1* was the mean log concentration for the 60 min ending 1 h before urine collection, and *hr2*, ending 2 h before collection. The *hr0* to *hr2* variables were tested individually as predictors in the same-day uLTE_4_ models. Although effects generally did strengthen as time-to-measurement shortened, none of the hourly concentrations were significantly associated with uLTE_4_ levels (p > 0.2). When adding *hr* variables to models with exposure *event* indicators (i.e., co-pollutant models), the *hr* variables remained insignificant and the exposure event indicators did not weaken and, in fact, increased slightly for associations with transit exposures. As previously mentioned, without an *hr* variable, there was a 24.0 % increase in lag 0 uLTE_4_ when there was one or more exposure *events of at least 5 μg/m* ^*3*^ *increase* occurring in the transit microenvironment (95 % CI: 1.5, 51.5 %); when adding *hr* variables, uLTE_4_ effect estimates (95 % CIs) with exposure *events* in transit were: 27.2 % (3.6, 56.3 %) when *hr0 was added*; 29.6 % (5.5, 59.2 %) when *hr1* was added and 30.3 % (4.0, 63.2 %) when *hr2* was added to the models. Similarly, slight increases in estimates were also observed for the school microenvironment when adding *hr* variables (from 10.6 % without an *hr* variable, up to 13.7 % when adding *hr1*), while estimates stayed approximately the same for the home microenvironment with the additions.

Health effect estimates using the *mean* metric for PM exposure are summarized in the Additional file 1: Table S2 and shown in Additional file 1: Figures S2 and S3. Similar to exposur*e events*, *mean* PM concentrations during transit were marginally associated with both uLTE_4_ and albuterol usage, while associations for school and home exposures were somewhat weaker. Not surprisingly, health-effect associations with 1-min *max* concentrations yielded similar results as with *mean* concentrations (not shown) as these exposure variables were strongly correlated within the same microenvironment (*r* > 0.75).

Urinary cotinine levels were assayed in samples used to measure uLTE_4_. Urinary cotinine levels were significantly related to same-day uLTE_4_ levels, but not to albuterol usage in models that did not include PM exposure variables. Adding same-day cotinine levels as a predictor to models with a pollution variable (either *mean* PM_1.5_ or an exposure *event* indicator) generally yielded positive but either marginally significant or insignificant relationships between cotinine and health outcomes (uLTE_4_ and albuterol) for each microenvironment, and the addition of cotinine did not substantially change the pollutant effects on these health outcomes.

## Discussion

Studies of air pollution effects on childhood asthma have classically utilized area-wide monitors as a surrogate for personal exposures [14, 15]. This approach assumed that exposures were relatively homogenous across the population of concern. However, spatial gradients in air pollution exposures have been demonstrated using both static measurements (i.e., distance to roadway) and location-activity data across the built environment (e.g., moving from indoors to outdoors) [16–18]. In this context, health effect studies have generally shown greater effects in populations who live closer to air pollution sources and have indirectly suggested (by multivariable regression analysis controlling for other covariates) that this proximity effect was a result of spatially heterogeneous exposures that dissipate as the distance from the source increases [19–21].

In this pilot study we utilized a novel approach to measure spatially and temporally heterogeneous exposures and their potential health effects on children with asthma. In this context, however, we characterized heterogeneity in exposure by quantifying personal exposure in different microenvironments. This approach was enabled by the use of wearable direct-reading sensors (PM, GPS, temperature) with a spatial/temporal algorithm. Furthermore, we analyzed our data using an *exposure event* variable, in addition to the traditional method of calculating mean exposures. This *exposure event* metric was defined as a change that begins and ends in the same microenvironment and thus more clearly segregates exposures by microenvironment. In contrast, *mean* or *maximum* exposure levels were more strongly correlated across microenvironments, especially across proximal microenvironments such as home-to-school and transit-to-school. Thus, the use of a *mean* exposure variable may partially reflect spatially homogenous exposures that occur across microenvironments from common sources (i.e., ambient, regional air pollution) or carryovers in exposure from one microenvironment to the next (i.e., the personal cloud effect). As such, the use of a *mean* exposure variable may be subject to confounding if used to examine health effect associations related to a given microenvironment. For these reasons, the *exposure event* variable may be better suited for microenvironmental studies of personal exposure/health.

Personal monitoring studies in children with asthma have been previously performed by our group and others using time-integrated approaches [11, 22]. These studies reported on the impact of measurement error on health effects when area-wide measurements are used as a surrogate for personal exposures to ambient air pollutants. This more traditional approach to personal monitoring (utilizing time-averaged, filter-based measurements integrated over 24 h) is useful for determining personal exposures to regional ambient PM concentrations since correlations between personal and central monitor levels strengthen over longer time frames [2, 3]. This integrated personal monitoring approach, however, is less sensitive to spatially or temporally heterogeneous exposures that occur over shorter time periods. These transient changes in exposures may be especially biologically relevant in the context of acute asthma worsening mediated by cysteinyl leukotriene release from mast cells. Cysteinyl leukotrines and other important asthma mediators are stored in preformed granules and released within 15 min of allergen exposure resulting in acute elevations in uLTE_4_ levels and early allergen-induced bronchoconstriction [23]. Similar to patterns after allergen exposure, maximum morning PM_2.5_ concentrations were associated with same-day increased uLTE_4_ levels within a few hours of maximum concentrations and dissipated by the following day [24]. This association was attenuated in children previously exposed to second hand smoke on the same day thus suggesting a relatively early homeostatic response that limits further responses to exposure over time [12, 25]. Together these data imply that, continuous exposure and health monitoring might be critical to study acute effects of cysteinyl leukotriene induced asthma worsening and that, in this context, the rate of *change* in exposure might be more biologically relevant than the absolute *level* of exposure over a given time period.

Although health effect studies using real-time personal monitoring and/or microenvironmental apportioning have been reported, there are important differences in the present approach compared to previous work. For example, Adar et al. [26] measured microenvironmental exposures for seniors riding on a group bus trip and evaluated associations between PM_2.5_ exposures and exhaled nitric oxide. These measures were group-wide (area monitoring) and did not account for personal exposure (between-subject). Delfino et al. [27] examined FEV_1_ exposure using a real-time monitor. Although they reported on exposures by time, they did not report associations by microenvironment (i.e., Delfino et al. tested various time lags from hourly to daily). In addition, they examined peak exposures but not the within-microenvironment increase variable described in the present study. In fact, we are unaware of any research that has used such metrics in the context of microenvironmental exposure and health outcomes.

Although previous research has reported on the health effects of PM exposure by microenvironment, these reports have inherent limitations compared to the approach used in the present study. These limitations include utilizing area-wide measurements [26] to attribute exposures to groups of individuals as they move between various microenvironments and reliance on time-activity diaries to determine exposure by microenvironment [27]. In contrast, the present study has utilized personal exposure monitoring (to capture inter-subject variability in exposure) in conjunction with automated [4, 28] microenvironment determination (to reduce participant burden and subject error from time-activity diaries) across a 24-h monitoring period.

The most important limitation of this study was its small sample size, which occurred primarily due to the increased resources required to perform personal sampling. Although this pilot study was somewhat underpowered to observe statistically significant health effects, the effect estimates suggest that microenvironment-specific exposures might result in clinically significant effects on asthma as health estimates were generally positive and consistent. Caution should be taken when applying these health effect estimates broadly, due to the relatively wide confidence intervals. Despite the small sample size, some health estimates (especially transit exposures) approached or attained statistical significance. There is also some suggestion that health effects to the same level of PM may vary by microenvironment as health effects were generally strongest for PM exposures occurring while in transit compared to home or school exposures. Such differential estimates might suggest PM sources or exposure patterns with different toxicities in different microenvironments. Unfortunately, because real-time measurement of PM source components were not available when the study was performed, we could not determine sources within microenvironments except for an assessment of the contribution from second hand smoke (via urinary cotinine). Other less substantial study limitations include the fact that some models using the *mean* metric were influenced strongly by outliers; however, this was not the case for the analyses using the *exposure event* metric. Although the use of light-scattering for estimating PM mass concentrations is subject to measurement error, [8, 9], we attempted to correct for such measurement error by normalizing the daily average mass concentration reported by the pDR to that of a filter sampler (located immediately downstream of the pDR sensing zone).

If confirmed in a larger study, these data would suggest that the amount of time spent in transit versus other microenvironments might be an important determinant of acute asthma worsening. Recent studies have reported that in-vehicle PM_2.5_ and NO_2_ concentrations consistently exceeded regional outdoor levels and that each unit increase in the rate of encountering diesel vehicles (count/min) was associated with a more than doubling increase in in-vehicle concentrations of ultrafine particles, black carbon, and PM_2.5_ as well as substantial increases (>15 %) in indoor/outdoor ratios [29]. Mirabelli et al. recently reported increased exhaled nitric oxide levels in adults with asthma after scripted rush-hour commutes [30]. These findings, along with results from the present study, support an examination of transit patterns in for children with persistent asthma and suggest that health improvements may accrue by attenuating transit exposures. In this context, a recent study by Wu et al. [31] demonstrated that the strongest predictors of personal exposure to polycyclic aromatic hydrocarbons in adult women were time spent in vehicles and, to a lesser extent, overall traffic density during transit. If exposure patterns in children are similar to adults, potential recommendations for improving health in children with persistent asthma might include minimizing time in vehicles (especially those with poor filtration systems) and avoiding transit on busy roads.

## Conclusion

This pilot study has demonstrated, as proof of concept, the utility of spatial-temporal personal exposure monitoring to measure PM exposures as they occurred within three characteristic microenvironments. The potential for clinically significant health effects from spatially/temporally heterogeneous exposures in children with asthma is also suggested although a larger study will be needed to confirm this potentially important relationship.

## Additional file

Additional file 1: Supplementary materials. (PDF 1699 kb)

## Abbreviations

FEV_1_: Forced expiratory volume in 1 second
GIS: Geographic information system
GPS: Global positioning system
hr0: Mean log PM_1.5_ concentration in the 60 minutes preceding urine collection
hr1: Mean log PM_1.5_ concentration for the 60 minutes ending 1 hour before urine collection
hr2: Mean log PM_1.5_ concentration for the 60 minutes ending 2 hours before urine collection
NJH: National Jewish Health
NO_2_: Nitrogen dioxide
pDR: Personal DataRAM model 1200
PM: Particulate matter
PM_1.5_: Particulate matter with aerodynamic diameter less than 1.5 microns
uLTE_4_: Urinary leukotriene E4
URI: Upper respiratory infection
