# Supplementary material for: Within-microenvironment exposure to particulate matter and health effects in children with asthma: a pilot study utilizing real-time personal monitoring with GPS interface
Source: Environ Health. 2016 Oct 10;15:96. doi: 10.1186/s12940-016-0181-5 (PMC5057244; doi:10.1186/s12940-016-0181-5)
Supplement: Supplementary file 1 — Supplementary materials. (PDF 1699 kb) [file 12940_2016_181_MOESM1_ESM.pdf]

Microenvironmental Exposure to Particulate Matter and Health Effects In Children with Asthma:

A Pilot Study Utilizing Real-Time Personal Monitoring with GPS Interface

Nathan Rabinovitch, Colby D. Adams, Matthew Strand, Kirsten Koehler, John Volckens

Supplementary Material

Exposure Assessment Protocol

Personal particulate matter (PM) exposures were assessed through space and time using mobile monitoring backpacks that were worn by students each day. Students were instructed to wear these backpacks as much as possible throughout the day and to place them by their bed at night. Monitoring backpacks contained an aerosol nephelometer (personal DataRAM 1200, or 'pDR', Thermo-Fisher Scientific Inc., Waltham, MA) to measure fine particle mass concentration, a global positioning system (GPS) receiver (GPSMap 60Cx, Garmin Inc. Olathe KS) to record geographic position data, and a temperature sensor (Thermo Record TR-52, T and D Inc., Saratoga Springs, NY) to record ambient temperature within the child's breathing zone. The monitors recorded data at 10-second intervals. A time integrated filter sample (Teflo 37mm, Pall Inc., Ann Arbor, MI) was also collected immediately downstream of the nephelometer each day. Flow through the sampling train was maintained with a calibrated personal sampling pump (6.8 L/min flow, Omni Personal Pump, BGI Inc., Waltham MA); a size selective cyclone (1.5 μm size cut, Model GK2.05, BGI Inc., Waltham MA) was placed at the inlet to limit the measurement to fine PM. Particulate mass collected on the filter was weighed to the nearest

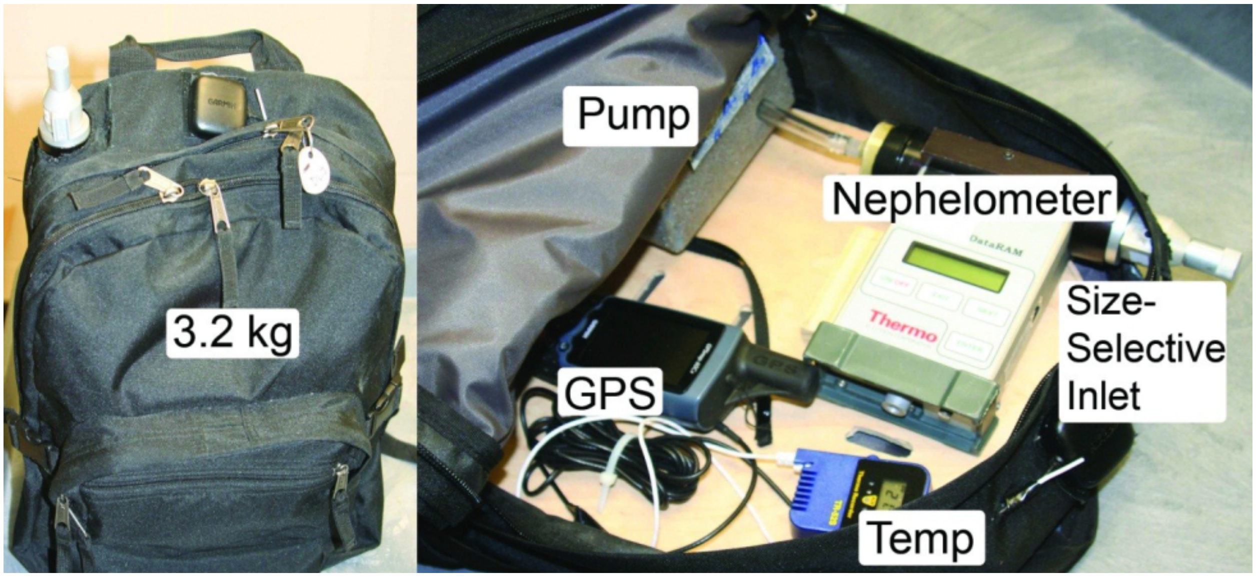

Figure S1. Personal Sampling Backpack

microgram; this measurement was used to standardize the instrument's readings, as recommended to improve instrument accuracy (1).

The total weight of the backpack apparatus was 3.2 kg (7 lbs). The air sampling inlet was positioned 2 inches above the top surface of the backpack, slightly to the rear of the child's left shoulder. This inlet location was chosen to capture a breathing zone sample, defined as the envelope around the head that is considered to have the same concentration of pollutant as the air breathed in by the person. An image of the sampling backpack and associated equipment is shown in Figure 1. The rear pockets on each backpack contained the sampling equipment (Figure 1, right panel); the intermediate and front pockets of each backpack (Figure 1; left panel) were made available for students to carry books and school supplies.

The following steps were performed to prepare the sampling apparatus for use each day: 1) A pre-weighed filter was placed downstream of the pDR sensor chamber; 2) pump flowrate was calibrated; 3) the GPS receiver was powered on and allowed to acquire signal lock on at least four satellites; 4) the internal clocks of the pDR and TR-52 were synchronized to the clock of the GPS Receiver; 5) the data-logging memory of each monitor was reset; 6) the monitoring equipment and pump were secured to an internal custom frame (Figure S1, right panel); 7) sampling hoses were connected and sampling equipment was placed in backpack; 8) monitors were activated (i.e., data-logging was initiated) and the sampling pump was turned on. The downtime for these procedures lasted approximately 3 hours each day (all within the school microenvironment, after the collection of $uLTE_4$), so each child carried the backpack for approximately 21 of the 24 hours in a day.

At the end of each monitoring day, the backpack was collected from each child and sampling equipment was inspected, pump flow was re-calibrated, and then each monitor was de-activated. Subjects were then surveyed about behaviors (e.g. mode of transport during commute), and potential household exposure sources (cigarette smoke, cooking) from the previous 24 hours. Data from each monitor were then downloaded to a personal computer. Air sampling filters were removed, labeled, and then equilibrated at room temperature for 24 hours prior to gravimetric analysis. Data from the pDR, GPS receiver, and temperature monitor were collated into a database by matching the associated timestamps from each instrument, thereby integrating the data into a common array.

Data Post Processing

To include 10-sec personal $PM_{1.5}$ readings that were assigned zero values by the instrument (i.e., values lower than the instrument detection limit of 1 $\mu g/m^3$); an imputed value was substituted for the zero reading. This substitution was performed prior to (and necessary for) log-transformation of the data set. Imputed values were created first by stratifying the dataset by subject. Next, zero values for each subject were replaced with a heuristic value equal to one half of the smallest concentration recorded by the pDR (0.5 $\mu g/m^3$). Geometric means and geometric

standard deviations of the log-transformed stratified data subsets were then calculated. These distribution parameters were used to impute values for the original zero readings via a probability integral transformation (2). The imputed, or modeled, values were then substituted for the original zero readings. These data were then log-transformed and aggregated up to the microenvironmental level. Multiple imputation procedures were performed and the outputs were evaluated for consistency; the average change in the mean values of the full dataset was less than 0.4%.

Time-Activity Apportionment

The microenvironmental apportionment methodology has been published (3), however, a brief description follows. Collected exposure data was post-processed using a temporal spatial algorithm that apportioned morning data into pre-determined location-activity categories (e.g., home, transit, school). Each 10-second data point was assigned a specific location-activity category (home, morning transit, school) using geographic proximity analyses supported by time-based rules and temperature measurement – the latter used to confirm when a subject passed from indoors to outdoors. The geographic proximity analysis determined if a recorded point lied within a predefined, two-dimensional area (i.e., a home boundary). The time-based rules further supported the proximity analysis by establishing expected times for the individual to be in the home or work/school area. For example, if the recorded position of a sample was within a certain radius of the work/school position (e.g., 50 m) during expected work/school hours then the exposure was assigned to the work/school category. Similarly, the home category was assigned if the recorded position of the sample was within the defined home area during expected home hours. If the recorded position of the sample was neither at home or work/school, the sample was considered in-transit.

Models and interpretations of effect estimates

Models for LTE_4 take the form

$$ln(Y_{ij}) = \beta_0 + \beta_1 x_{ij} + \beta_2 temp_{ij} + \beta_3 cold_{ij} + b_i + \varepsilon_{ij},$$

where Y is LTE_4, x is the pollutant variable of interest, and i and j index subject and time, respectively. In the model, it is assumed that $\varepsilon_{ij} \sim N(0, \sigma_\varepsilon^2)$, independent of the random intercept $b_i \sim N(0, \sigma_b^2)$. Additionally, errors ε_{ij} are assumed to follow a spatial power structure (i.e., an AR(1) structure that accounts for intermittent responses) within subjects, and independent between subjects;. Due to taking the natural log of LTE_4, e^{β_1} is the multiplicative increase in the mean of Y for a 1-unit increase in x, while $100(e^{\beta_1} - 1)\%$ is the relative increase in the mean of Y for a 1-unit increase in x.

The generalized linear model for albuterol use counts take the form

$$ln(\mu_{ij}) = \beta_0 + \beta_1 x_{ij} + \beta_2 temp_{ij} + \beta_3 Friday_{ij}$$

where μ_{ij} is the mean albuterol use for subject *i* at time *j*, *Friday* is an indicator for that day of the week. (On Monday through Thursday children received an albuterol pre-treat before exercising, but not on Fridays because they did not have a physical education class that day.)

The model was fit using generalized estimating equations, using an AR(1) working correlation structure to account for repeated measures. Due to the use of the natural log link, e^{β_1} will also have a multiplicative increase interpretation, although in this case the natural log is applied to the mean of outcome rather than the outcome itself.

Construction of increase and exposure event variables

Initially, exposure patterns within each sample-day (n=125) were examined individually by 2 of the co-authors (MS and JV) to determine, qualitatively, whether a noticeable increase in PM exposure occurred within a given microenvironment. We then more formally constructed *increase* and *exposure event* variables to capture concentration spikes within microenvironments. The initial qualitative approach was very consistent with the more quantitative metrics (92% agreement with exposure events of at least $5\mu g/m^3$ and 88% agreement with exposure events of at least $10\mu g/m^3$). Due to the better fits in health outcome models for $5\mu g/m^3$, we chose to primarily focus on that cut-point in the manuscript.

References Cited:

1. Benton-Vitz K, Volckens J. Evaluation of the pdr-1200 real-time aerosol monitor. *J Occup Environ Hyg* 2008;5:353-359.
2. Casella G, Berger RL. Statistical inference. Duxbury Pacific Grove, CA; 2002.
3. Adams C, Riggs P, Volckens J. Development of a method for personal, spatiotemporal exposure assessment. *J Environ Monit* 2009;11:1331-1339.

TABLE S1: DESCRIPTIVE STATISTICS FOR MEAN AND 1-MINUTE MAXIMUM $PM_{1.5}$ VARIABLES USED IN ANALYSIS.

Microenvironment or time frame	Within-subject-sample variable	Mean	Min value	25th Quantile	Median	75th Quantile	Max value
Home	Average	4.2	0.2	1.8	4.4	11.5	103.5
Transit		3.7	0.1	1.8	4.7	8.5	56
School		2.8	0.1	1.4	3.2	5.6	23.9
24-hr		4.3	0.2	2.6	4.3	8.3	29.8
Home	1-minute maximum	12.0	0.3	5.5	10.8	31.8	295.1
Transit		9.9	0.2	4.8	10.1	25.9	275.5
School		8.1	0.2	3.9	7.8	18.5	151.7

Statistics were computed by first determining the mean or 1-minute averaged maximum of logged concentrations within subject samples, then determining composite statistics across subject samples, then exponentiating. Units are micrograms per cubic meter.

TABLE S2. ASSOCIATIONS BETWEEN HEALTH OUTCOMES (LTE_4 AND ALBUTEROL USAGE) AND MEAN PERSONAL EXPOSURE TO $PM_{1.5}$ AS MEASURED BY THE PERSONAL MONITOR.

Micro-environment	Lag	% increase per IQR increase in pollutant (95% CI)	
		**Albuterol usage**	**LTE_4**
Home	0	-0.8 (-12.1, 11.9)	7.3 (-5.9, 22.4)
	1	5.7 (-12.1, 27.1)	-8.4 (-22.7, 8.6)
	2-day	3.3 (-9.4, 17.7)	2.1 (-8.8, 14.2)
Transit	0	4.1 (-6.1, 15.5)	10.7 (-0.4, 22.9)
	1	12.0 (-2.0, 28.0)	6.2 (-5.9, 19.8)
	2-day	9.3 (-0.2, 19.8)	9.3 (0.3, 19.2)
School	0	3.8 (-7.0, 15.7)	5.3 (-6.7, 18.8)
	1	4.7 (-10.7, 22.7)	12.6 (-5.0, 33.6)
	2-day	6.5 (-7.0, 22.0)	9.6 (-2.1, 22.7)

For 2-day moving average estimates, records were weighted by number of records used in the moving average (1 or 2). For Albuterol usage models, temperature and Friday indicator covariates were used; for LTE_4 models, temperature and cold indicator were used (the lag for cold was set as the same for the air pollution variable). IQR for 0 and 1 day lags were 1.84, 1.39 and 1.54 for home, school and transit, respectively (for 2-day average they were 1.72, 1.36 and 1.47). 30 subjects were available for analysis; number of records used for model fits were: 114, 80 and 143 for 0, 1 and 2-day moving averages for albuterol usage, respectively, and 80, 58 and 111 for LTE_4. For more detail, see the text.

Figure S2: Percent increase in $uLTE_4$ per IQR increase in *mean* personal exposure to $PM_{1.5}$ by home, a.m. transit, and school microenvironments for same day (lag 0), one-day lag, and a 2-day moving average. Error bars represent 95% confidence intervals.

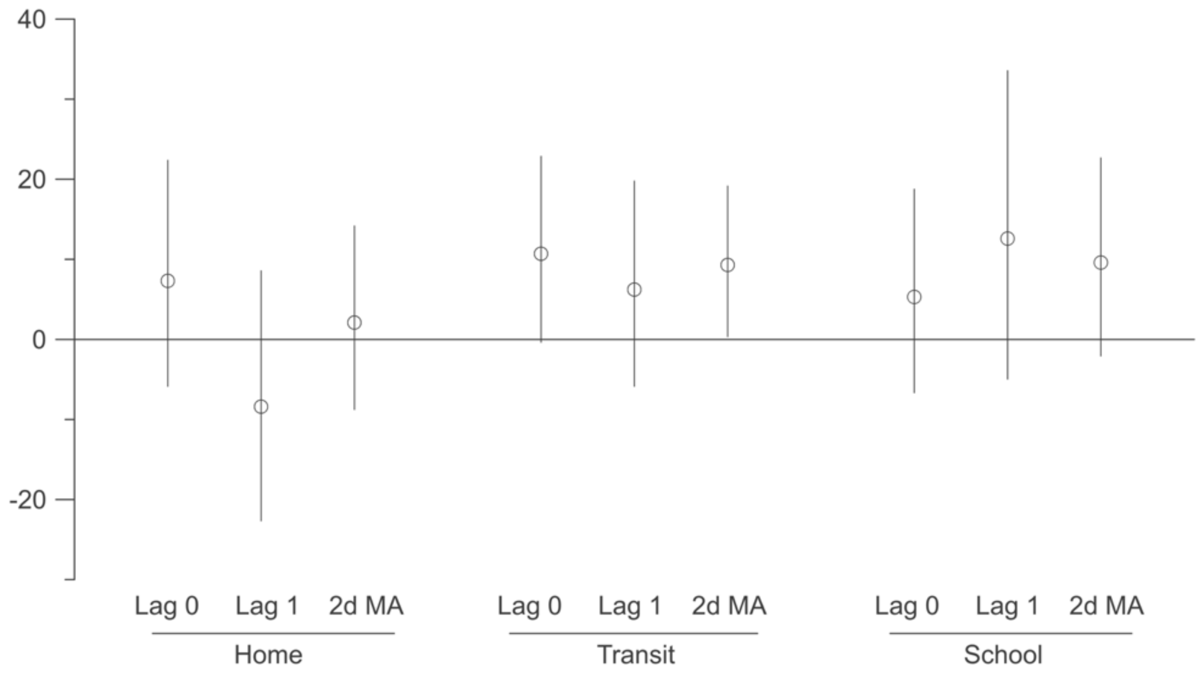

Figure S3: Percent increase in school-day albuterol usage per IQR increase in *mean* personal exposure to $PM_{1.5}$ by home, a.m. transit, and school microenvironments for same day (lag 0), one-day lag, and a 2-day moving average. Error bars represent 95% confidence intervals.

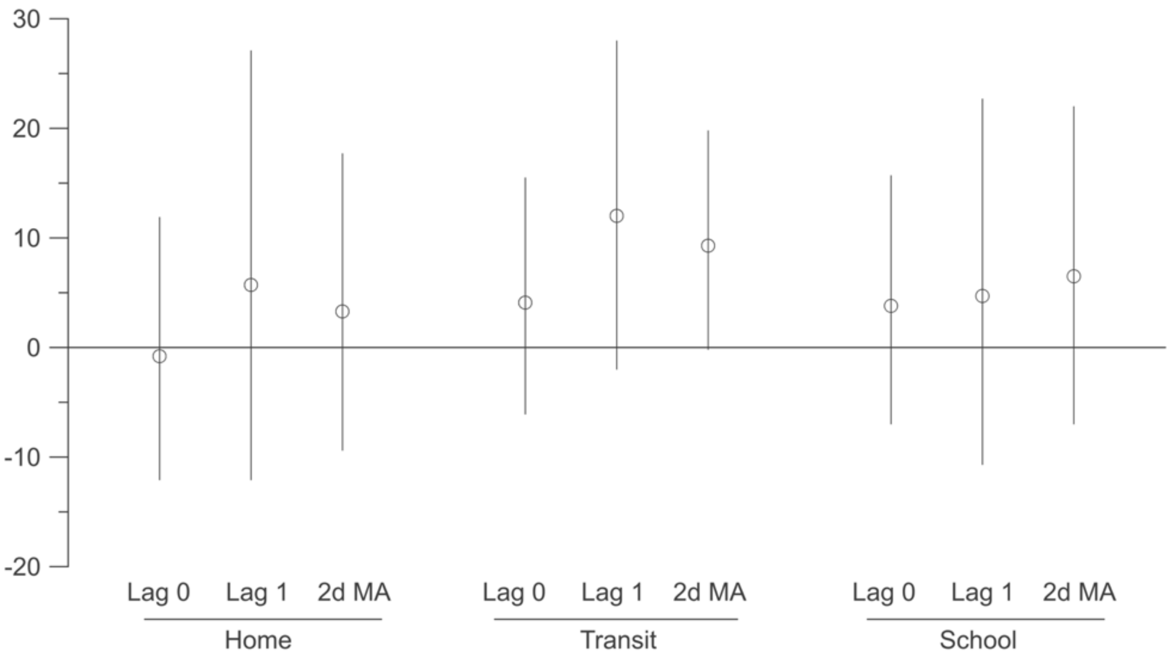

TABLE S3. COMPARISON OF AIC (FOR LTE_4) OR QICu (FOR ALBUTEROL USAGE) BETWEEN MODELS. THE BEST FIT (LOWEST AIC OR QICu) WITHIN OUTCOME, MICROENVIRONMENT AND LAG IS BOLDED. THE 5µg/m^3 EVENT INDICATOR PROVIDED THE BEST FIT MOST OFTEN AMONG METRICS.

Outcome	Micro-environment	Metric*	Lag 0 (n=80, LTE4; n=114, alb.)	Lag 1 (n=58, LTE4; n=80, alb.)	2-day MA (n=111, LTE4; n=143, alb.)
LTE4	Home	Mean	136.2	107.6	168.1
		1-min max	136.4	108.4	168.2
		5µg/m^3 event	133.7	**106.4**	166.2
		10µg/m^3 event	**132.8**	106.8	**165.2**
		Increase amount	135.0	108.2	166.5
	Transit	Mean	133.8	108.3	164.4
		1-min max	136.5	108.5	168.0
		5µg/m^3 event	**131.0**	**104.8**	**164.0**
		10µg/m^3 event	134.2	106.5	166.2
		Increase amount	135.4	108.1	167.5
	School	Mean	136.2	**106.3**	**165.2**
		1-min max	133.4	107.9	165.4
		5µg/m^3 event	134.2	107.0	**165.2**
		10µg/m^3 event	**132.7**	106.9	165.3
		Increase amount	134.7	109.0	167.6
Albuterol usage	Home	Mean	737.4	401.3	826.8
		1-min max	734.2	398.2	821.3
		5µg/m^3 event	**734.1**	404.3	822.9
		10µg/m^3 event	741.2	**396.7**	814.8
		Increase amount	731.2	396.9	**813.4**
	Transit	Mean	728.15	396.0	812.9
		1-min max	728.5	396.9	**811.2**
		5µg/m^3 event	**727.55**	**394.6**	814.0
		10µg/m^3 event	733.7	396.3	813.3
		Increase amount	728.8	400.8	817.1
	School	Mean	**739.0**	398.7	822.7
		1-min max	752.5	**398.5**	826.9
		5µg/m^3 event	752.1	399.8	**815.6**
		10µg/m^3 event	742.6	**398.5**	817.4
		Increase amount	751.2	399.3	828.5

*Continuous variables (Mean, 1-min max, Increase amount) all analyzed on the log scale.

Figure S4 (beginning on the following page): Profiles of 1-minute averaged $PM_{1.5}$ across 3 microenvironments: home (to left of vertical dashed bars), transit (in between bars) and school (to right of bars) by subject and date. $PM_{1.5}$ exposure data were computed by averaging logged concentrations by minute, then exponentiating.

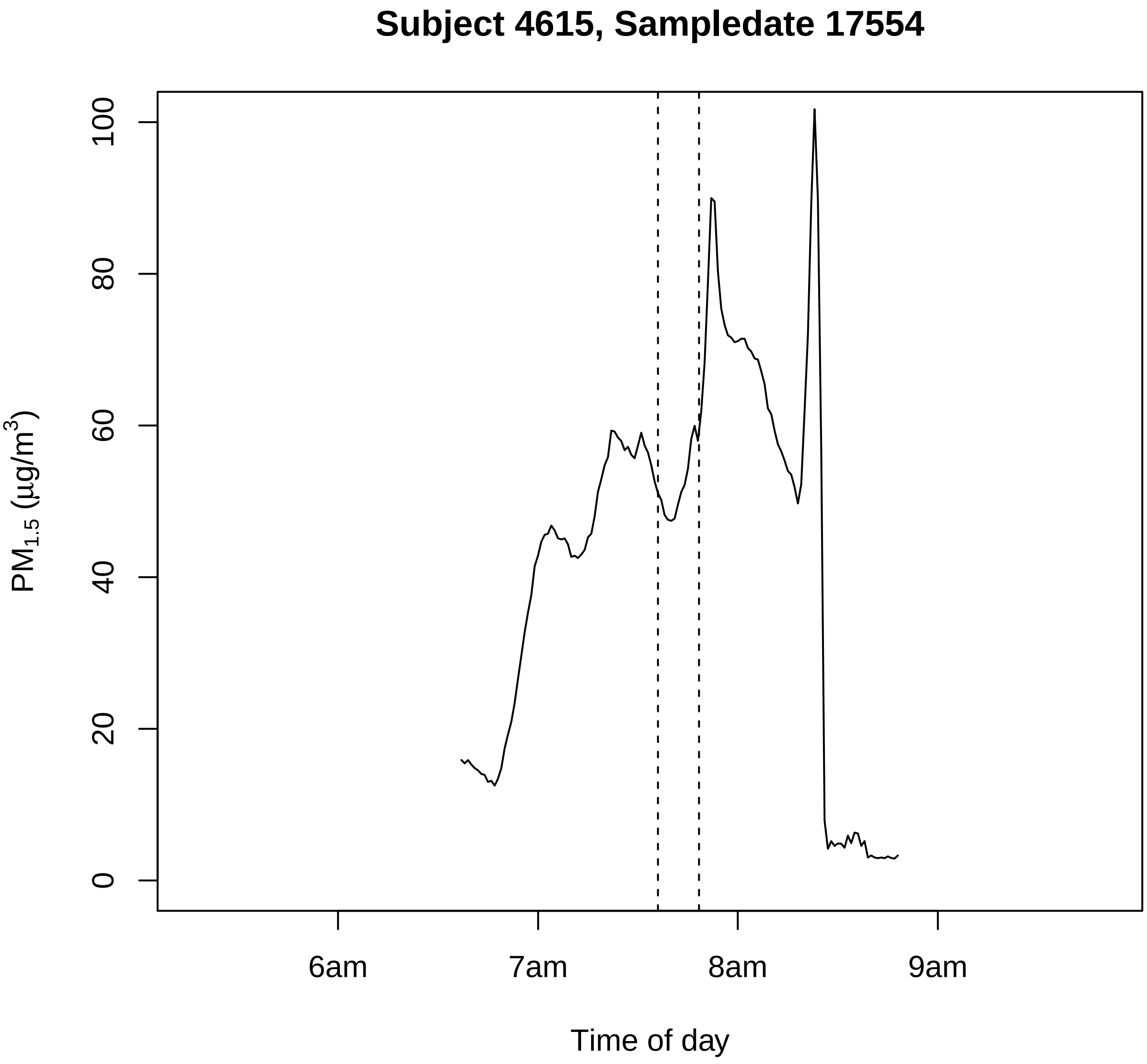

Subject 4615, Sampledate 17554
$PM_{1.5}$ (µg/m^3)
0
20
40
60
80
100
6am
7am
8am
9am
Time of day

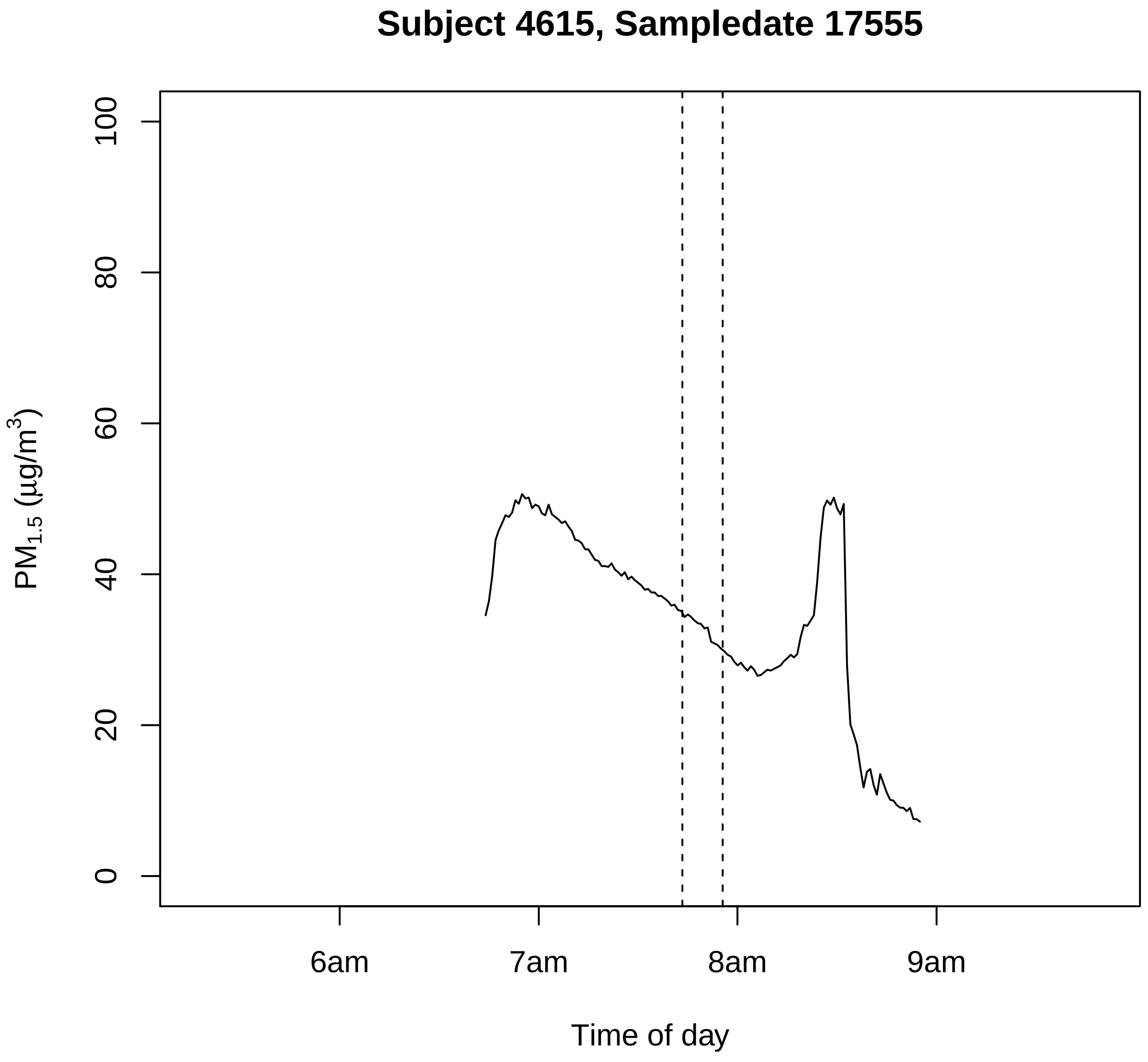

Subject 4615, Sampledate 17555
$PM_{1.5}$ (µg/m^3)
0
20
40
60
80
100
6am
7am
8am
9am
Time of day

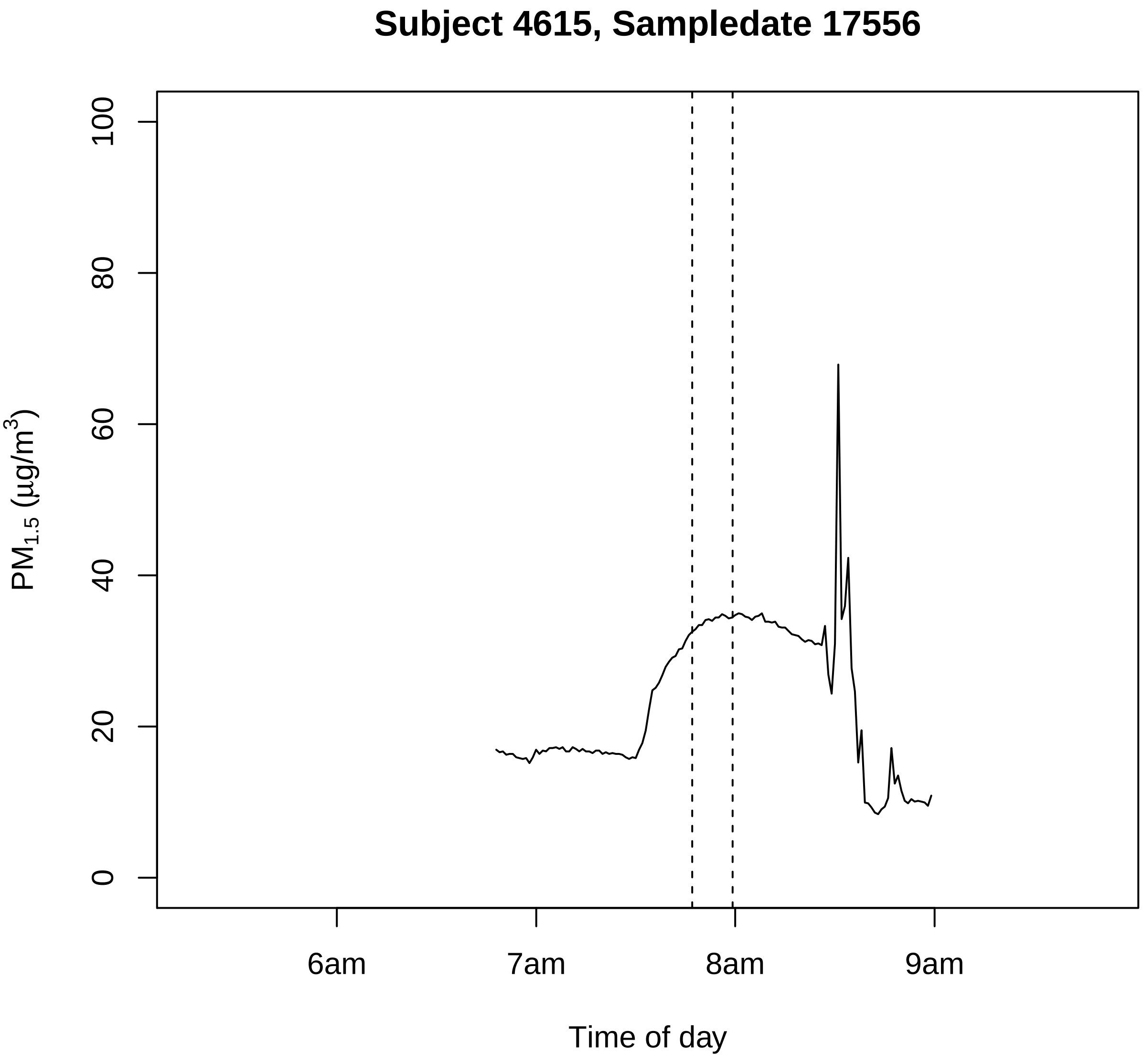

Subject 4615, Sampledate 17556
100
80
60
40
20
0
PM1.5 (µg/m3)
6am
7am
8am
9am
Time of day

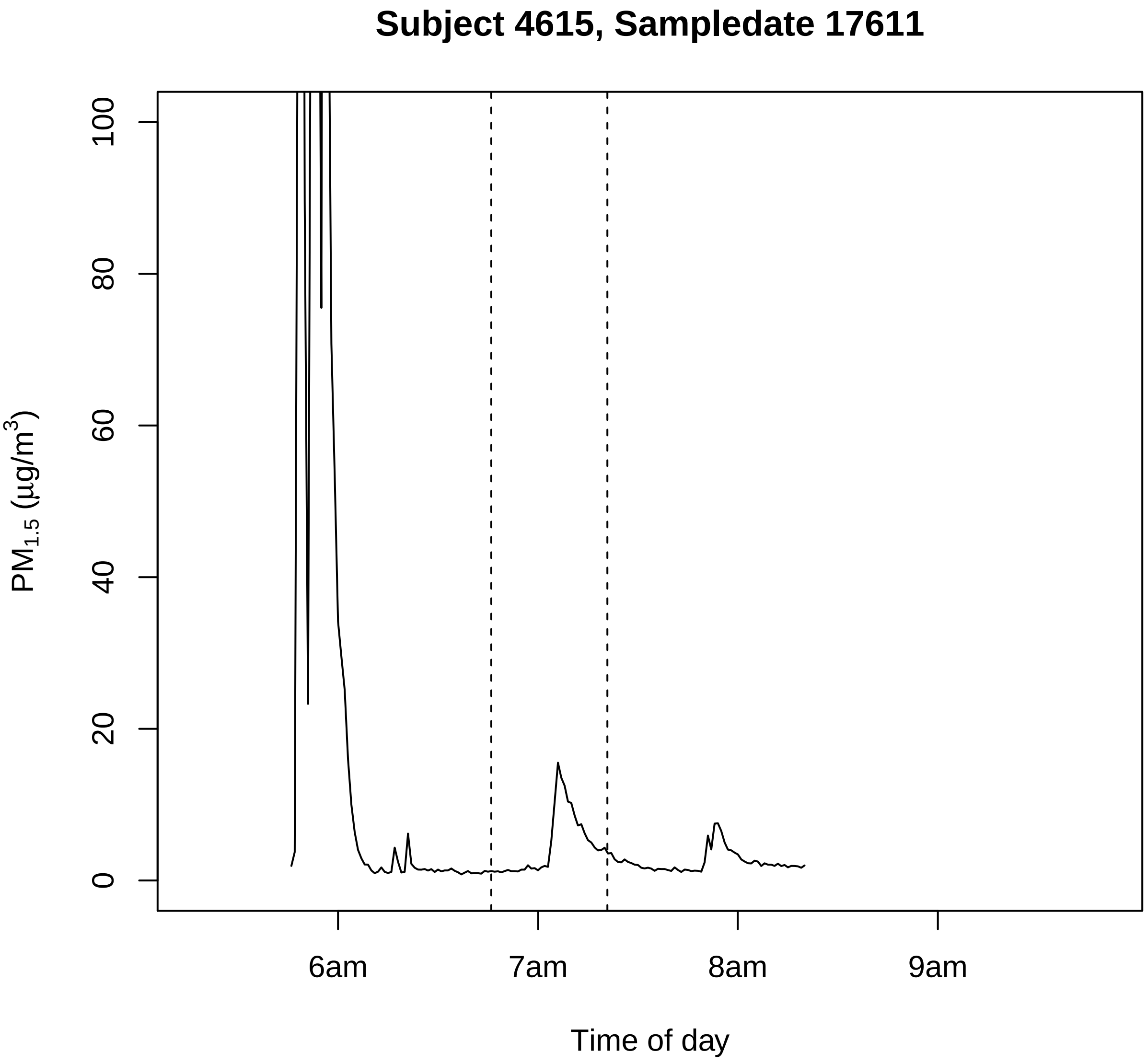
Subject 4615, Sampledate 17611
PM1.5 (µg/m3)
0
20
40
60
80
100
6am
7am
8am
9am
Time of day

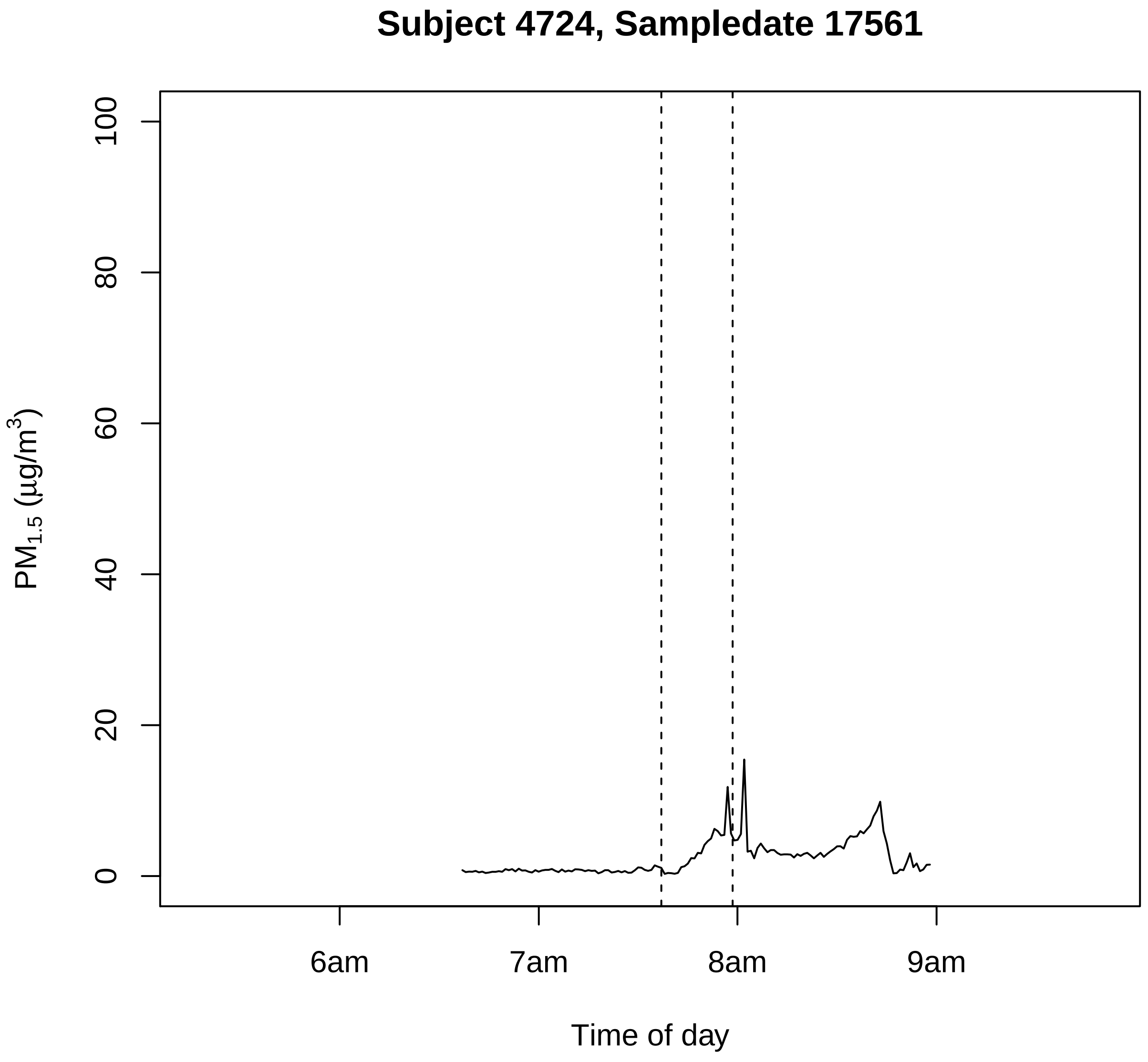

Subject 4724, Sampledate 17561
PM1.5 (µg/m3)
0
20
40
60
80
100
6am
7am
8am
9am
Time of day

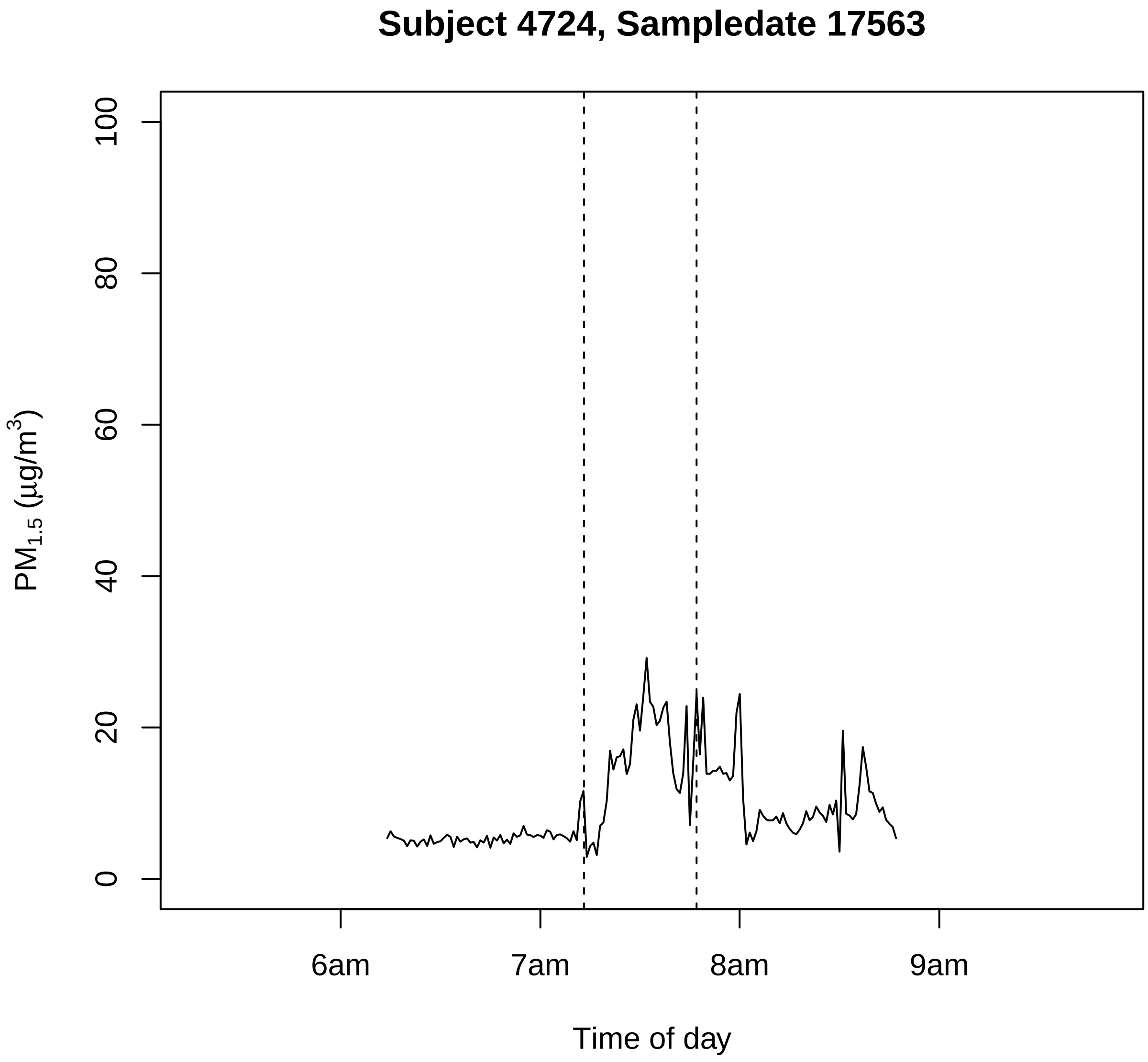
Subject 4724, Sampledate 17563
PM1.5 (µg/m3)
0
20
40
60
80
100
6am
7am
8am
9am
Time of day

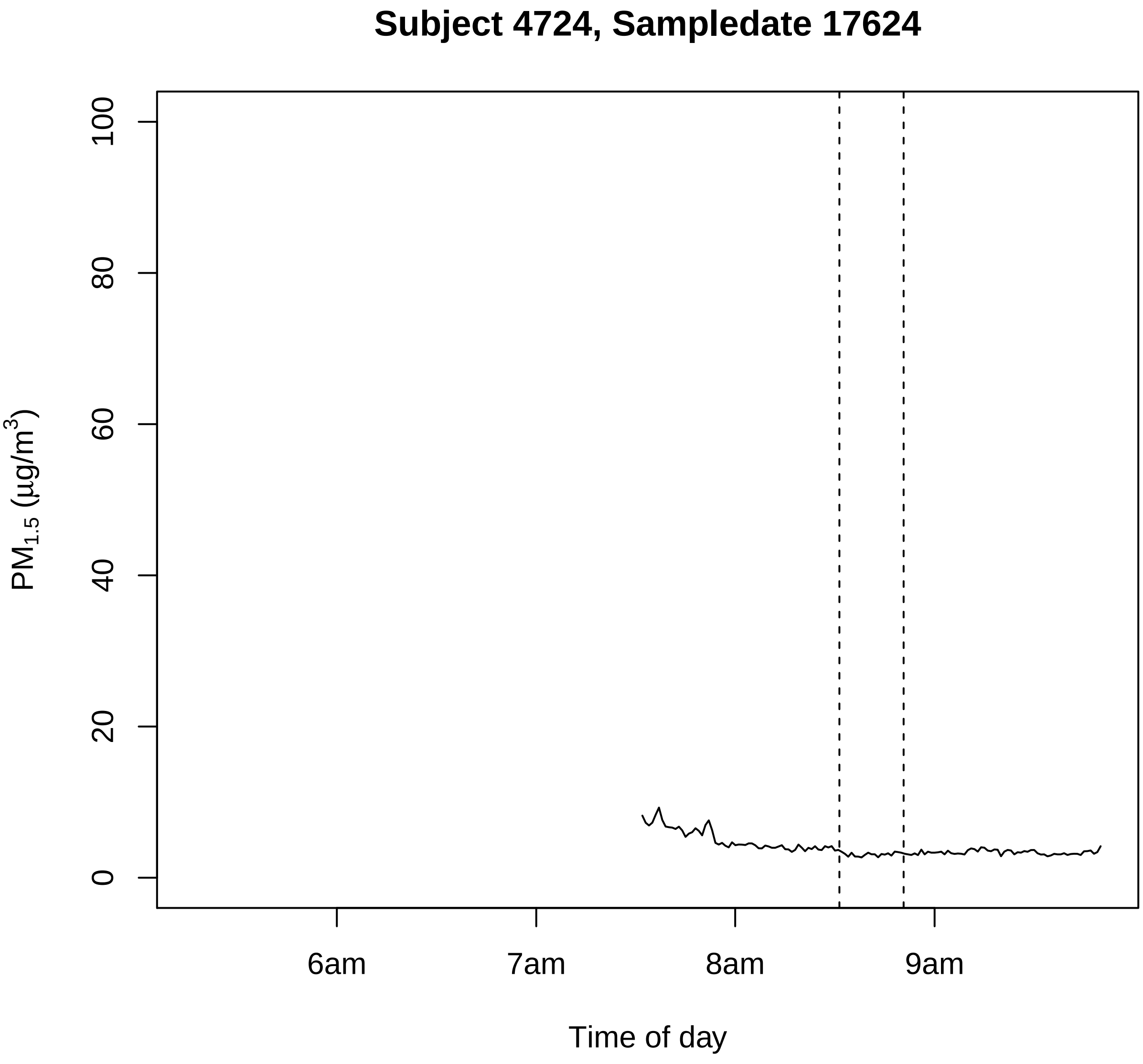

Subject 4724, Sampledate 17624
100
80
60
40
20
0
PM1.5 (µg/m3)
6am
7am
8am
9am
Time of day

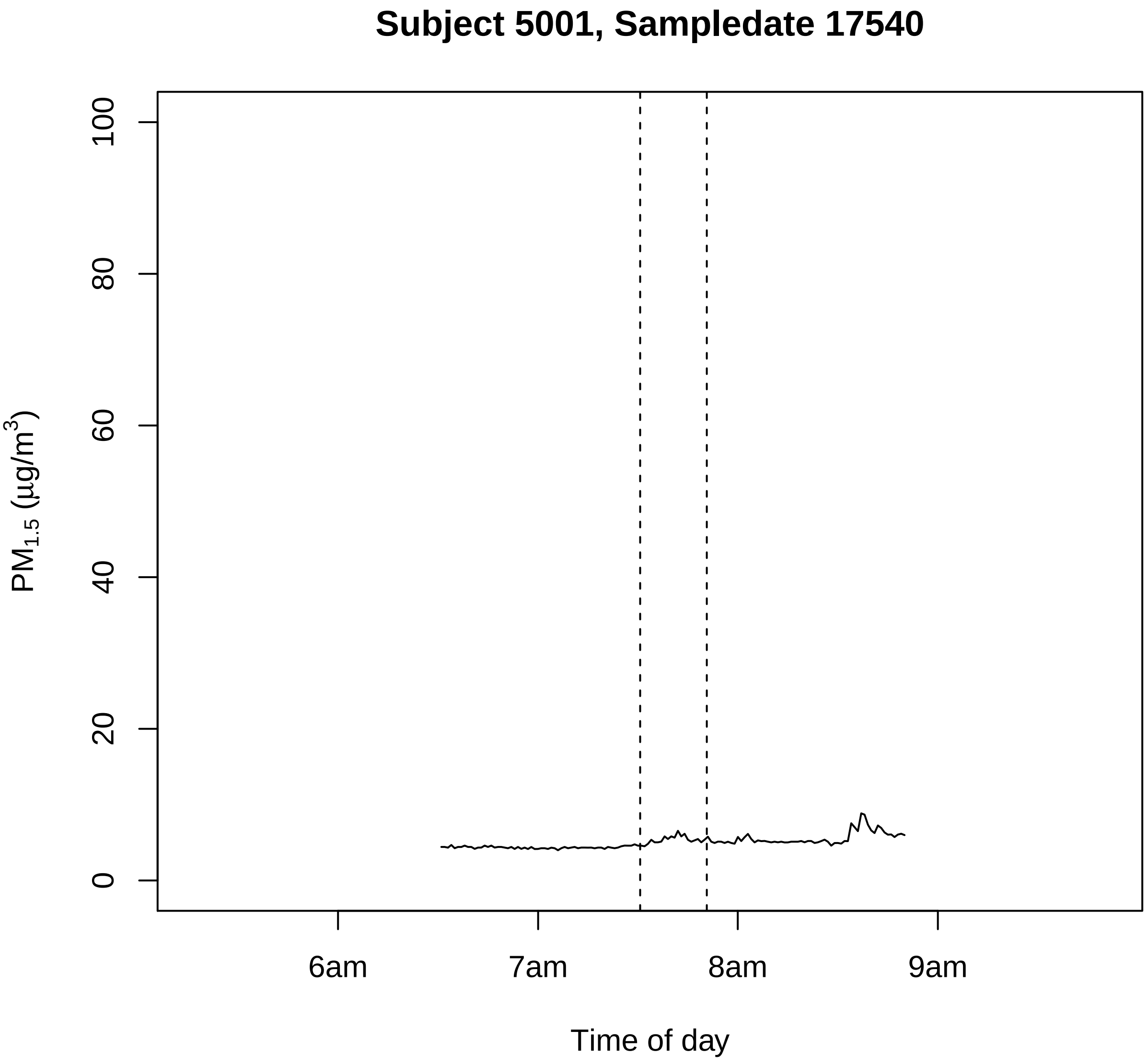
Subject 5001, Sampledate 17540
$PM_{1.5}$ (µg/m^3)
0
20
40
60
80
100
6am
7am
8am
9am
Time of day

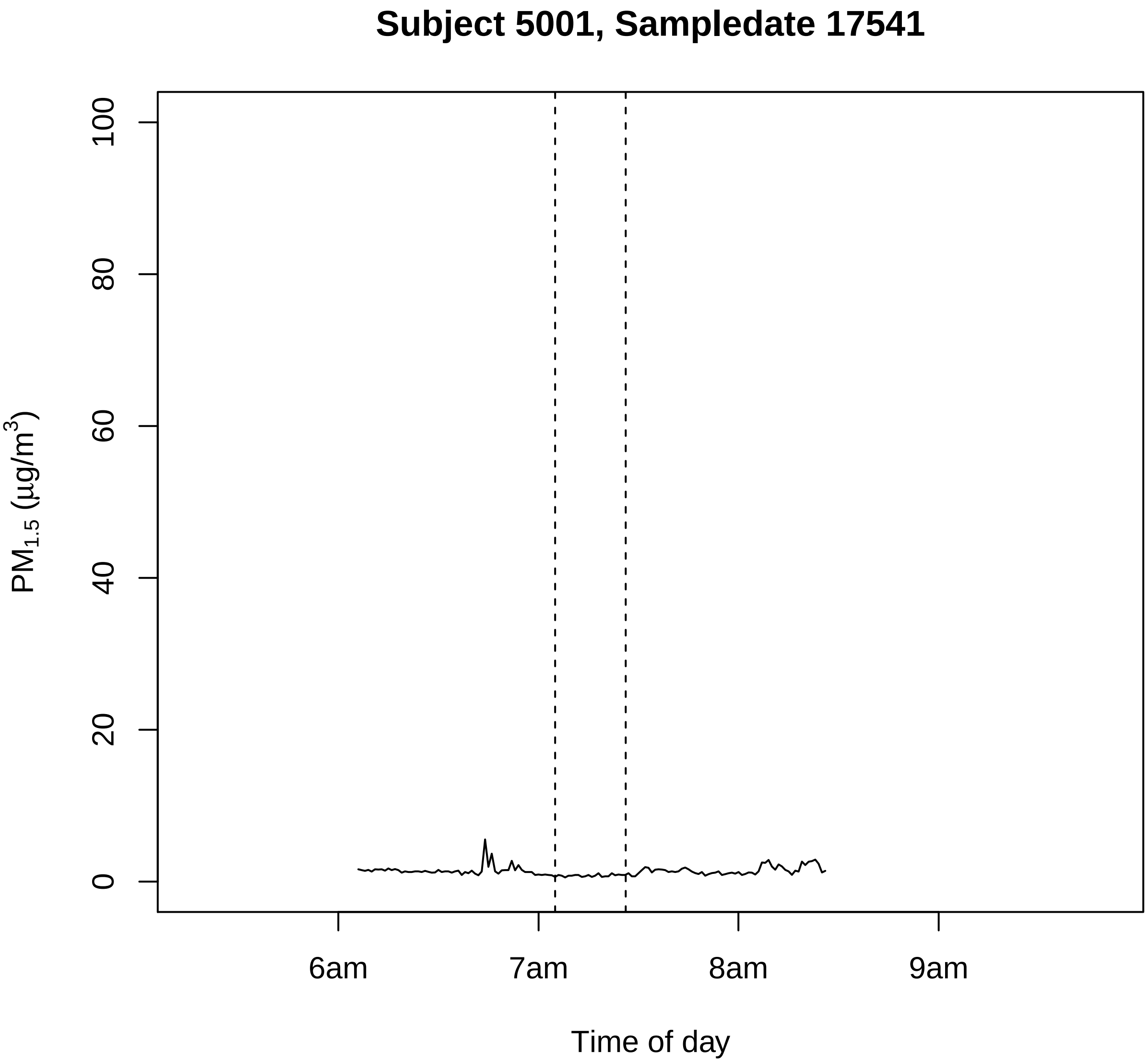
Subject 5001, Sampledate 17541
$PM_{1.5}$ (µg/m^3)
0
20
40
60
80
100
6am
7am
8am
9am
Time of day

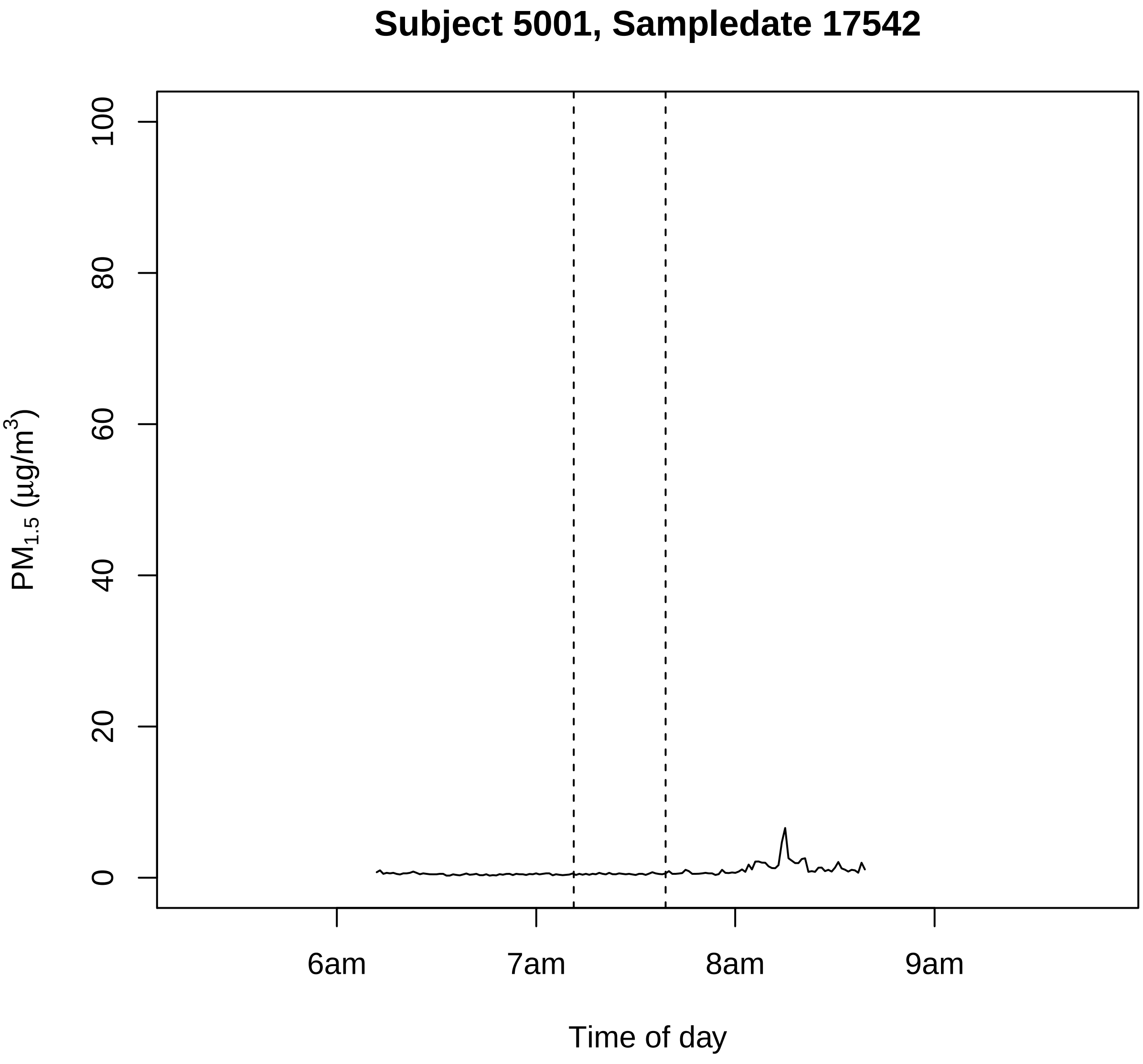
Subject 5001, Sampledate 17542
PM1.5 (µg/m3)
0
20
40
60
80
100
6am
7am
8am
9am
Time of day

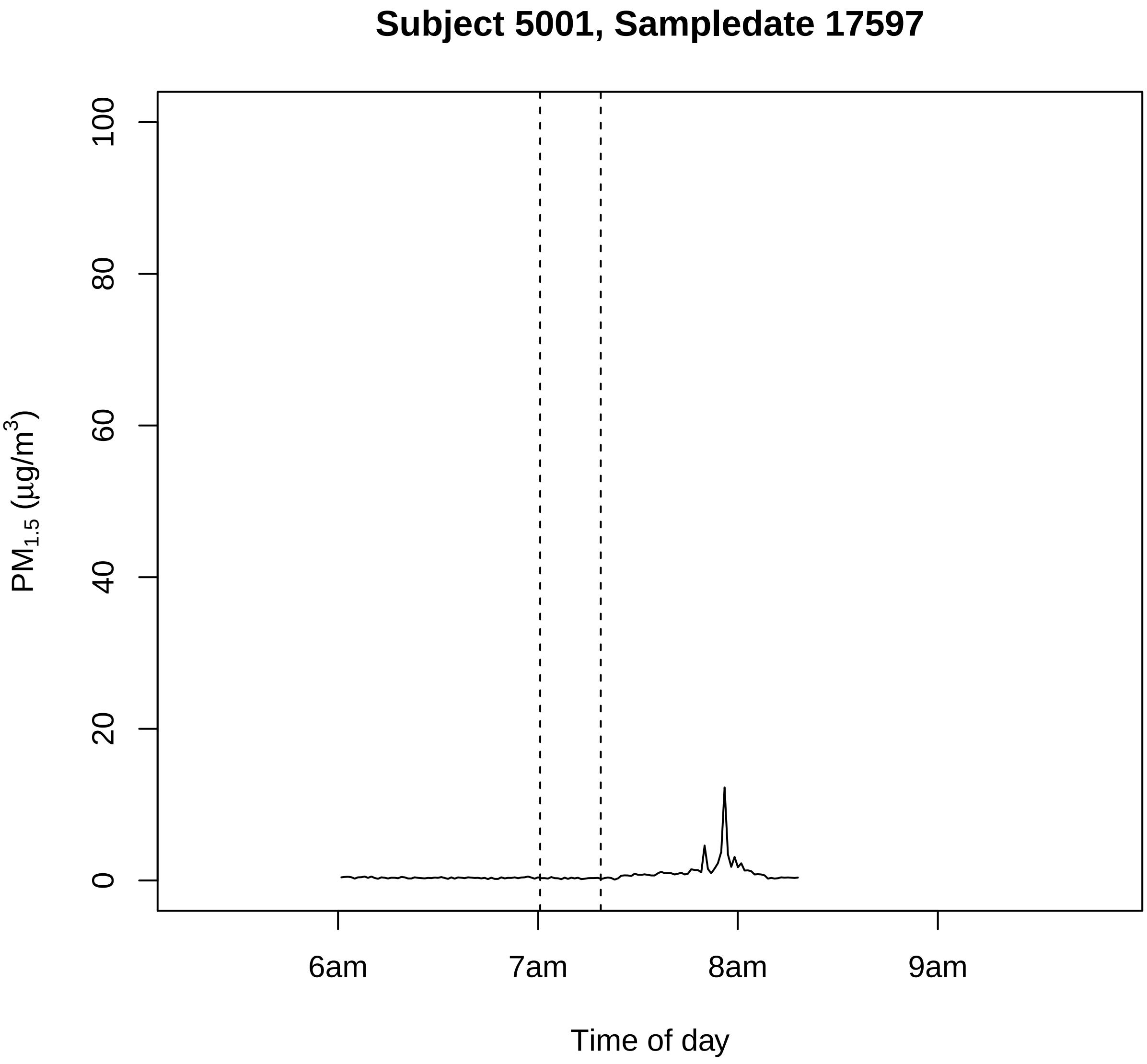

Subject 5001, Sampledate 17597
PM1.5 (µg/m3)
0
20
40
60
80
100
6am
7am
8am
9am
Time of day

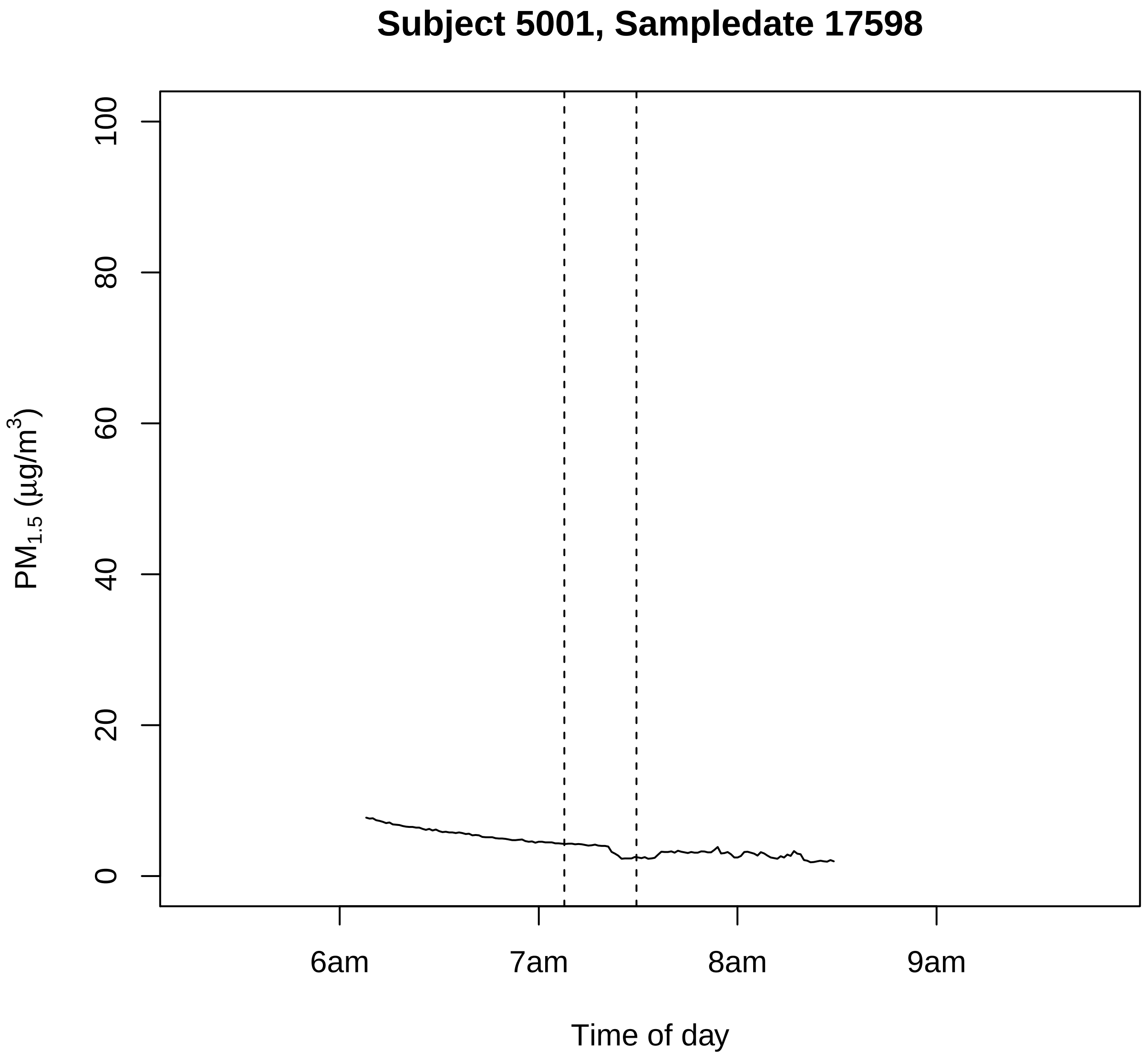
Subject 5001, Sampledate 17598
$PM_{1.5}$ (µg/m^3)
0
20
40
60
80
100
6am
7am
8am
9am
Time of day

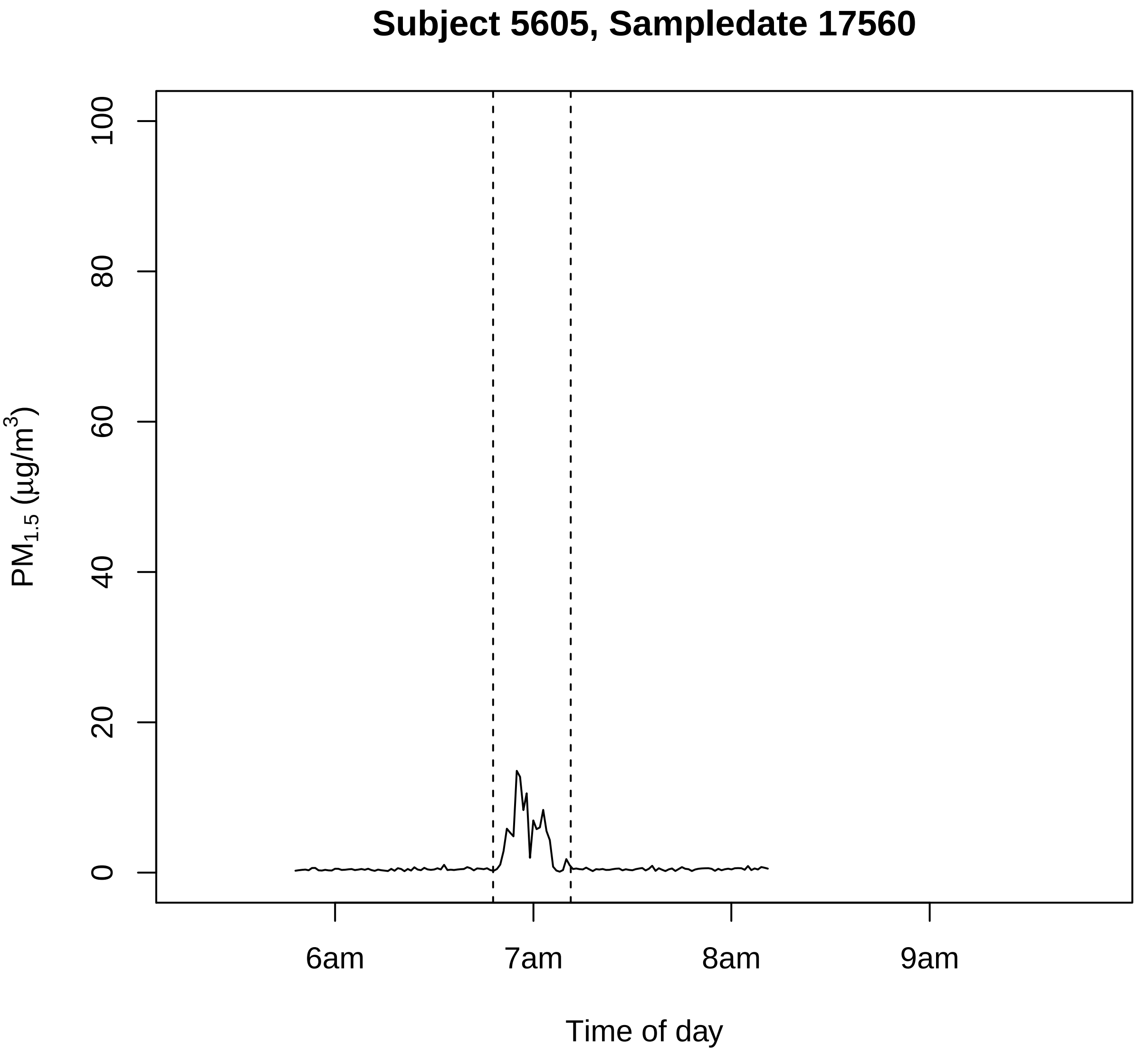

Subject 5605, Sampledate 17560
PM1.5 (µg/m3)
0
20
40
60
80
100
6am
7am
8am
9am
Time of day

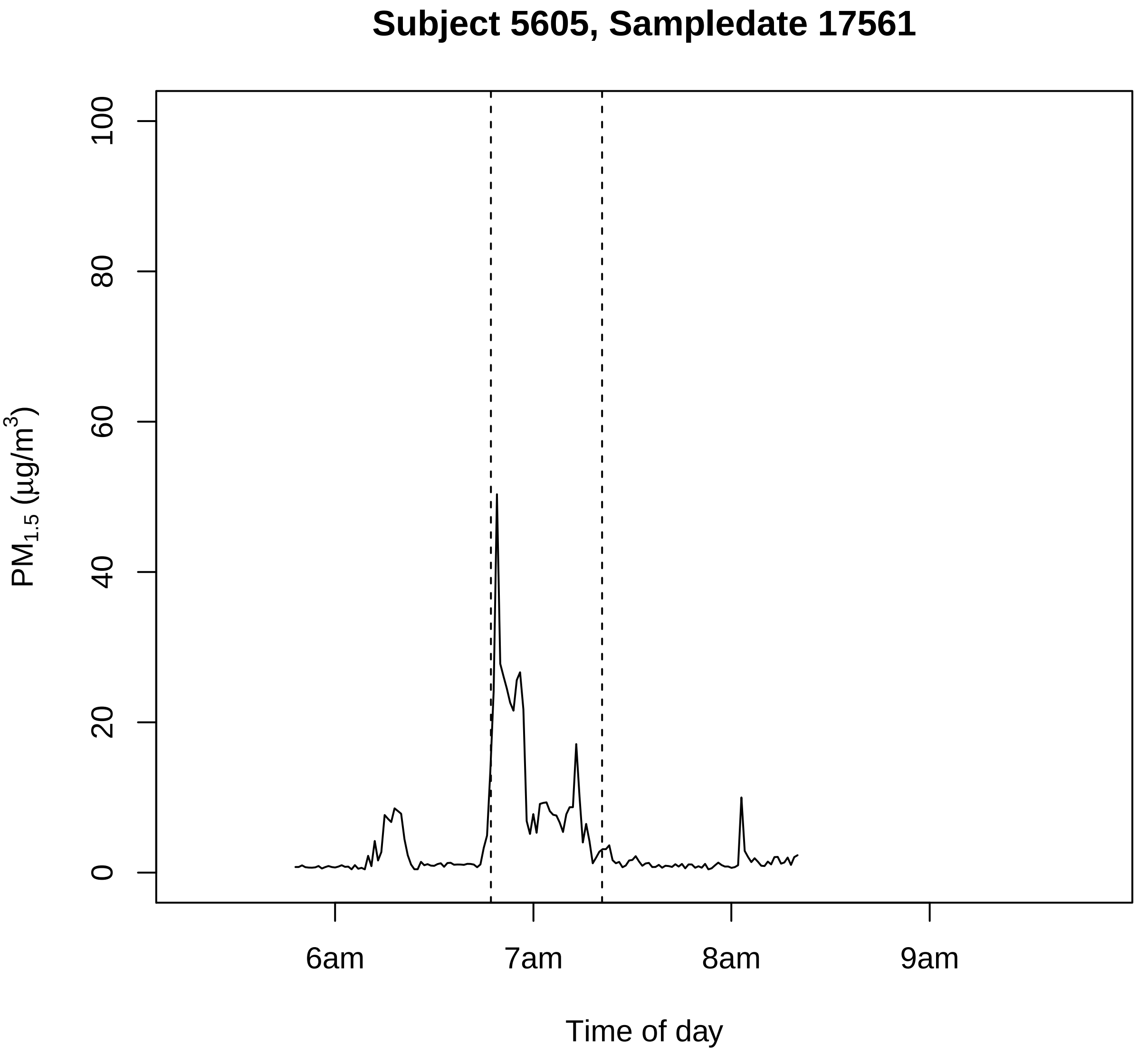
Subject 5605, Sampledate 17561
PM1.5 (µg/m3)
0
20
40
60
80
100
6am
7am
8am
9am
Time of day

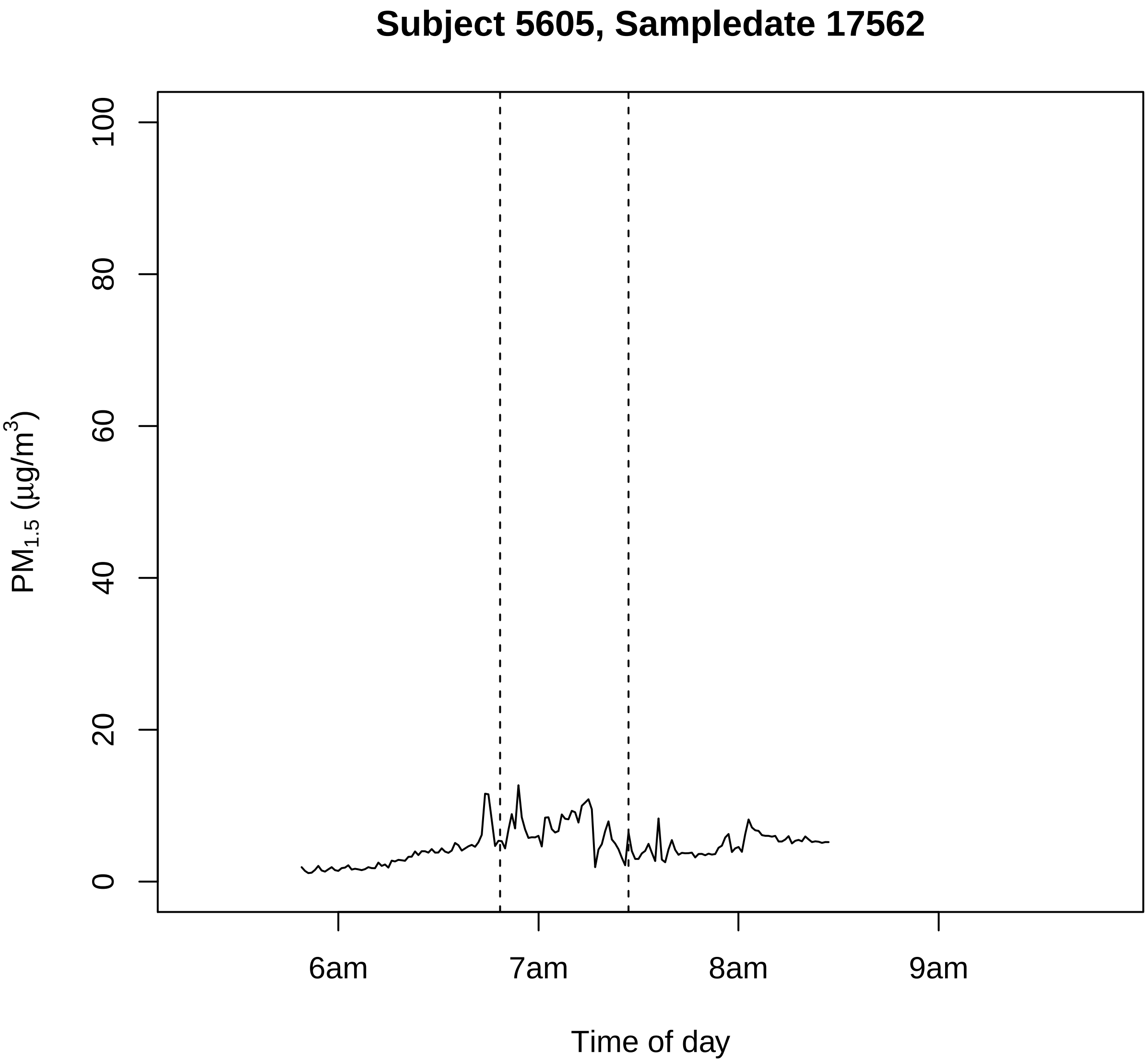
Subject 5605, Sampledate 17562
PM1.5 (µg/m3)
0
20
40
60
80
100
6am
7am
8am
9am
Time of day

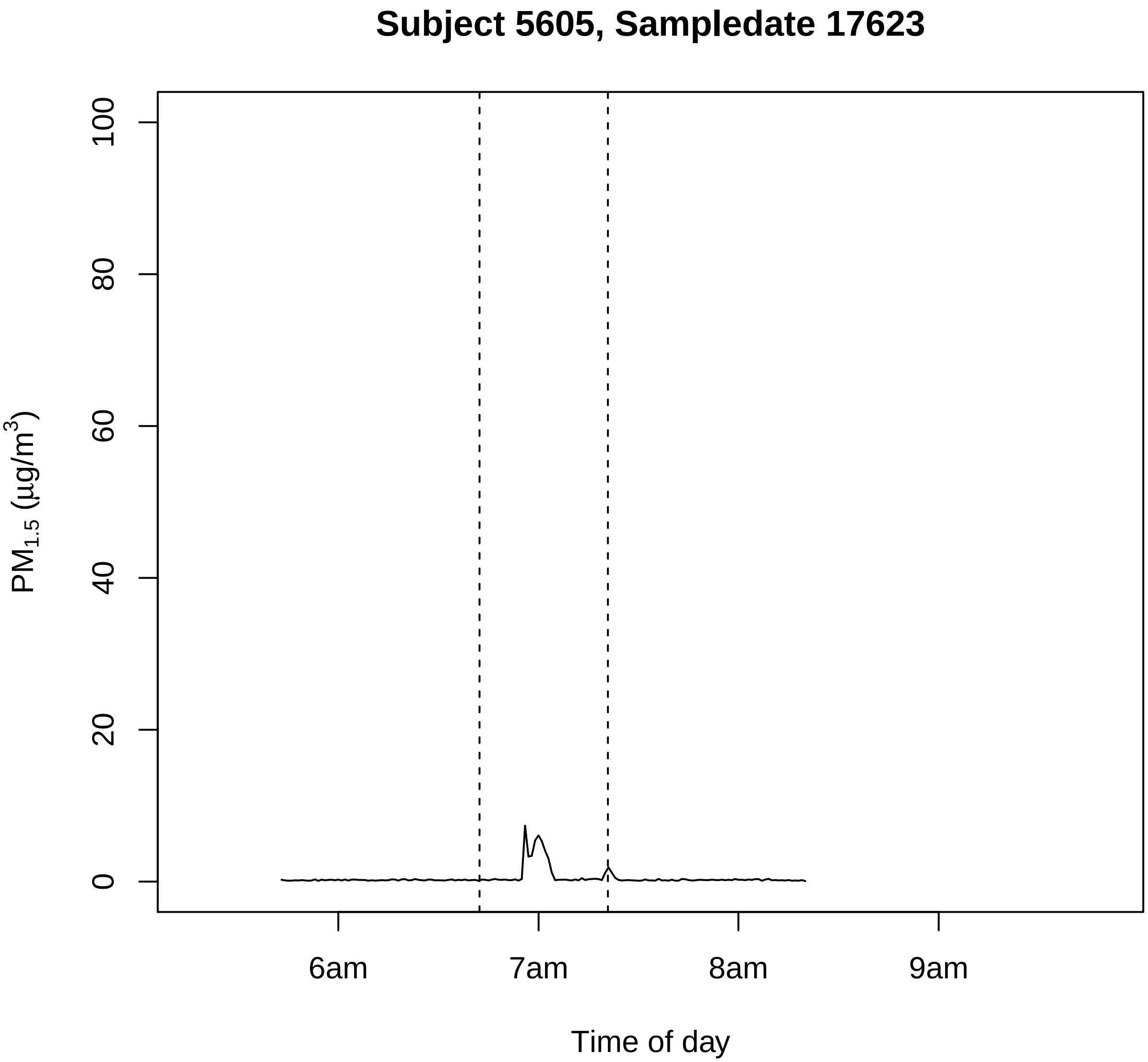
Subject 5605, Sampledate 17623
$PM_{1.5}$ (µg/m^3)
0
20
40
60
80
100
6am
7am
8am
9am
Time of day

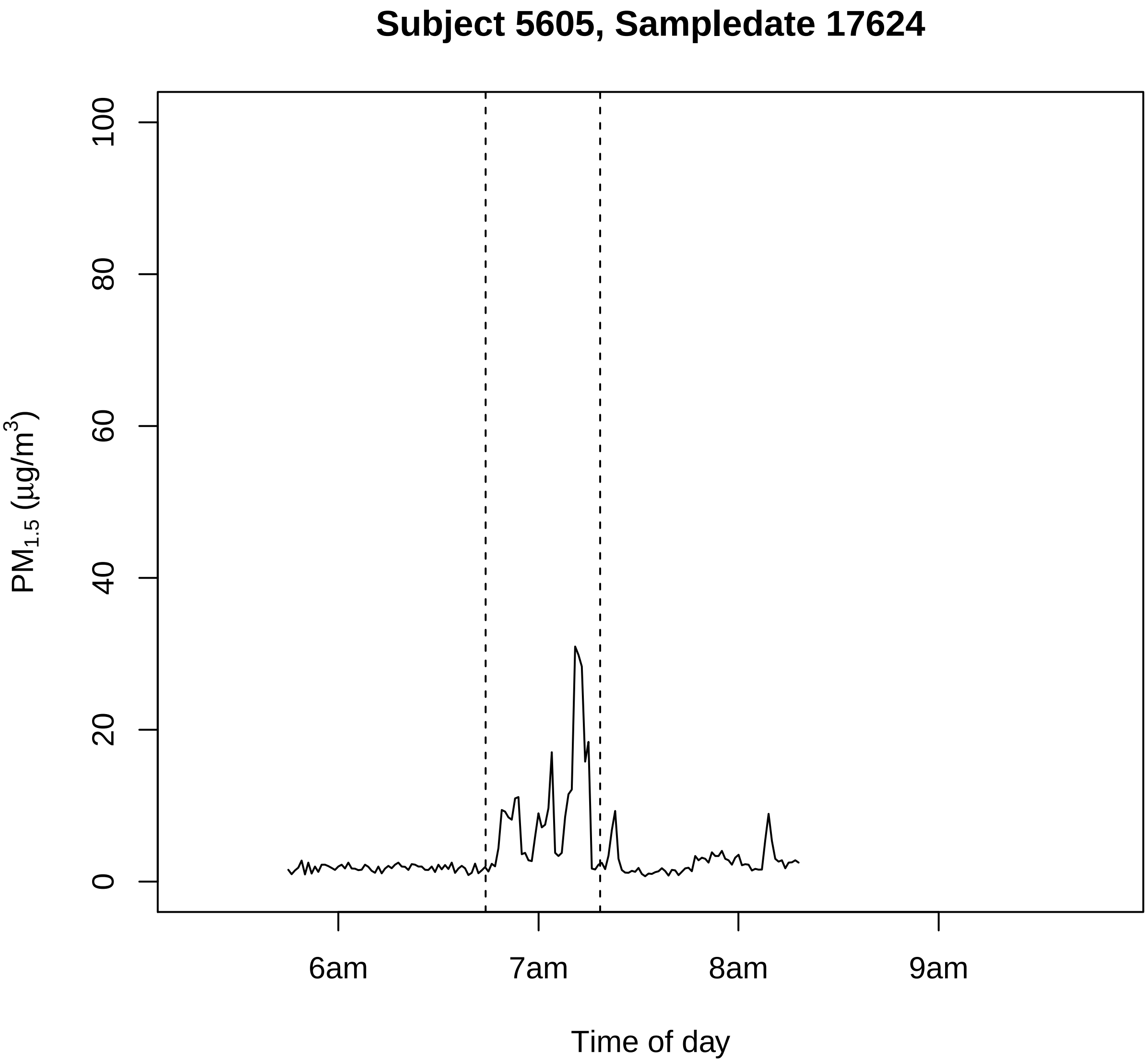

Subject 5605, Sampledate 17624
PM1.5 (µg/m3)
0
20
40
60
80
100
6am
7am
8am
9am
Time of day

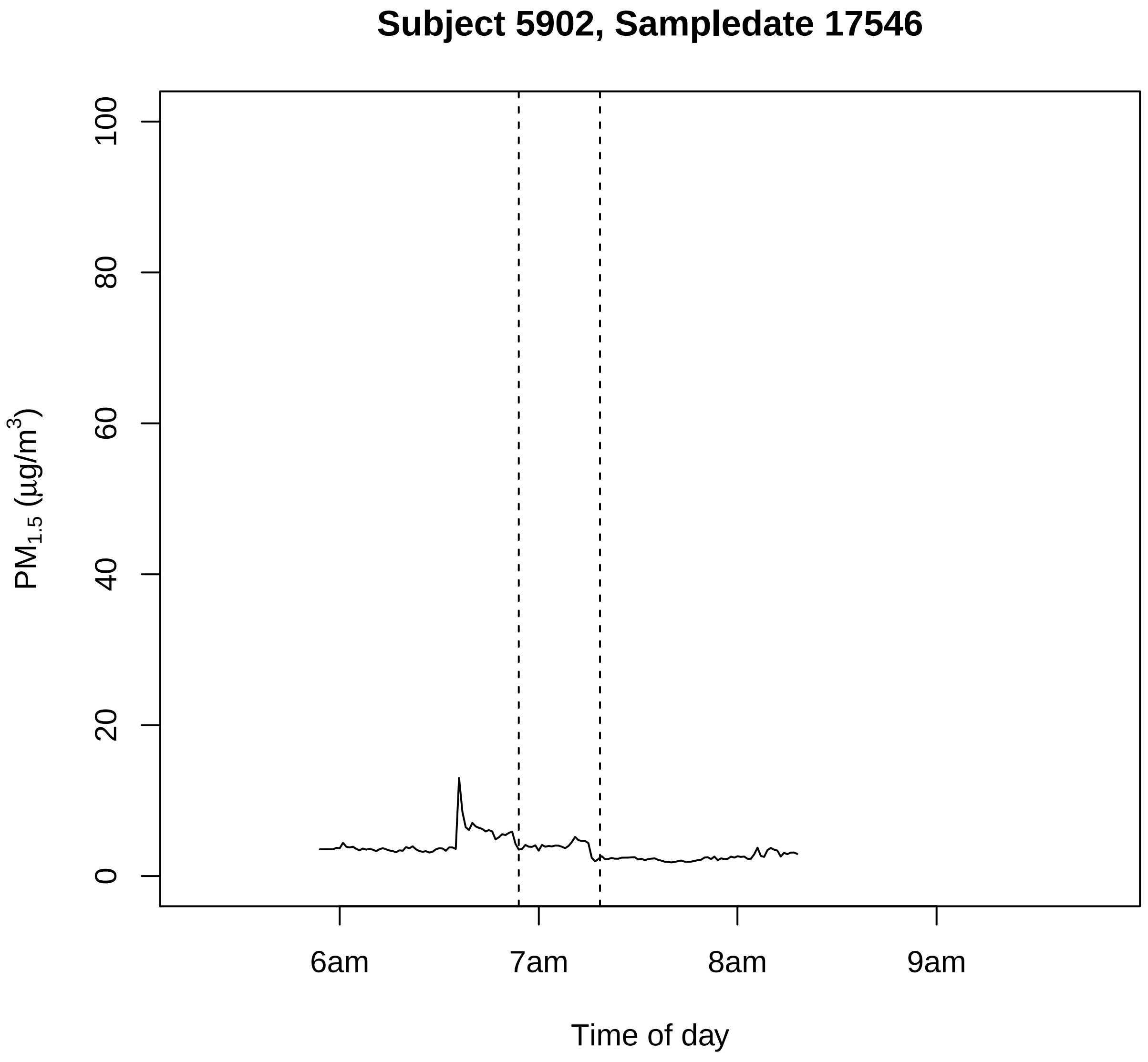

Subject 5902, Sampledate 17546
0
20
40
60
80
100
$PM_{1.5}$ (µg/m^3)
6am
7am
8am
9am
Time of day

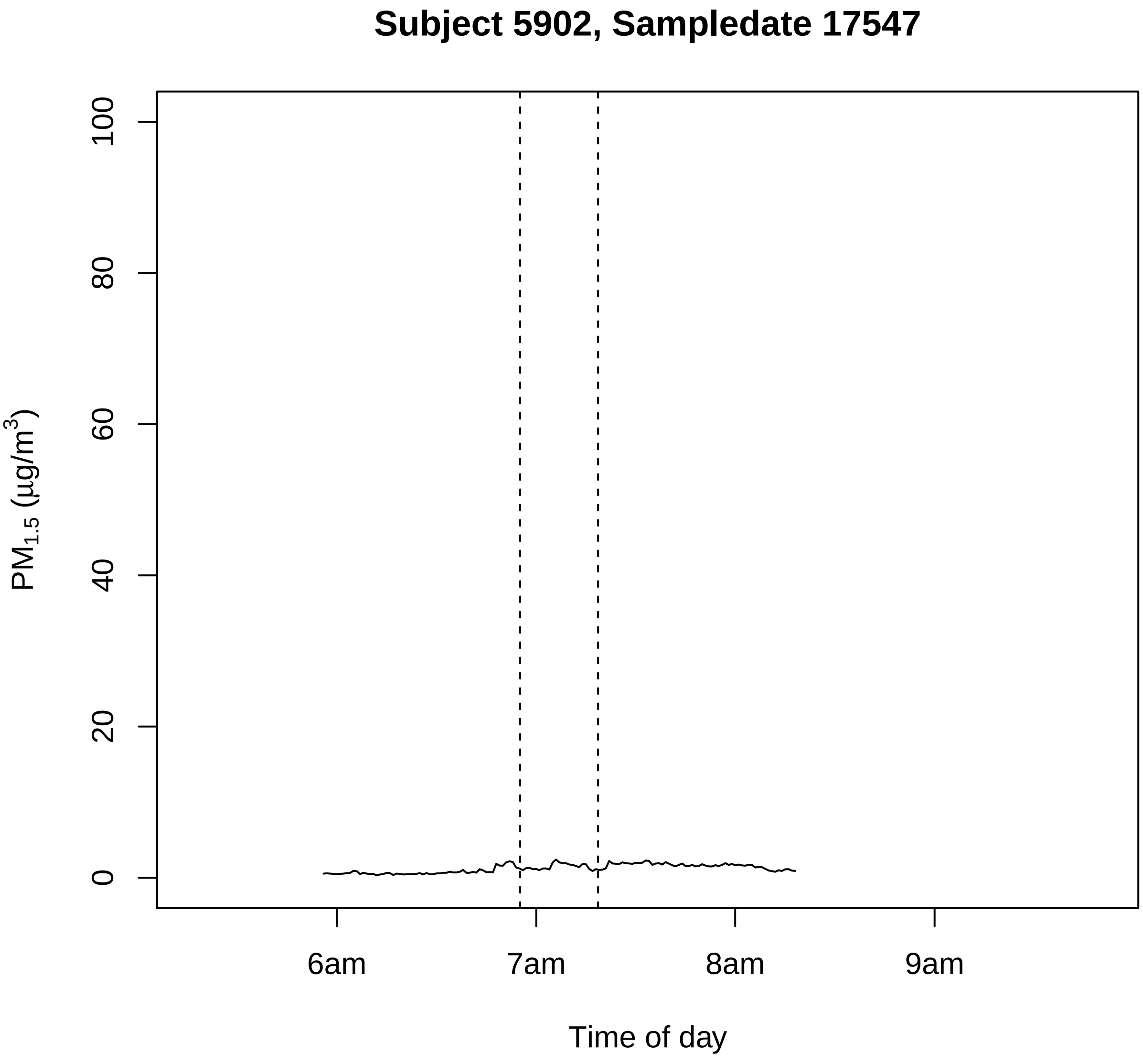

Subject 5902, Sampledate 17547
PM1.5 (µg/m3)
0
20
40
60
80
100
6am
7am
8am
9am
Time of day

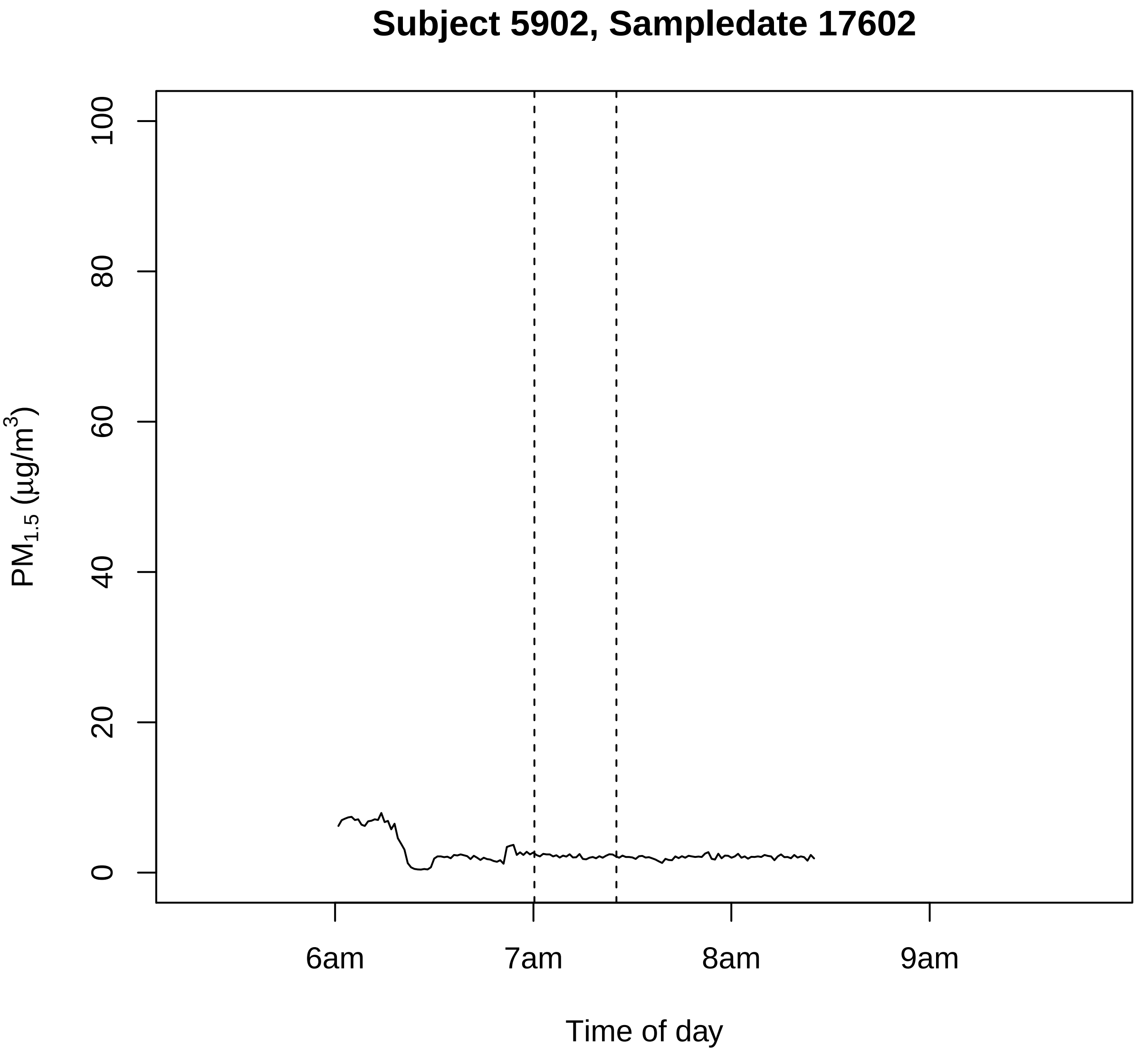
Subject 5902, Sampledate 17602
$PM_{1.5}$ (µg/m^3)
0
20
40
60
80
100
6am
7am
8am
9am
Time of day

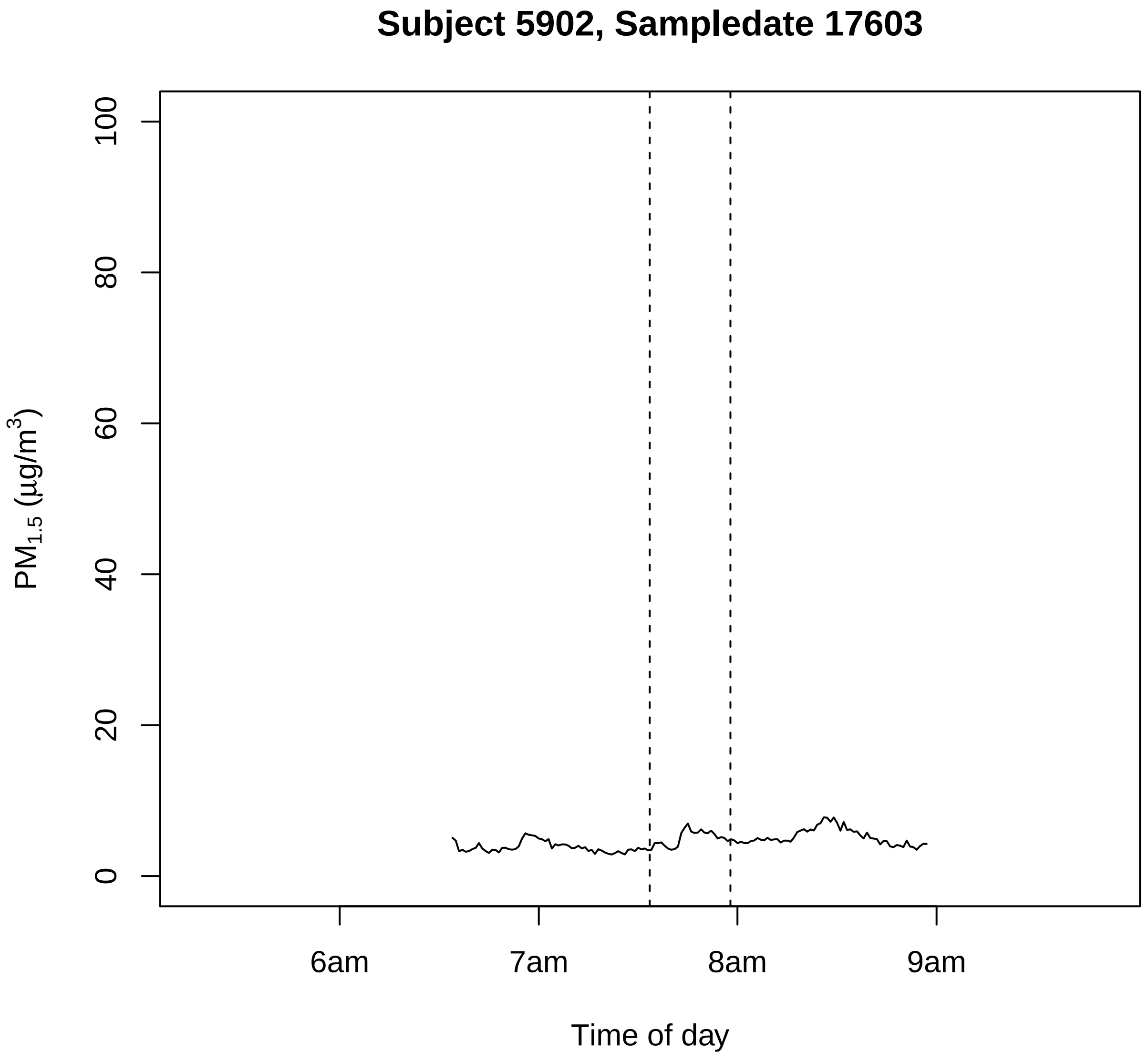
Subject 5902, Sampledate 17603
PM1.5 (µg/m3)
0
20
40
60
80
100
6am
7am
8am
9am
Time of day

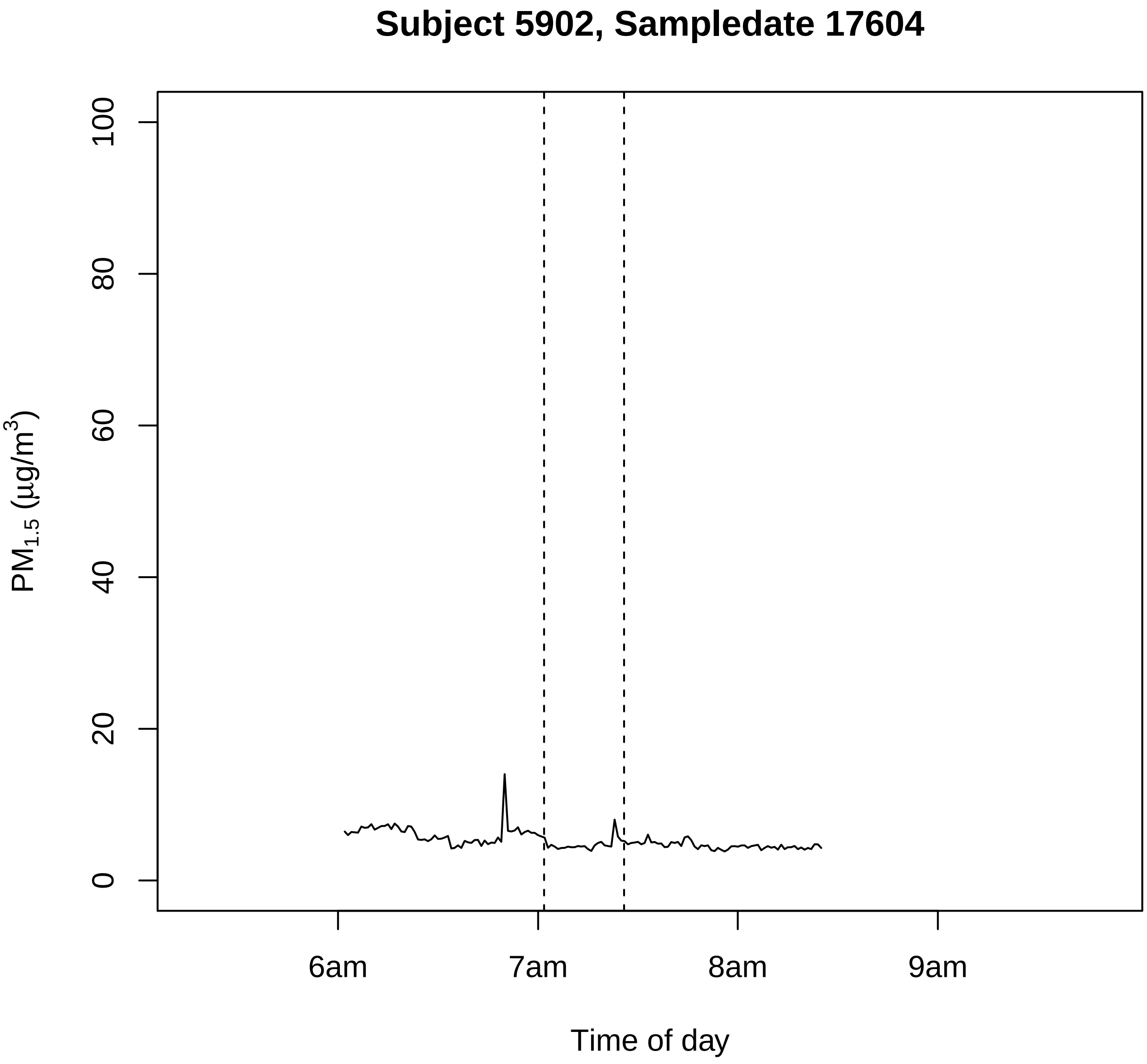
Subject 5902, Sampledate 17604
PM1.5 (µg/m3)
0
20
40
60
80
100
6am
7am
8am
9am
Time of day

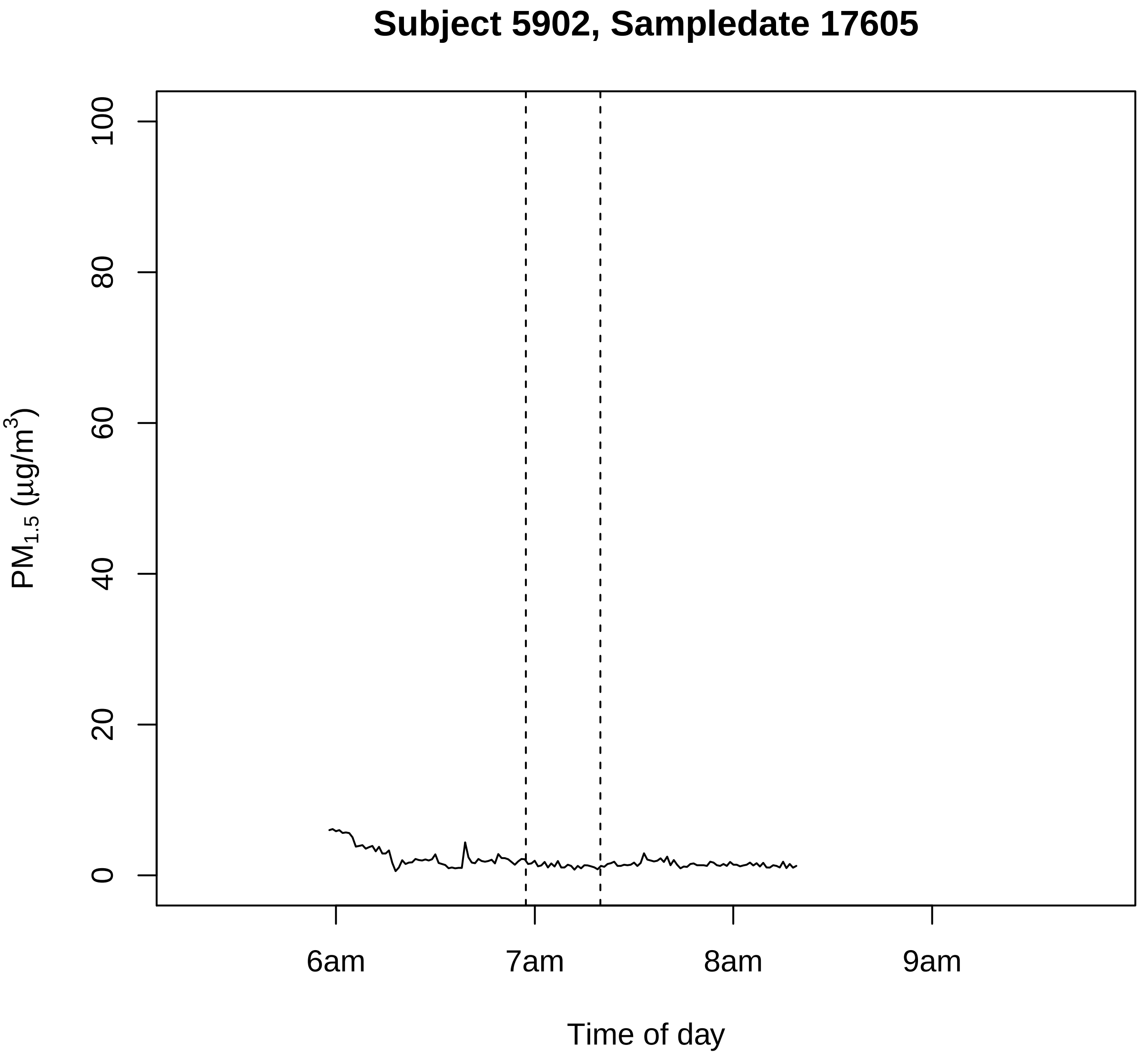

Subject 5902, Sampledate 17605
PM1.5 (µg/m3)
0
20
40
60
80
100
6am
7am
8am
9am
Time of day

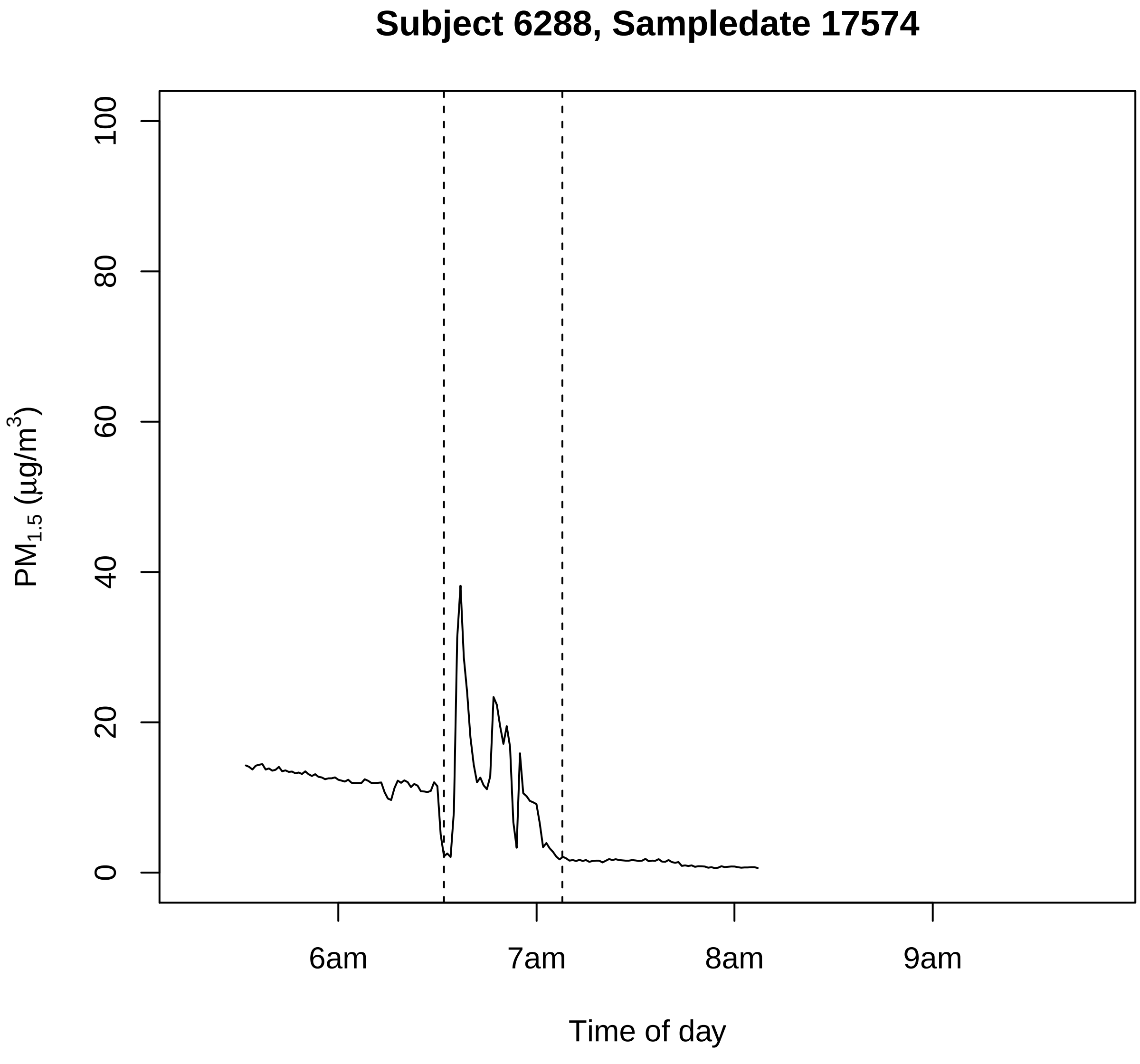

Subject 6288, Sampledate 17574
0
20
40
60
80
100
PM1.5 (µg/m3)
6am
7am
8am
9am
Time of day

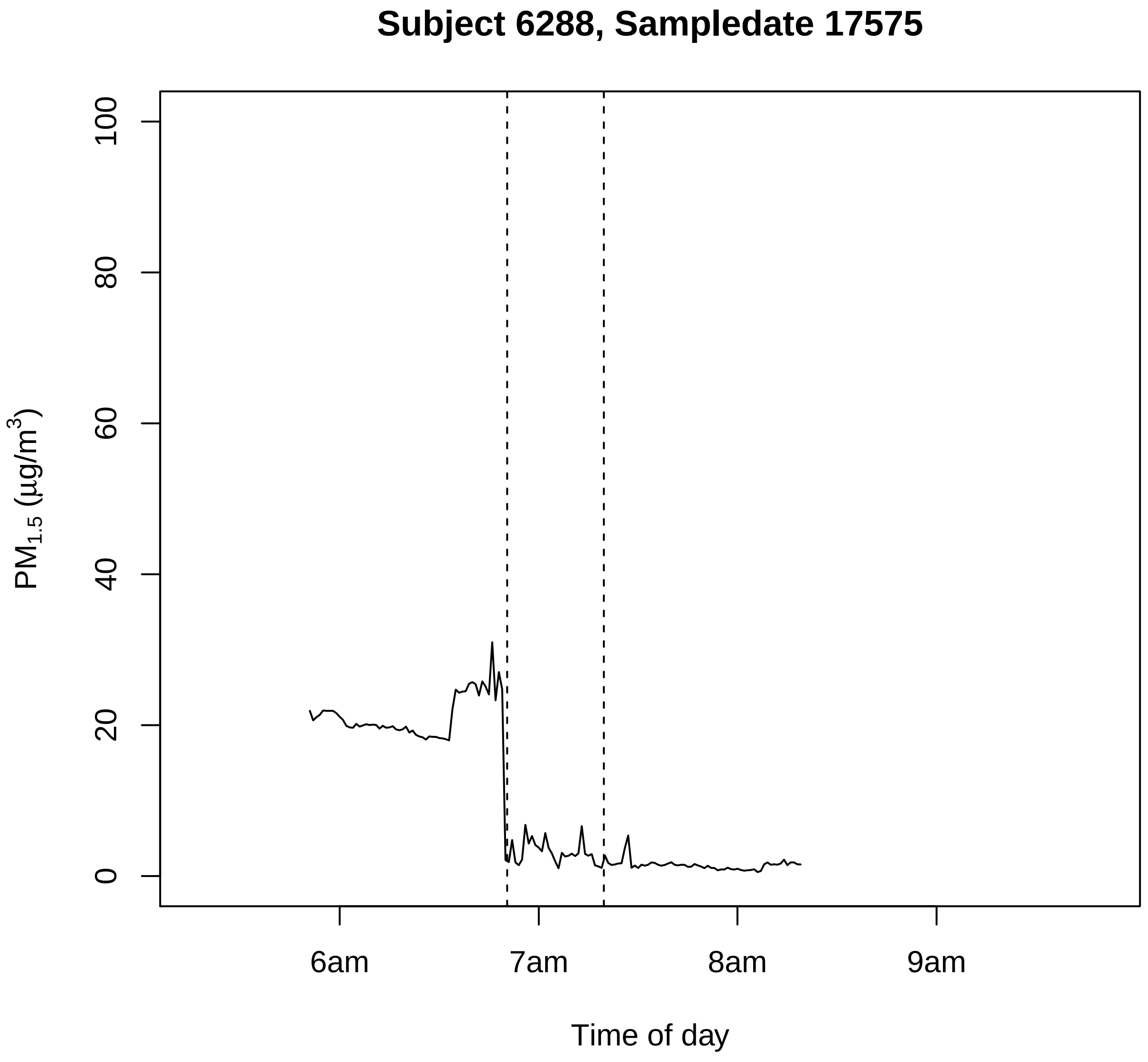
Subject 6288, Sampledate 17575
PM1.5 (µg/m3)
0
20
40
60
80
100
6am
7am
8am
9am
Time of day

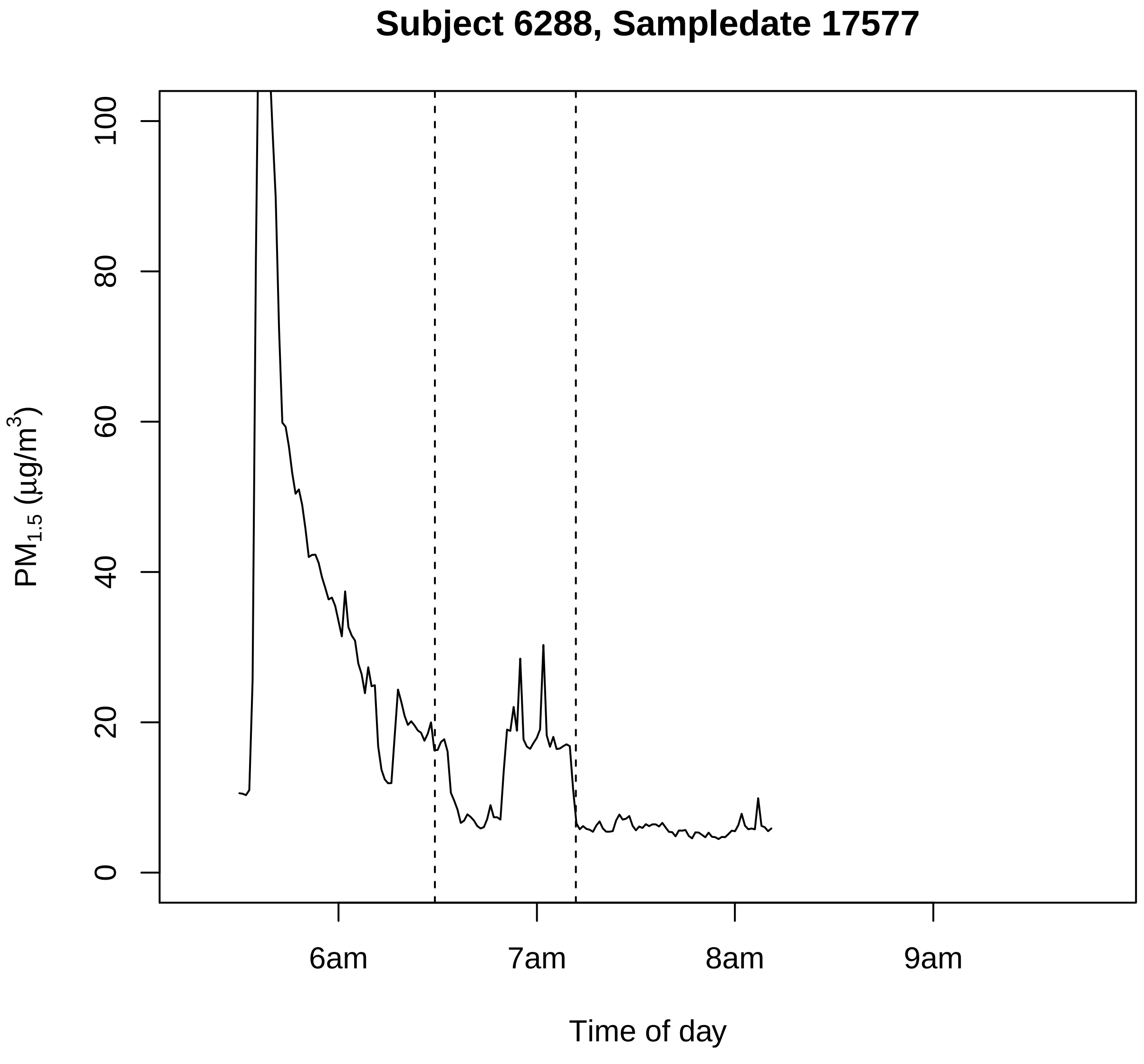

Subject 6288, Sampledate 17577
PM1.5 (µg/m3)
0
20
40
60
80
100
6am
7am
8am
9am
Time of day

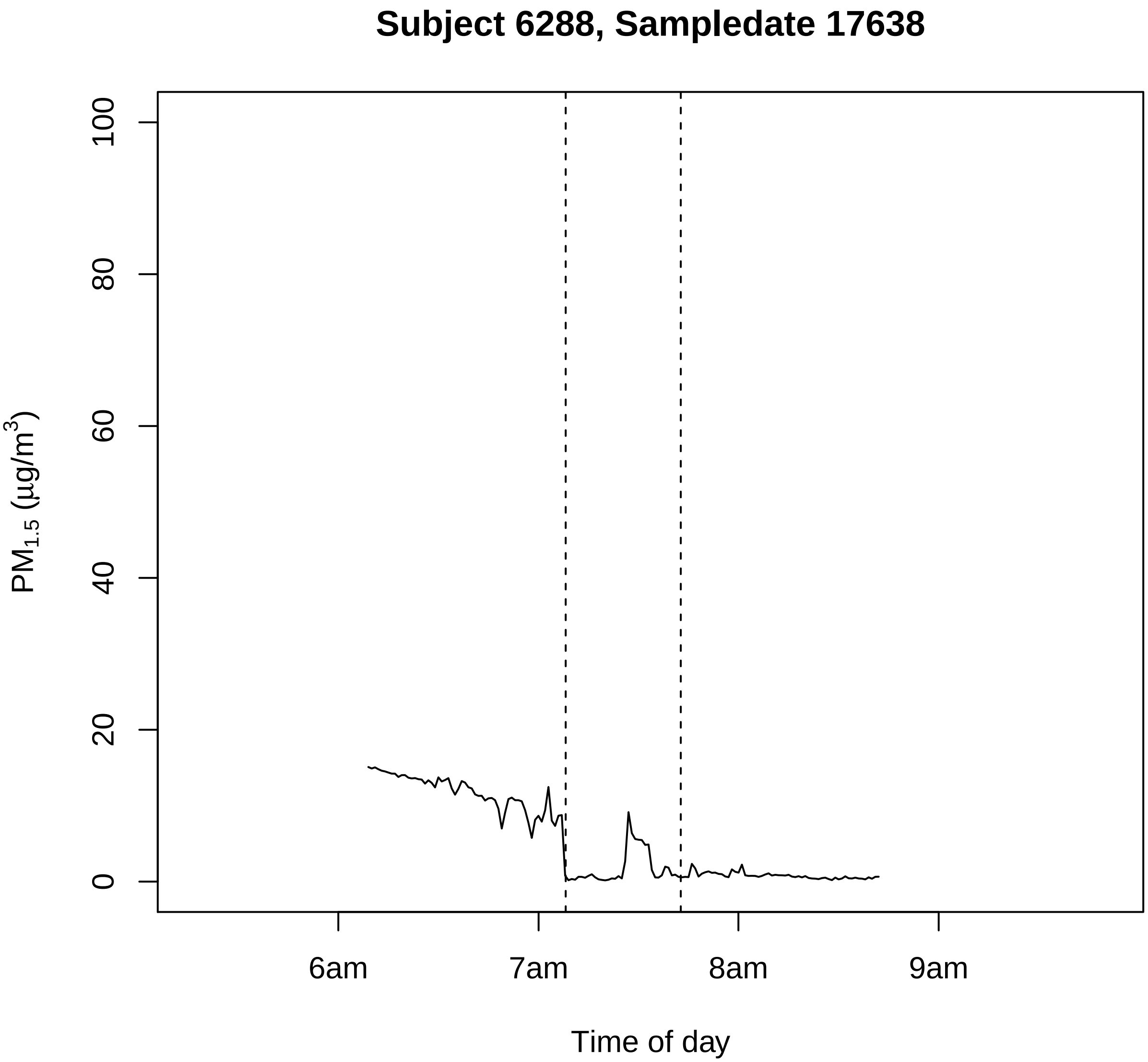

Subject 6288, Sampledate 17638
PM1.5 (µg/m3)
0
20
40
60
80
100
6am
7am
8am
9am
Time of day

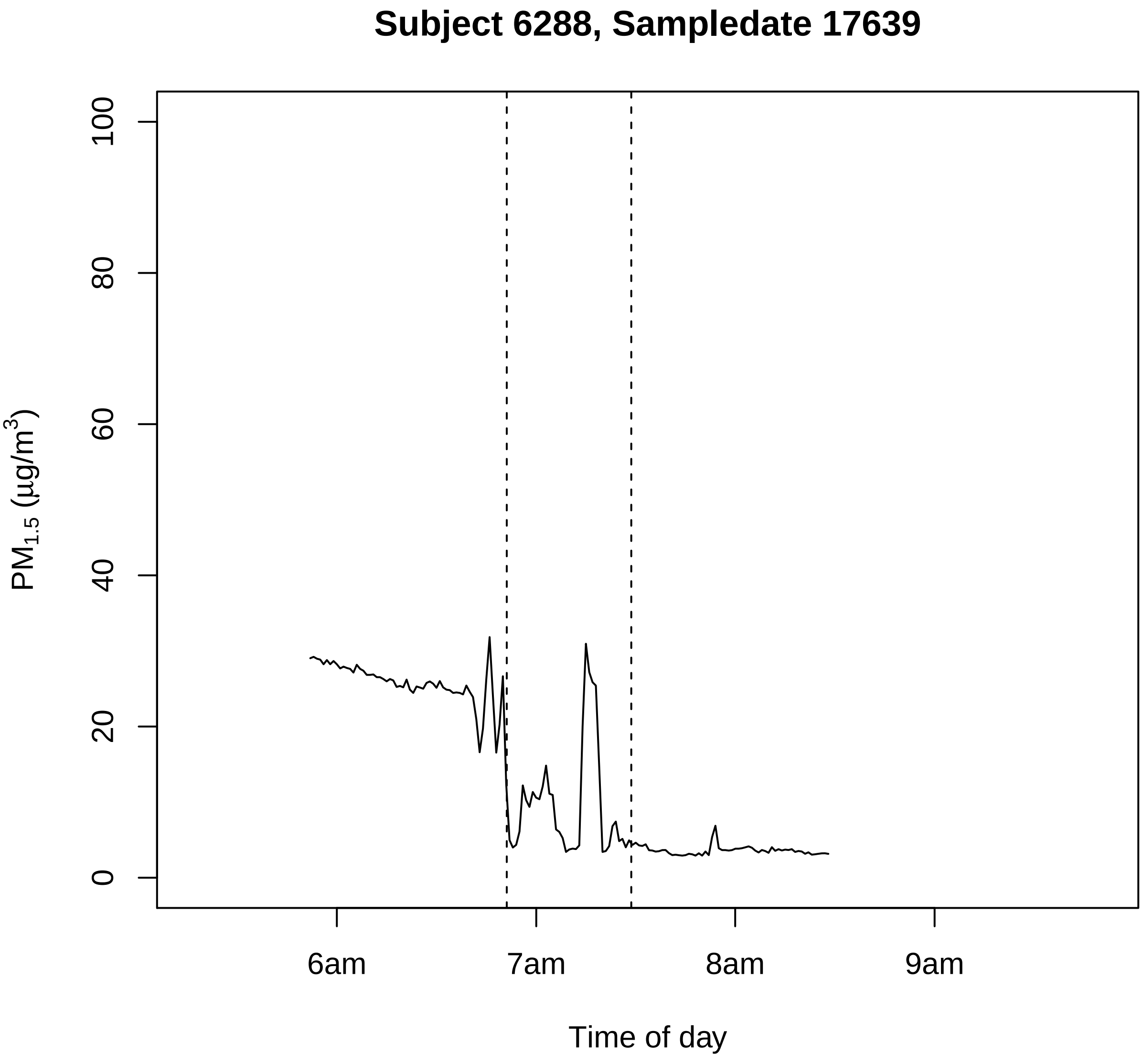

Subject 6288, Sampledate 17639
PM1.5 (µg/m3)
0
20
40
60
80
100
6am
7am
8am
9am
Time of day

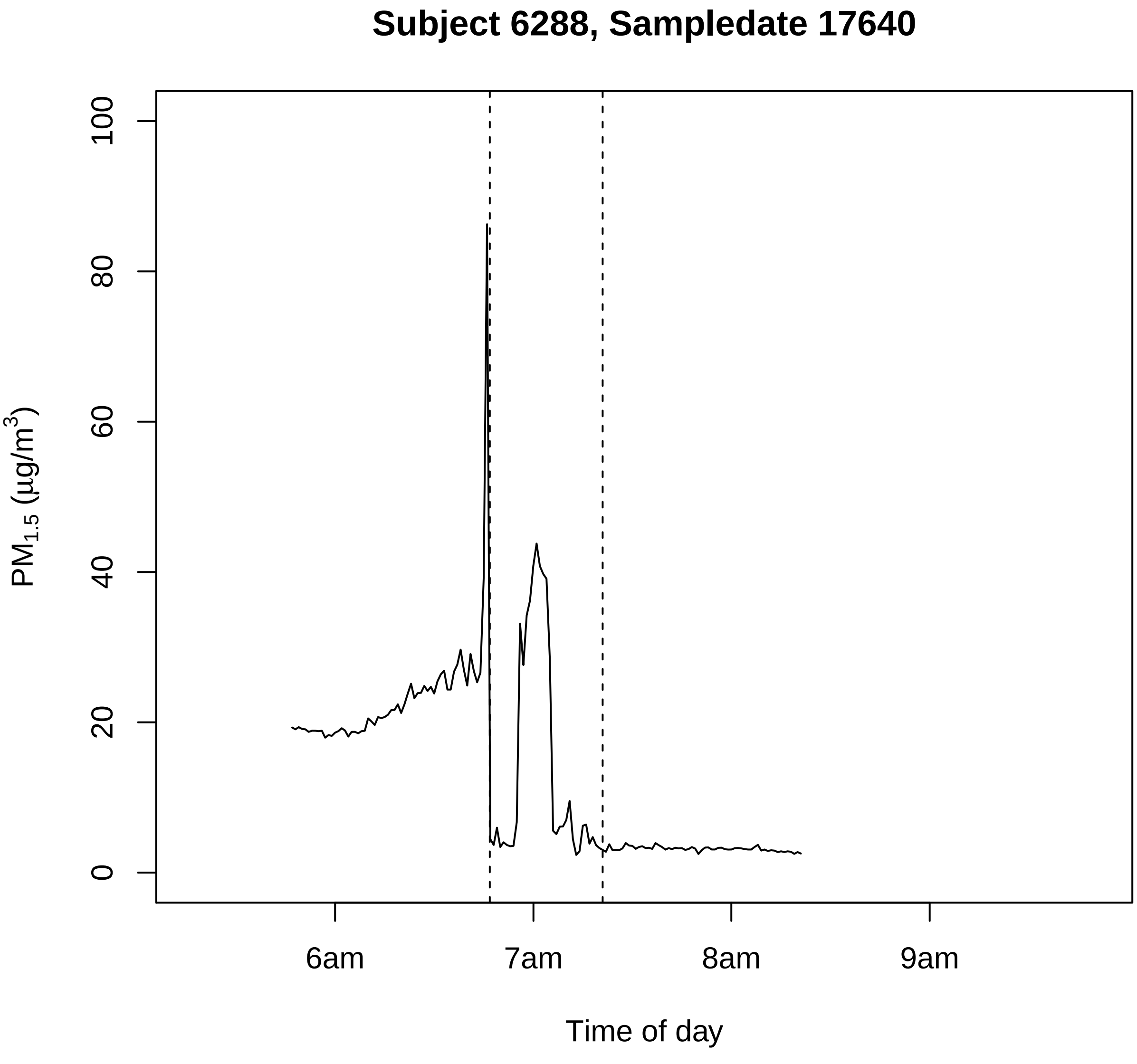

Subject 6288, Sampledate 17640
0
20
40
60
80
100
$PM_{1.5}$ (µg/m^3)
6am
7am
8am
9am
Time of day

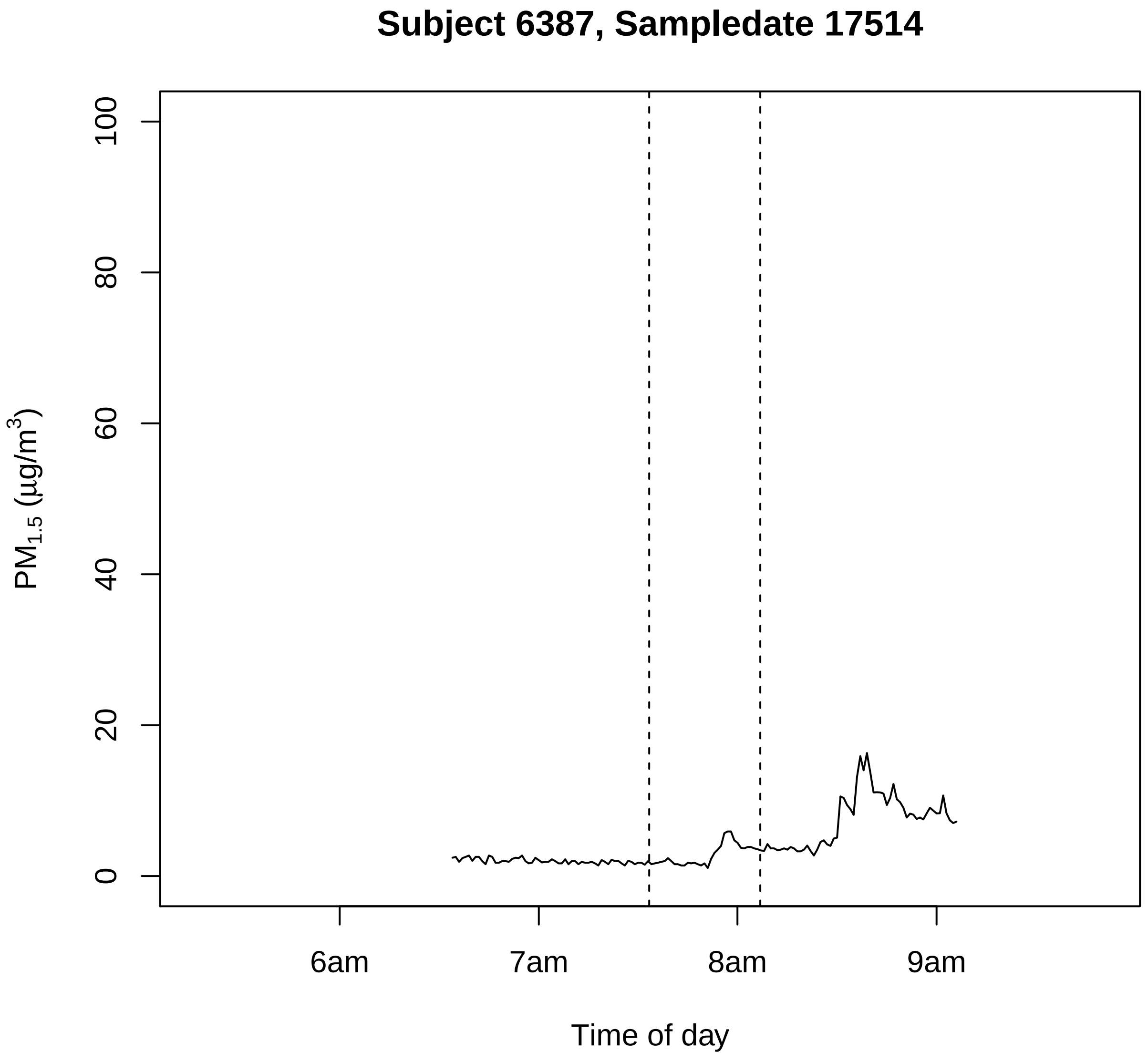
Subject 6387, Sampledate 17514
$PM_{1.5}$ (µg/m^3)
0
20
40
60
80
100
6am
7am
8am
9am
Time of day

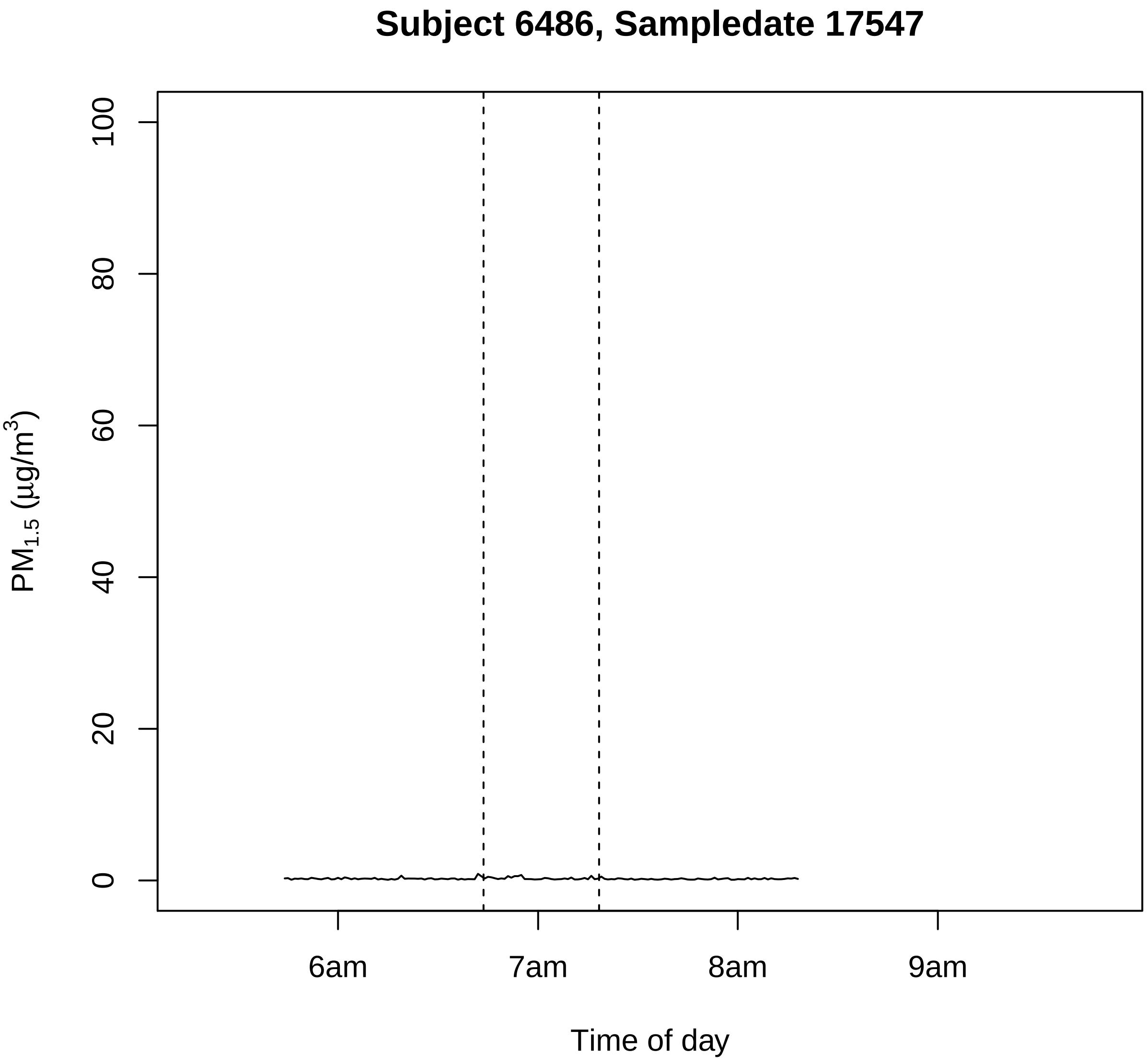
Subject 6486, Sampledate 17547
PM1.5 (µg/m3)
0
20
40
60
80
100
6am
7am
8am
9am
Time of day

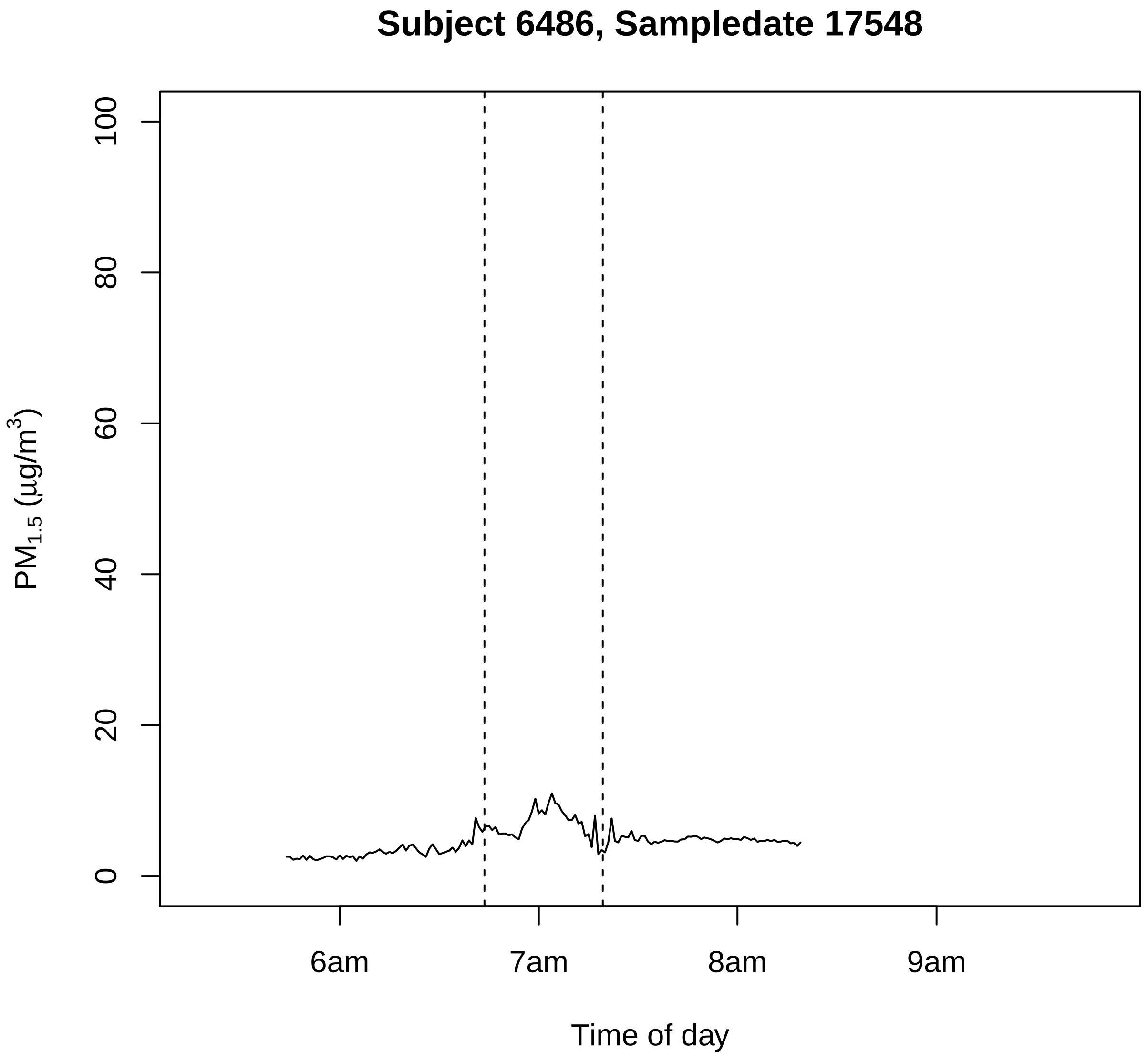

Subject 6486, Sampledate 17548
$PM_{1.5}$ (µg/m^3)
0
20
40
60
80
100
6am
7am
8am
9am
Time of day

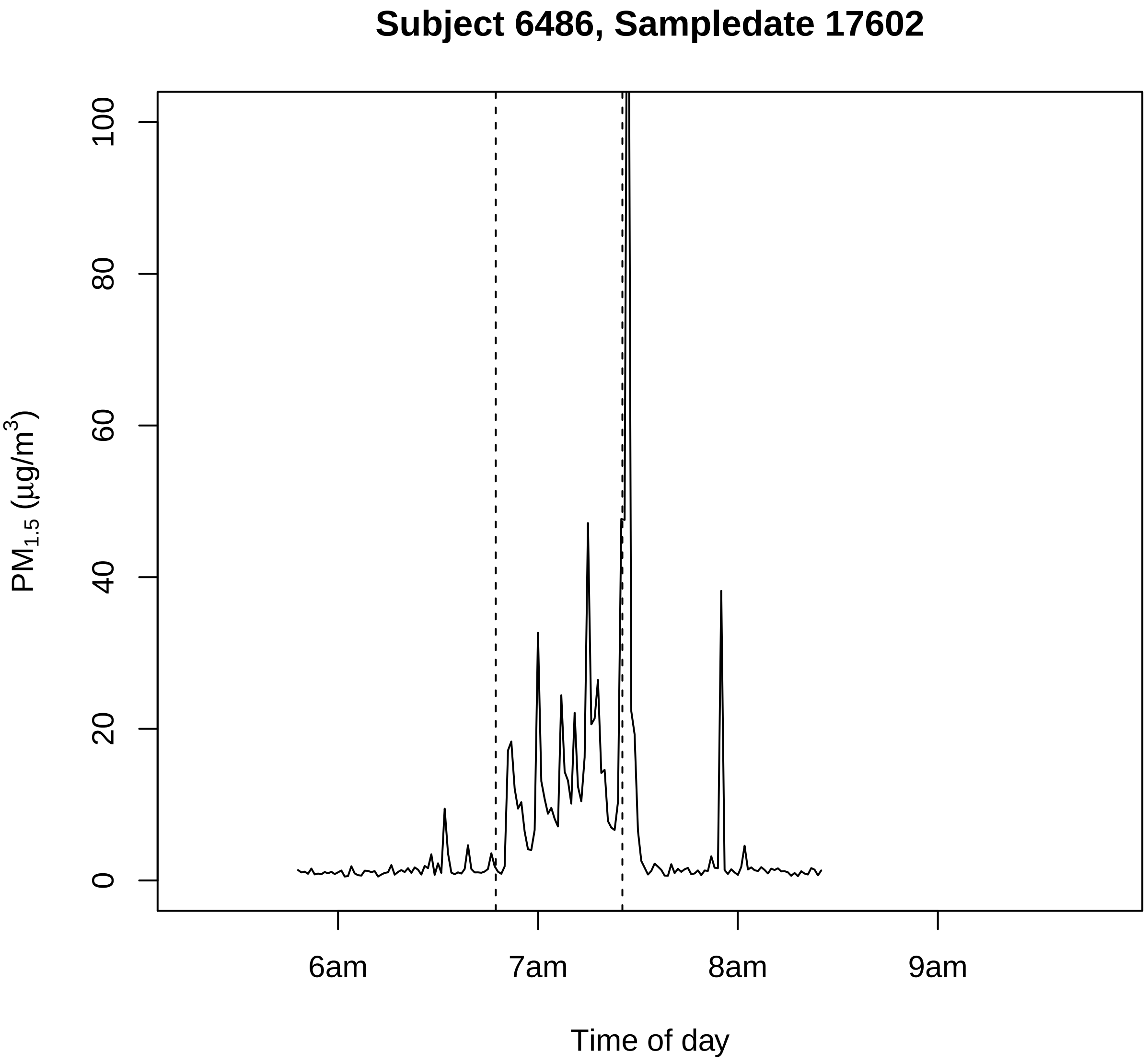
Subject 6486, Sampledate 17602
PM1.5 (µg/m3)
0
20
40
60
80
100
6am
7am
8am
9am
Time of day

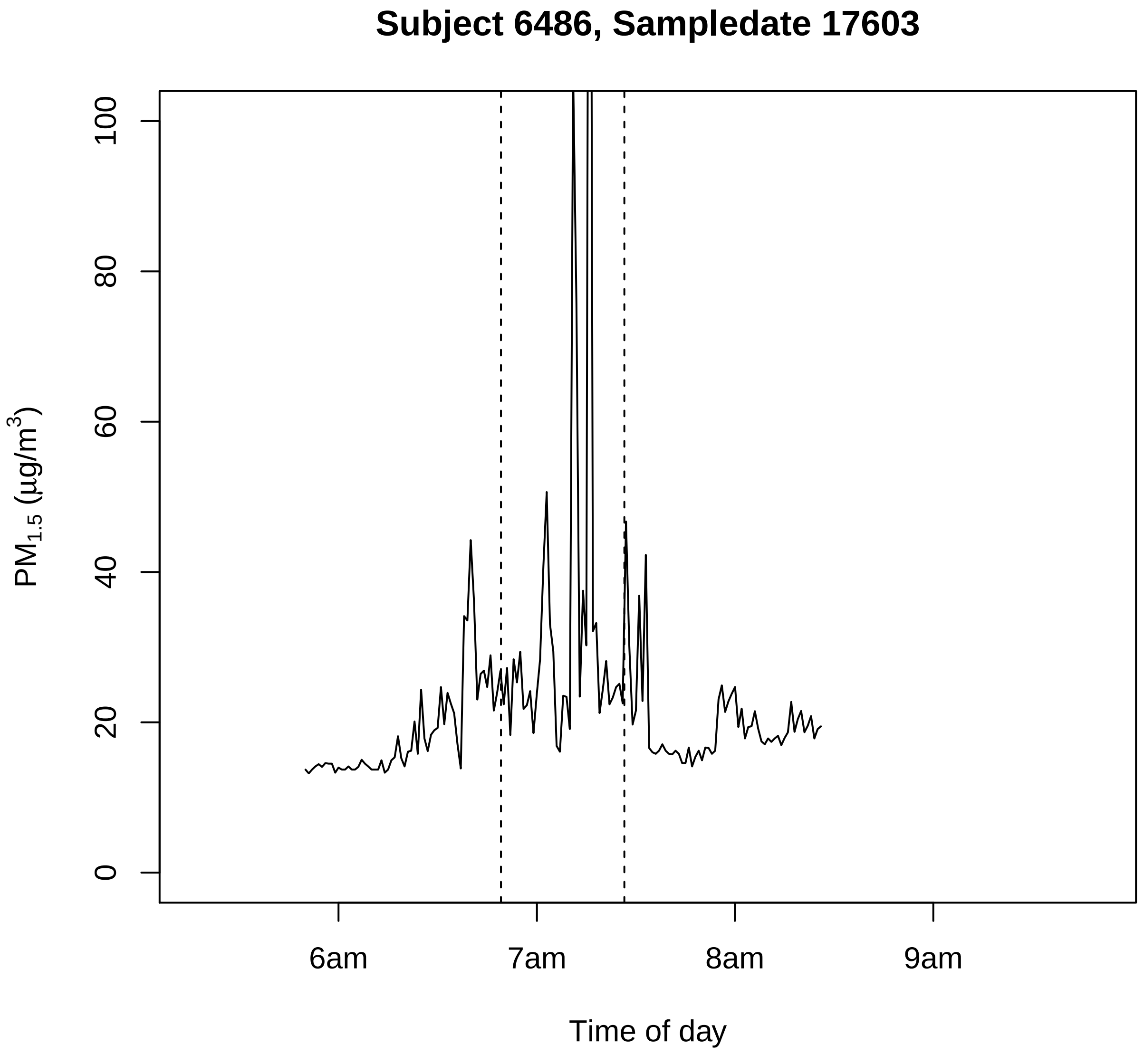

Subject 6486, Sampledate 17603
PM1.5 (µg/m3)
0
20
40
60
80
100
6am
7am
8am
9am
Time of day

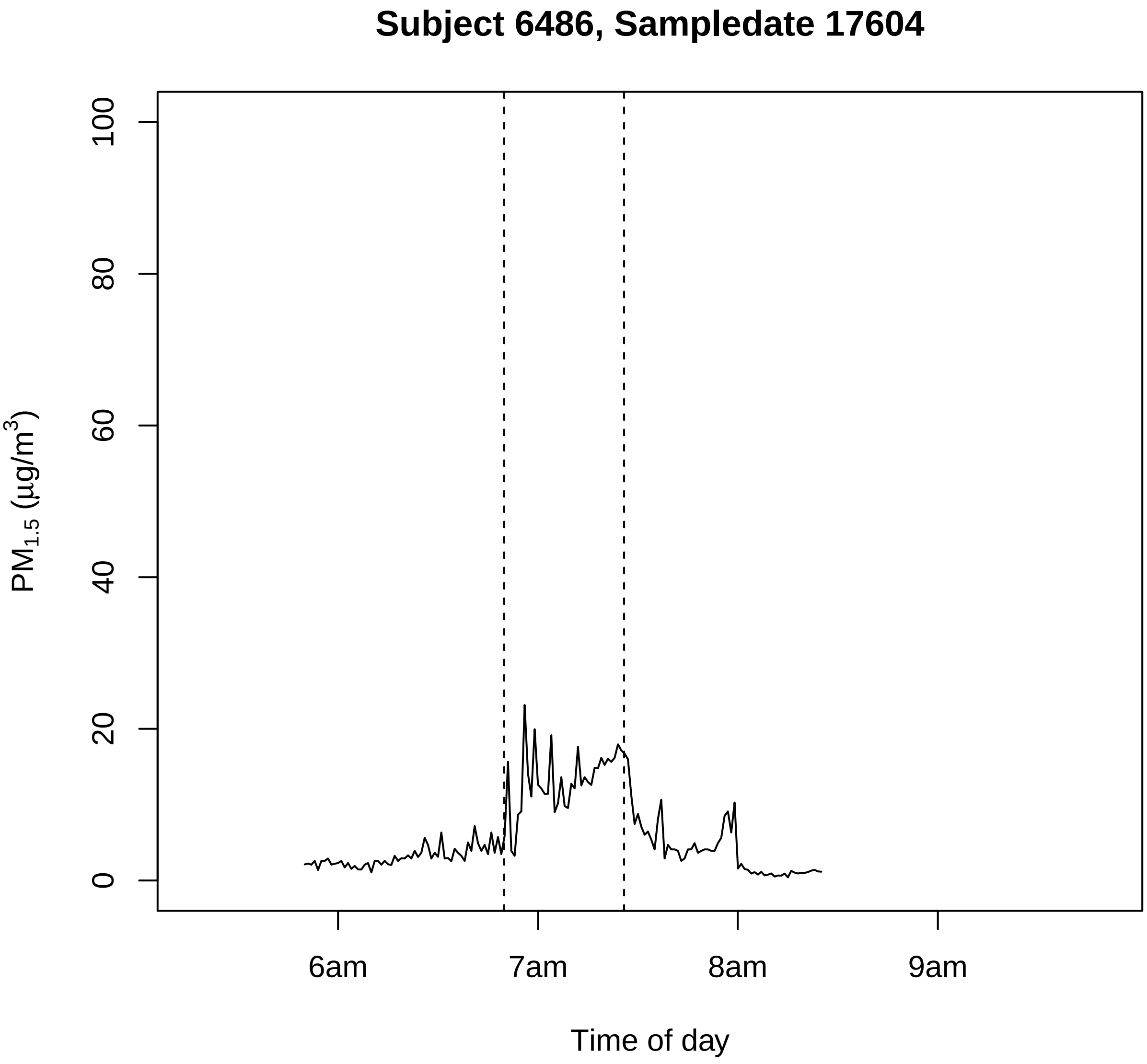
Subject 6486, Sampledate 17604
PM1.5 (µg/m3)
0
20
40
60
80
100
6am
7am
8am
9am
Time of day

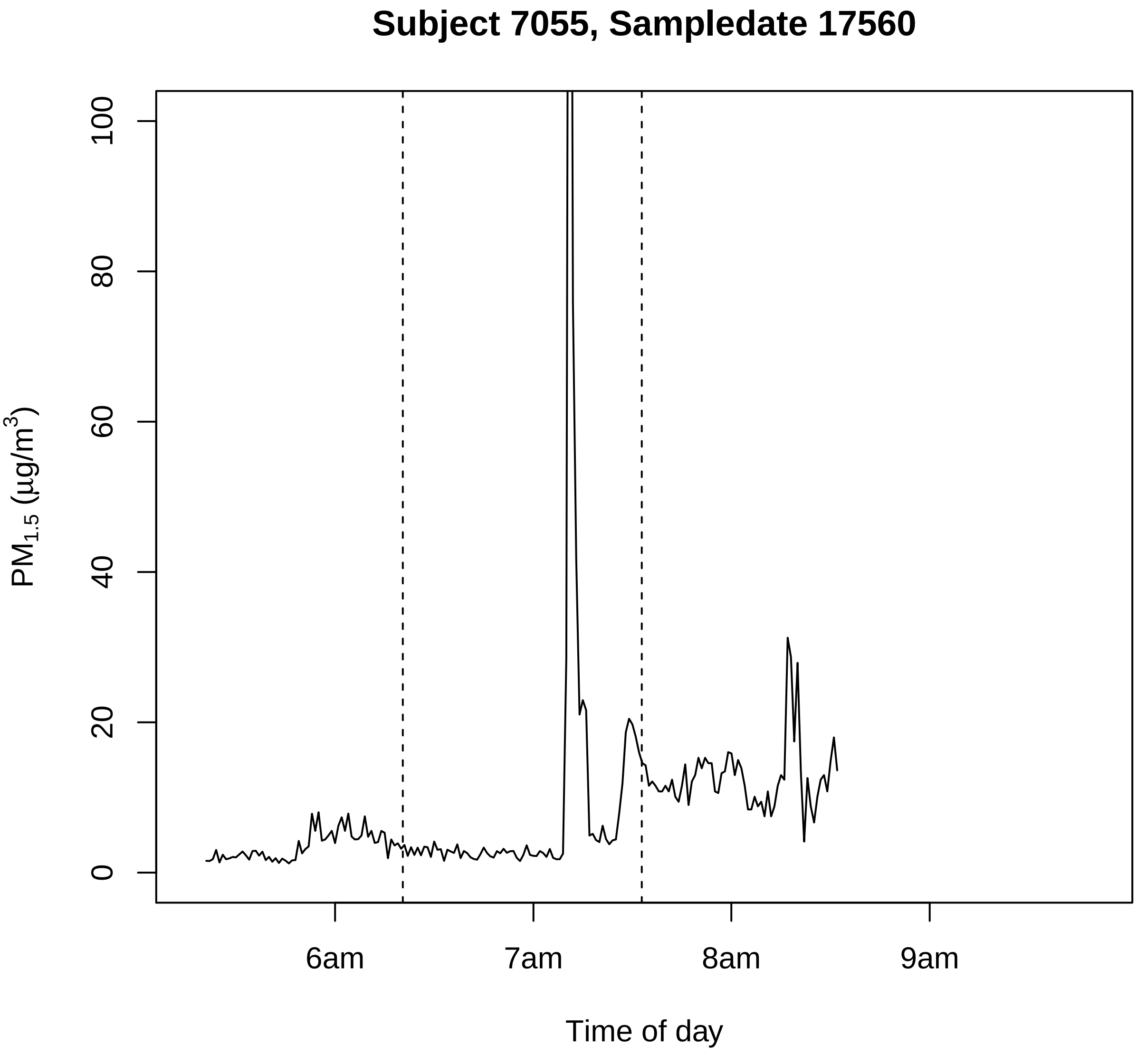

Subject 7055, Sampledate 17560
PM1.5 (µg/m3)
0
20
40
60
80
100
6am
7am
8am
9am
Time of day

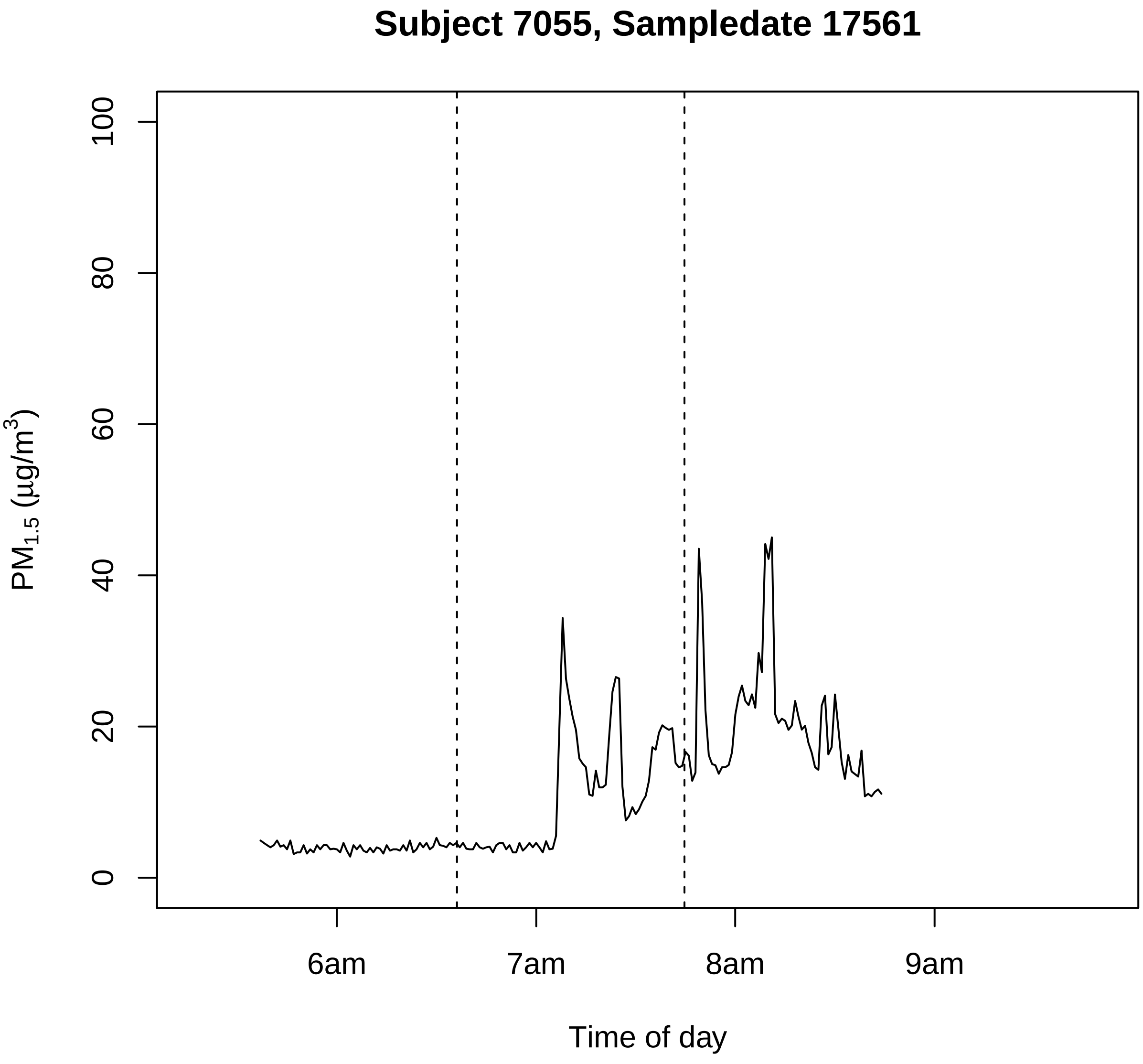

Subject 7055, Sampledate 17561
PM1.5 (µg/m3)
0
20
40
60
80
100
6am
7am
8am
9am
Time of day

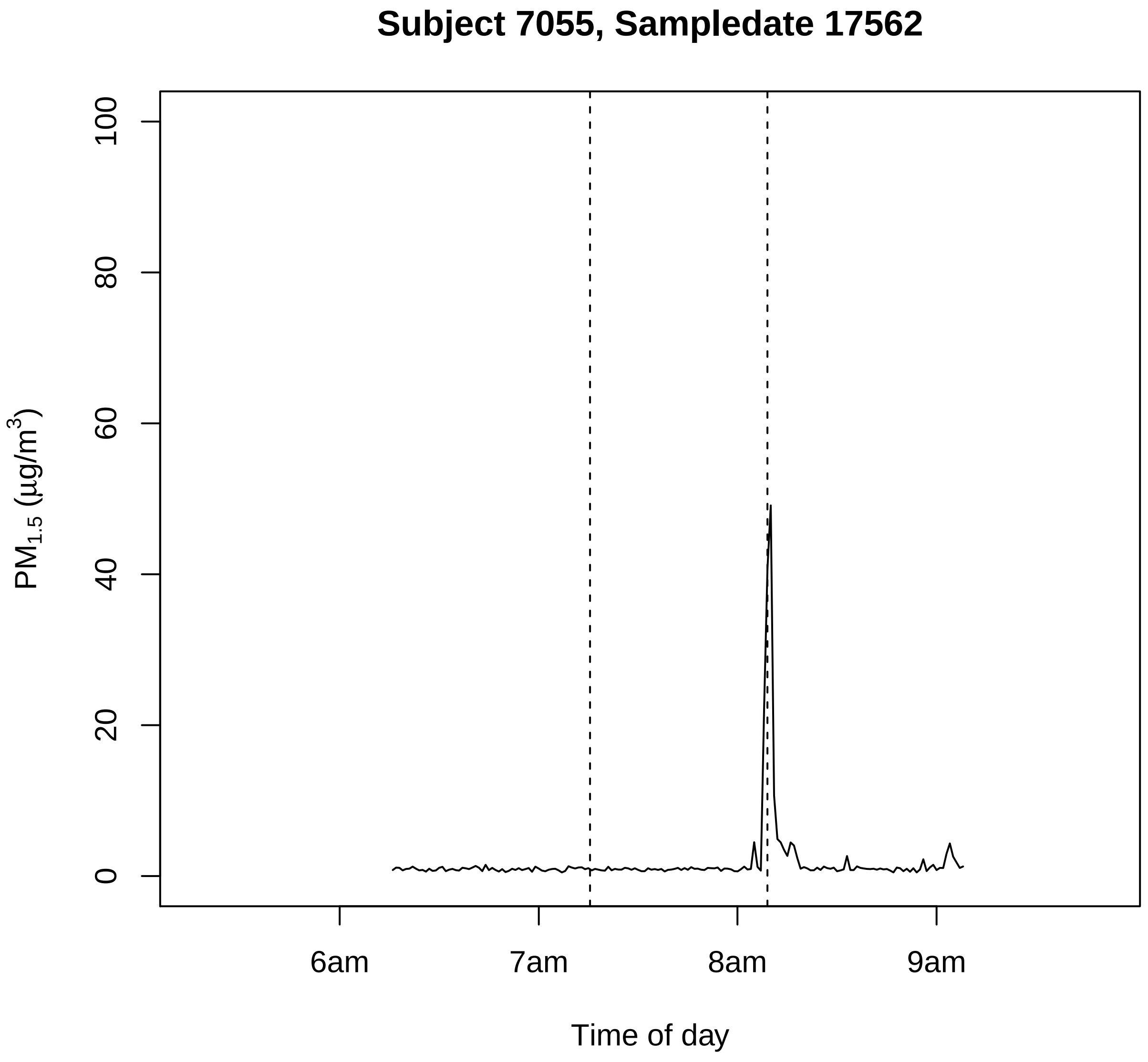

Subject 7055, Sampledate 17562
100
80
60
40
20
0
PM1.5 (µg/m3)
6am
7am
8am
9am
Time of day

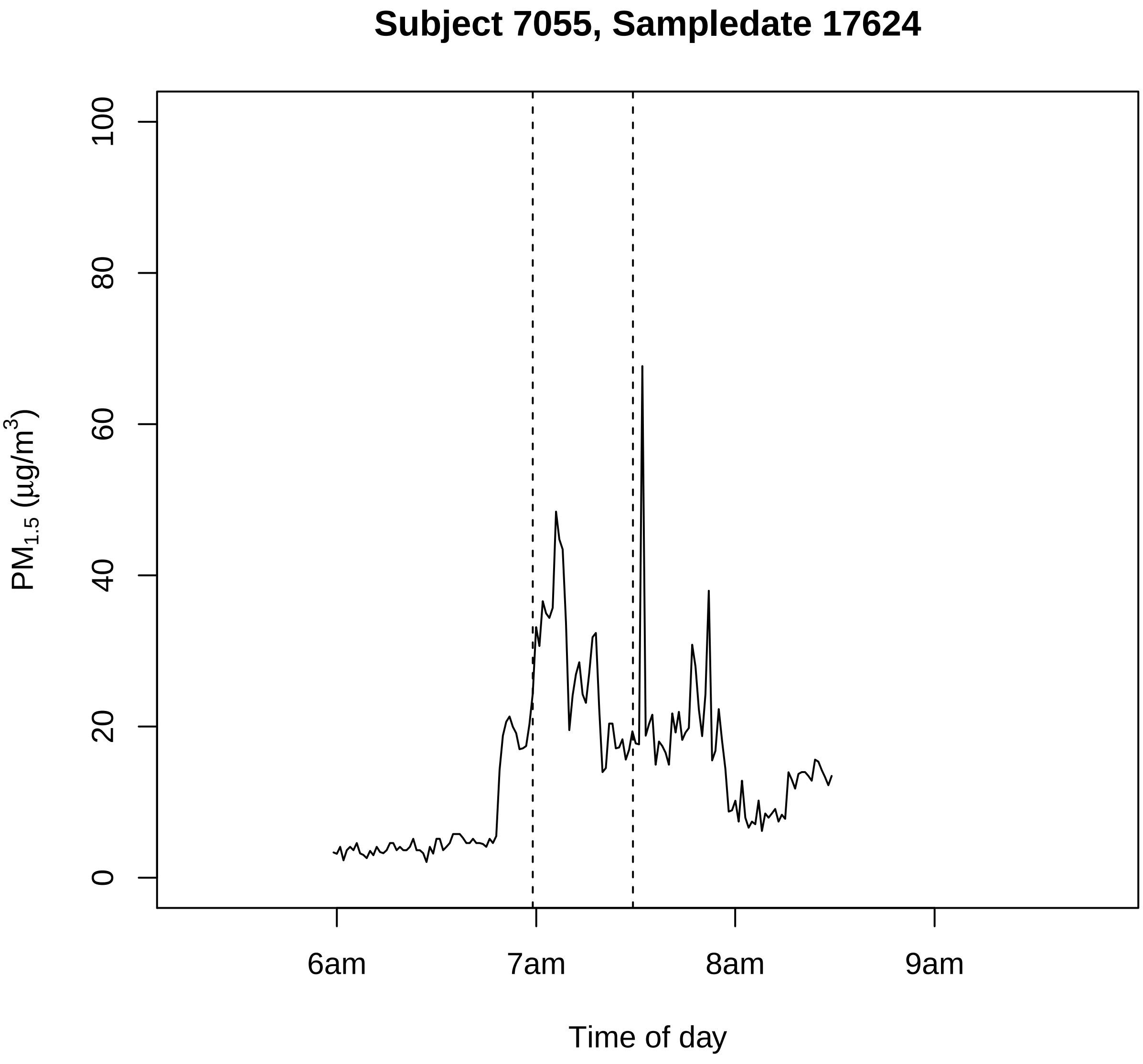
Subject 7055, Sampledate 17624
PM1.5 (µg/m3)
0
20
40
60
80
100
6am
7am
8am
9am
Time of day

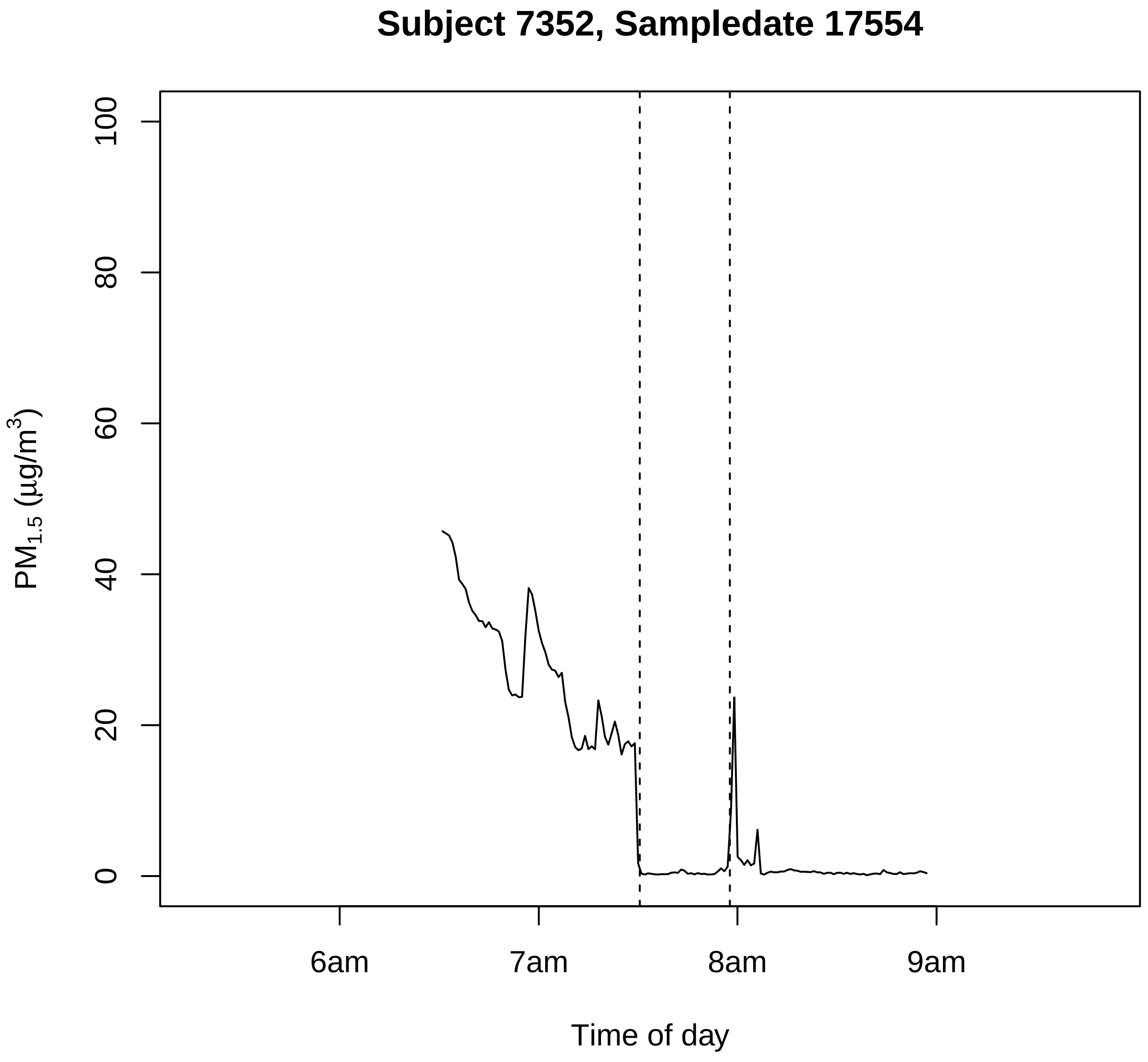

Subject 7352, Sampledate 17554
PM1.5 (µg/m3)
100
80
60
40
20
0
6am
7am
8am
9am
Time of day

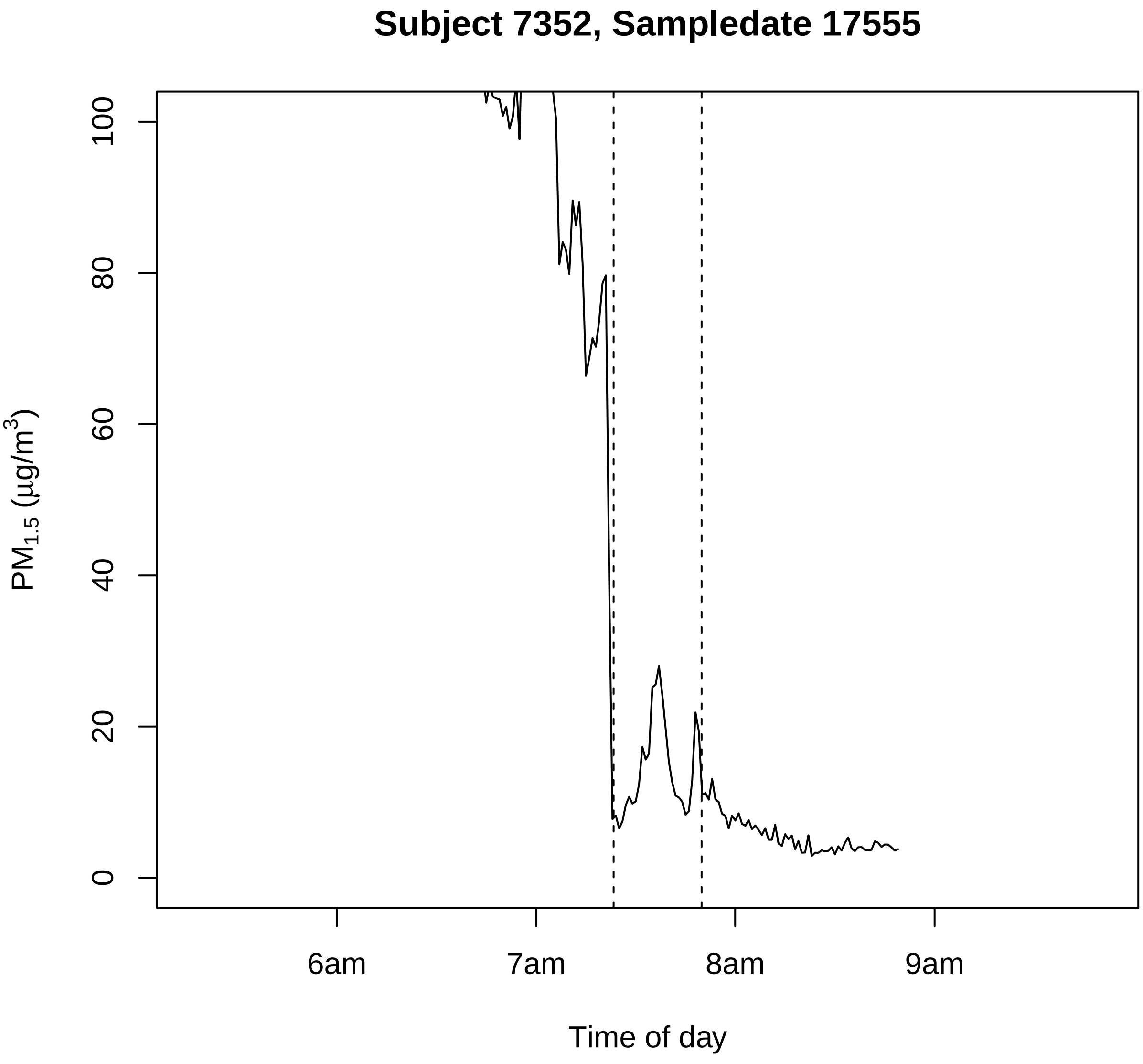

Subject 7352, Sampledate 17555
0
20
40
60
80
100
PM1.5 (µg/m3)
6am
7am
8am
9am
Time of day

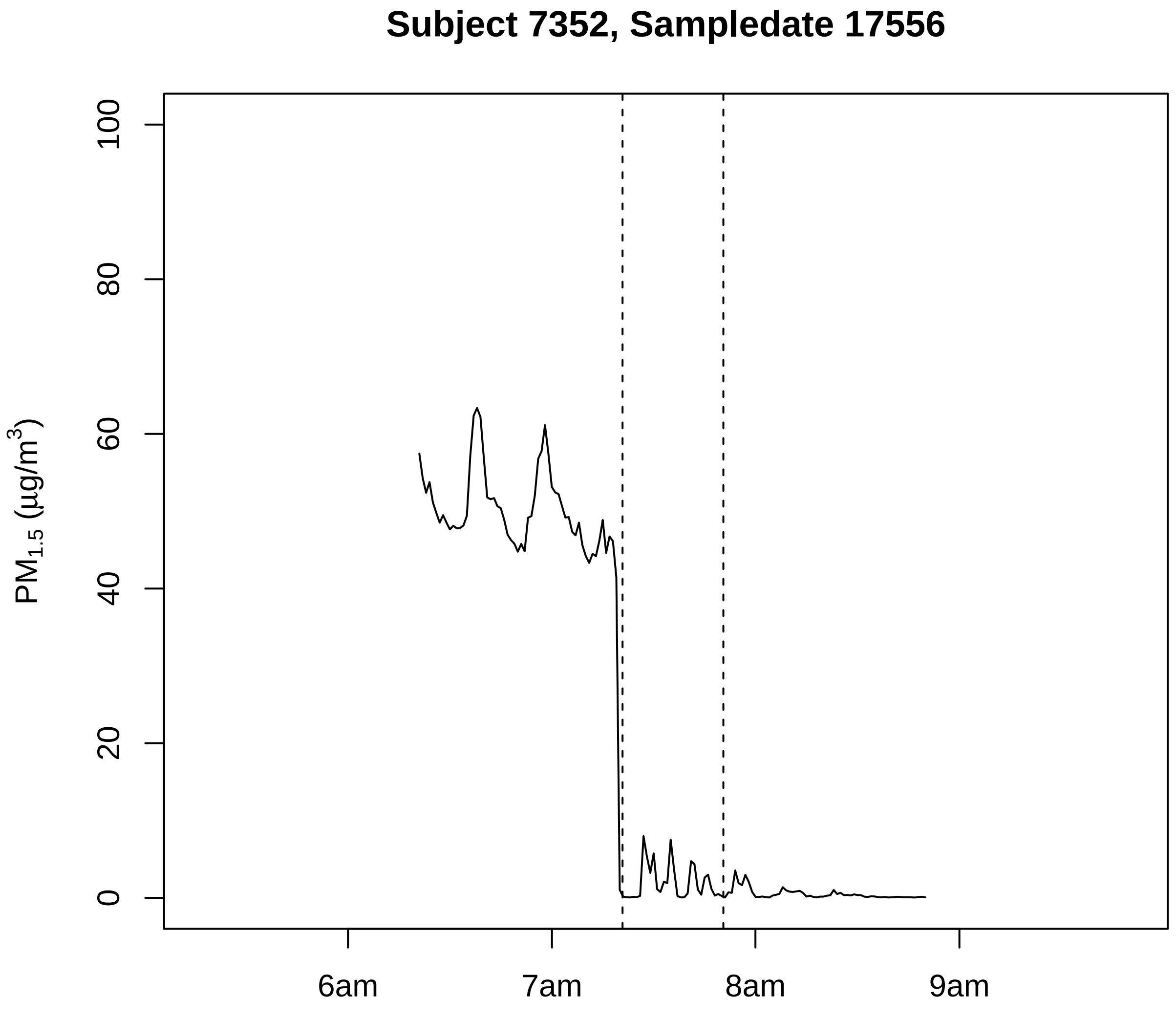
Subject 7352, Sampledate 17556
PM1.5 (µg/m3)
0
20
40
60
80
100
6am
7am
8am
9am
Time of day

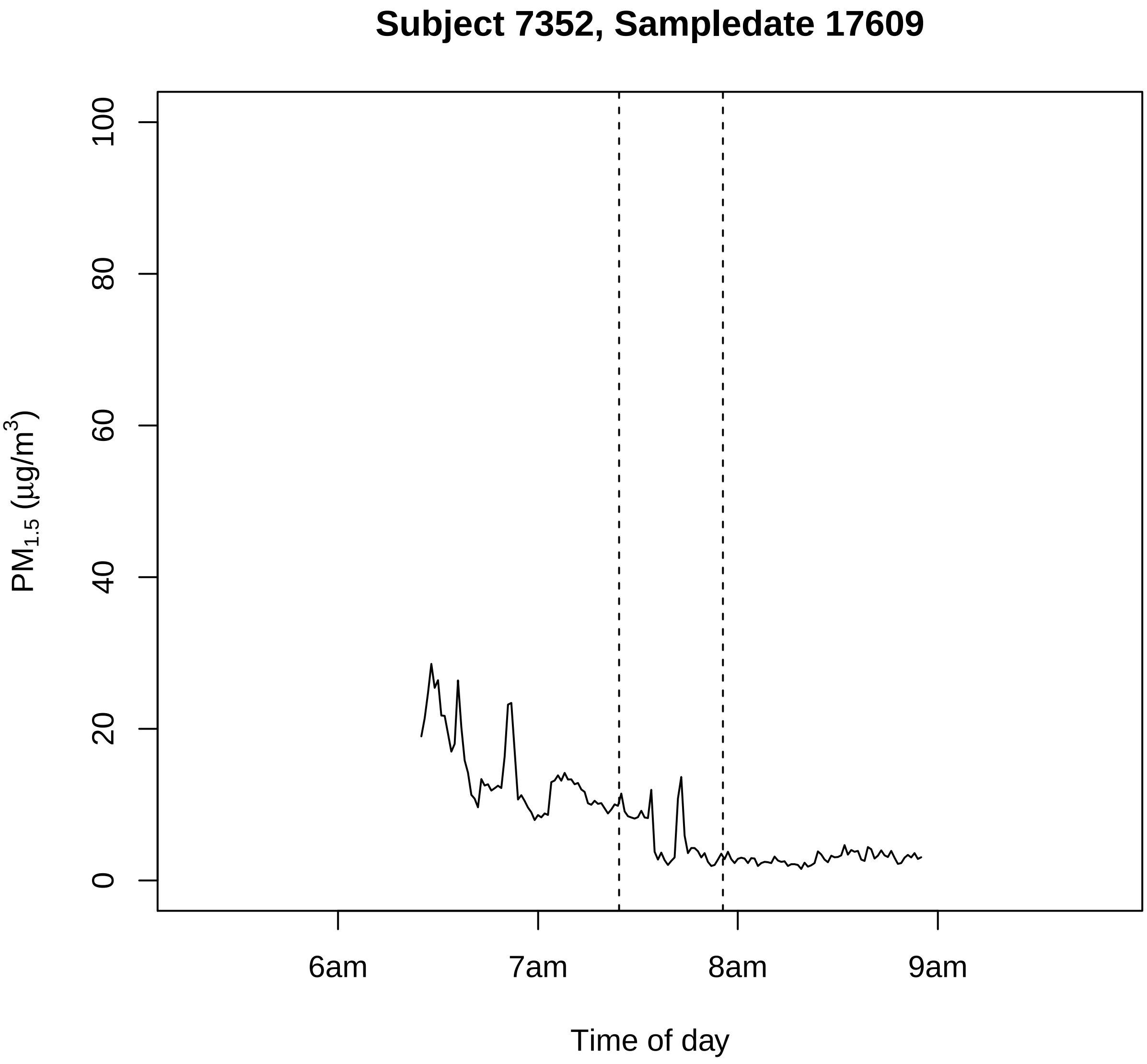

Subject 7352, Sampledate 17609
0
20
40
60
80
100
$PM_{1.5}$ (µg/m^3)
6am
7am
8am
9am
Time of day

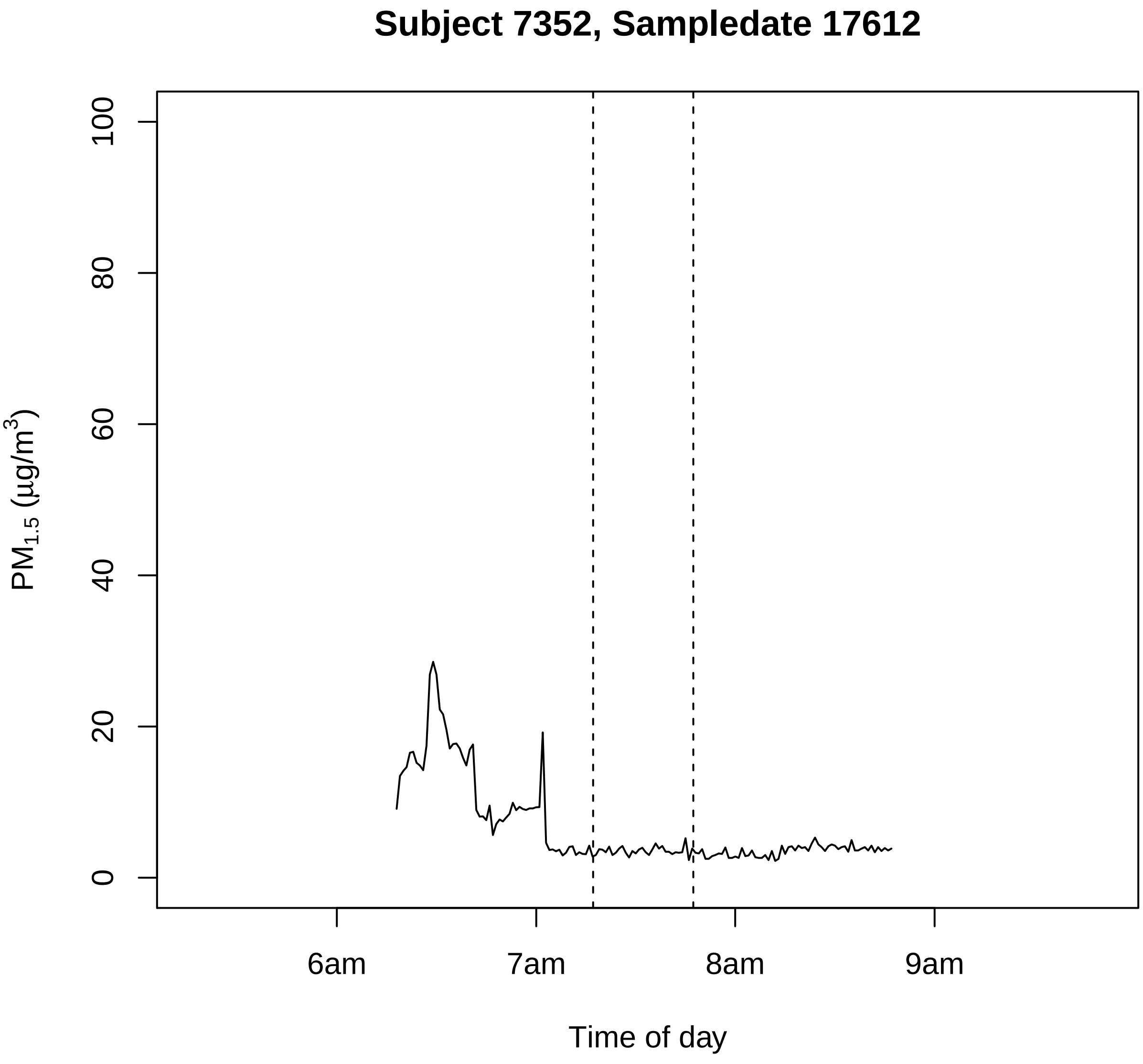
Subject 7352, Sampledate 17612
PM1.5 (µg/m3)
0
20
40
60
80
100
6am
7am
8am
9am
Time of day

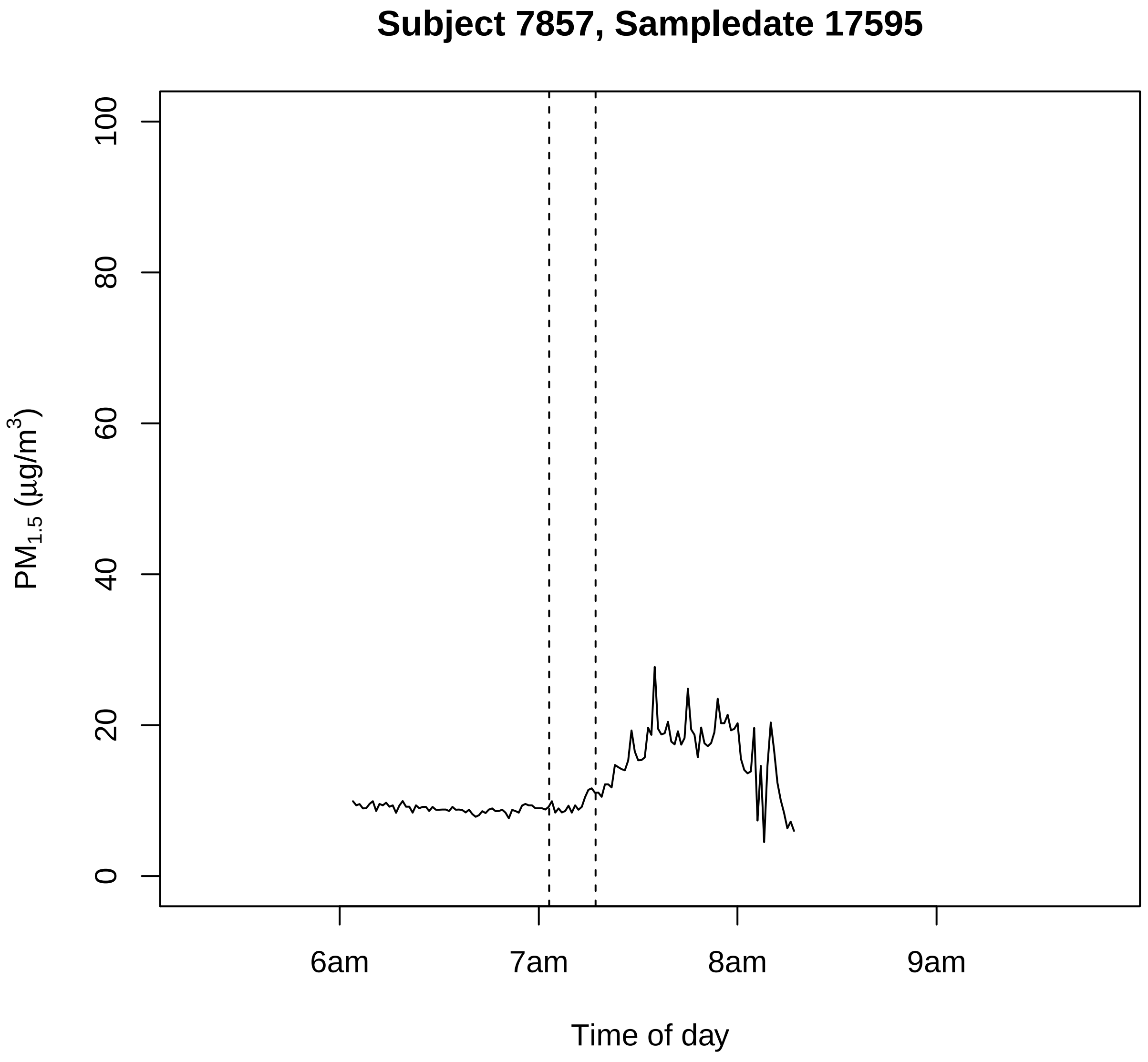

Subject 7857, Sampledate 17595
PM1.5 (µg/m3)
0
20
40
60
80
100
6am
7am
8am
9am
Time of day

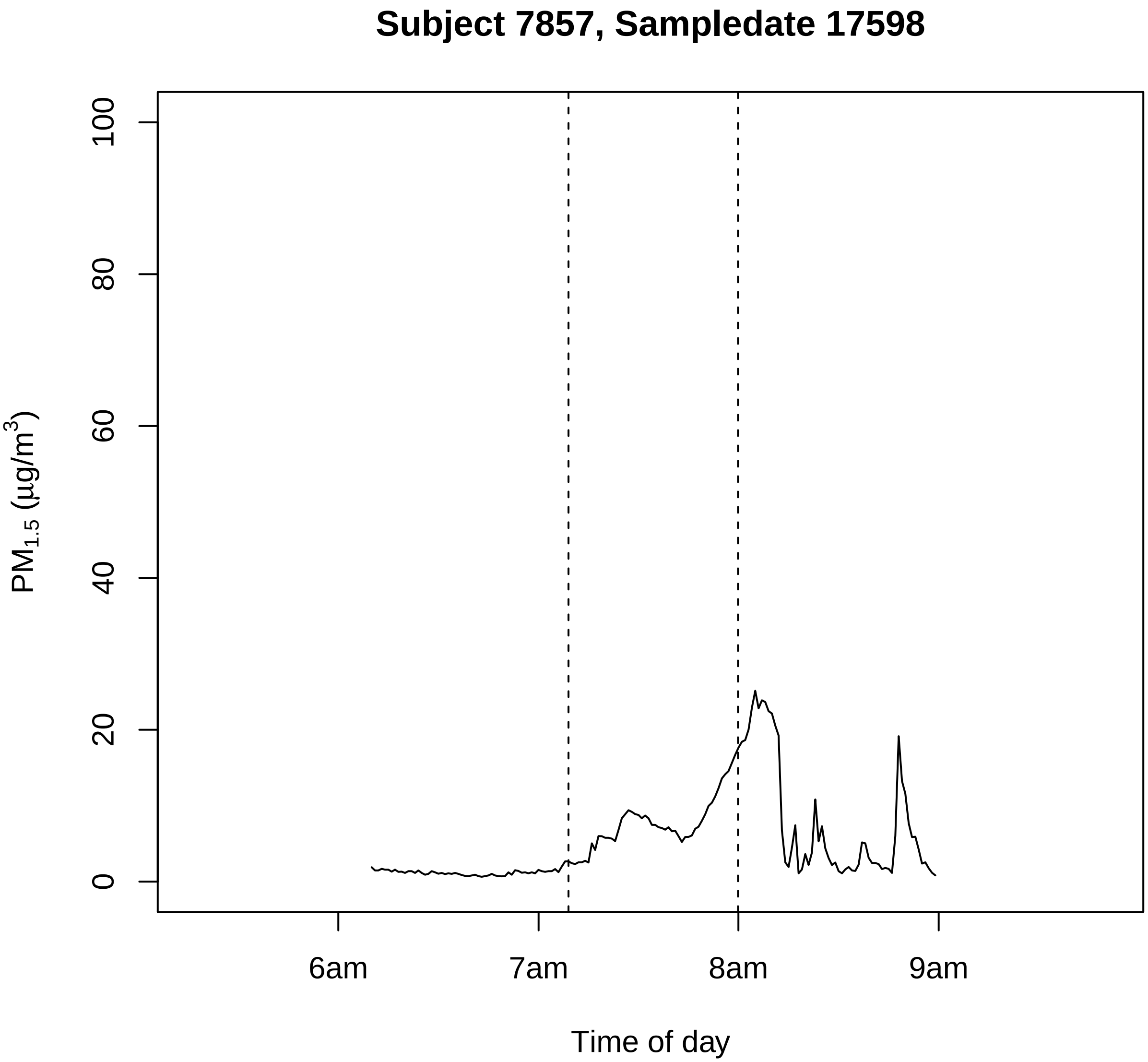

Subject 7857, Sampledate 17598
$PM_{1.5}$ (µg/m^3)
0
20
40
60
80
100
6am
7am
8am
9am
Time of day

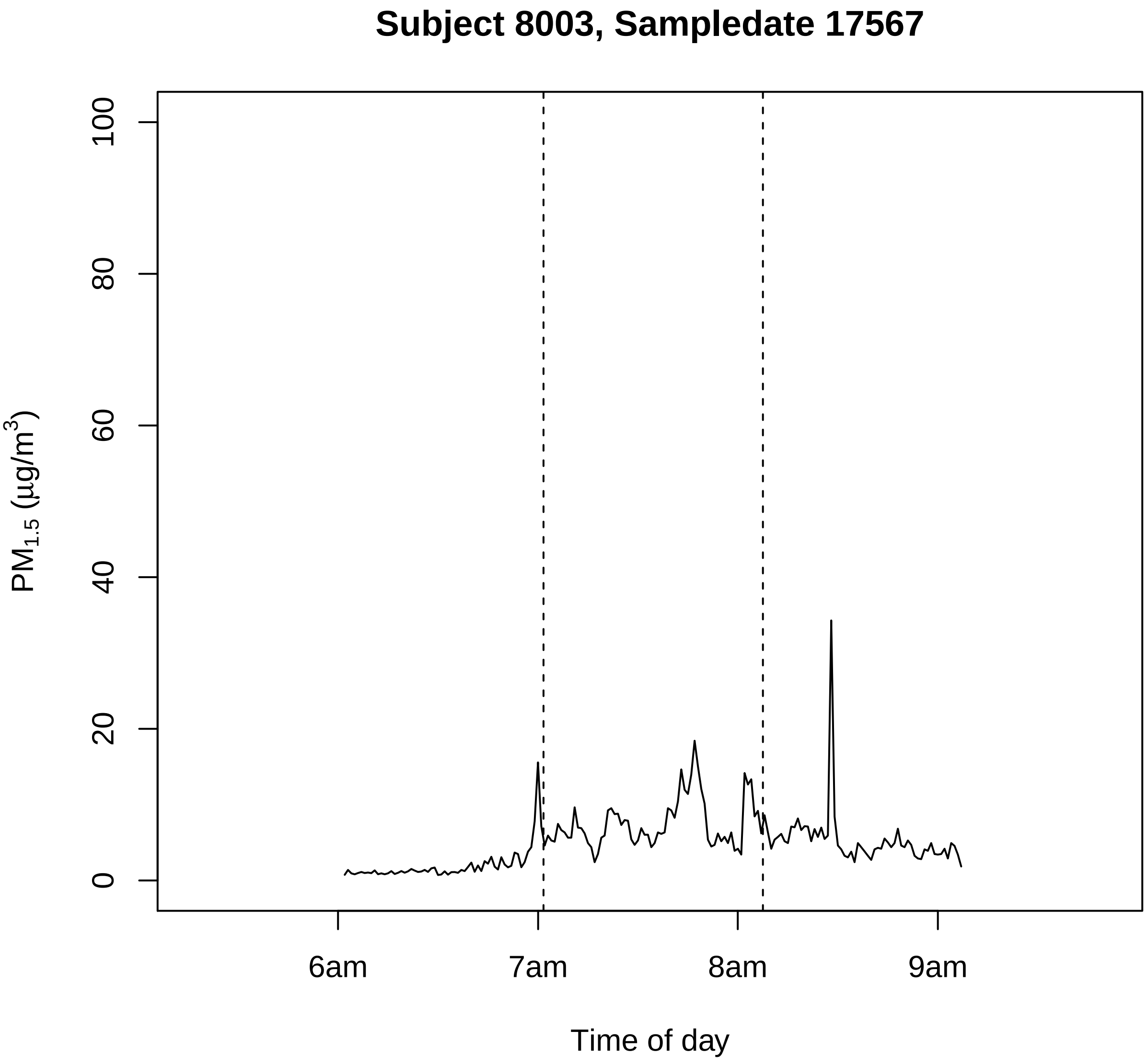

Subject 8003, Sampledate 17567
0
20
40
60
80
100
$PM_{1.5}$ (µg/m^3)
6am
7am
8am
9am
Time of day

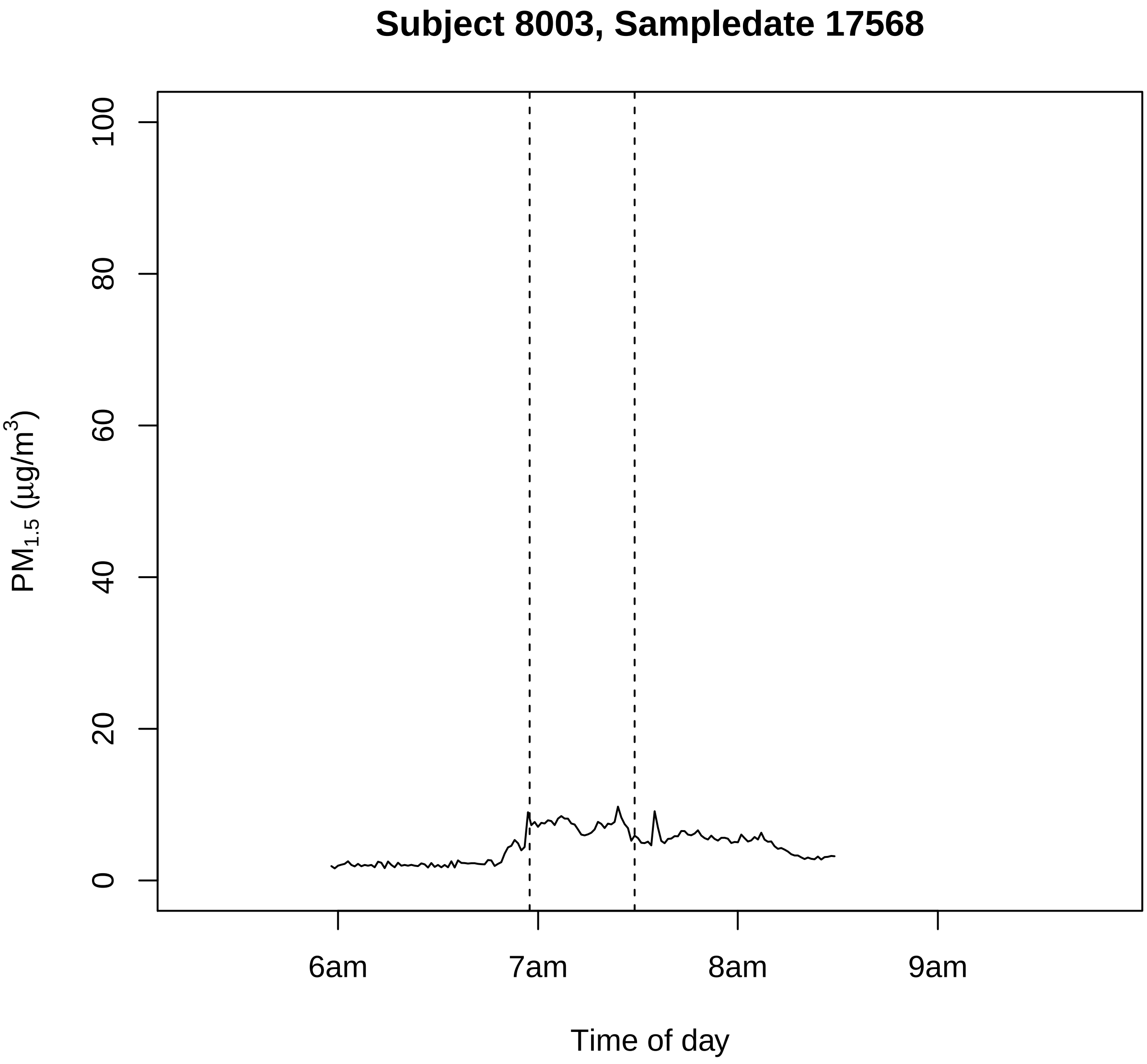
Subject 8003, Sampledate 17568
PM1.5 (µg/m3)
0
20
40
60
80
100
6am
7am
8am
9am
Time of day

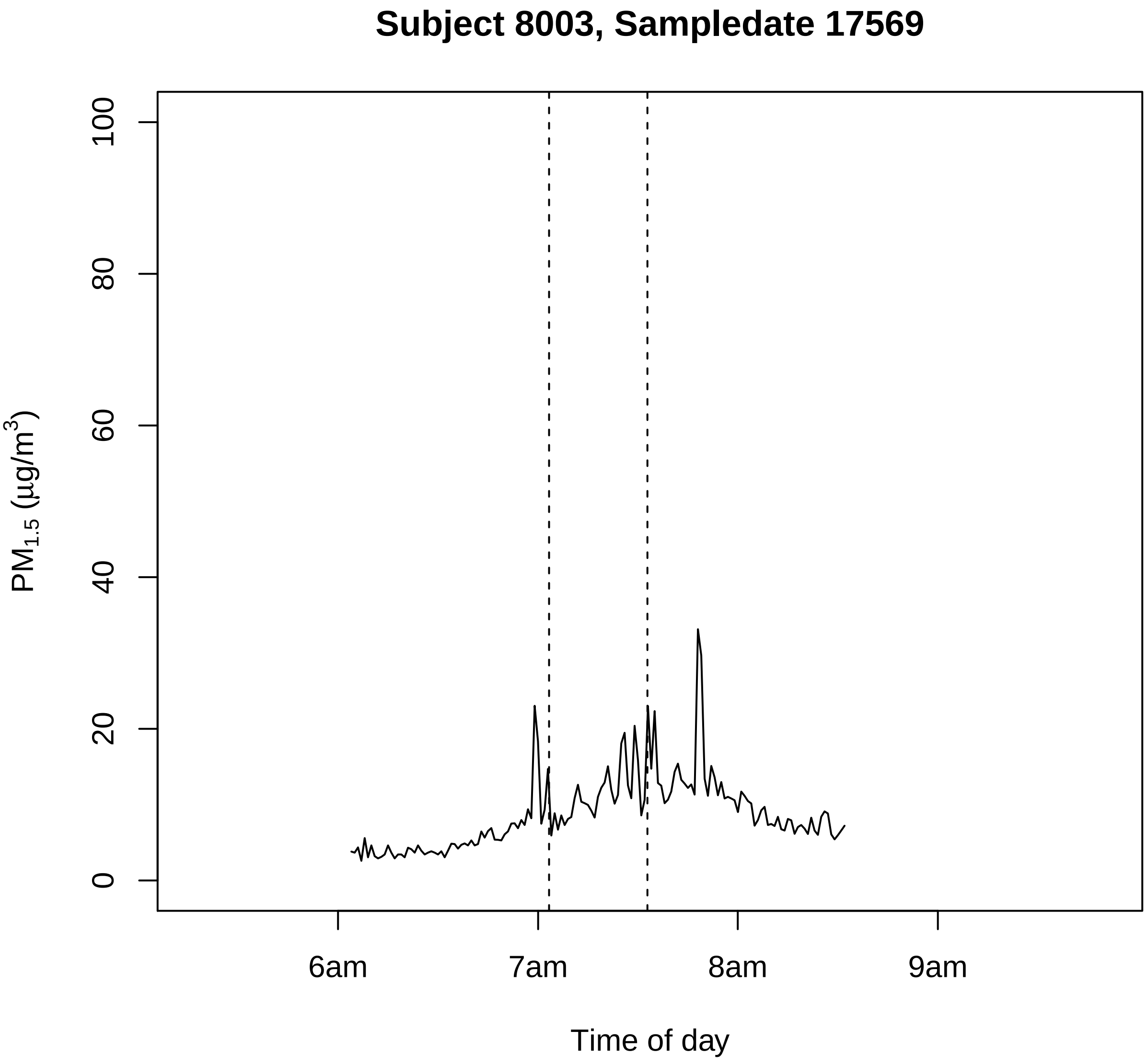

Subject 8003, Sampledate 17569
100
80
60
40
20
0
PM1.5 (µg/m3)
6am
7am
8am
9am
Time of day

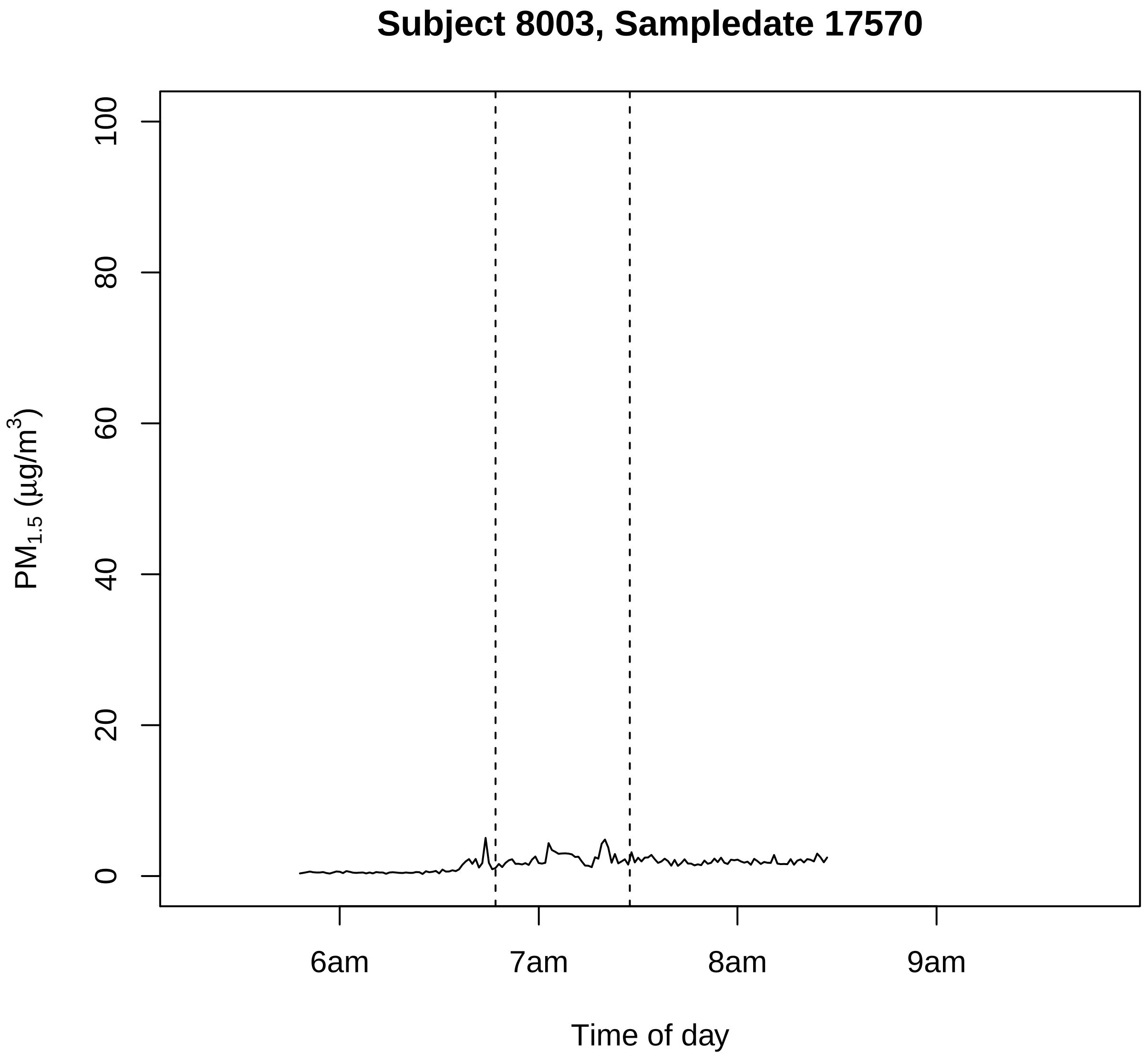
Subject 8003, Sampledate 17570
PM1.5 (µg/m3)
0
20
40
60
80
100
6am
7am
8am
9am
Time of day

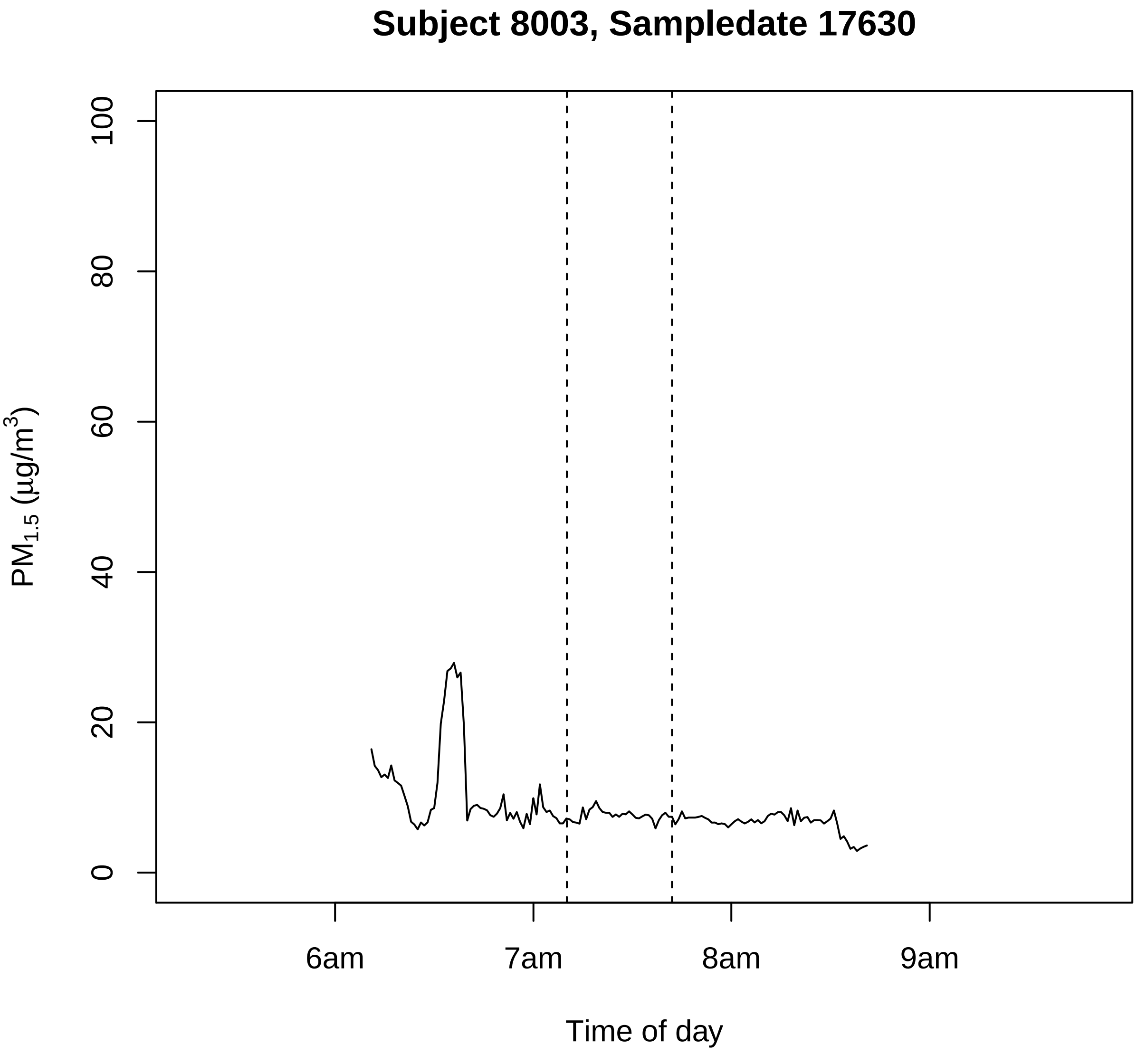

Subject 8003, Sampledate 17630
PM1.5 (µg/m3)
0
20
40
60
80
100
6am
7am
8am
9am
Time of day

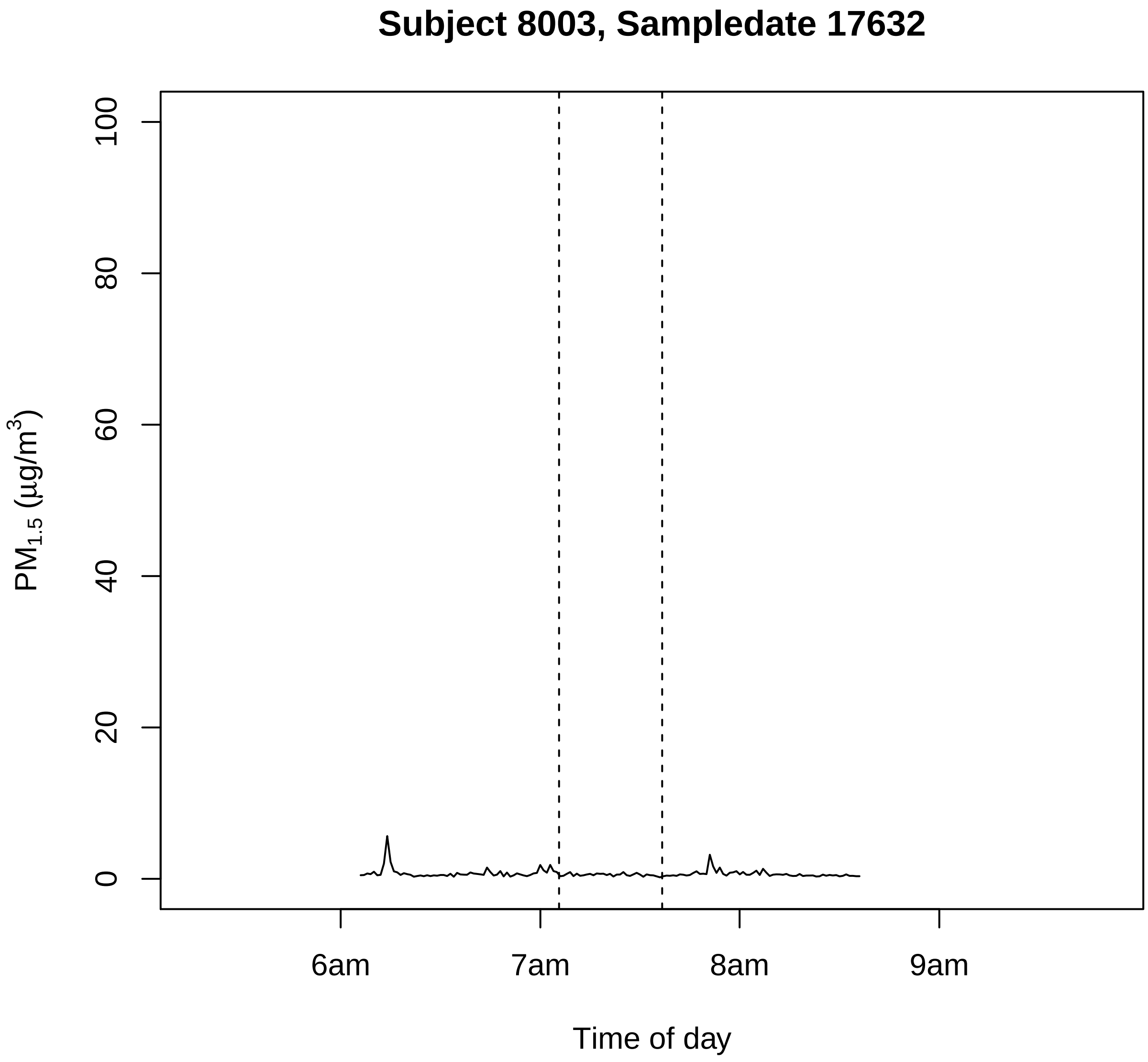

Subject 8003, Sampledate 17632
PM1.5 (µg/m3)
0
20
40
60
80
100
6am
7am
8am
9am
Time of day

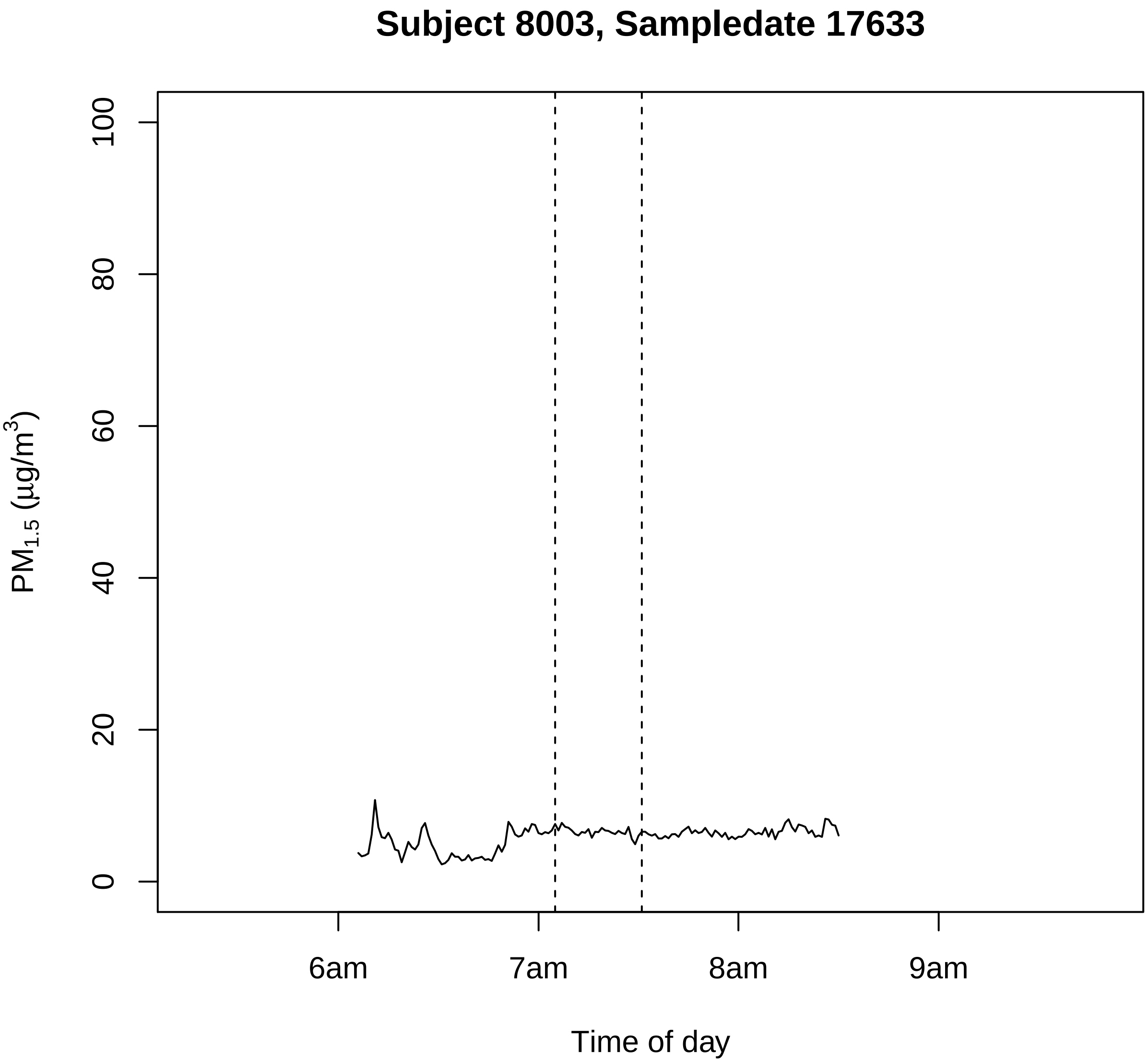

Subject 8003, Sampledate 17633
PM1.5 (µg/m3)
0
20
40
60
80
100
6am
7am
8am
9am
Time of day

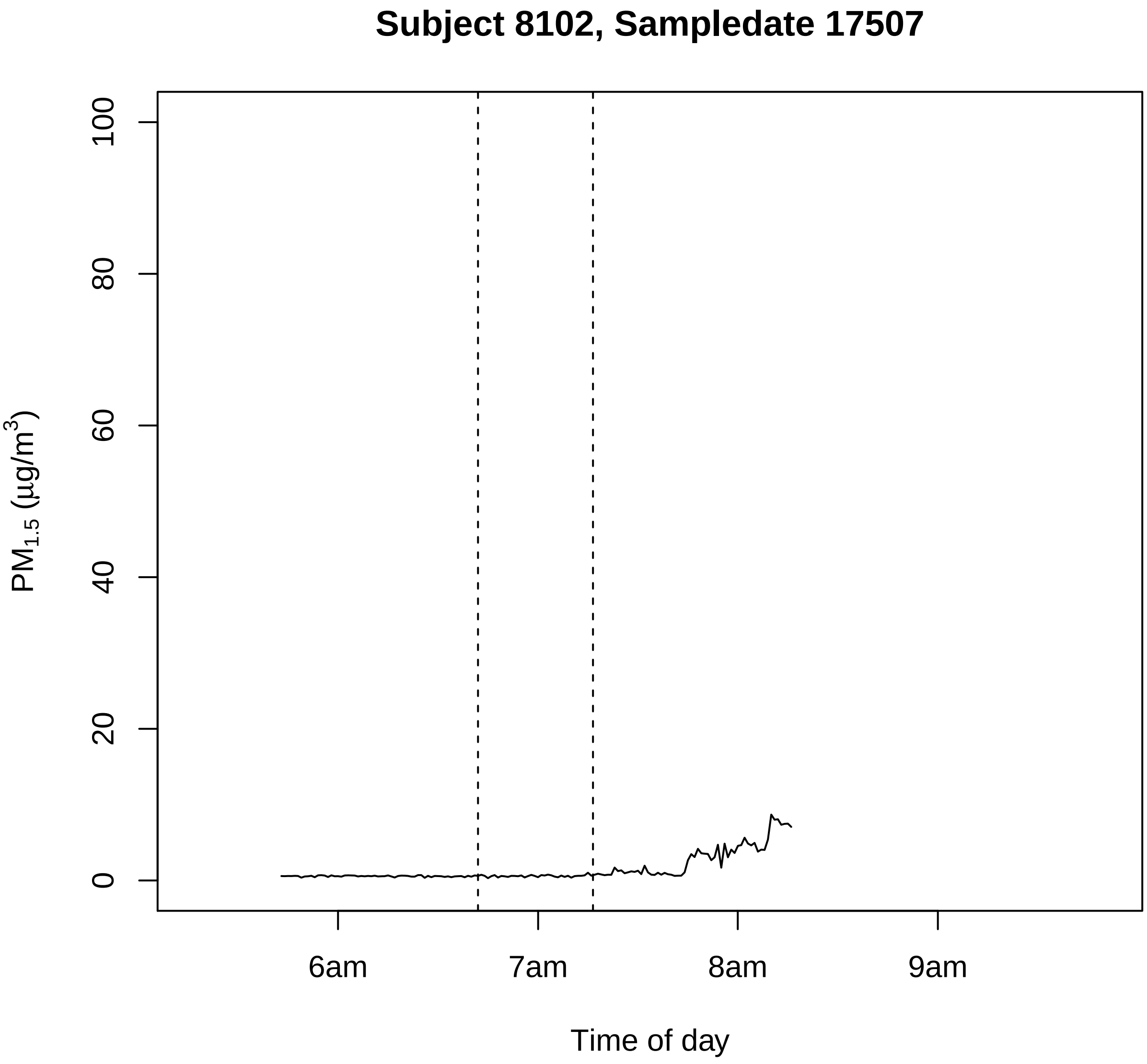

Subject 8102, Sampledate 17507
PM1.5 (µg/m3)
0
20
40
60
80
100
6am
7am
8am
9am
Time of day

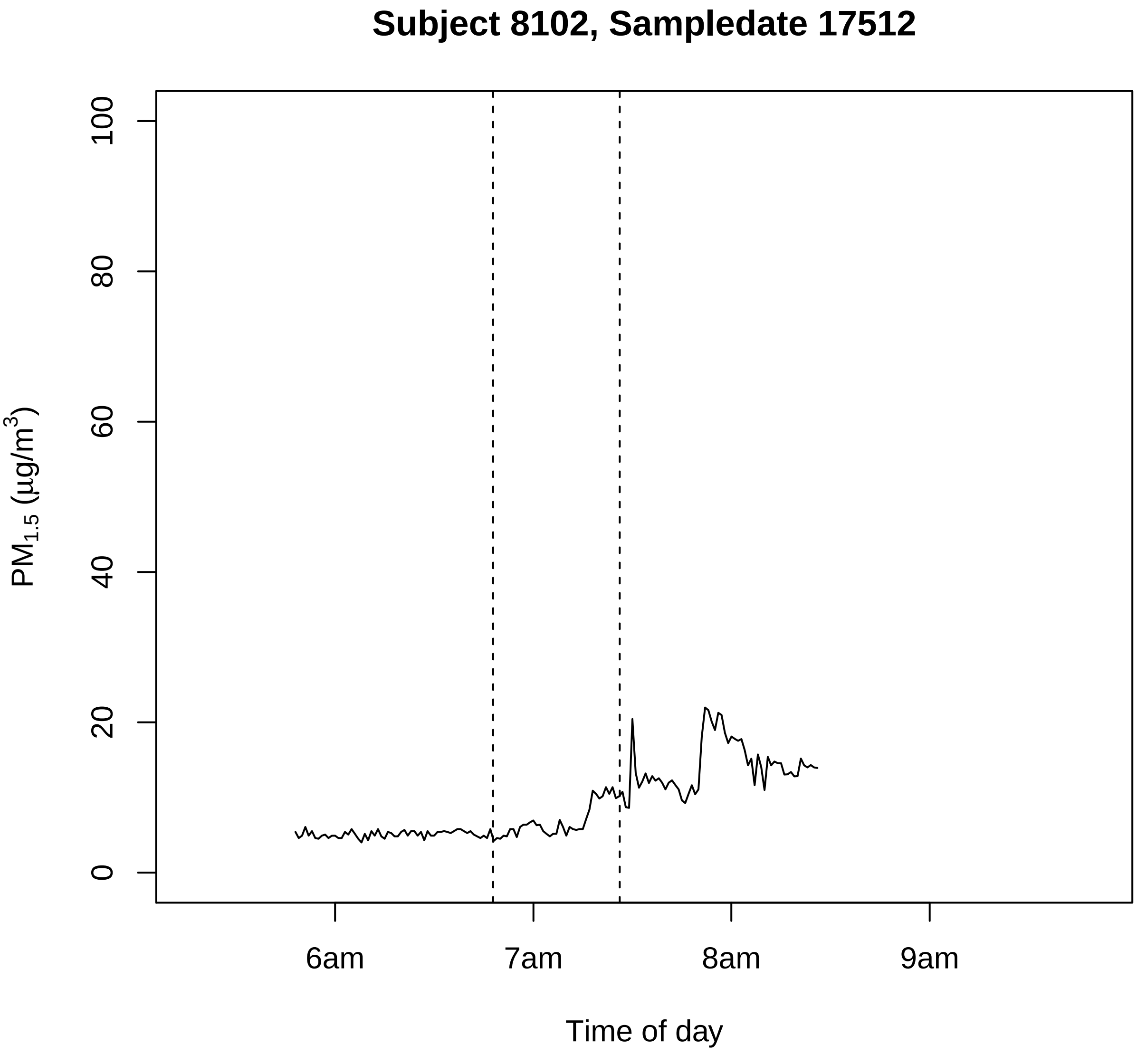

Subject 8102, Sampledate 17512
PM1.5 (µg/m3)
0
20
40
60
80
100
6am
7am
8am
9am
Time of day

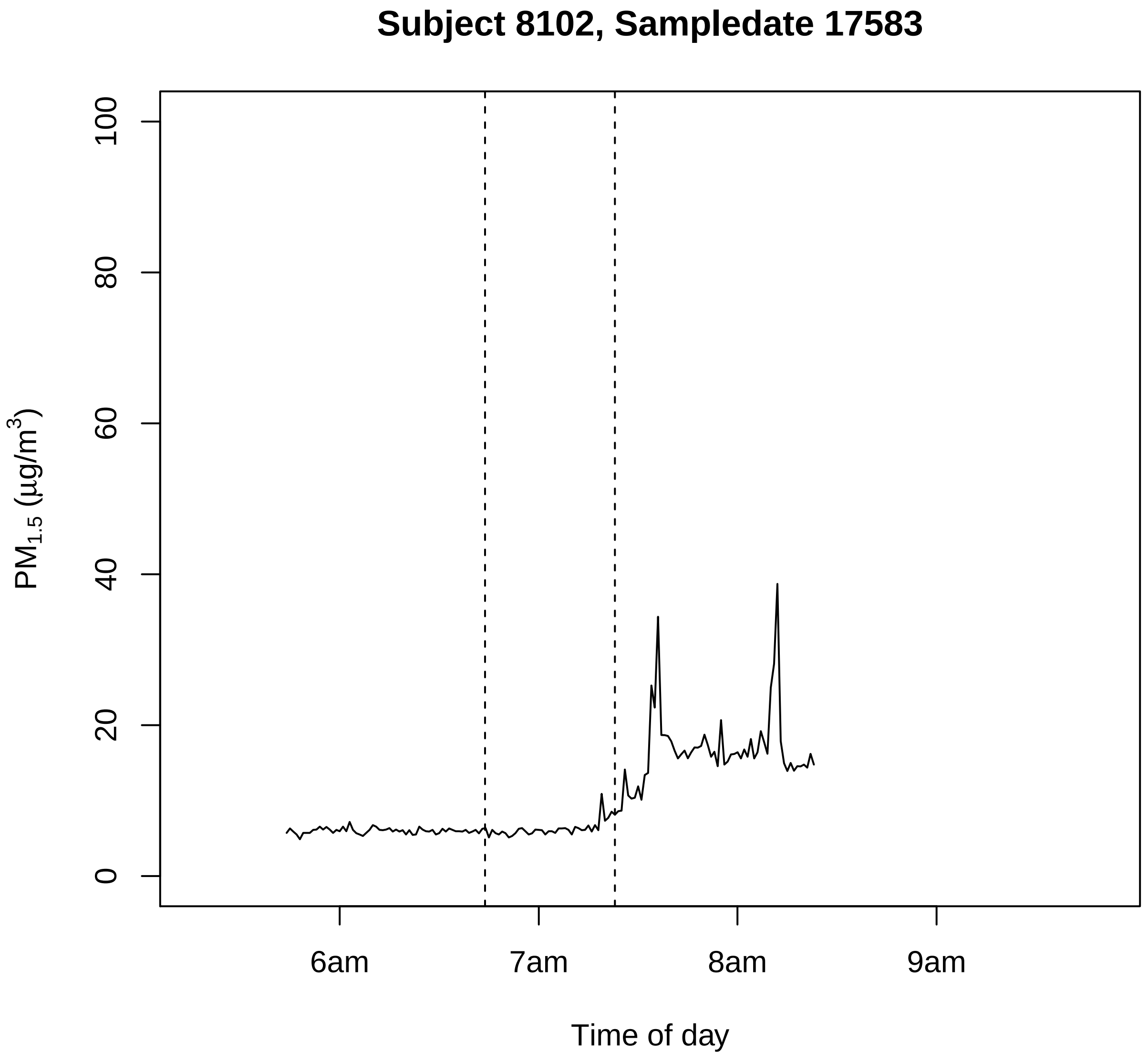

Subject 8102, Sampledate 17583
0
20
40
60
80
100
PM1.5 (µg/m3)
6am
7am
8am
9am
Time of day

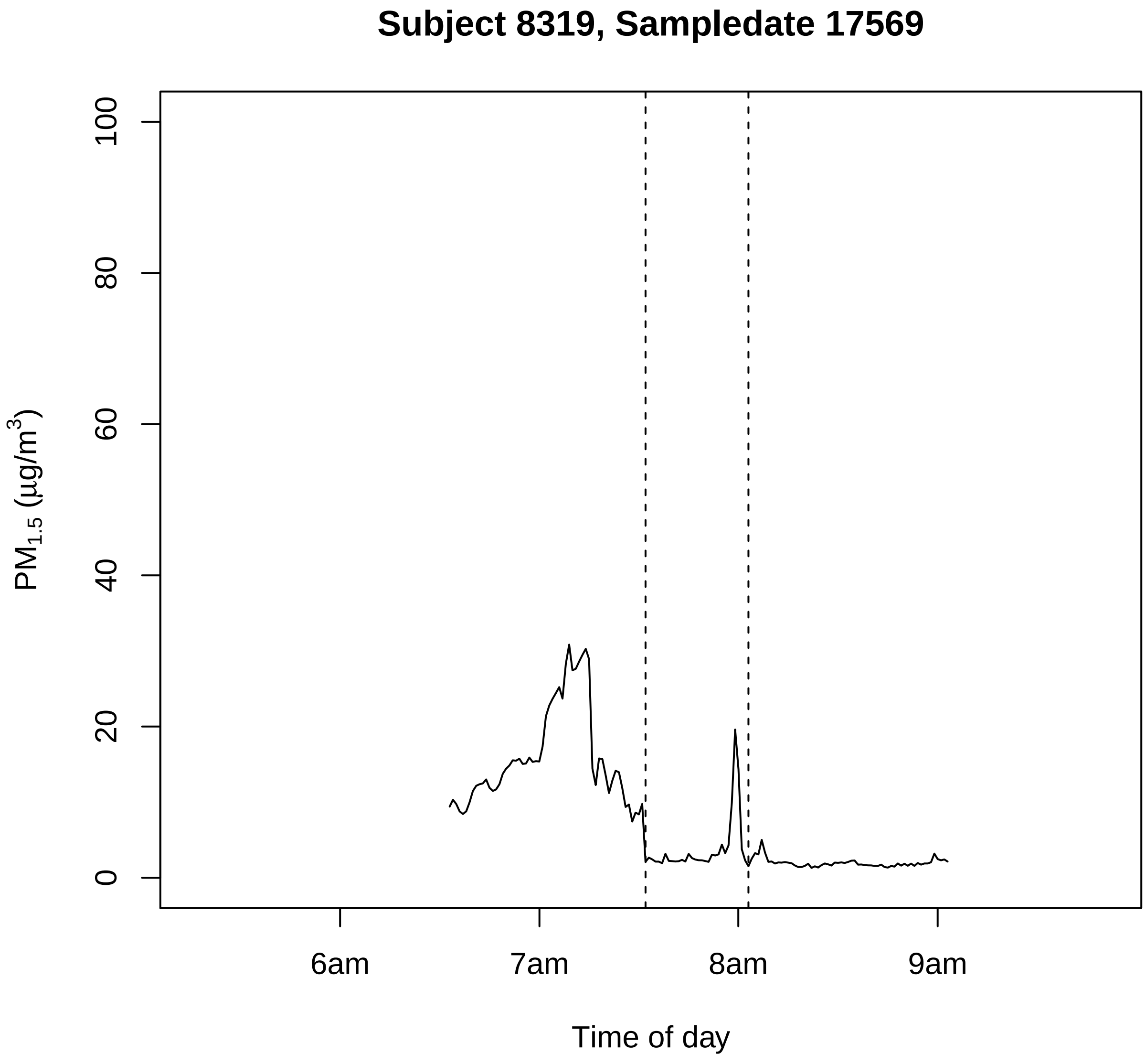

Subject 8319, Sampledate 17569
$PM_{1.5}$ (µg/m³)
0
20
40
60
80
100
6am
7am
8am
9am
Time of day

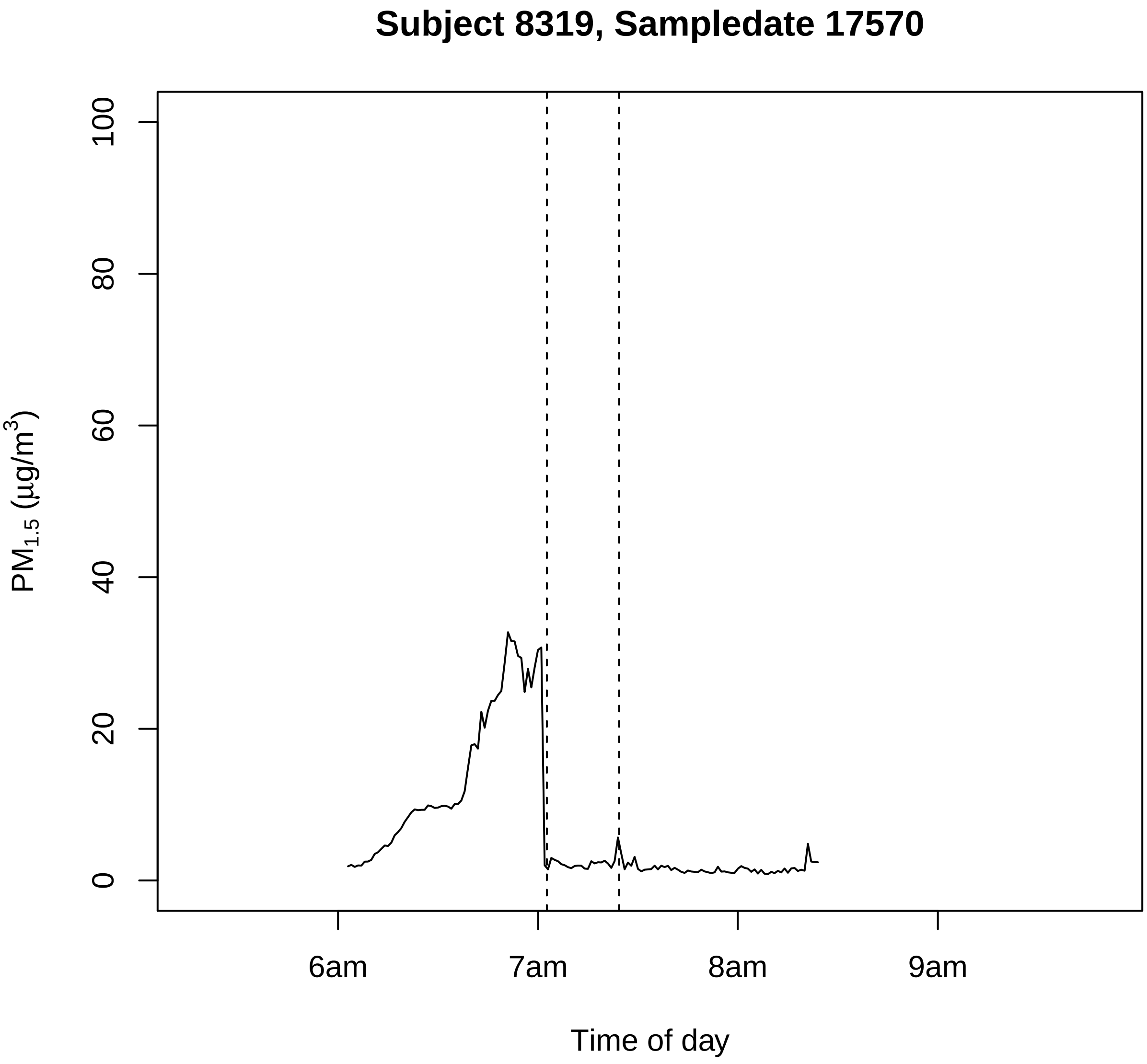

Subject 8319, Sampledate 17570
PM1.5 (μg/m3)
0
20
40
60
80
100
6am
7am
8am
9am
Time of day

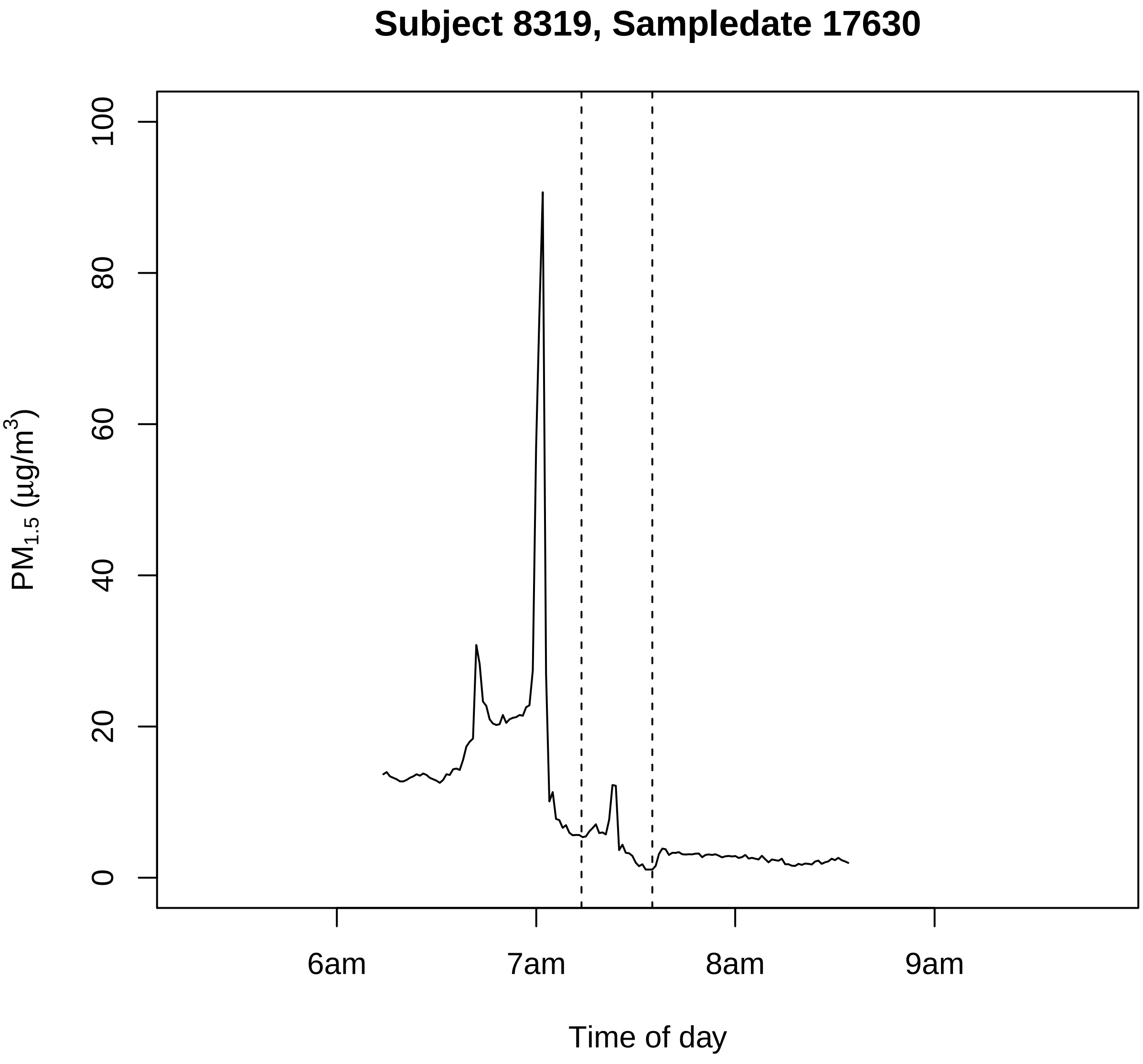

Subject 8319, Sampledate 17630
$PM_{1.5}$ (µg/m^3)
0
20
40
60
80
100
6am
7am
8am
9am
Time of day

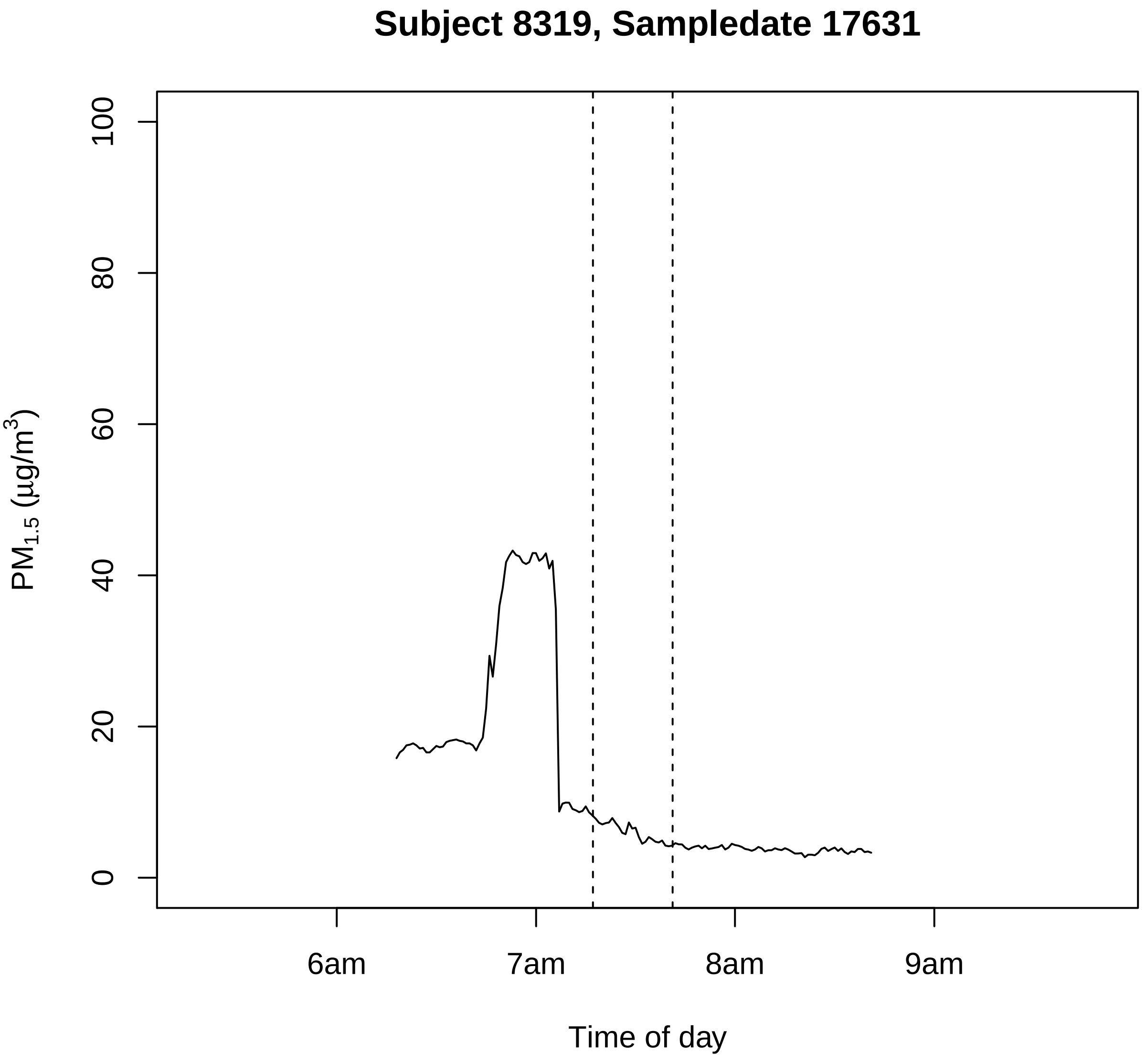
Subject 8319, Sampledate 17631
PM1.5 (µg/m3)
0
20
40
60
80
100
6am
7am
8am
9am
Time of day

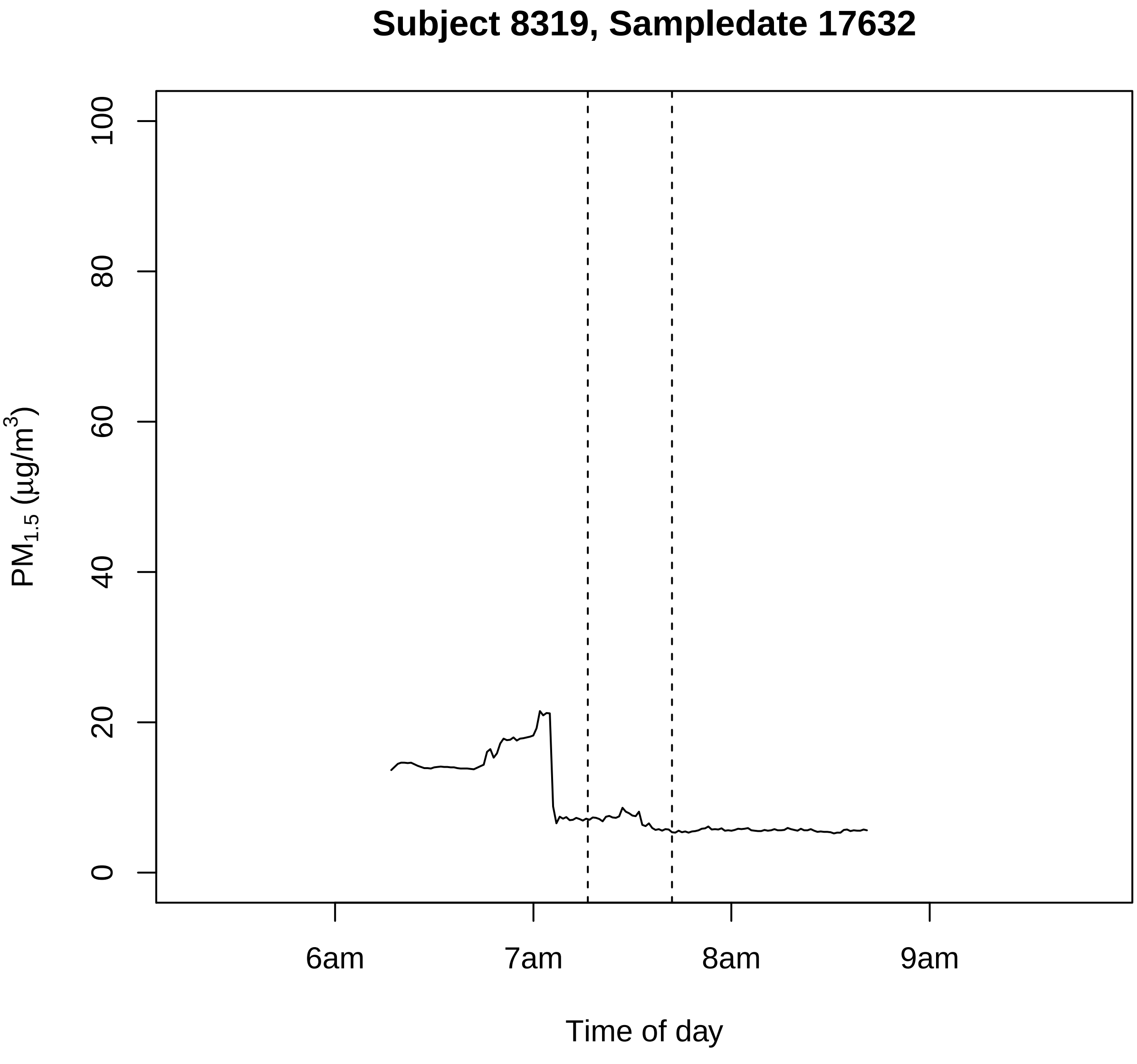
Subject 8319, Sampledate 17632
PM1.5 (µg/m3)
0
20
40
60
80
100
6am
7am
8am
9am
Time of day

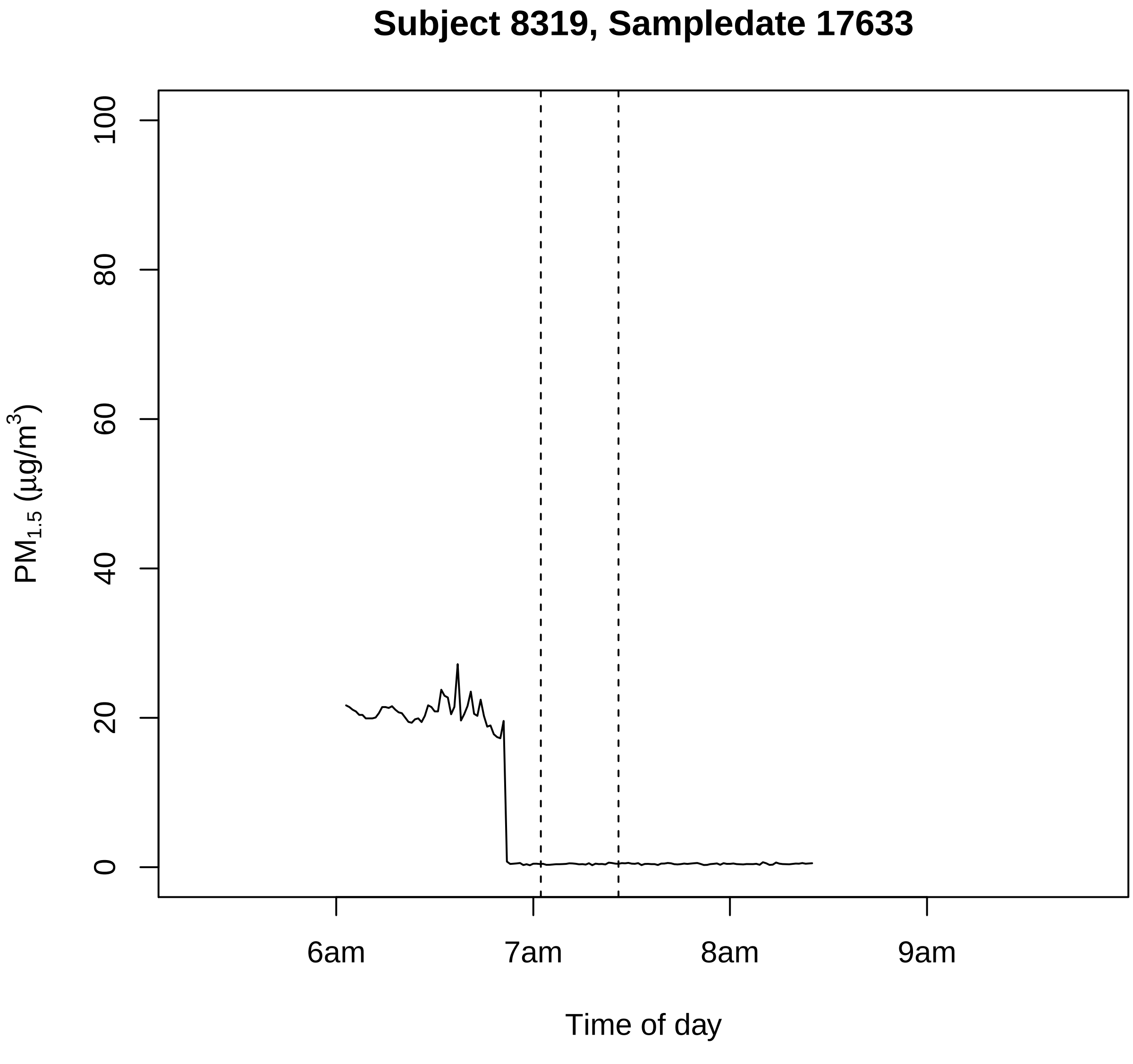
Subject 8319, Sampledate 17633
PM1.5 (µg/m3)
100
80
60
40
20
0
6am
7am
8am
9am
Time of day

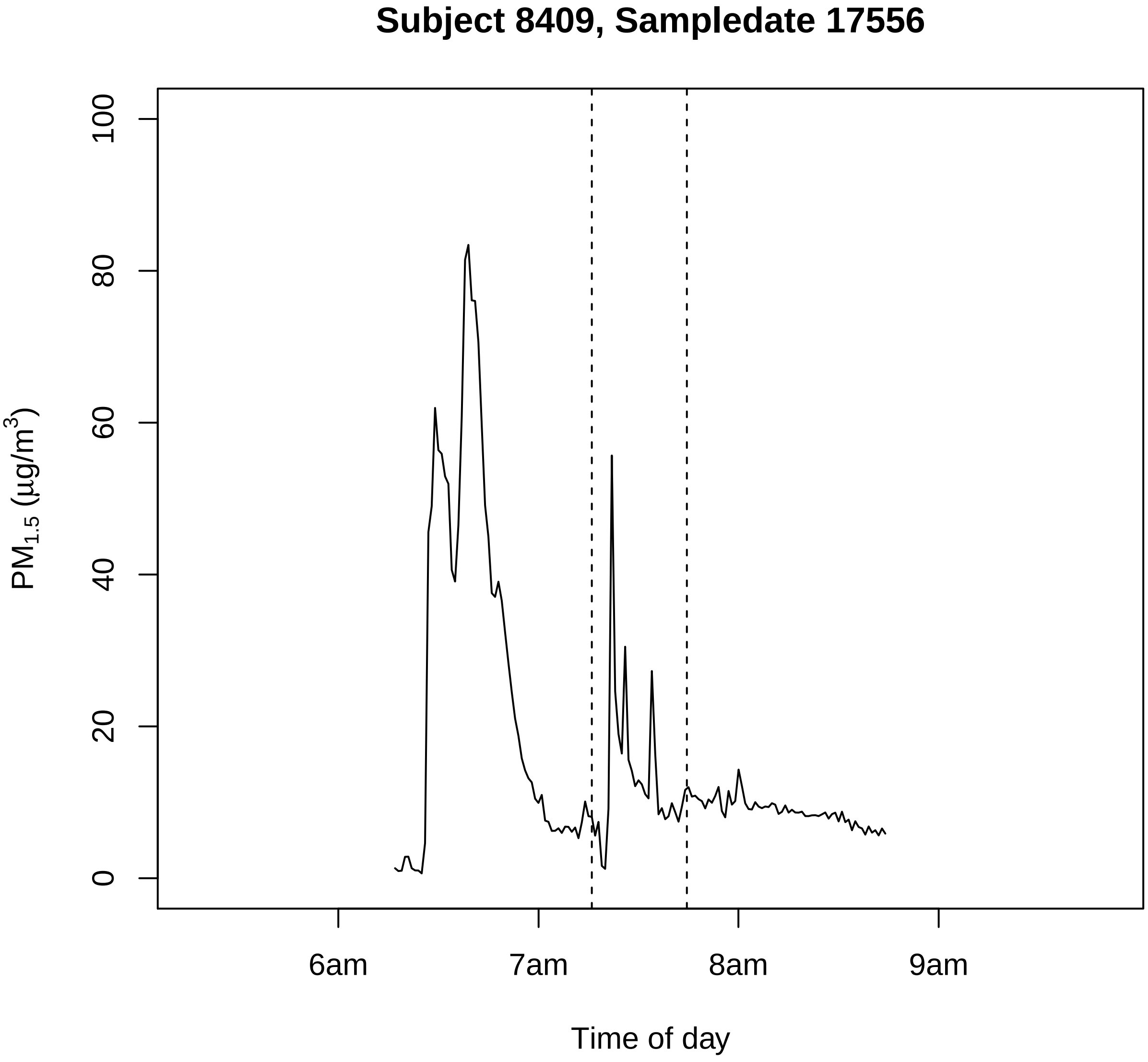

Subject 8409, Sampledate 17556
0
20
40
60
80
100
$PM_{1.5}$ (µg/m^3)
6am
7am
8am
9am
Time of day

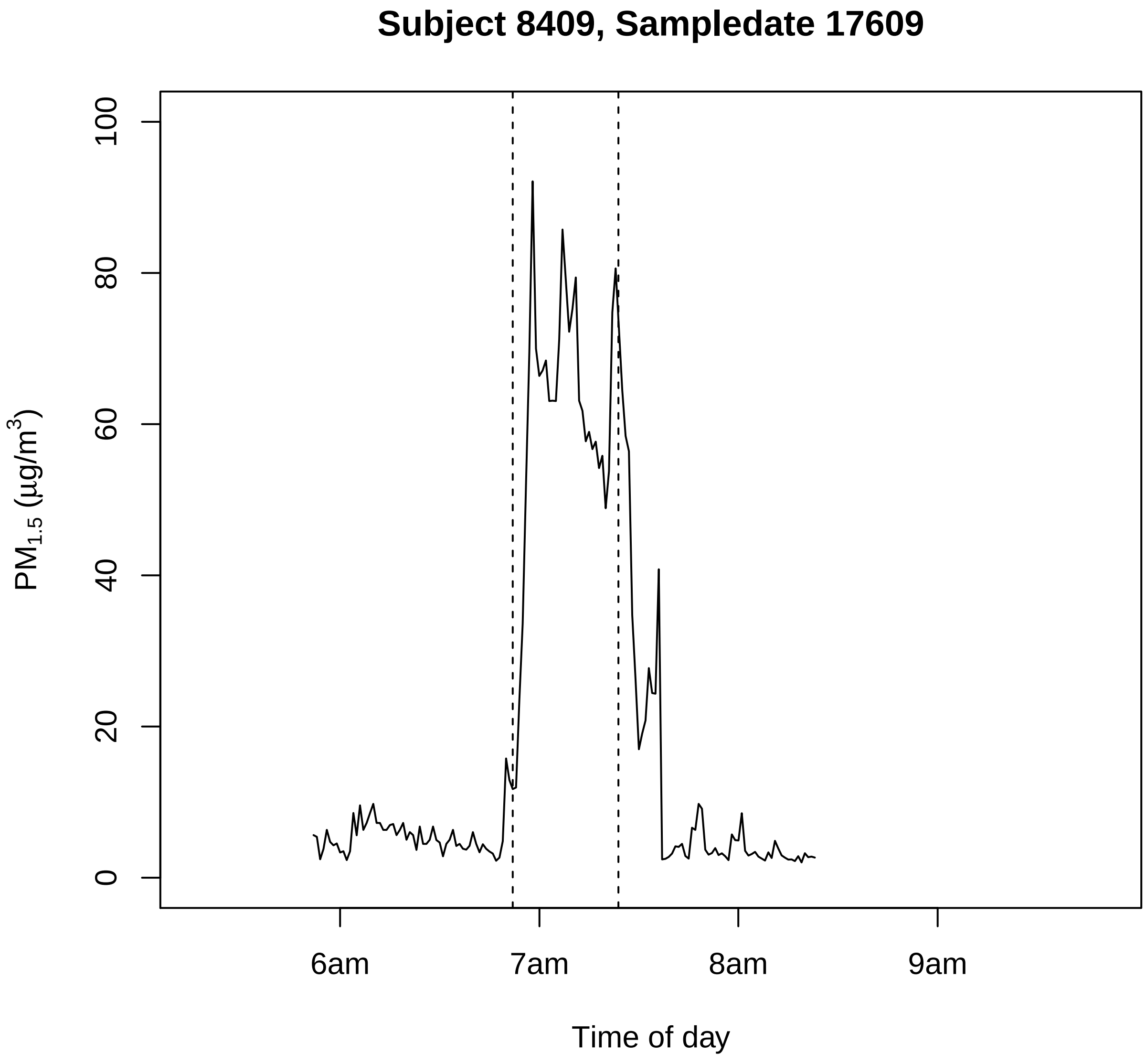

Subject 8409, Sampledate 17609
PM1.5 (µg/m3)
0
20
40
60
80
100
6am
7am
8am
9am
Time of day

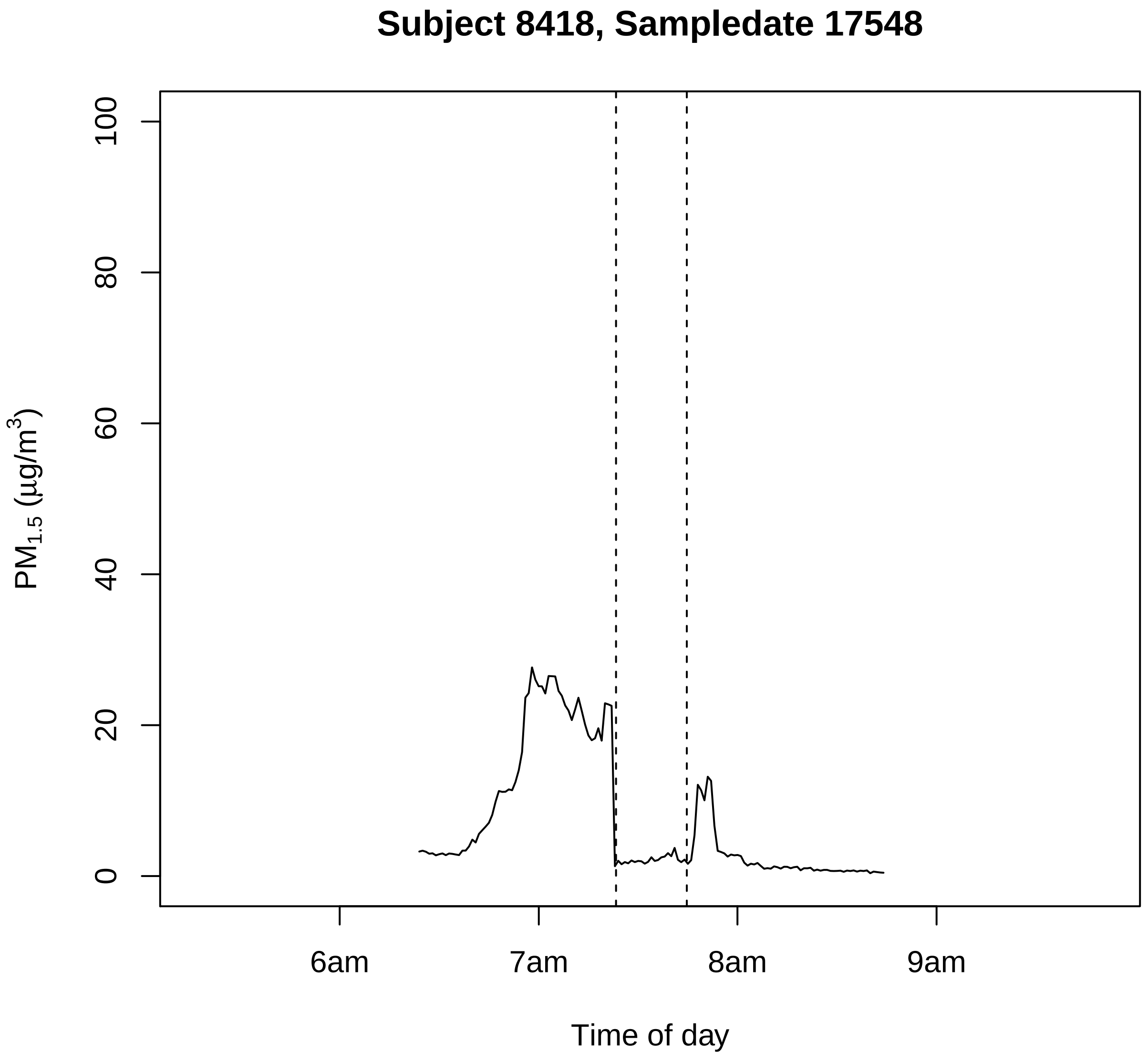
Subject 8418, Sampledate 17548
PM1.5 (µg/m3)
0
20
40
60
80
100
6am
7am
8am
9am
Time of day

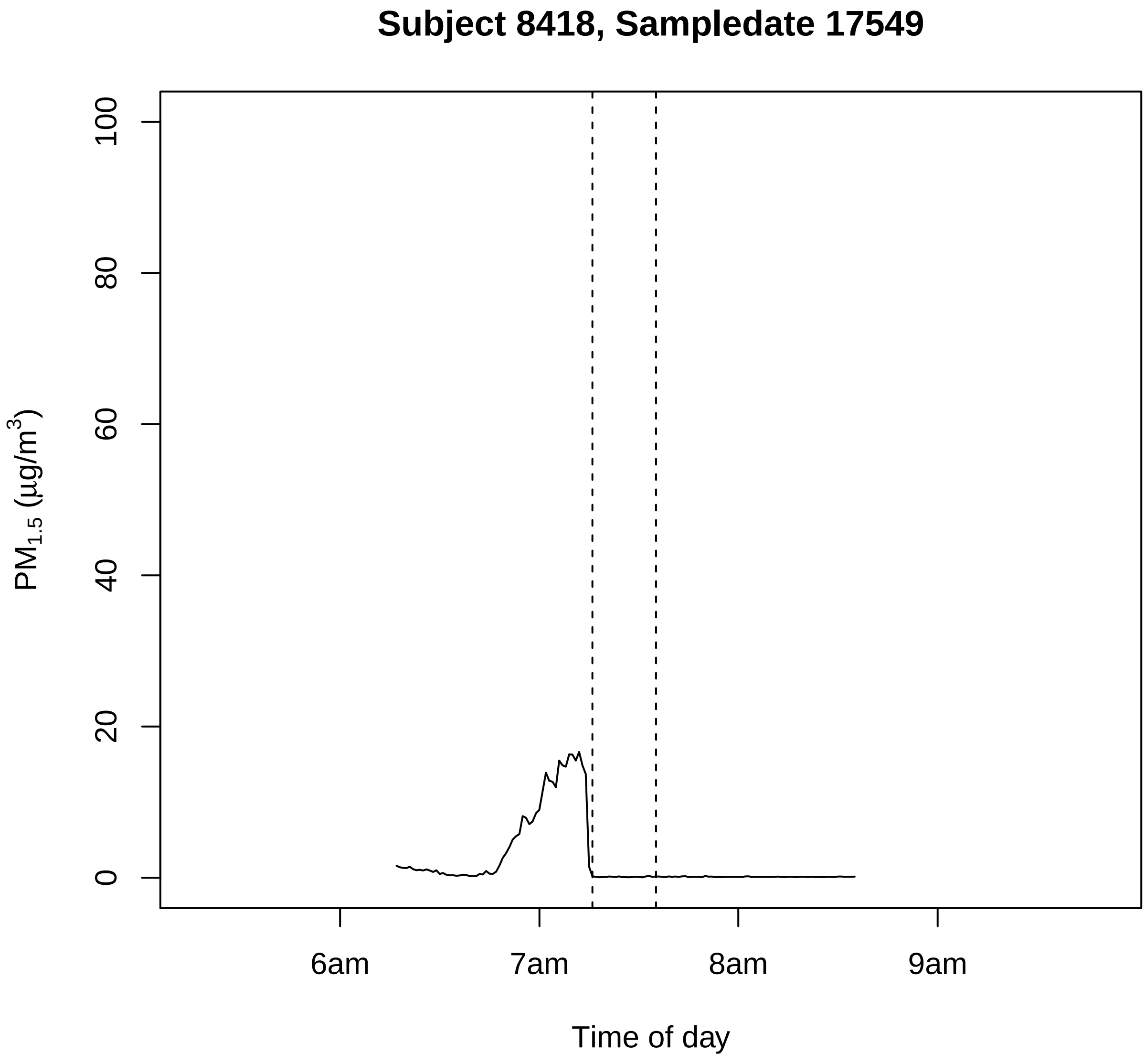
Subject 8418, Sampledate 17549
PM1.5 (µg/m3)
0
20
40
60
80
100
6am
7am
8am
9am
Time of day

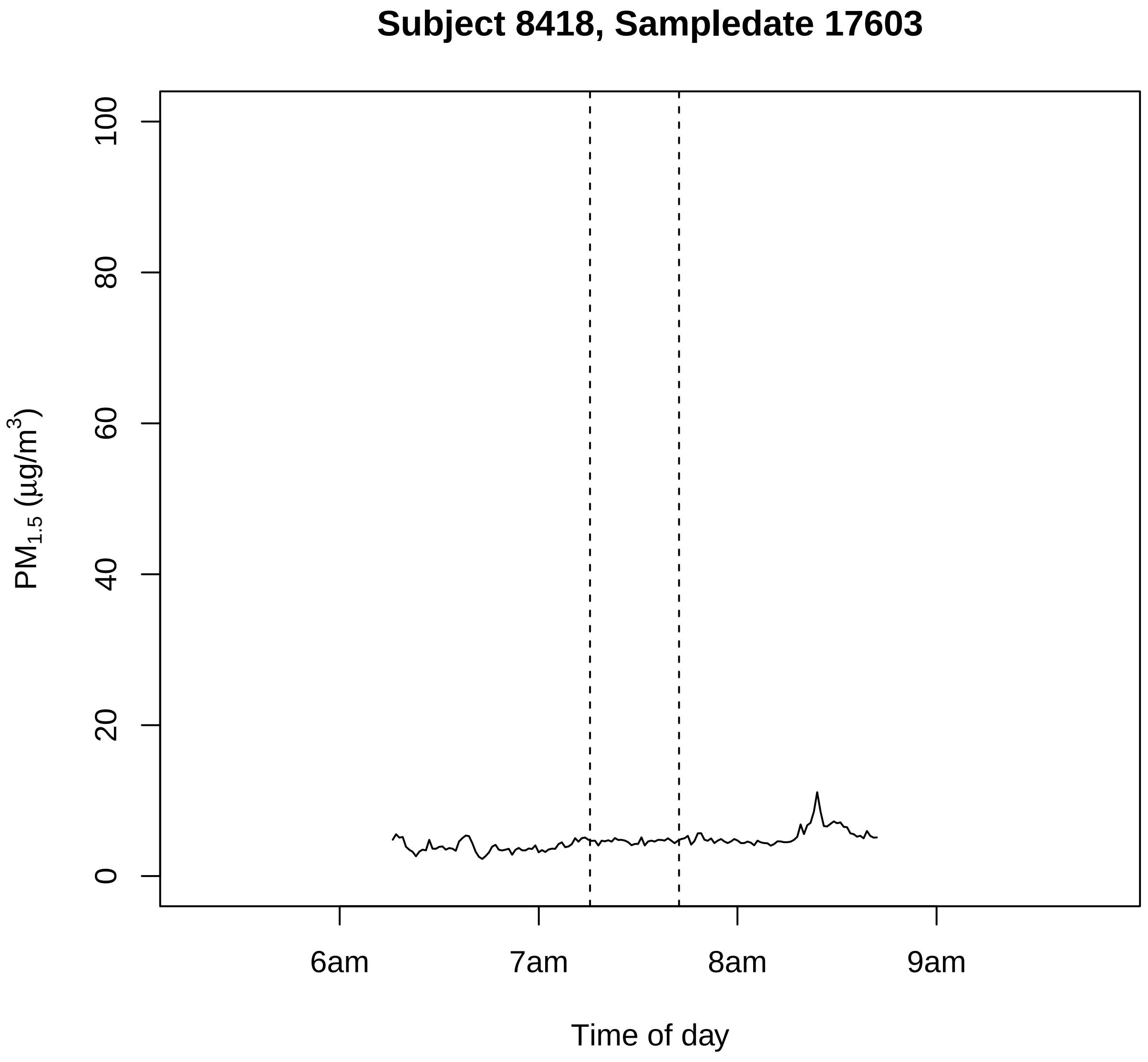

Subject 8418, Sampledate 17603
PM1.5 (µg/m3)
0
20
40
60
80
100
6am
7am
8am
9am
Time of day

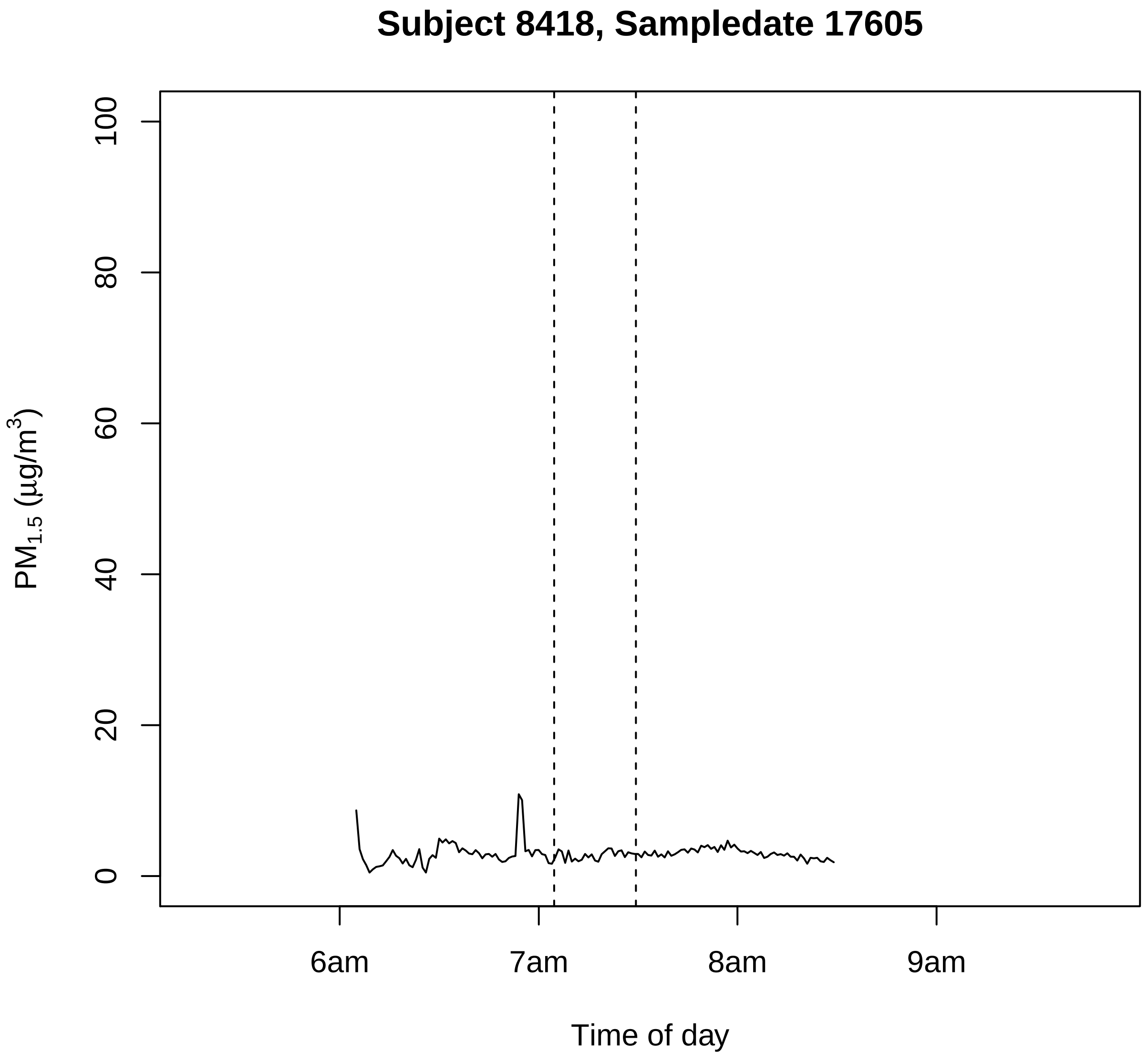

Subject 8418, Sampledate 17605
0
20
40
60
80
100
PM1.5 (µg/m3)
6am
7am
8am
9am
Time of day

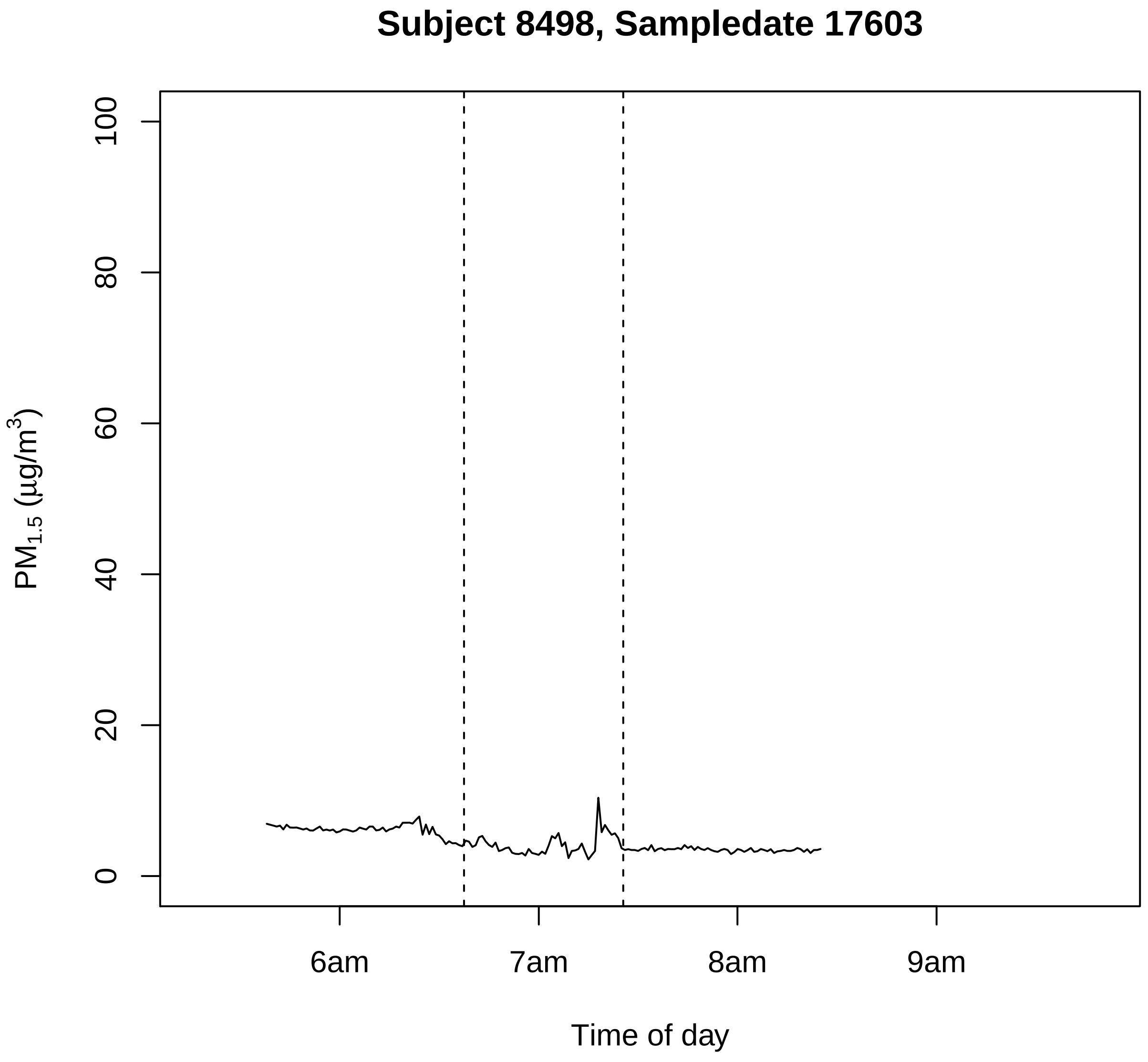
Subject 8498, Sampledate 17603
0
20
40
60
80
100
PM1.5 (µg/m3)
6am
7am
8am
9am
Time of day

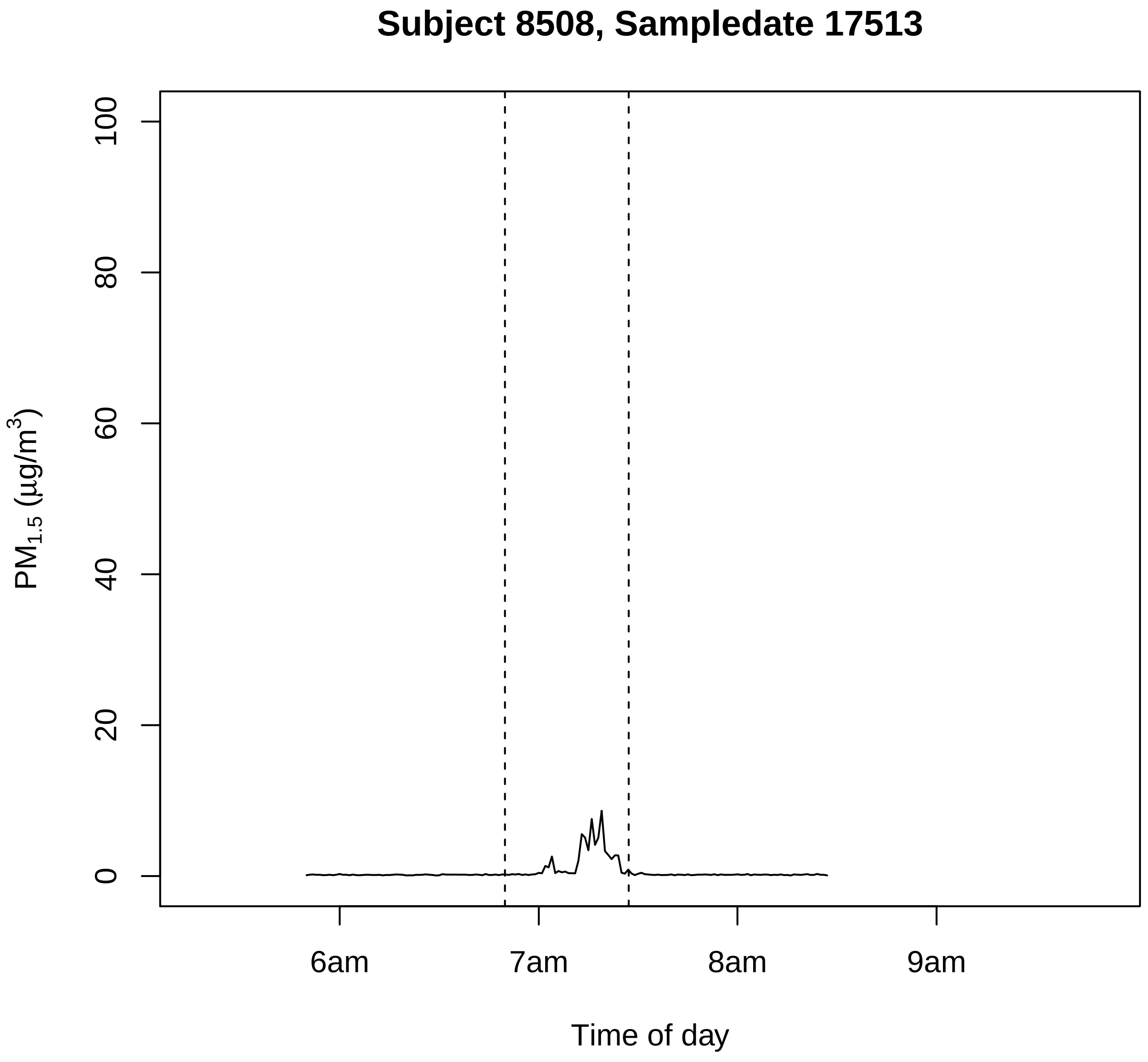

Subject 8508, Sampledate 17513
100
80
60
40
20
0
PM1.5 (µg/m3)
6am
7am
8am
9am
Time of day

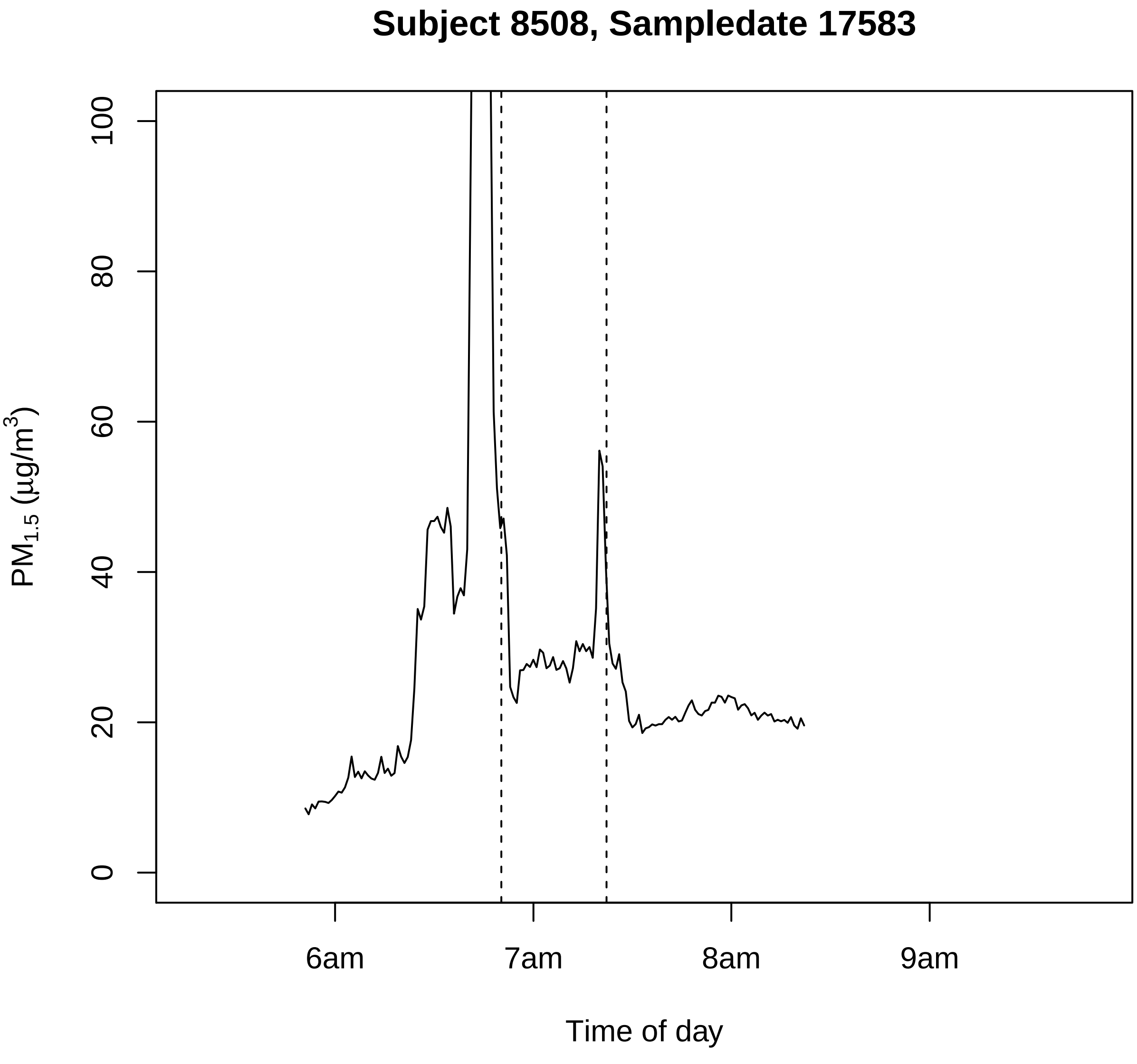

Subject 8508, Sampledate 17583
0
20
40
60
80
100
PM1.5 (µg/m3)
6am
7am
8am
9am
Time of day

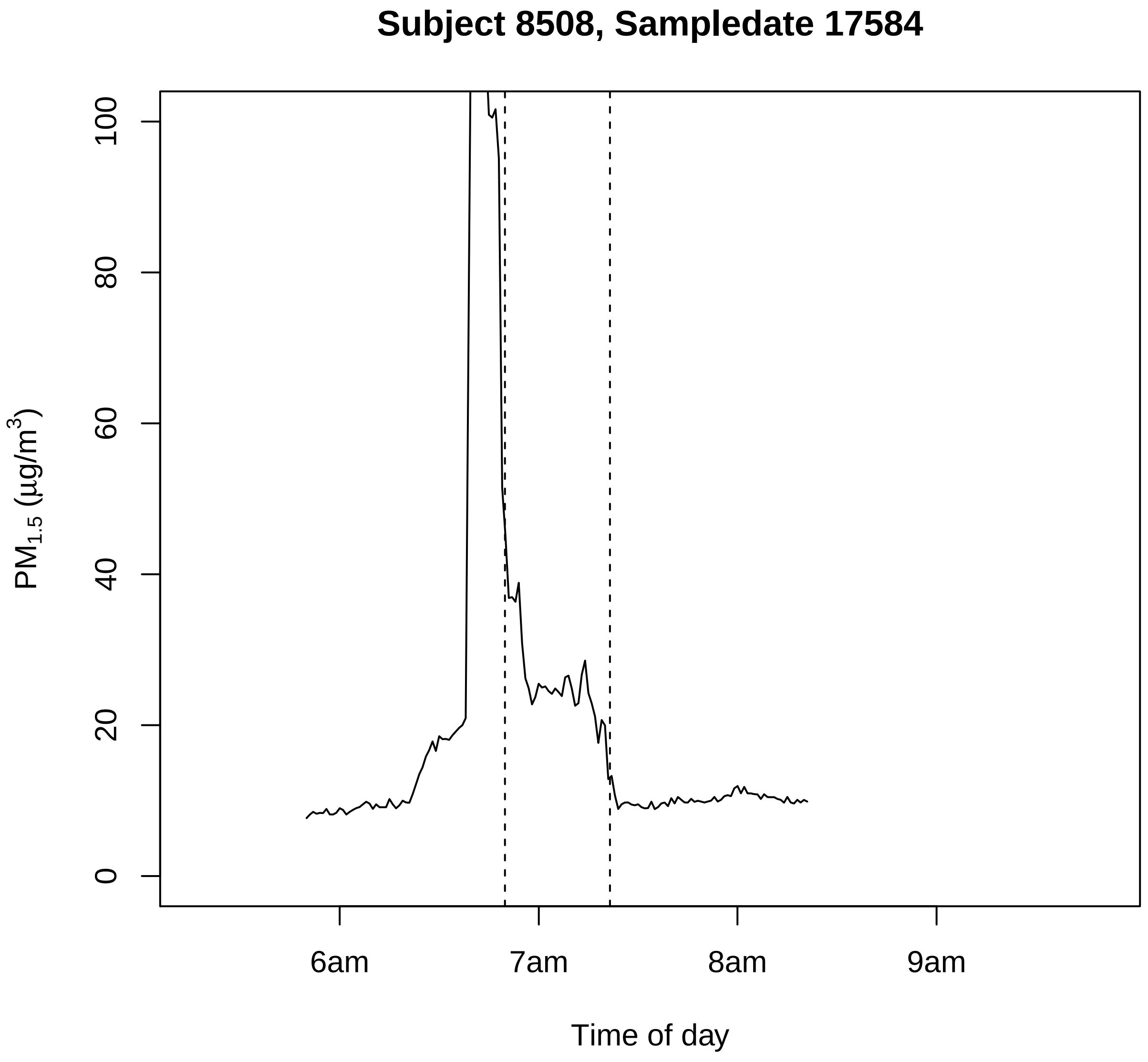

Subject 8508, Sampledate 17584
PM1.5 (µg/m3)
0
20
40
60
80
100
6am
7am
8am
9am
Time of day

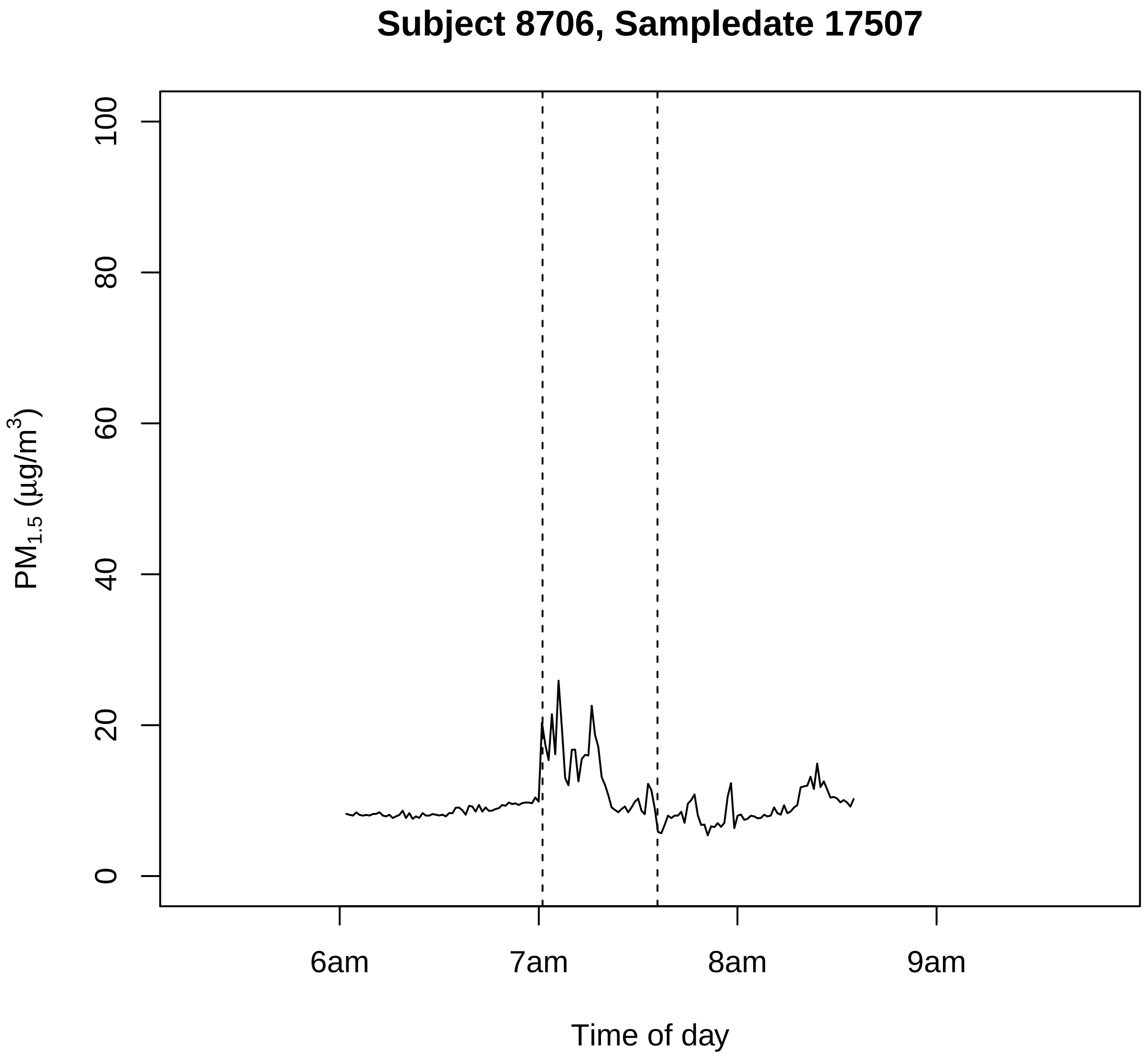

Subject 8706, Sampledate 17507
PM1.5 (µg/m3)
0
20
40
60
80
100
6am
7am
8am
9am
Time of day

Subject 8706, Sampledate 17583

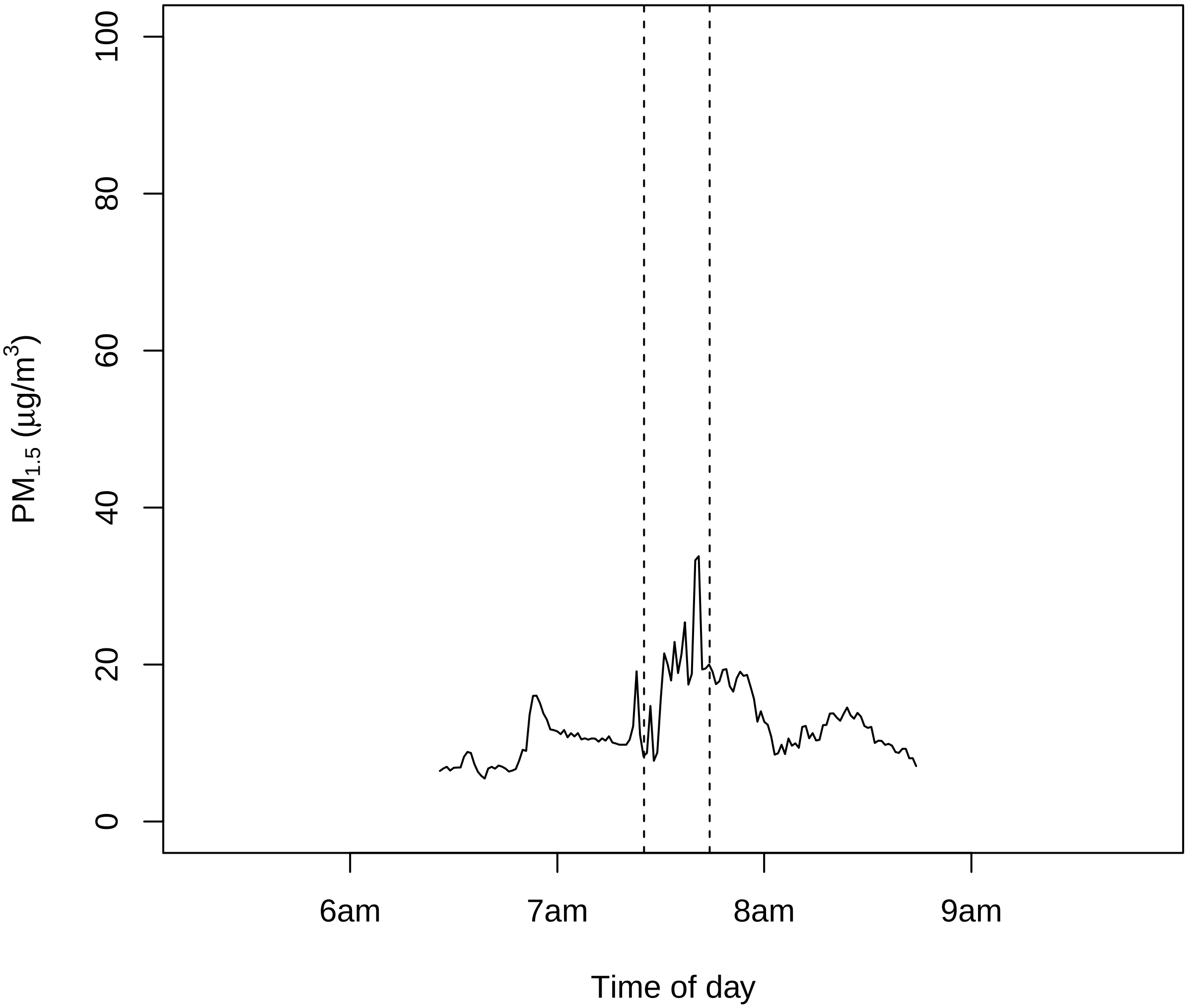

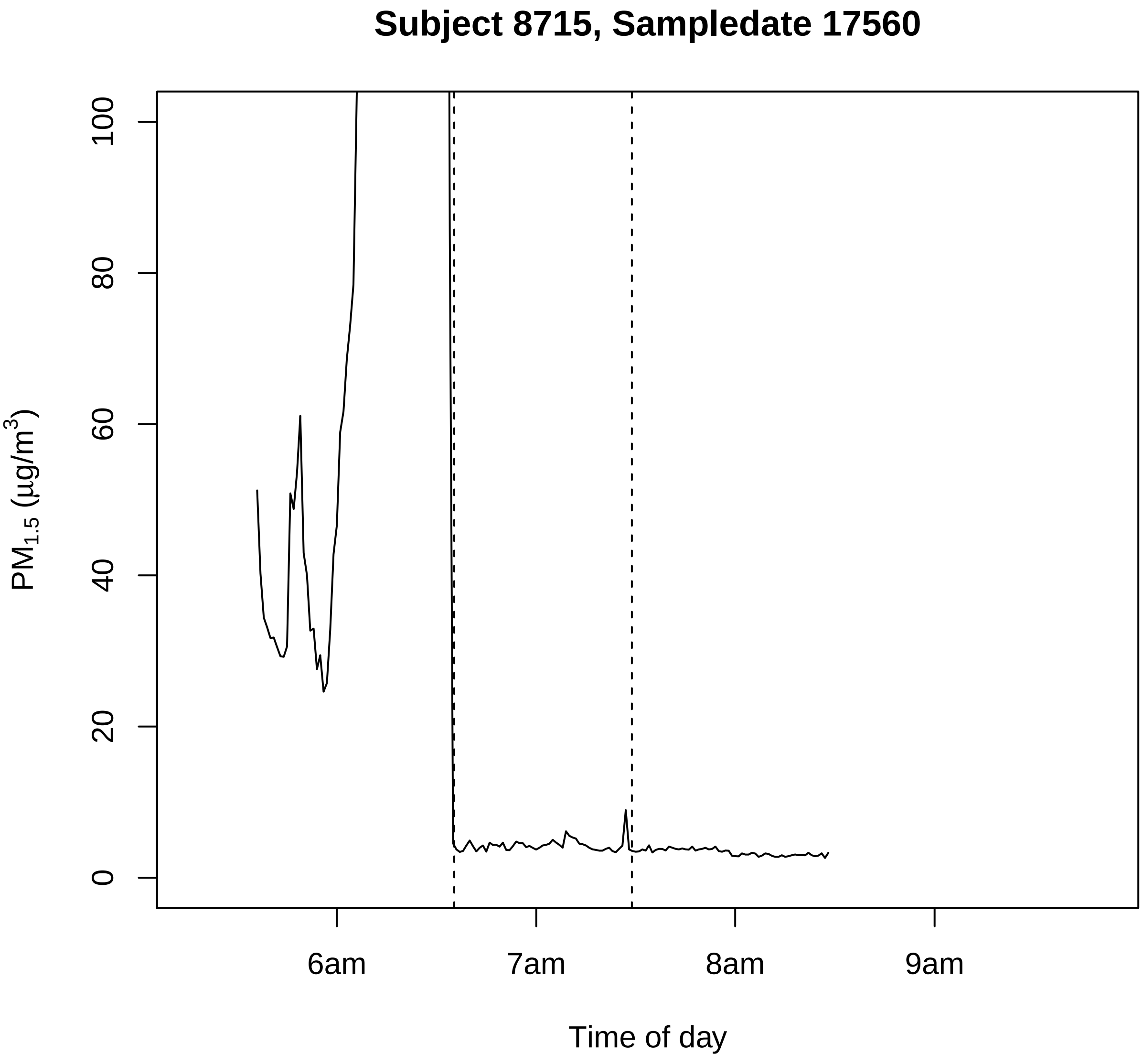

Subject 8715, Sampledate 17560
$PM_{1.5}$ (µg/m^3)
0
20
40
60
80
100
6am
7am
8am
9am
Time of day

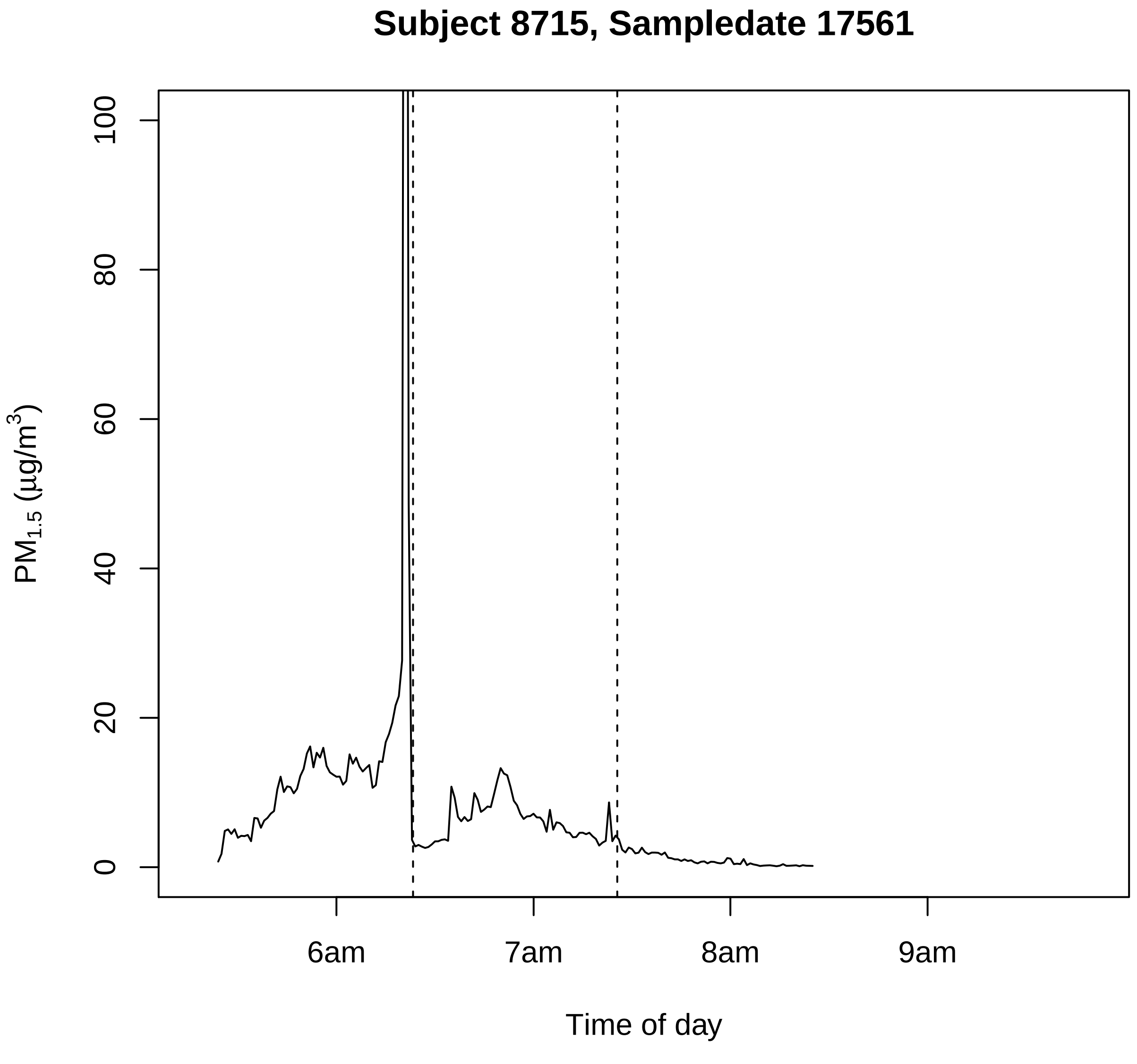

Subject 8715, Sampledate 17561
PM1.5 (µg/m3)
0
20
40
60
80
100
6am
7am
8am
9am
Time of day

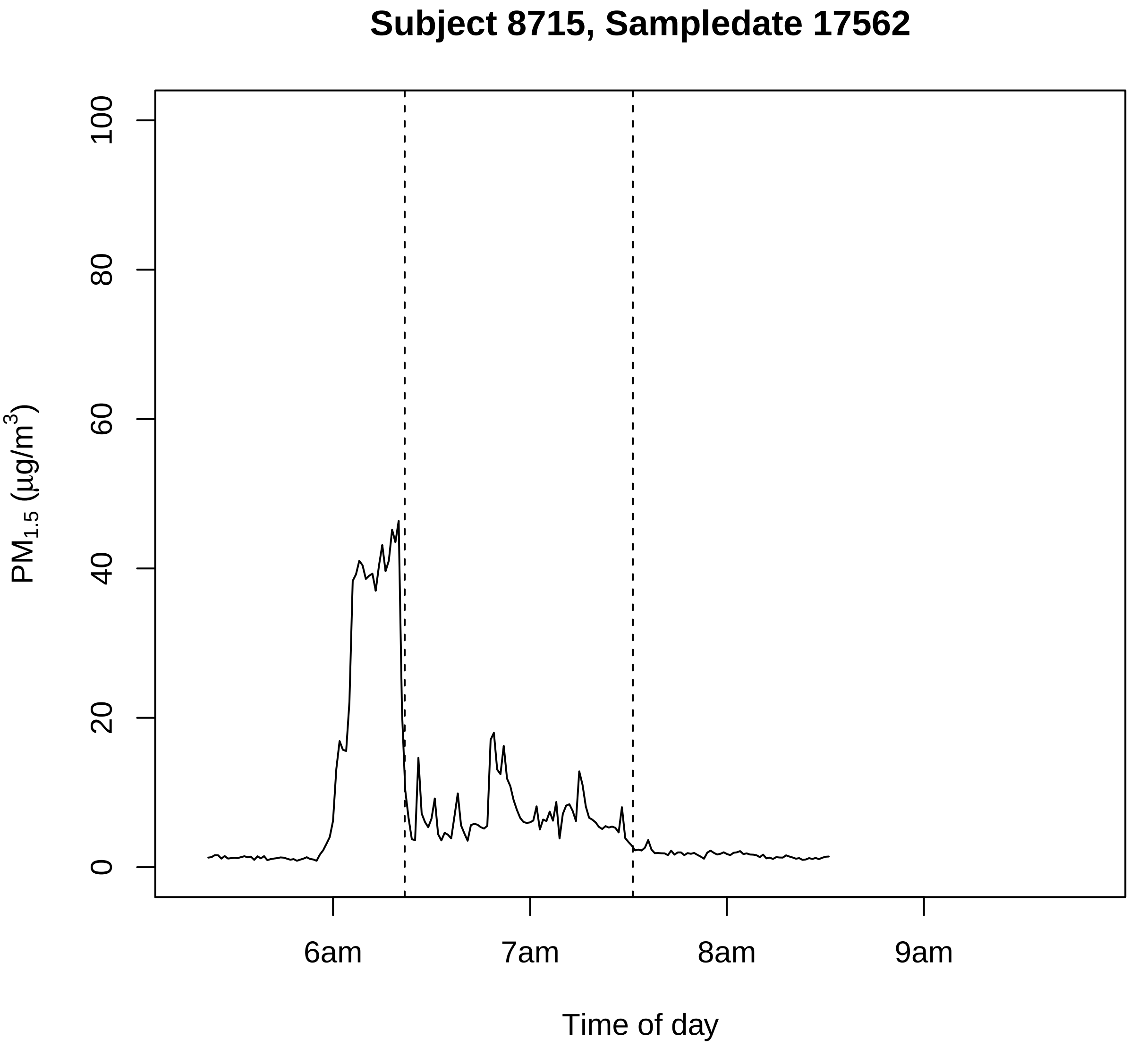

Subject 8715, Sampledate 17562
$PM_{1.5}$ (µg/m^3)
0
20
40
60
80
100
6am
7am
8am
9am
Time of day

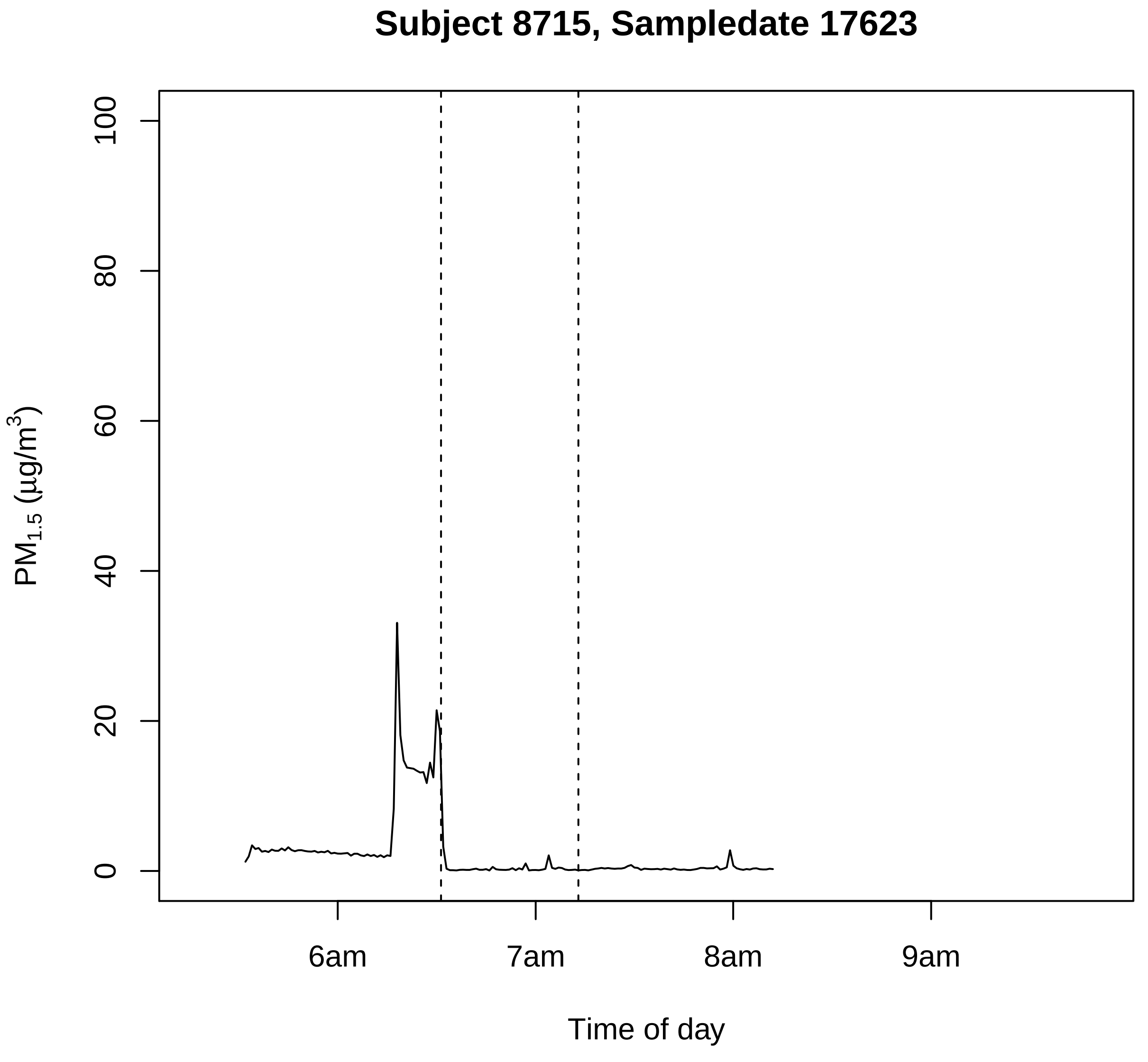

Subject 8715, Sampledate 17623
PM1.5 (µg/m3)
0
20
40
60
80
100
6am
7am
8am
9am
Time of day

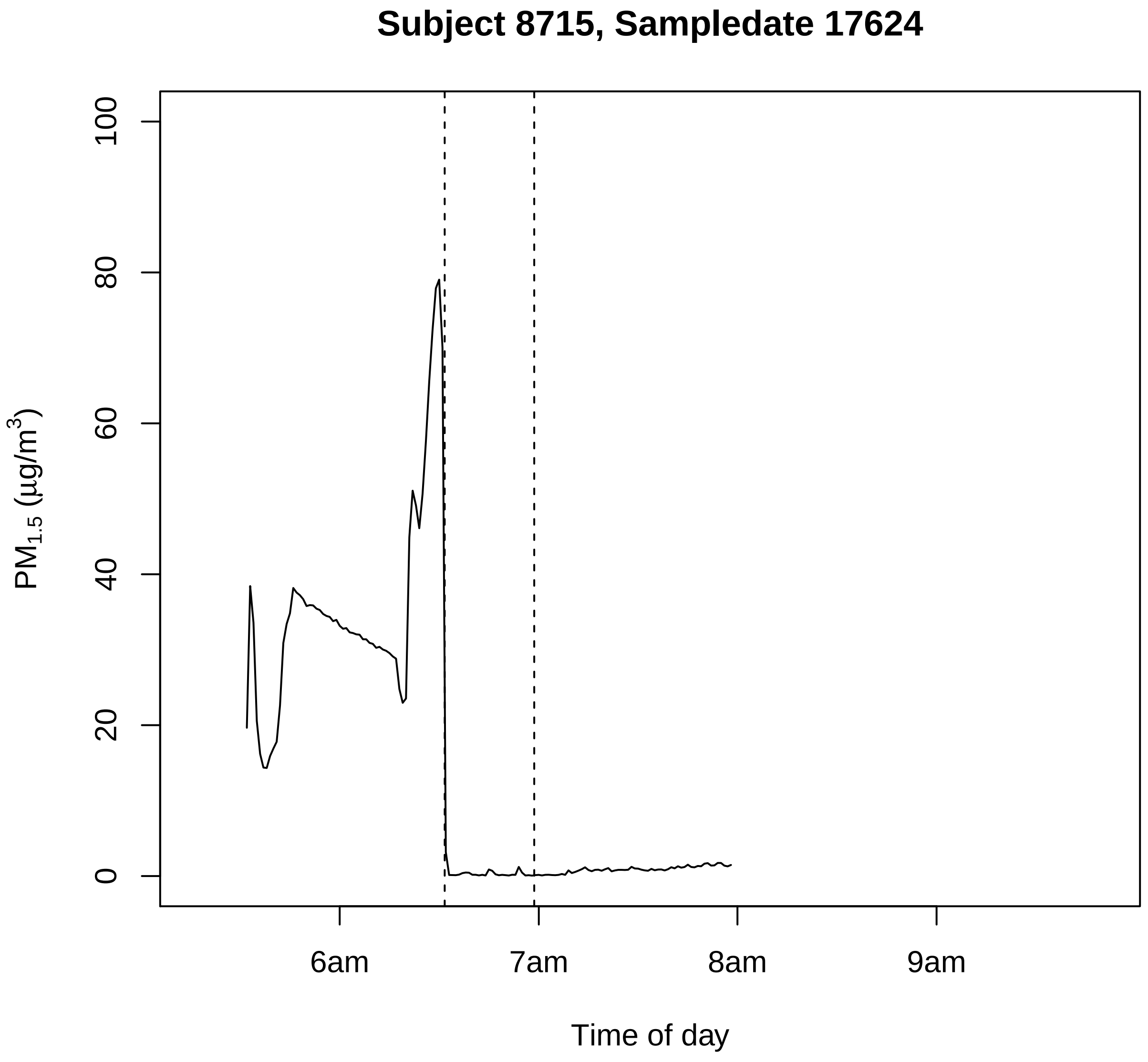
Subject 8715, Sampledate 17624
PM1.5 (µg/m3)
0
20
40
60
80
100
6am
7am
8am
9am
Time of day

Subject 8715, Sampledate 17626

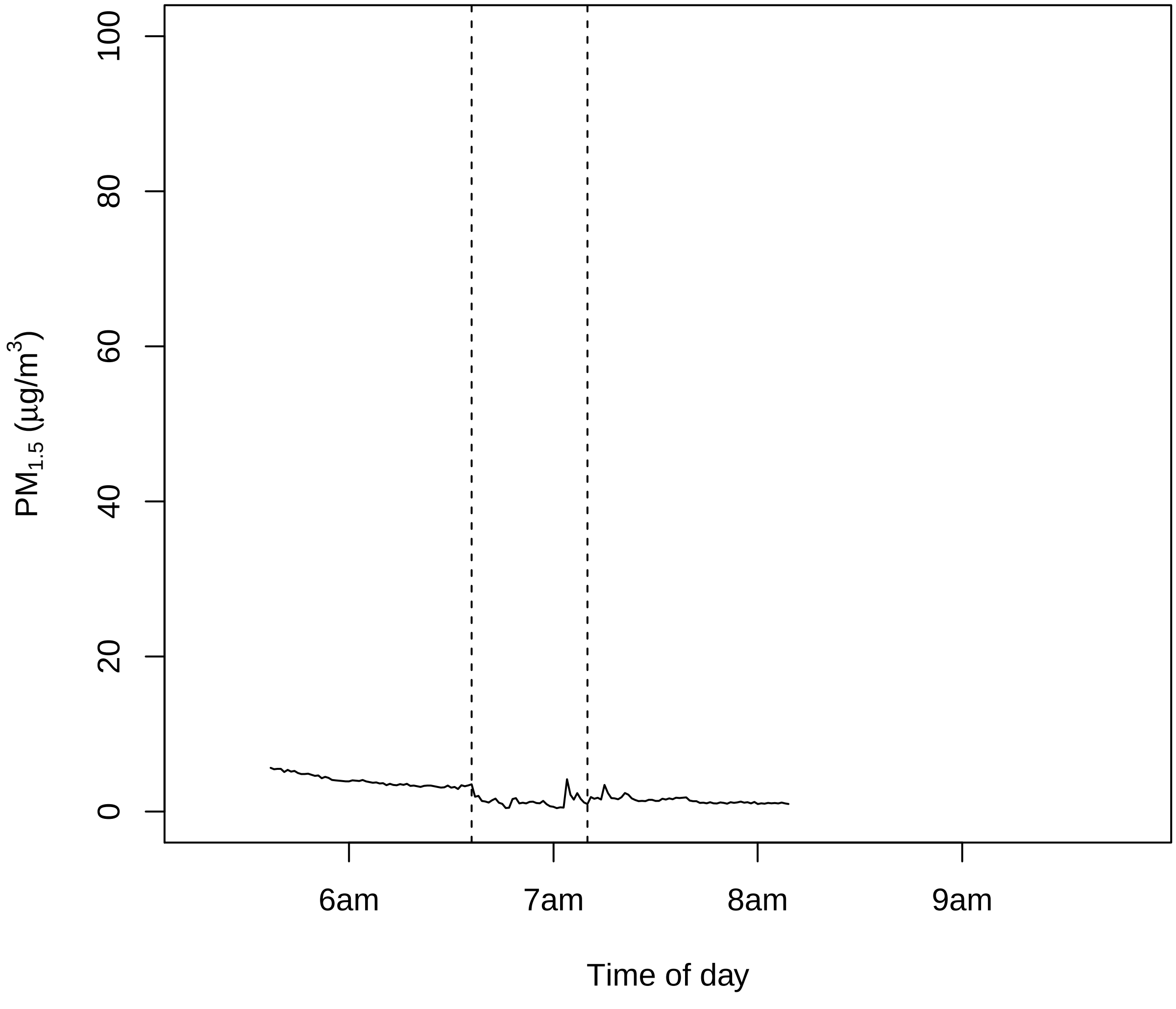

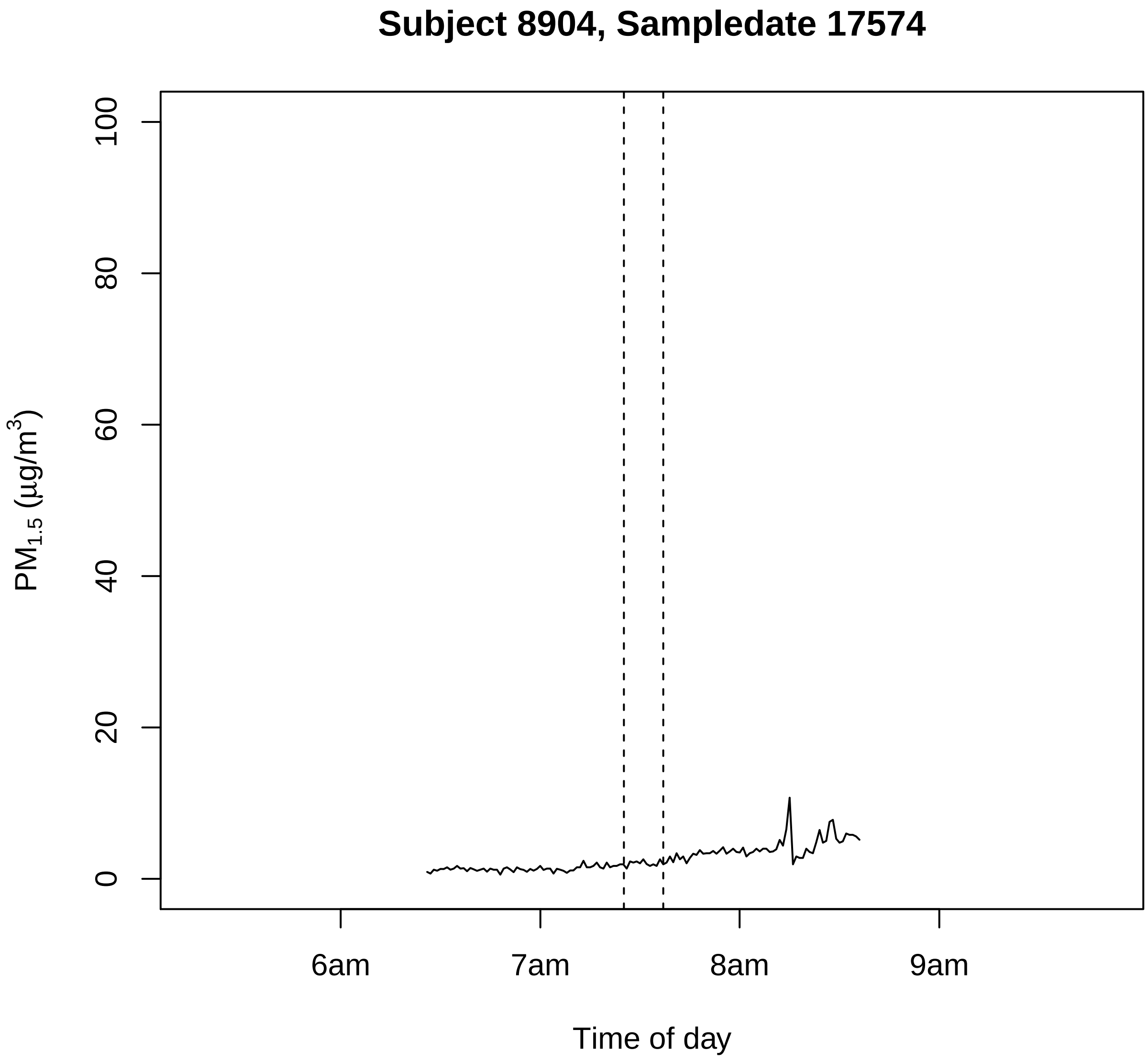

Subject 8904, Sampledate 17574
PM1.5 (µg/m3)
0
20
40
60
80
100
6am
7am
8am
9am
Time of day

Subject 8904, Sampledate 17575

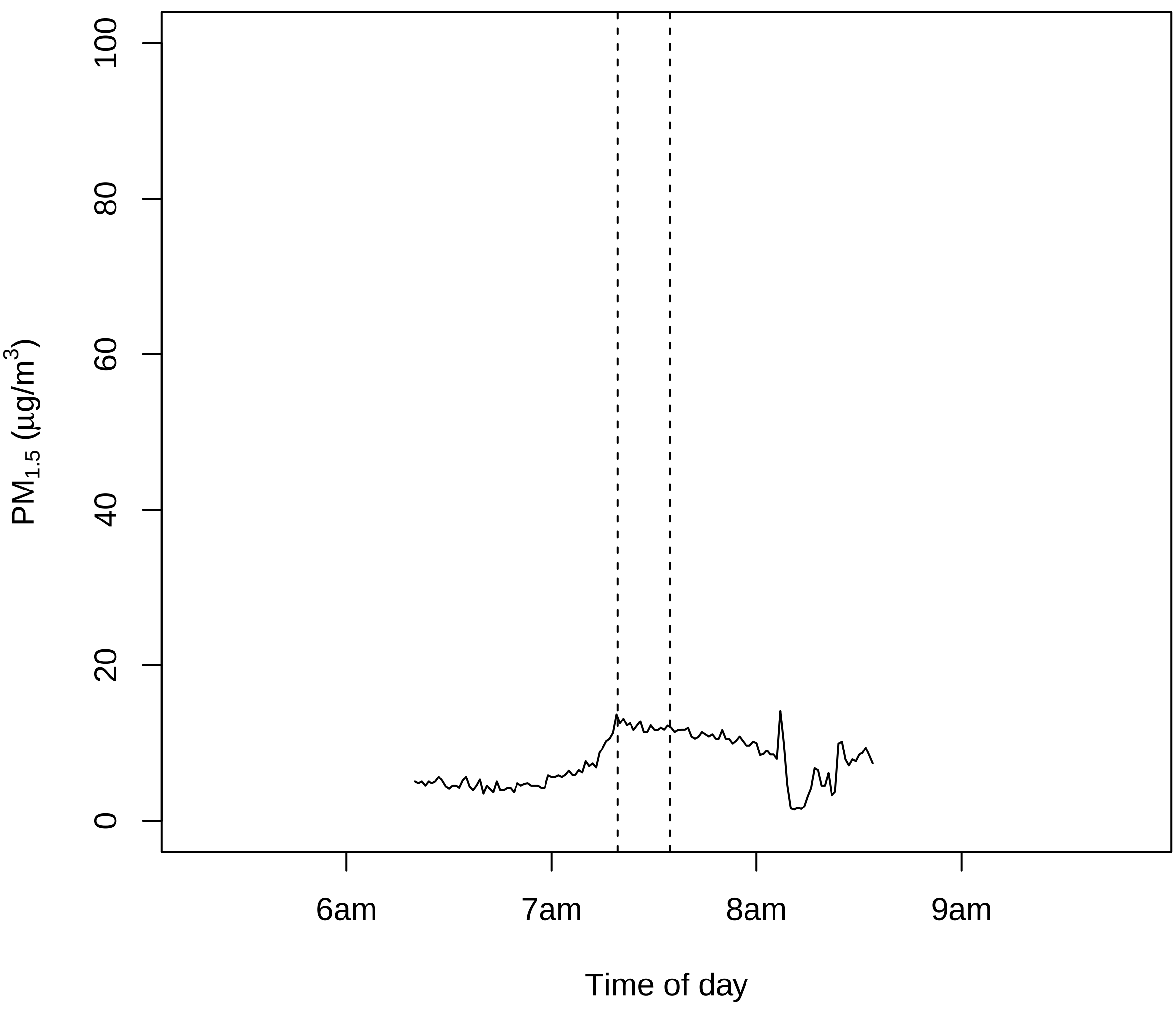

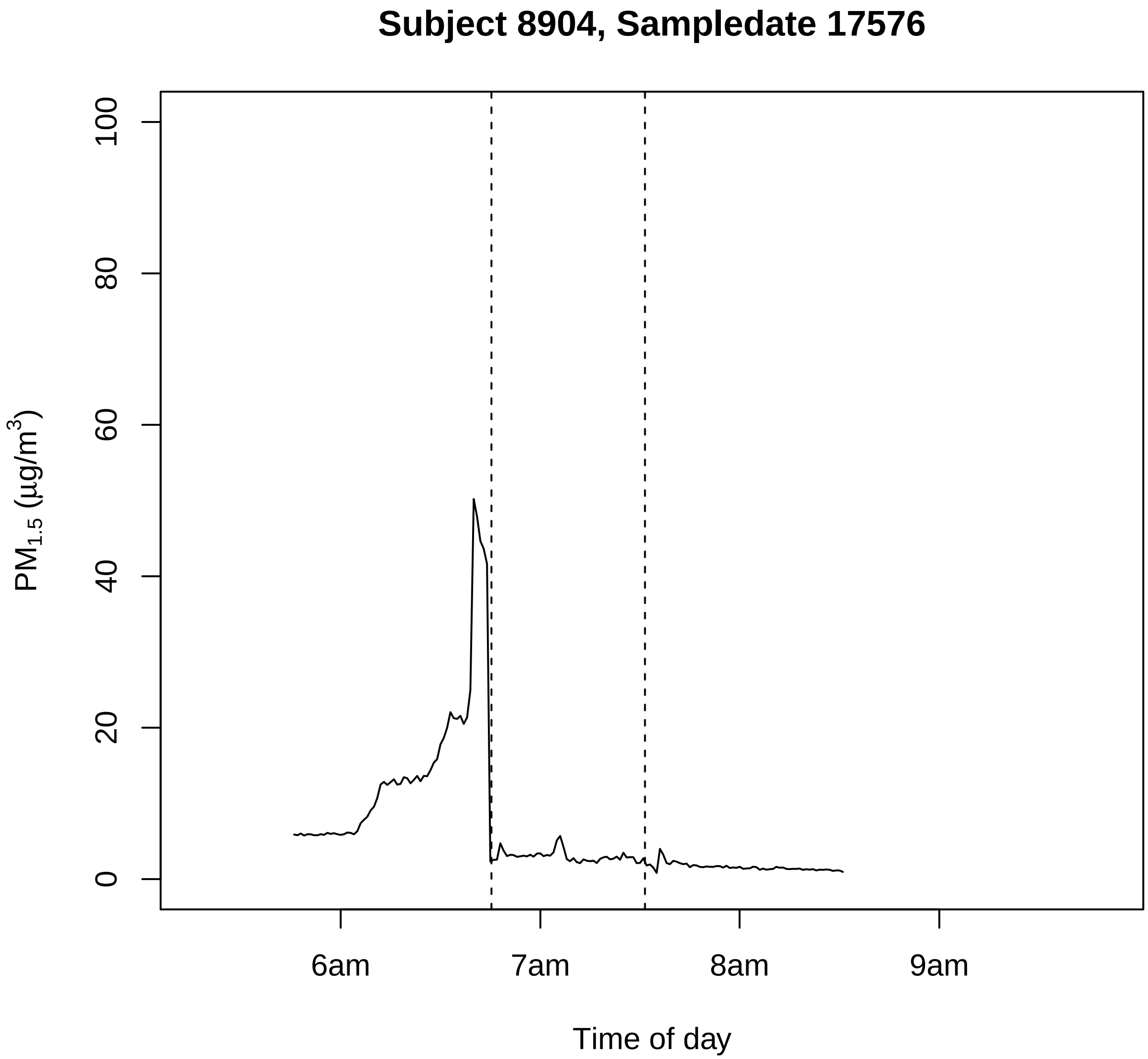

Subject 8904, Sampledate 17576
$PM_{1.5}$ (µg/m^3)
0
20
40
60
80
100
6am
7am
8am
9am
Time of day

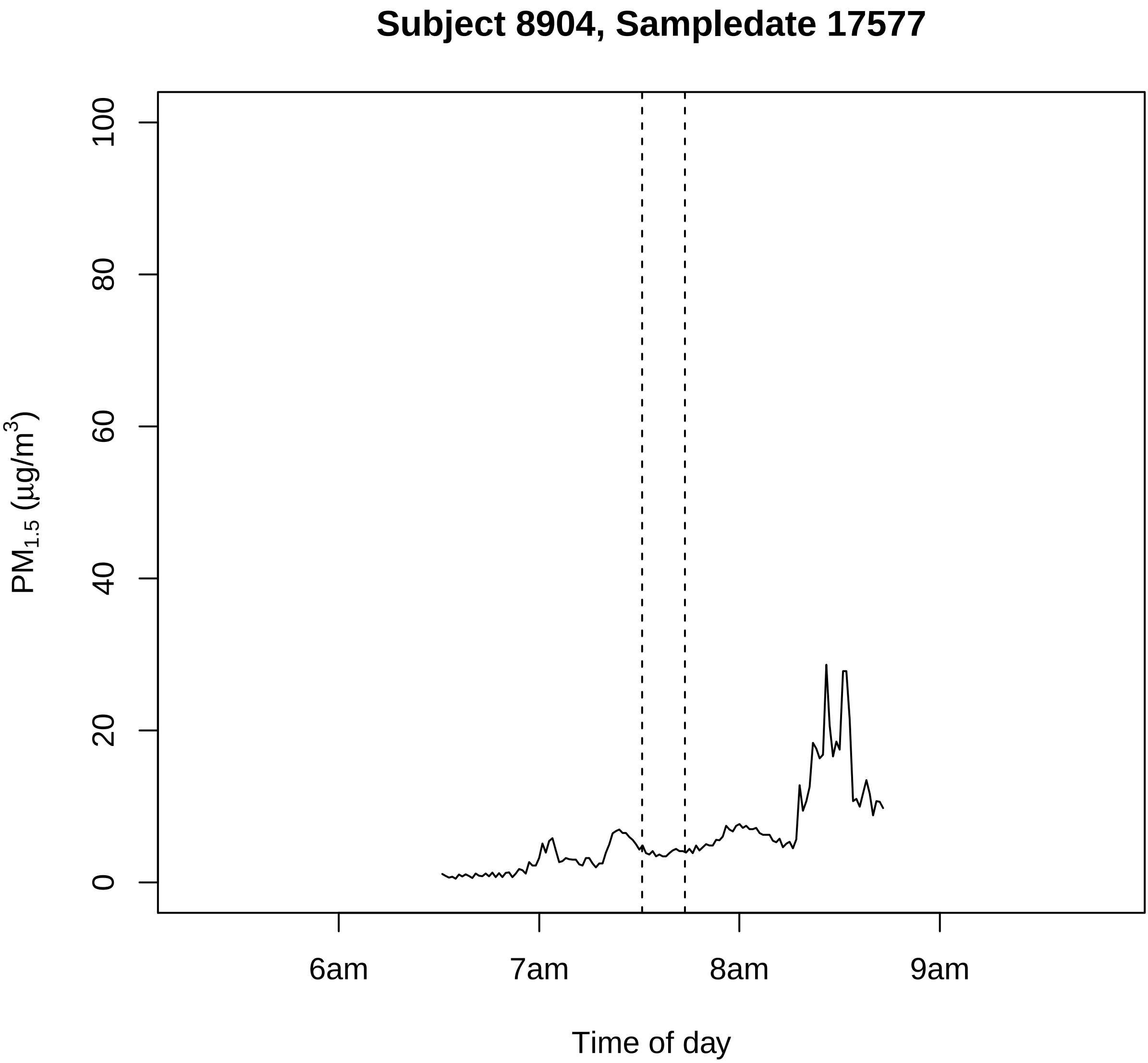
Subject 8904, Sampledate 17577
$PM_{1.5}$ (µg/m³)
0
20
40
60
80
100
6am
7am
8am
9am
Time of day

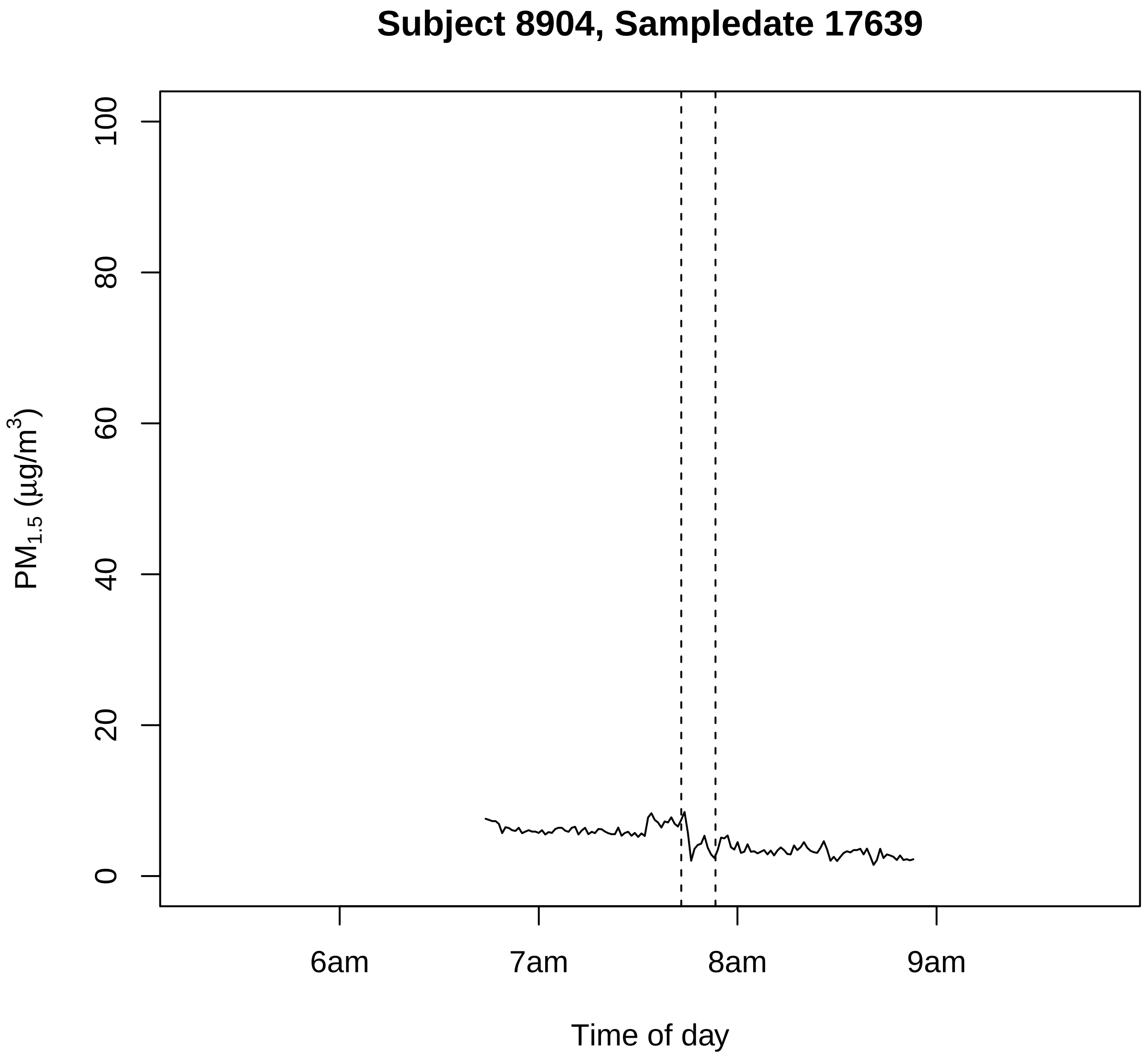

Subject 8904, Sampledate 17639
PM1.5 (µg/m3)
100
80
60
40
20
0
6am
7am
8am
9am
Time of day

Subject 8904, Sampledate 17640

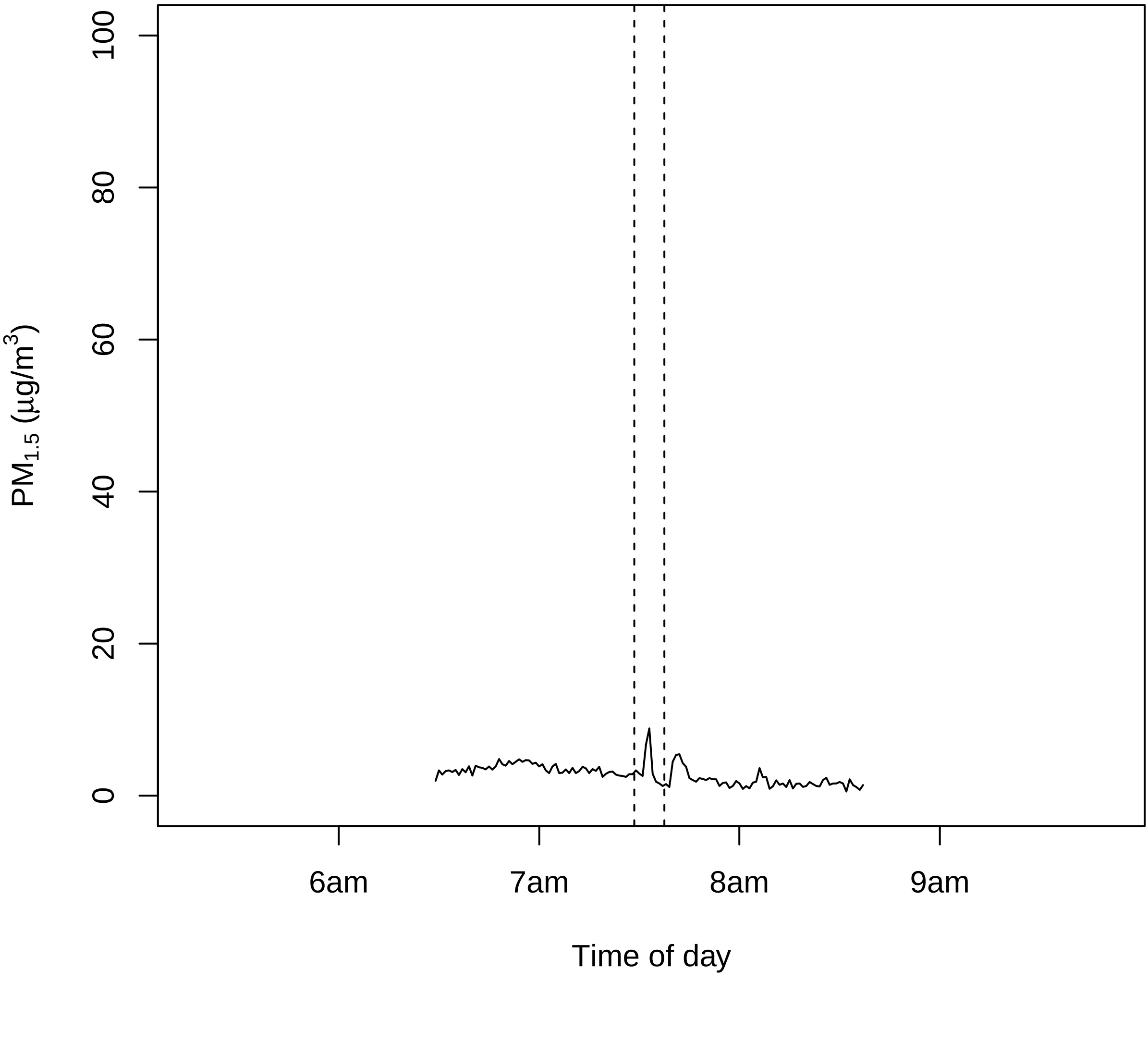

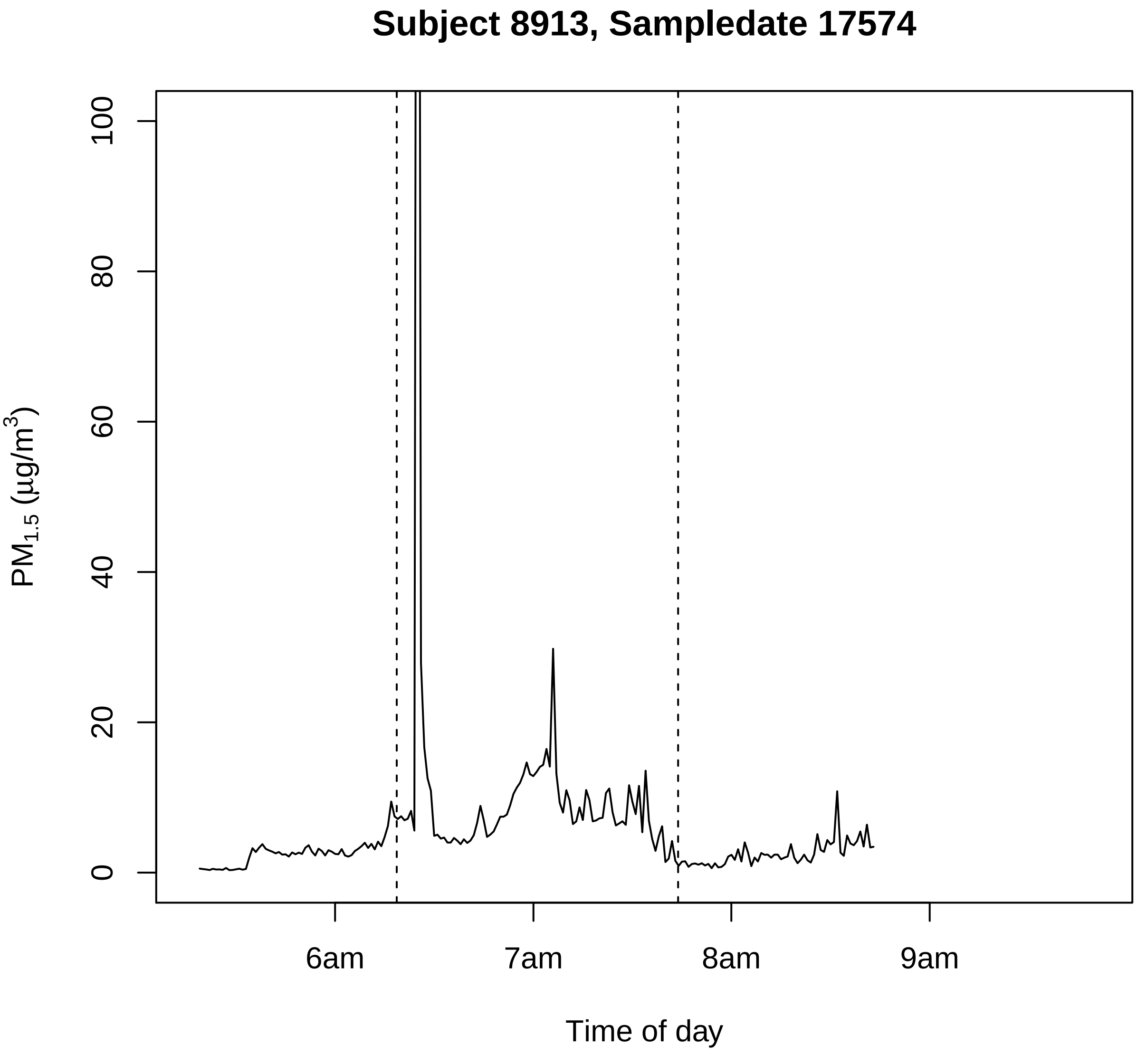

Subject 8913, Sampledate 17574
0
20
40
60
80
100
$PM_{1.5}$ (µg/m^3)
6am
7am
8am
9am
Time of day

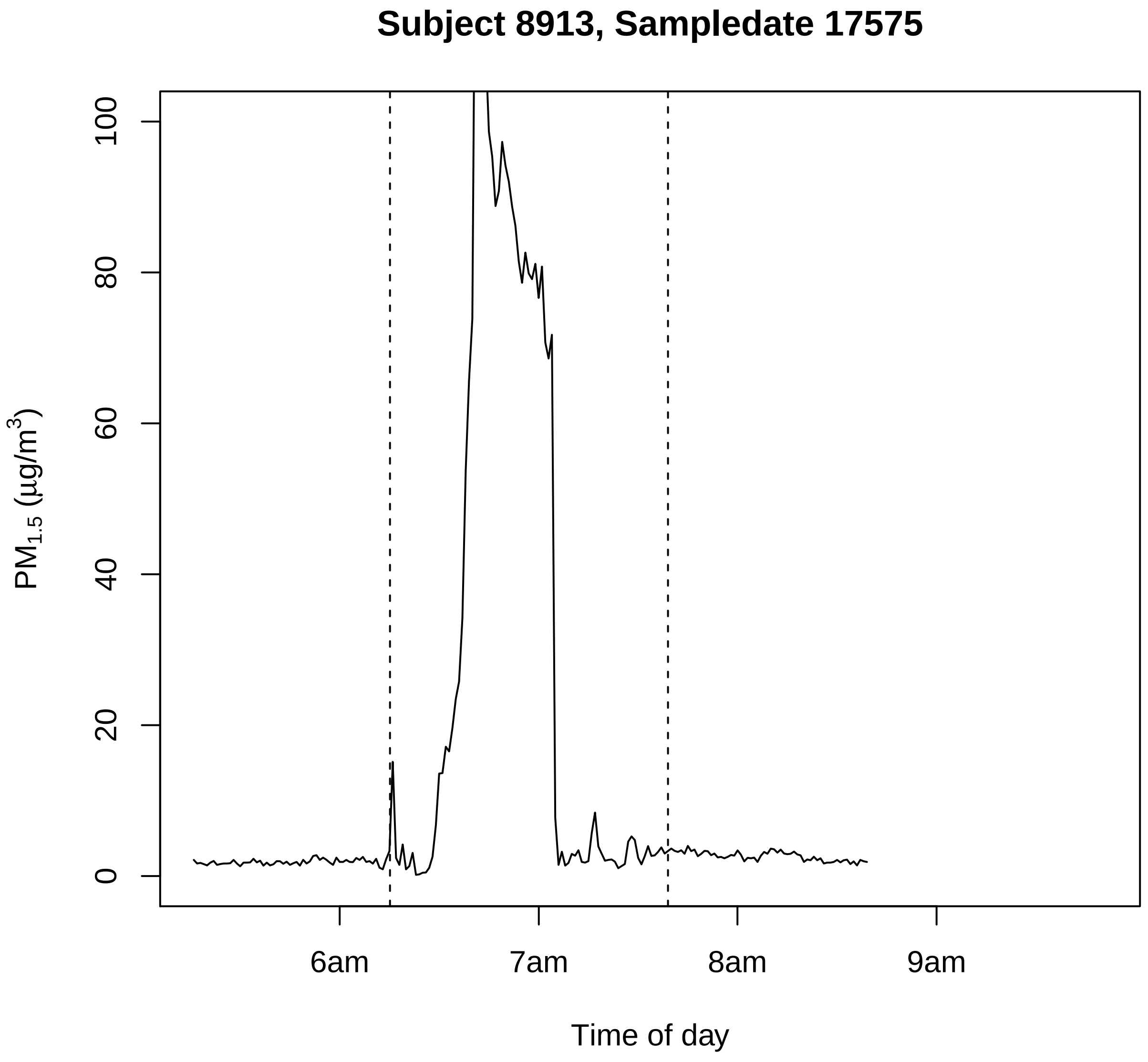
Subject 8913, Sampledate 17575
$PM_{1.5}$ (µg/m^3)
0
20
40
60
80
100
6am
7am
8am
9am
Time of day

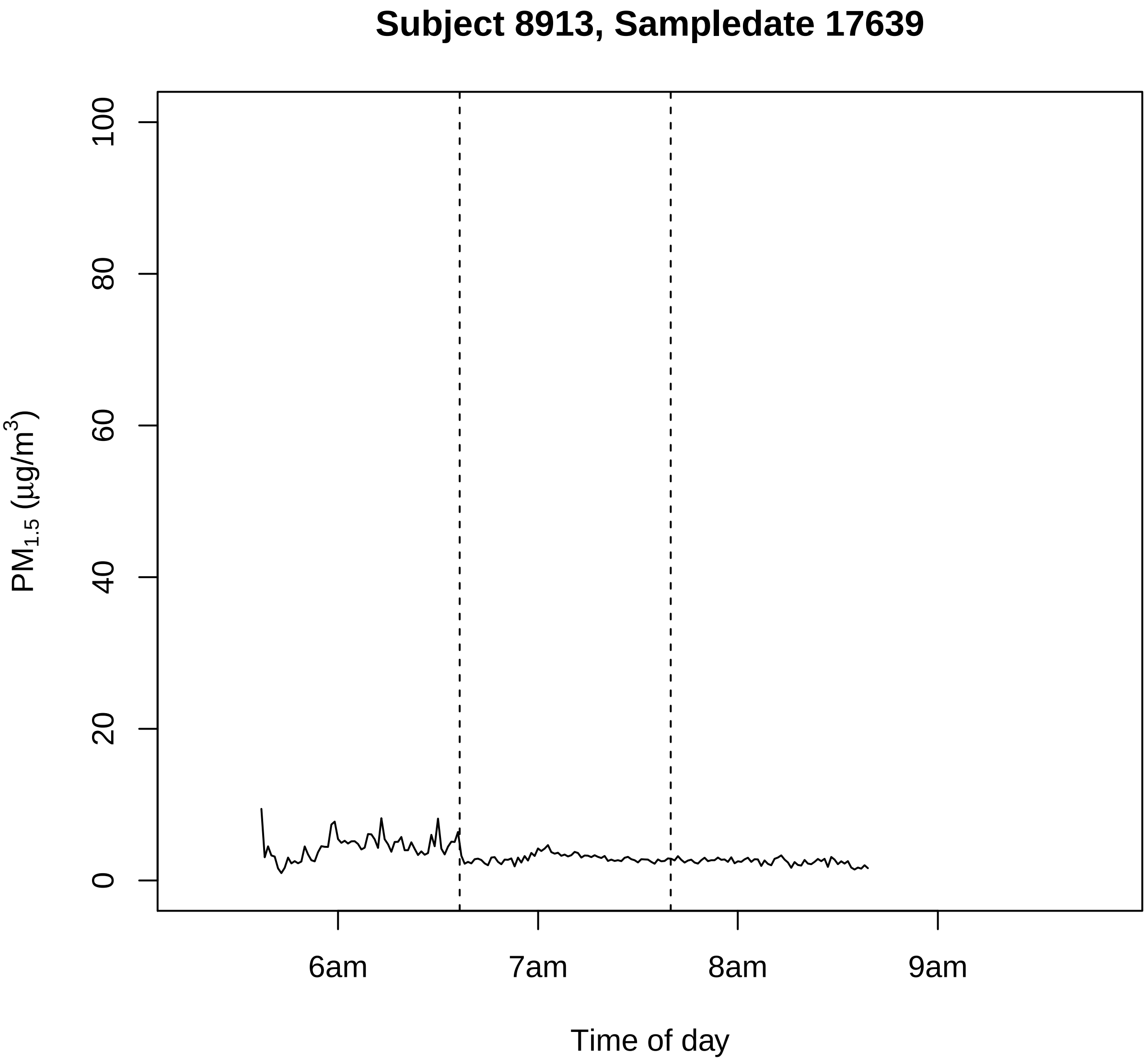

Subject 8913, Sampledate 17639
$PM_{1.5}$ (µg/m^3)
0
20
40
60
80
100
6am
7am
8am
9am
Time of day

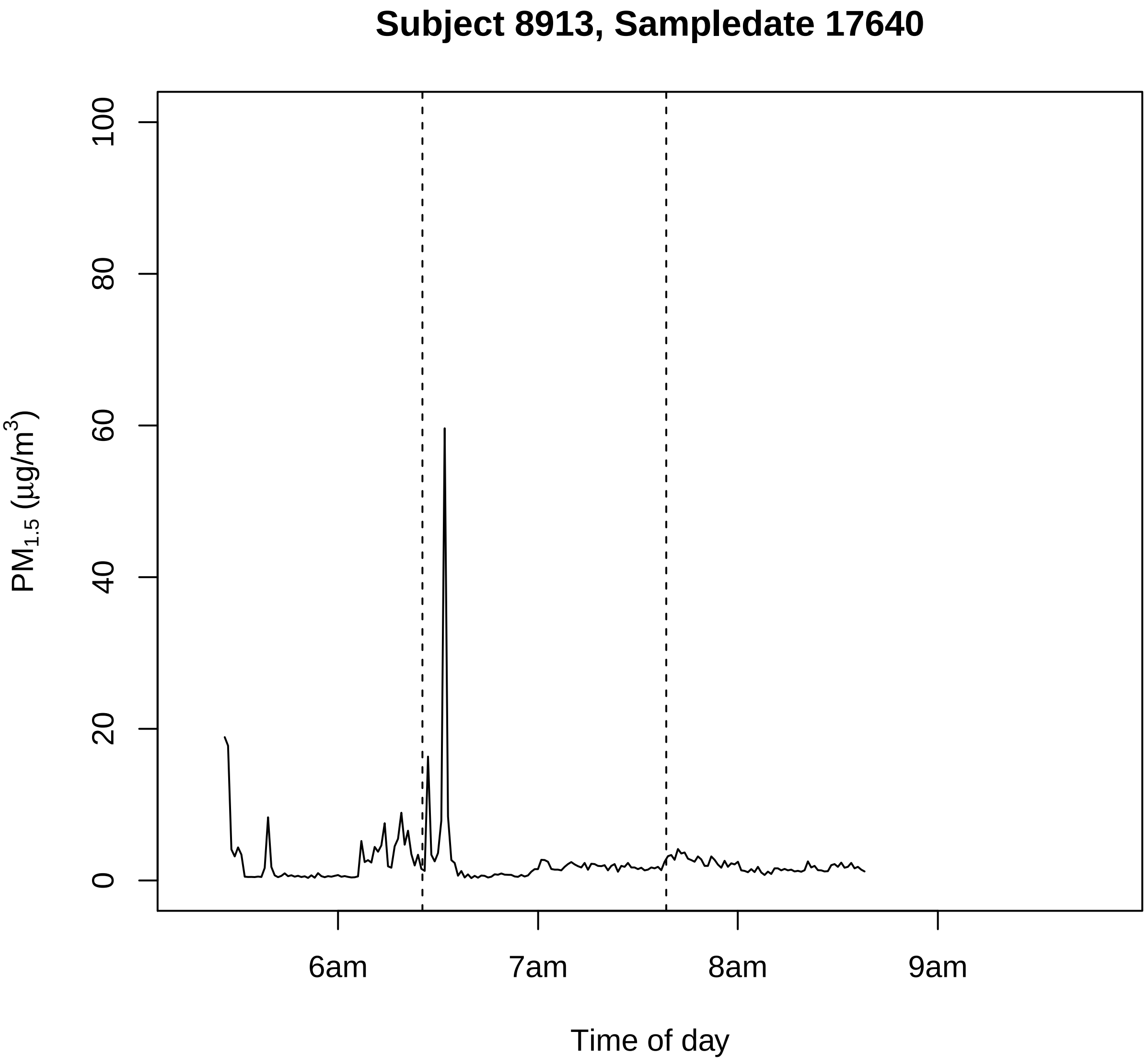

Subject 8913, Sampledate 17640
$PM_{1.5}$ (μg/m^3)
0
20
40
60
80
100
6am
7am
8am
9am
Time of day

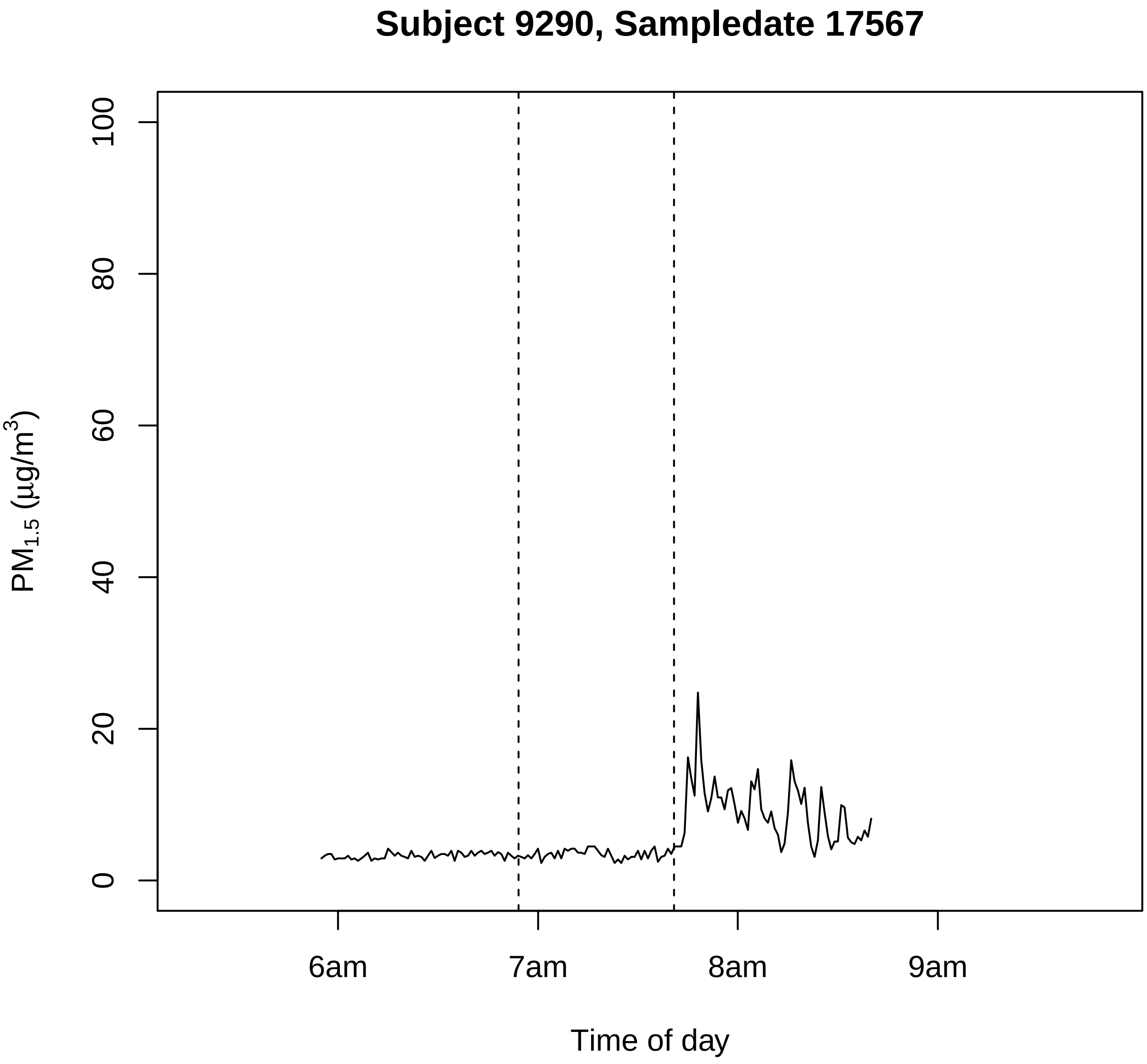
Subject 9290, Sampledate 17567
PM1.5 (µg/m3)
0
20
40
60
80
100
6am
7am
8am
9am
Time of day

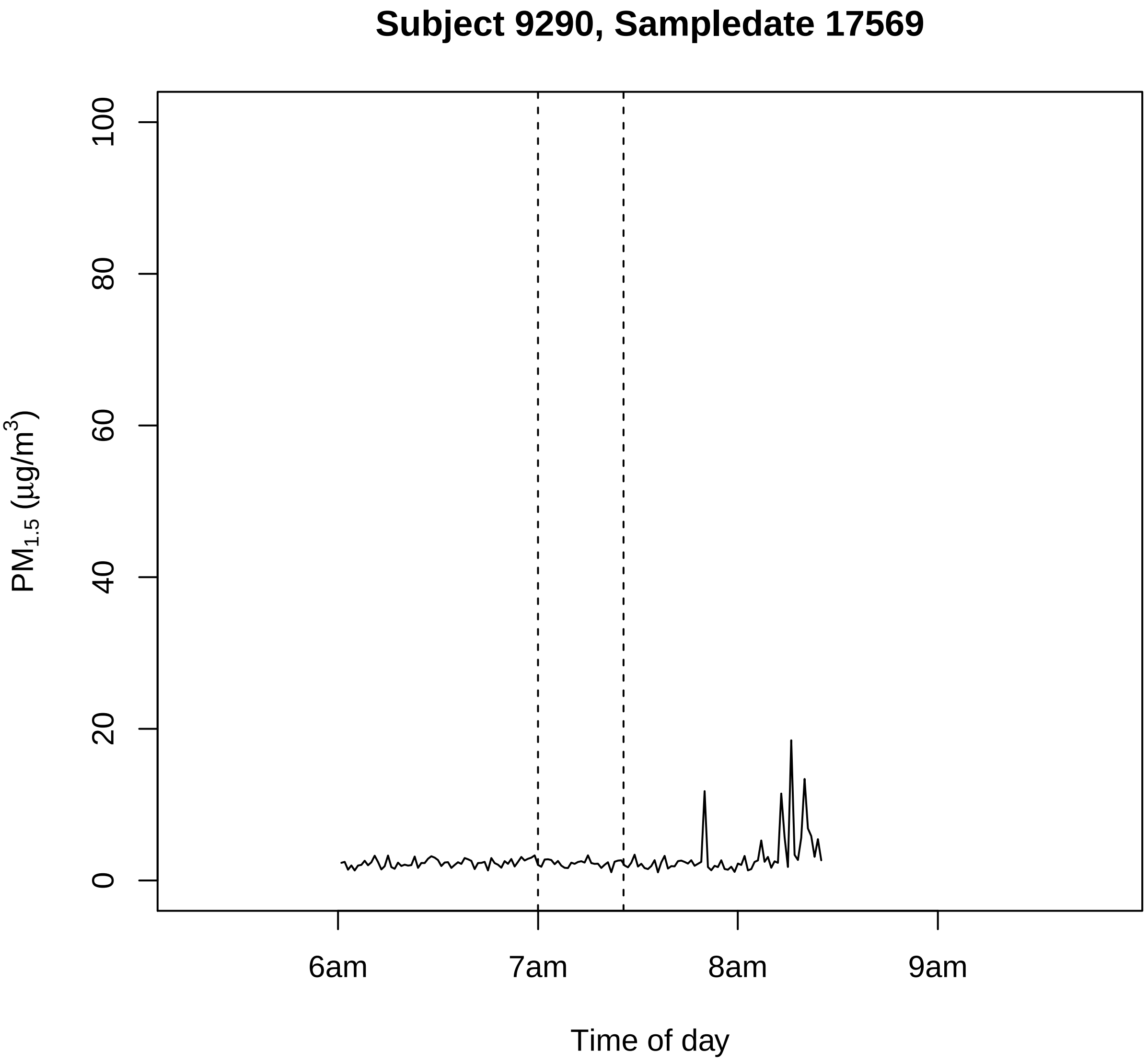

Subject 9290, Sampledate 17569
PM1.5 (µg/m3)
0
20
40
60
80
100
6am
7am
8am
9am
Time of day

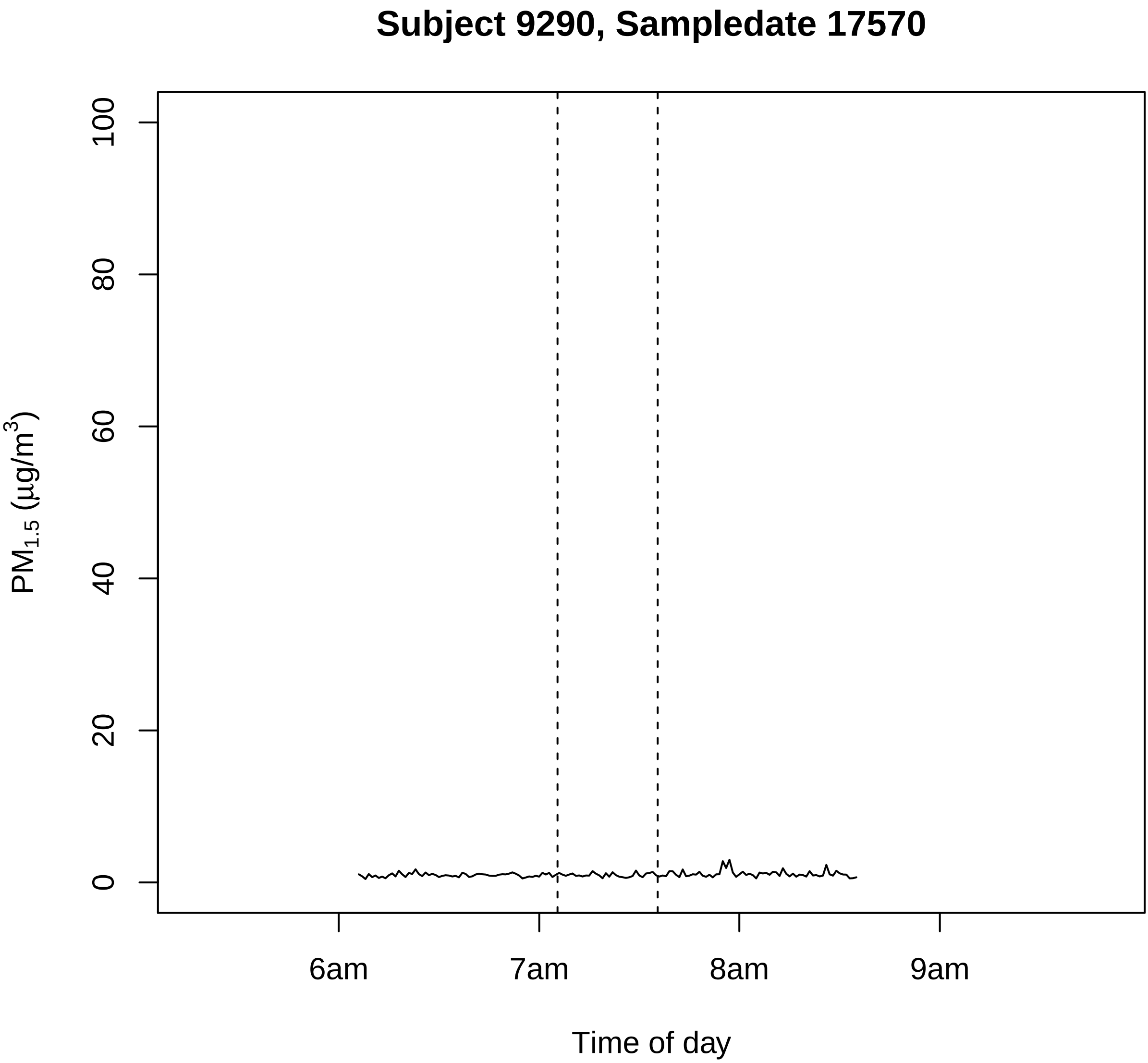

Subject 9290, Sampledate 17570
PM1.5 (µg/m3)
0
20
40
60
80
100
6am
7am
8am
9am
Time of day

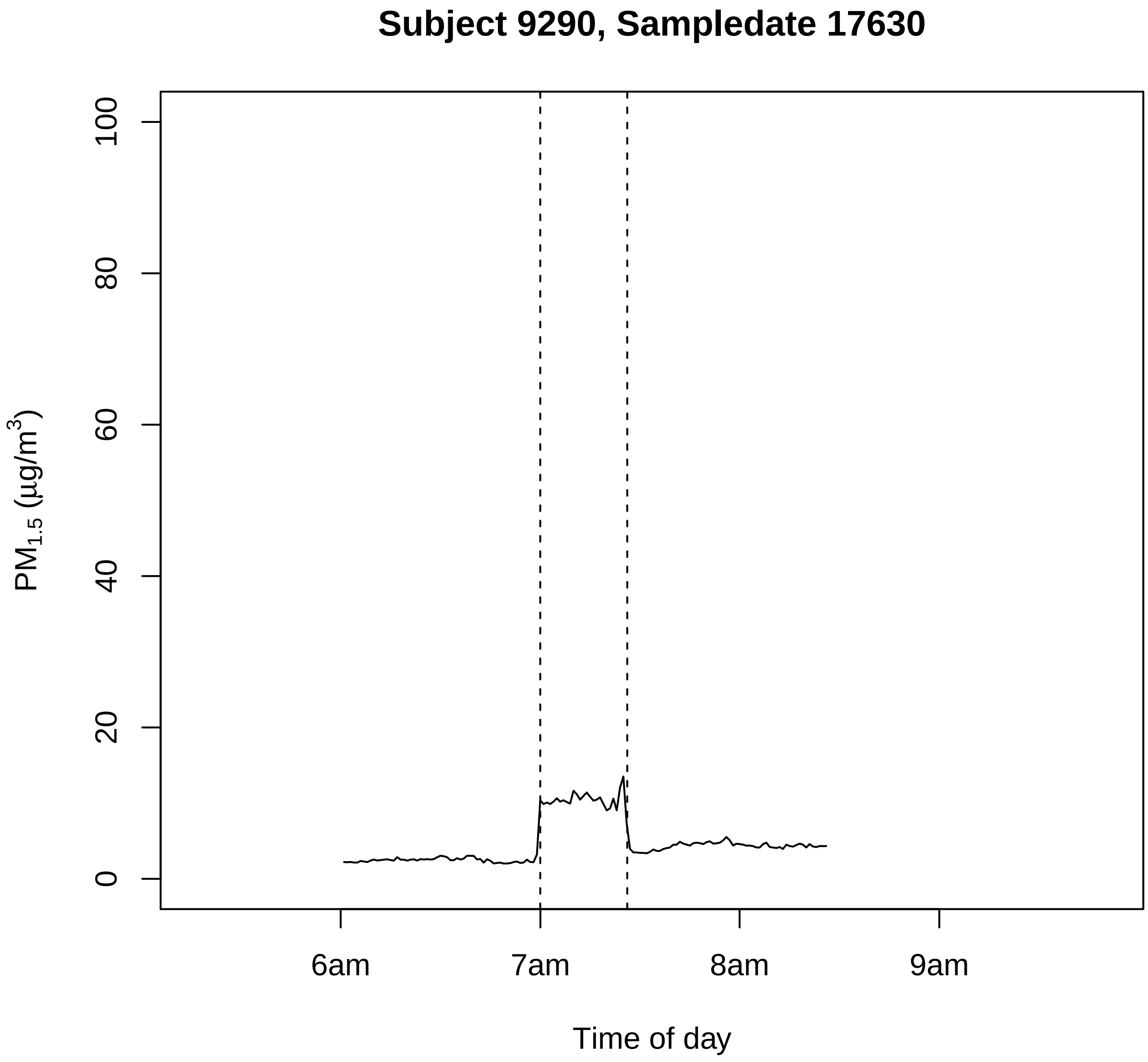
Subject 9290, Sampledate 17630
$PM_{1.5}$ (µg/m^3)
0
20
40
60
80
100
6am
7am
8am
9am
Time of day

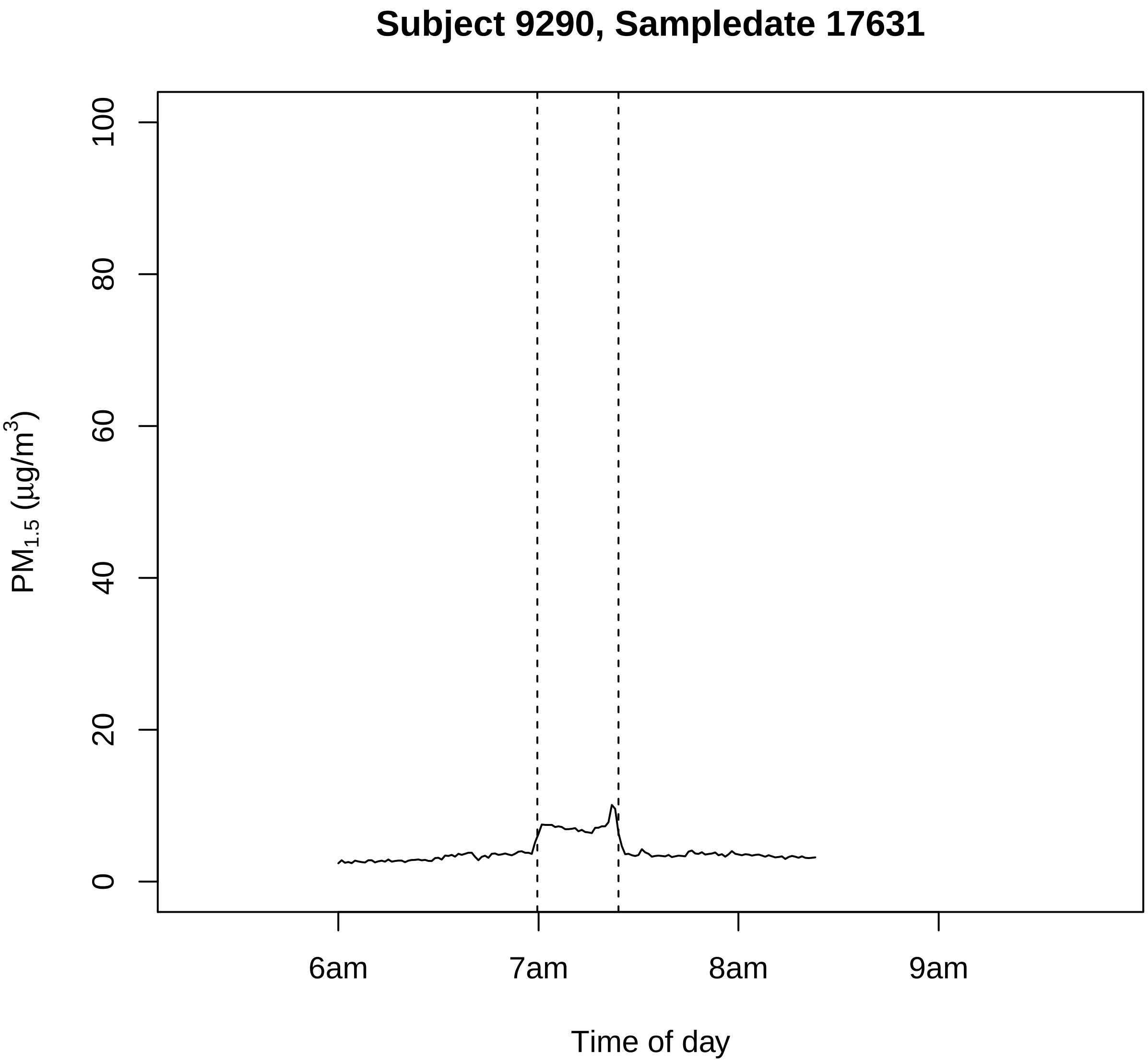

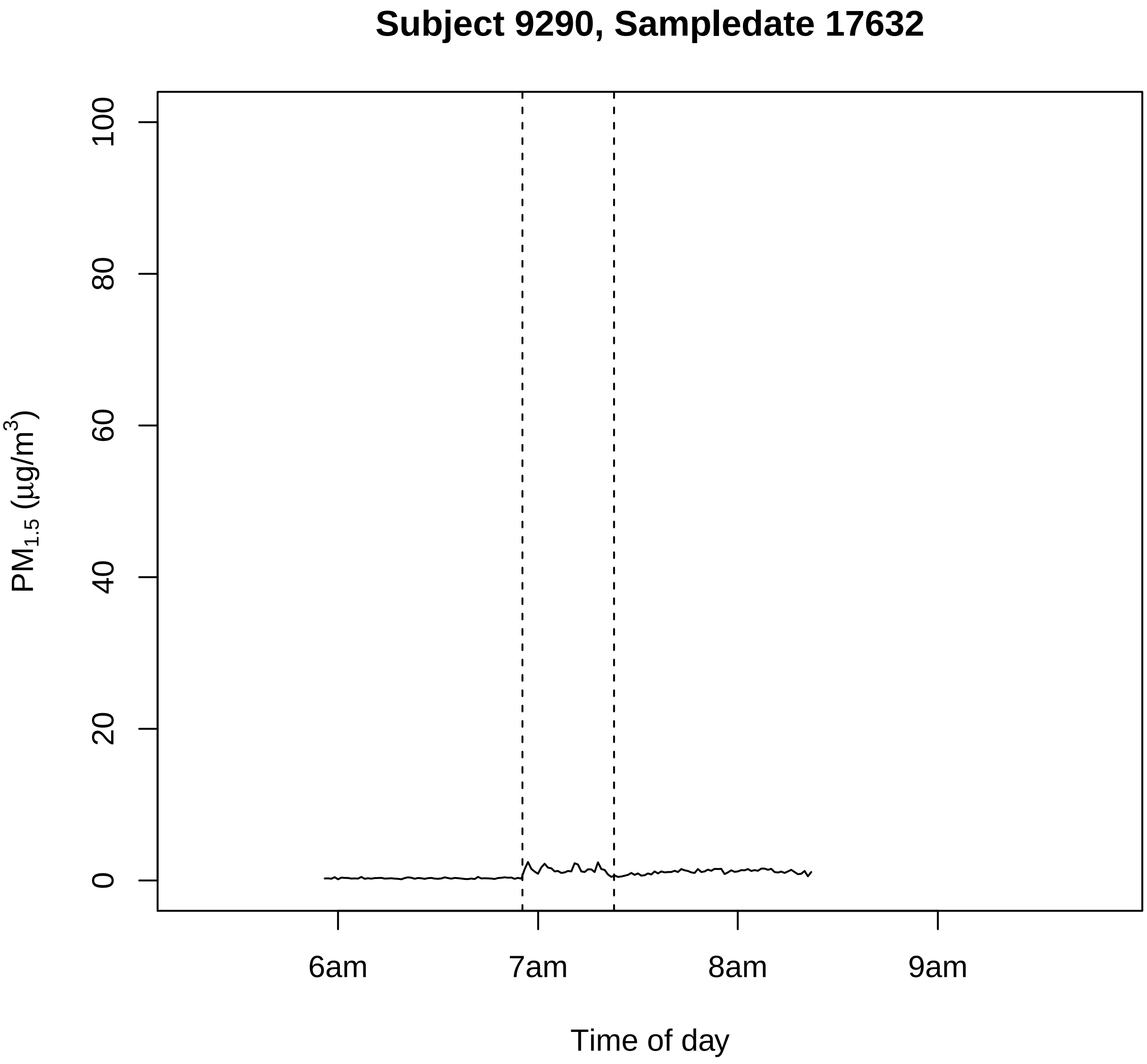

Subject 9290, Sampledate 17632
PM1.5 (µg/m3)
0
20
40
60
80
100
6am
7am
8am
9am
Time of day

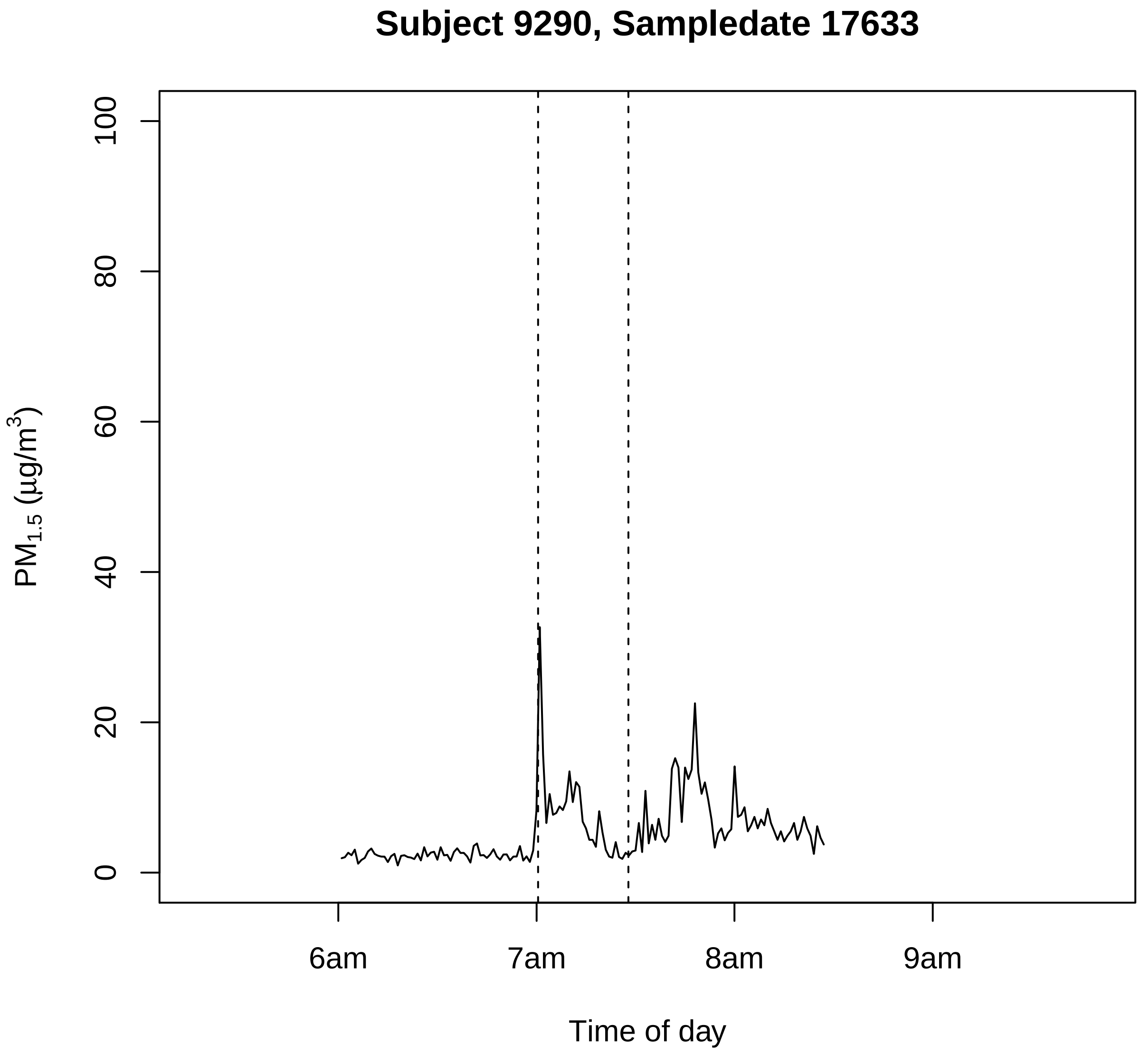

Subject 9290, Sampledate 17633
PM1.5 (µg/m3)
0
20
40
60
80
100
6am
7am
8am
9am
Time of day

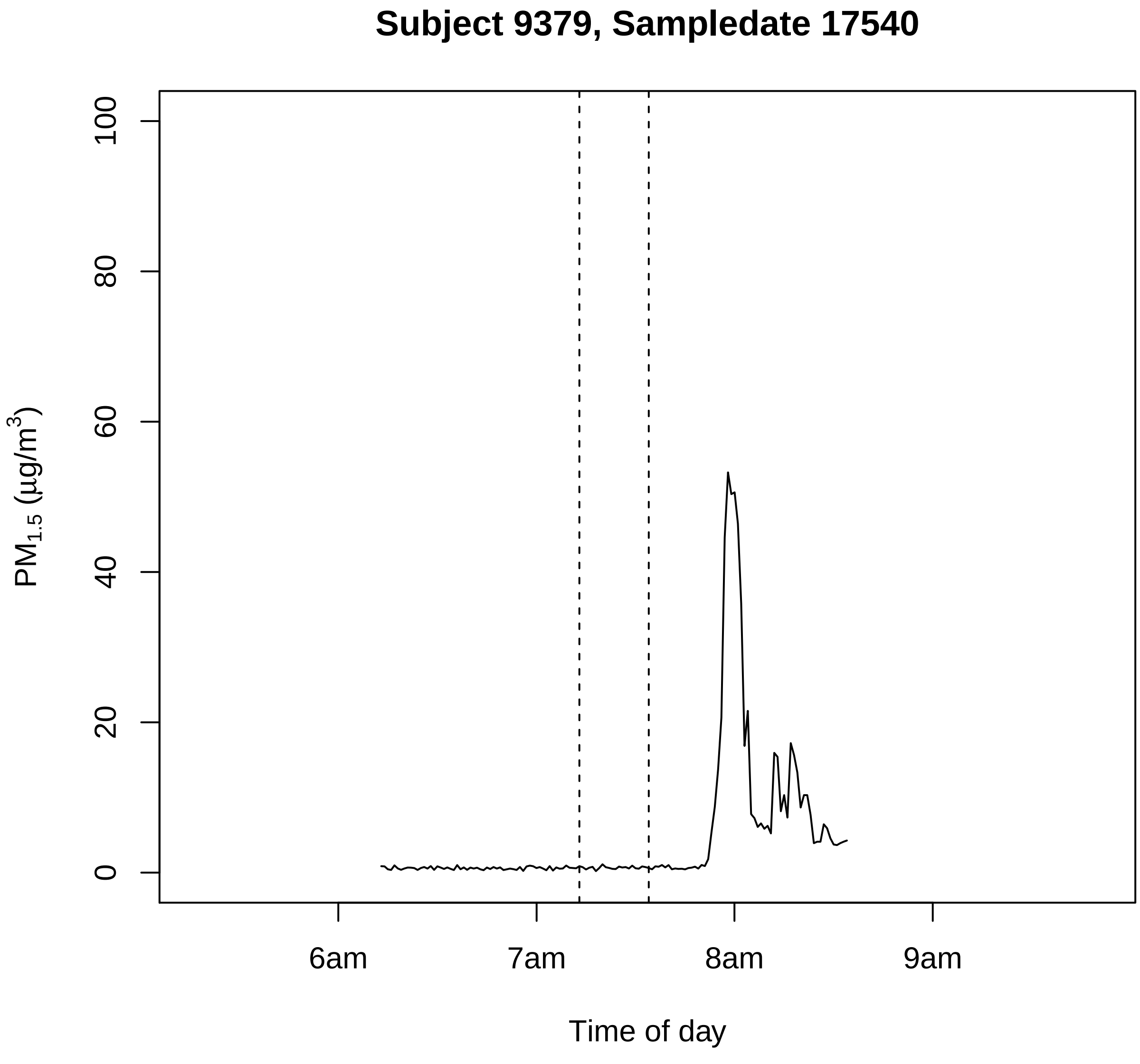
Subject 9379, Sampledate 17540
PM1.5 (µg/m3)
0
20
40
60
80
100
6am
7am
8am
9am
Time of day

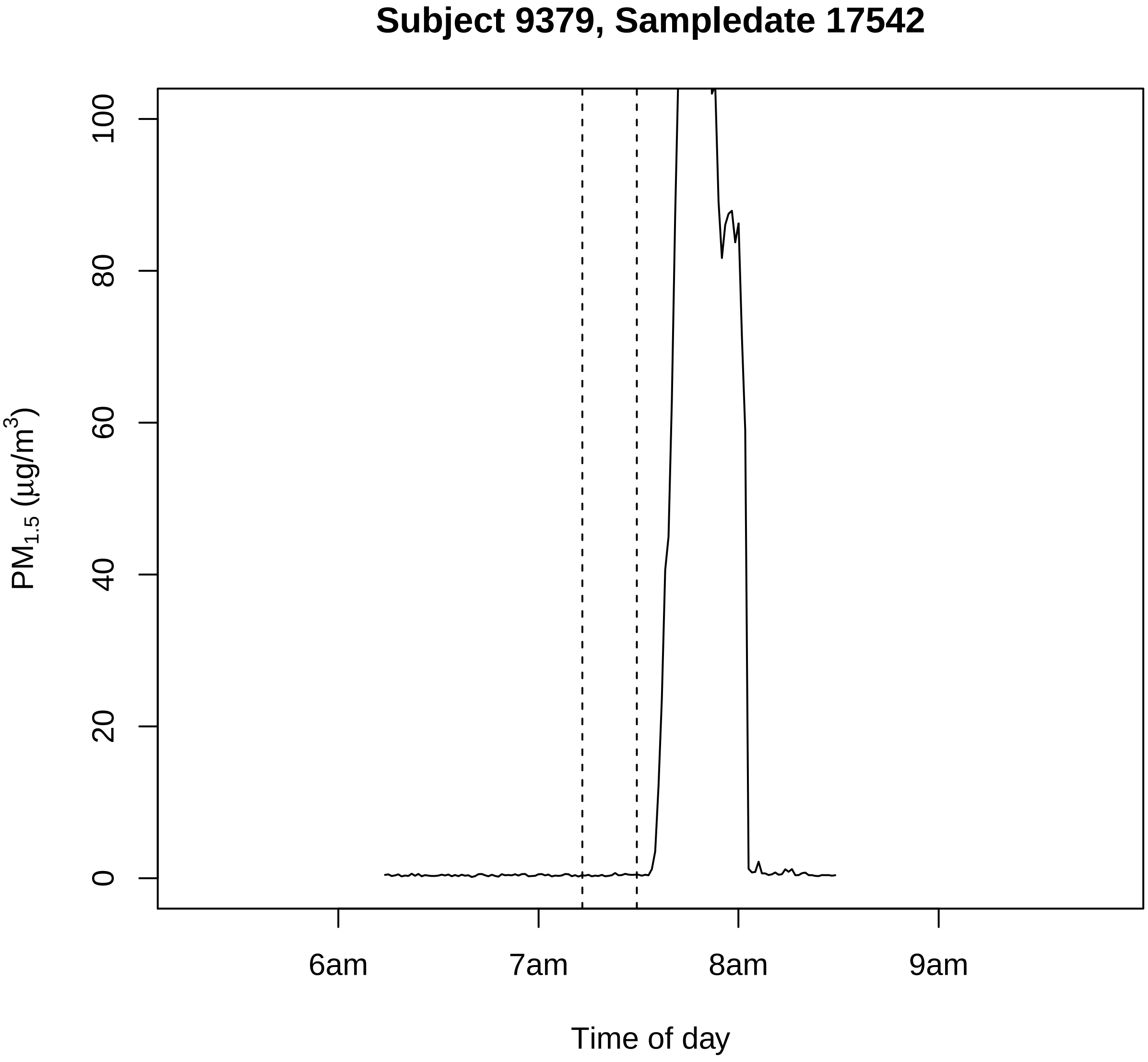
Subject 9379, Sampledate 17542
$PM_{1.5}$ (µg/m^3)
0
20
40
60
80
100
6am
7am
8am
9am
Time of day

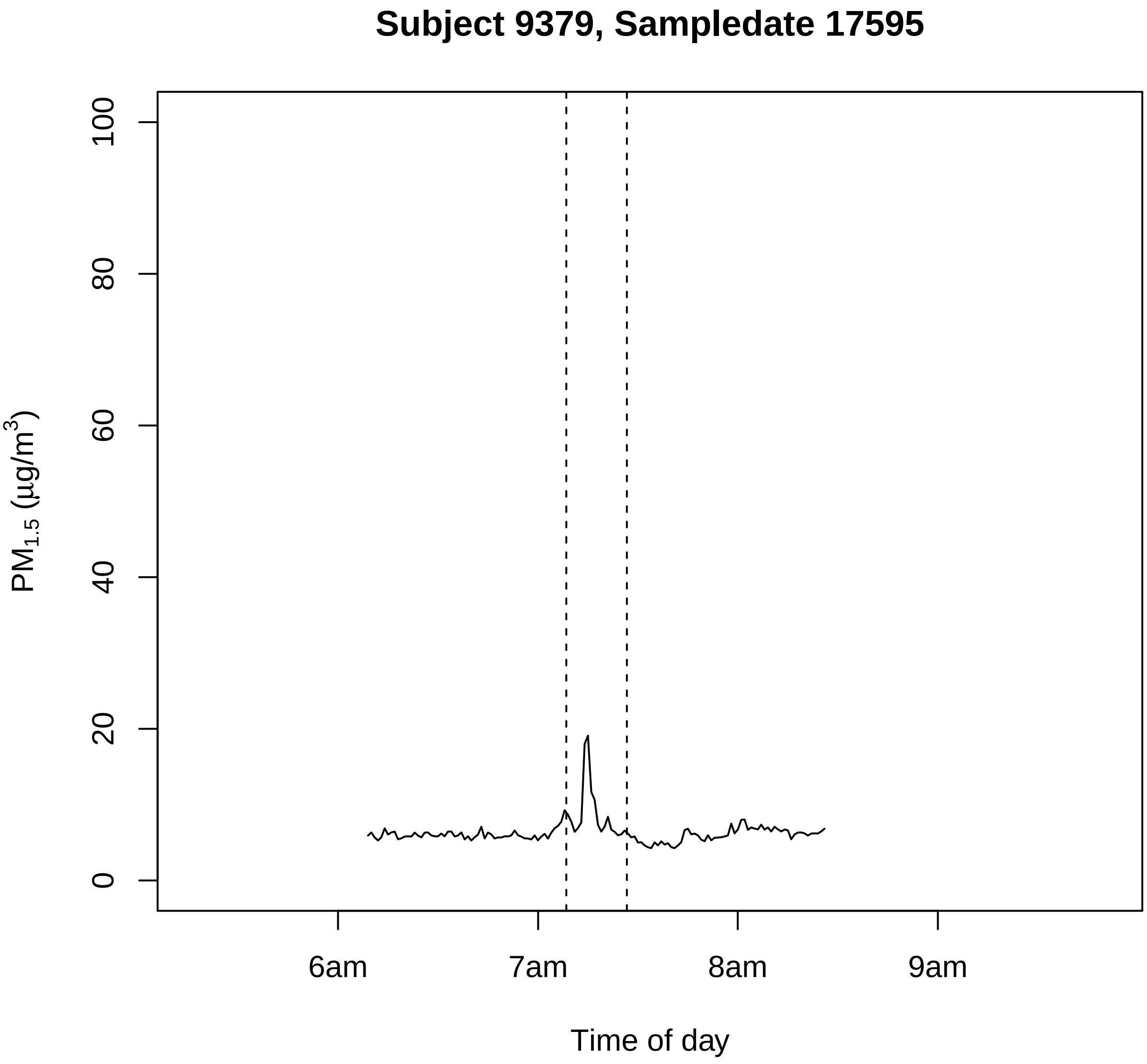

Subject 9379, Sampledate 17595
PM1.5 (µg/m3)
0
20
40
60
80
100
6am
7am
8am
9am
Time of day

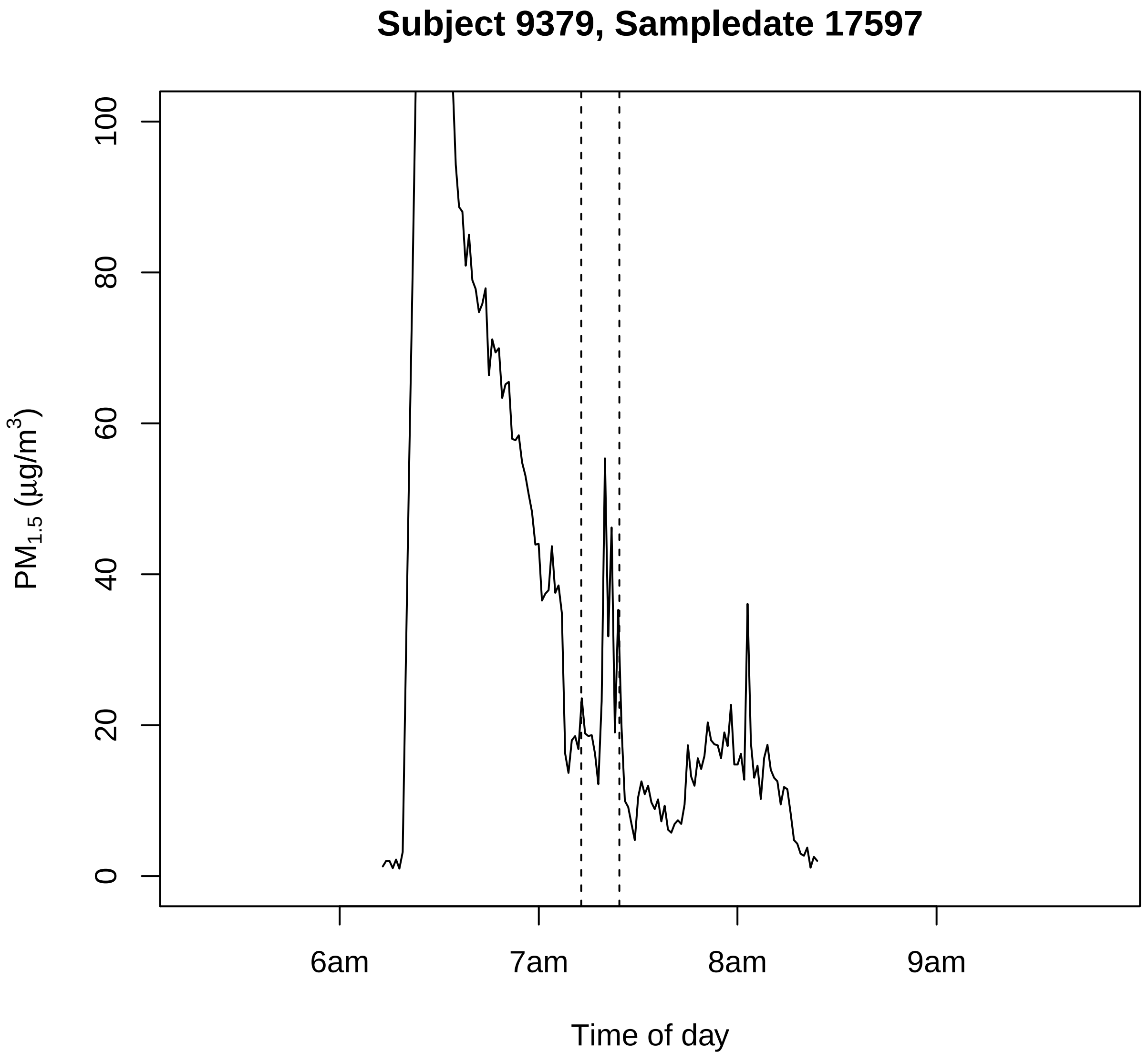

Subject 9379, Sampledate 17597
PM$_{1.5}$ (µg/m^3)
0
20
40
60
80
100
6am
7am
8am
9am
Time of day

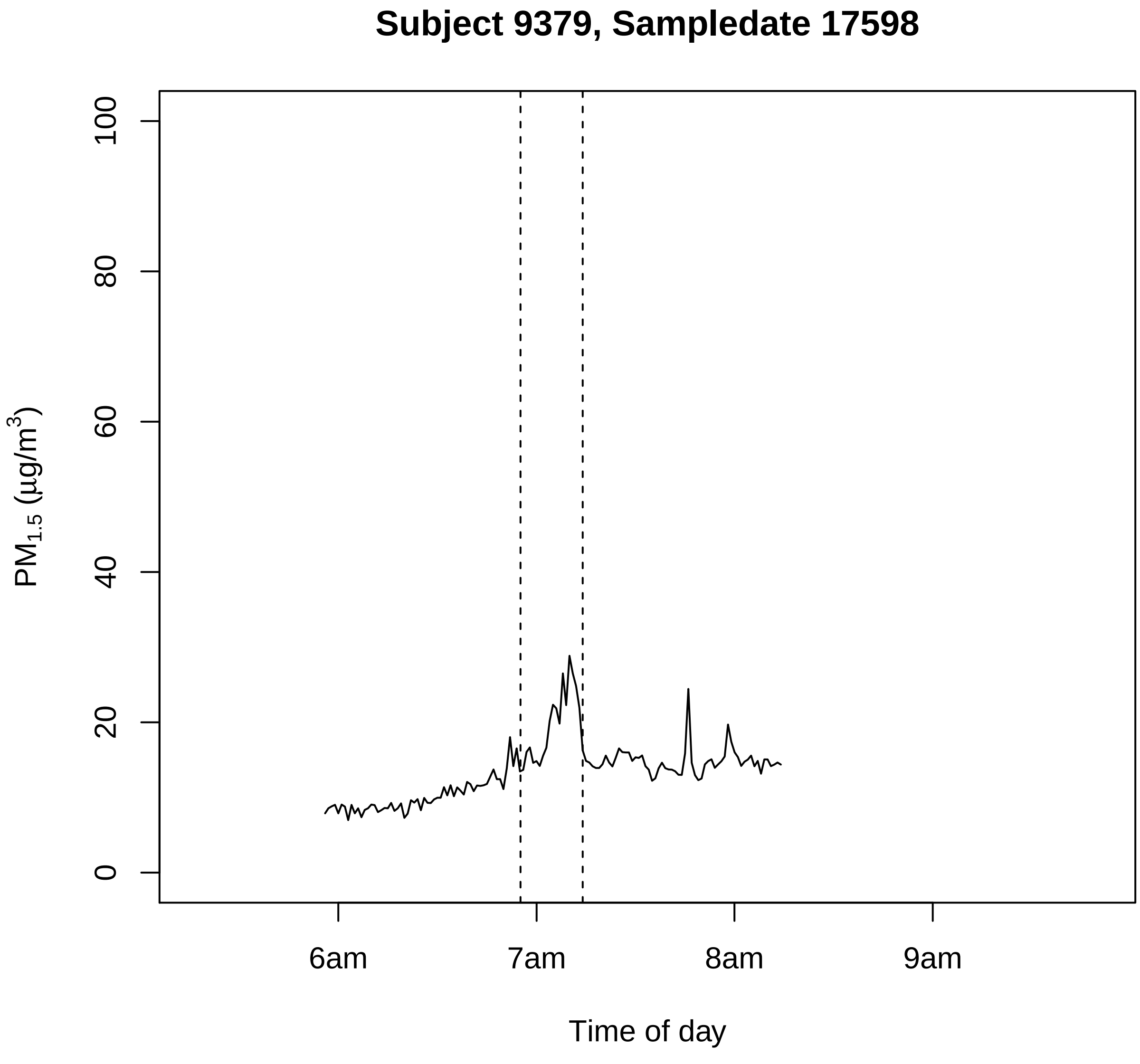
Subject 9379, Sampledate 17598
PM1.5 (µg/m3)
0
20
40
60
80
100
6am
7am
8am
9am
Time of day

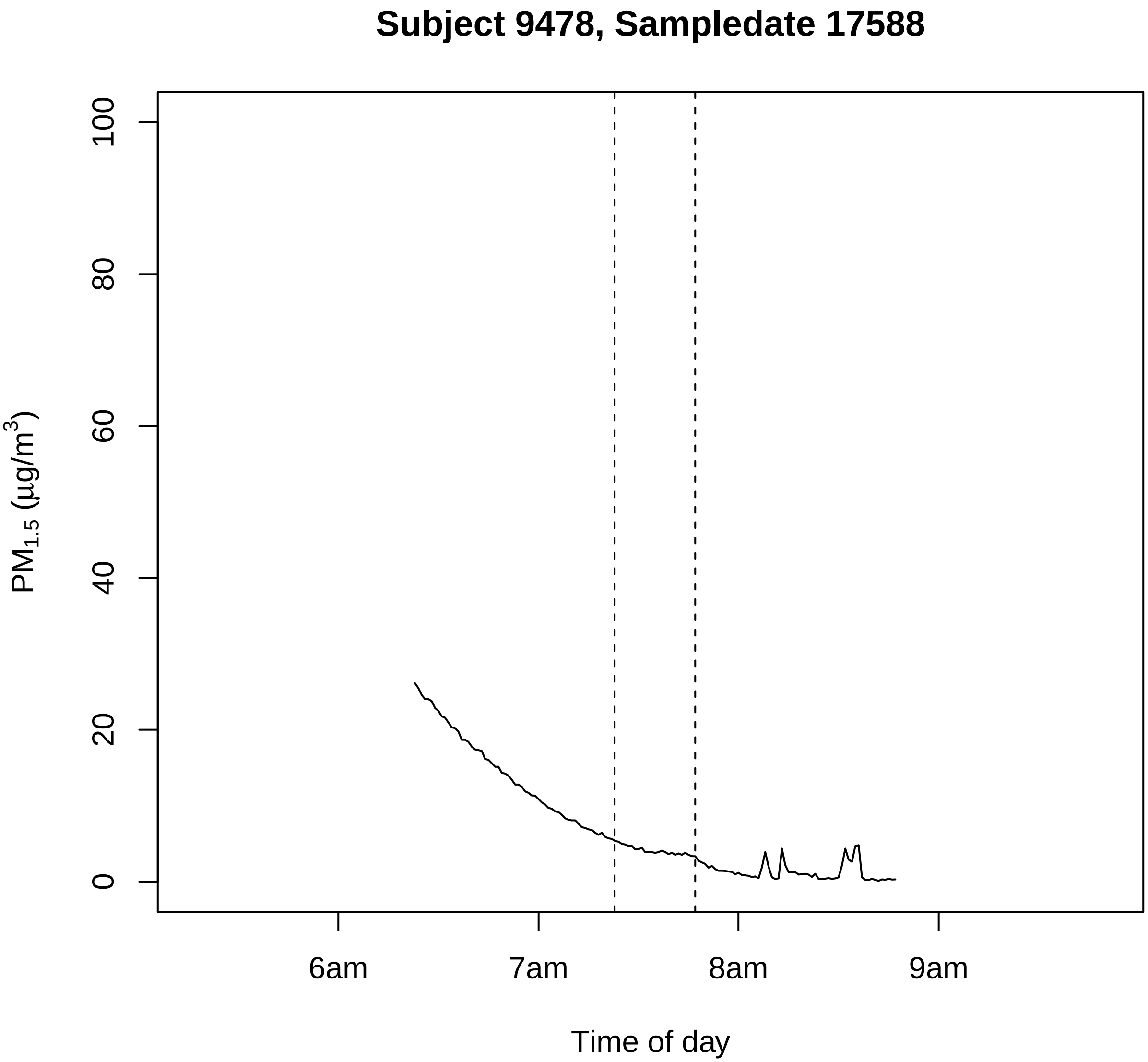

Subject 9478, Sampledate 17588
0
20
40
60
80
100
PM1.5 (µg/m3)
6am
7am
8am
9am
Time of day

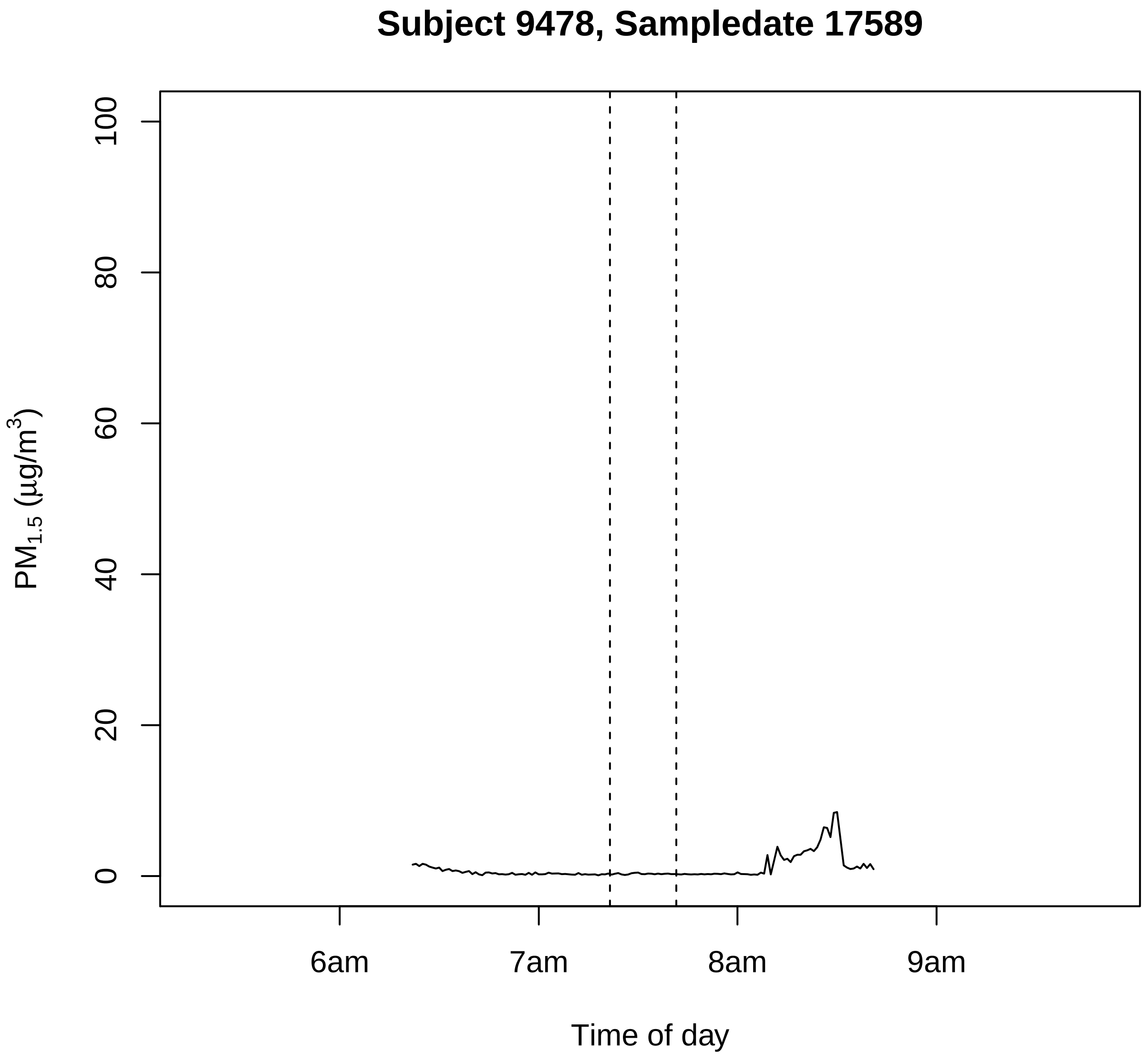
Subject 9478, Sampledate 17589
0
20
40
60
80
100
PM1.5 (µg/m3)
6am
7am
8am
9am
Time of day

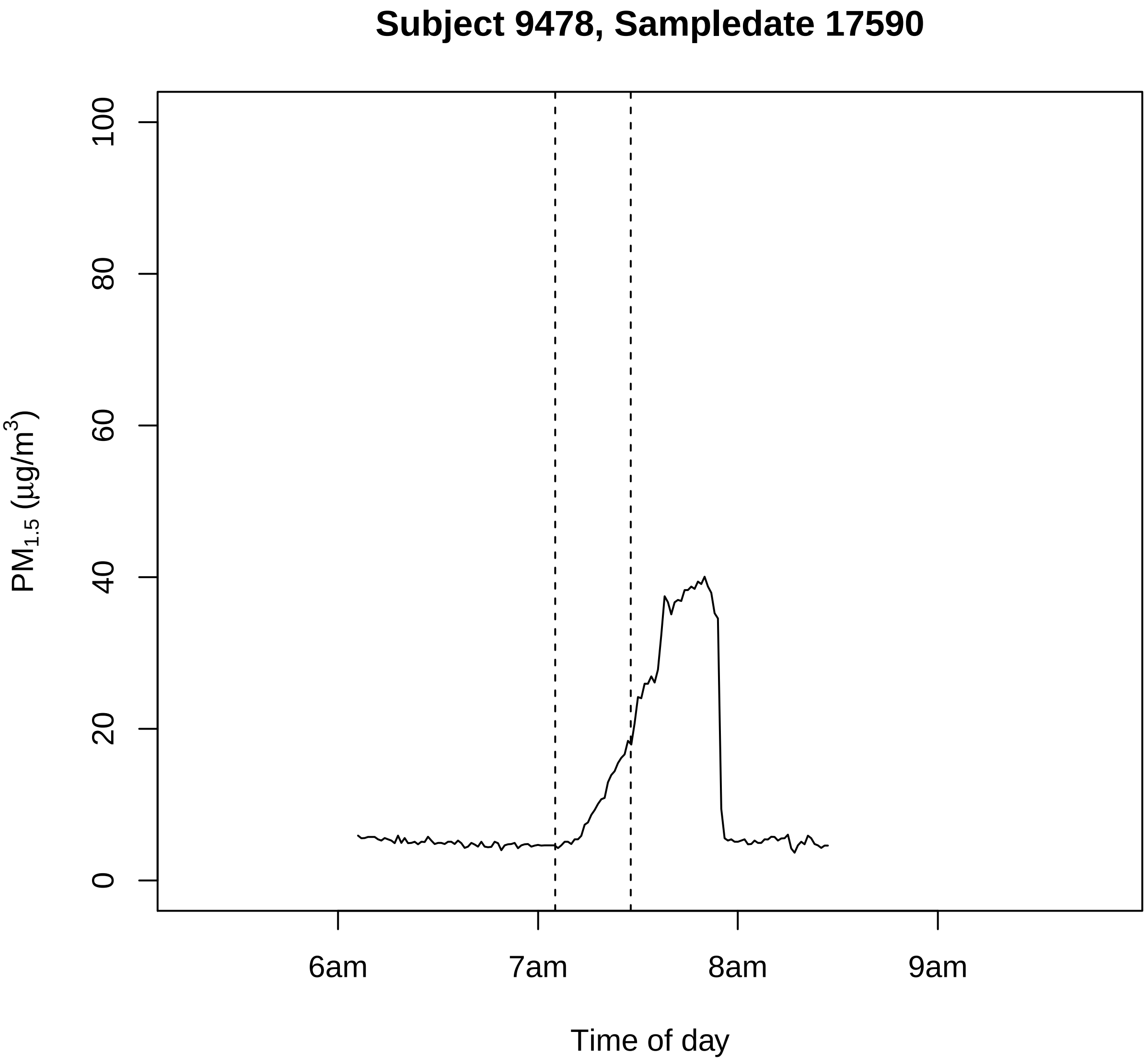

Subject 9478, Sampledate 17590
PM1.5 (µg/m3)
0
20
40
60
80
100
6am
7am
8am
9am
Time of day

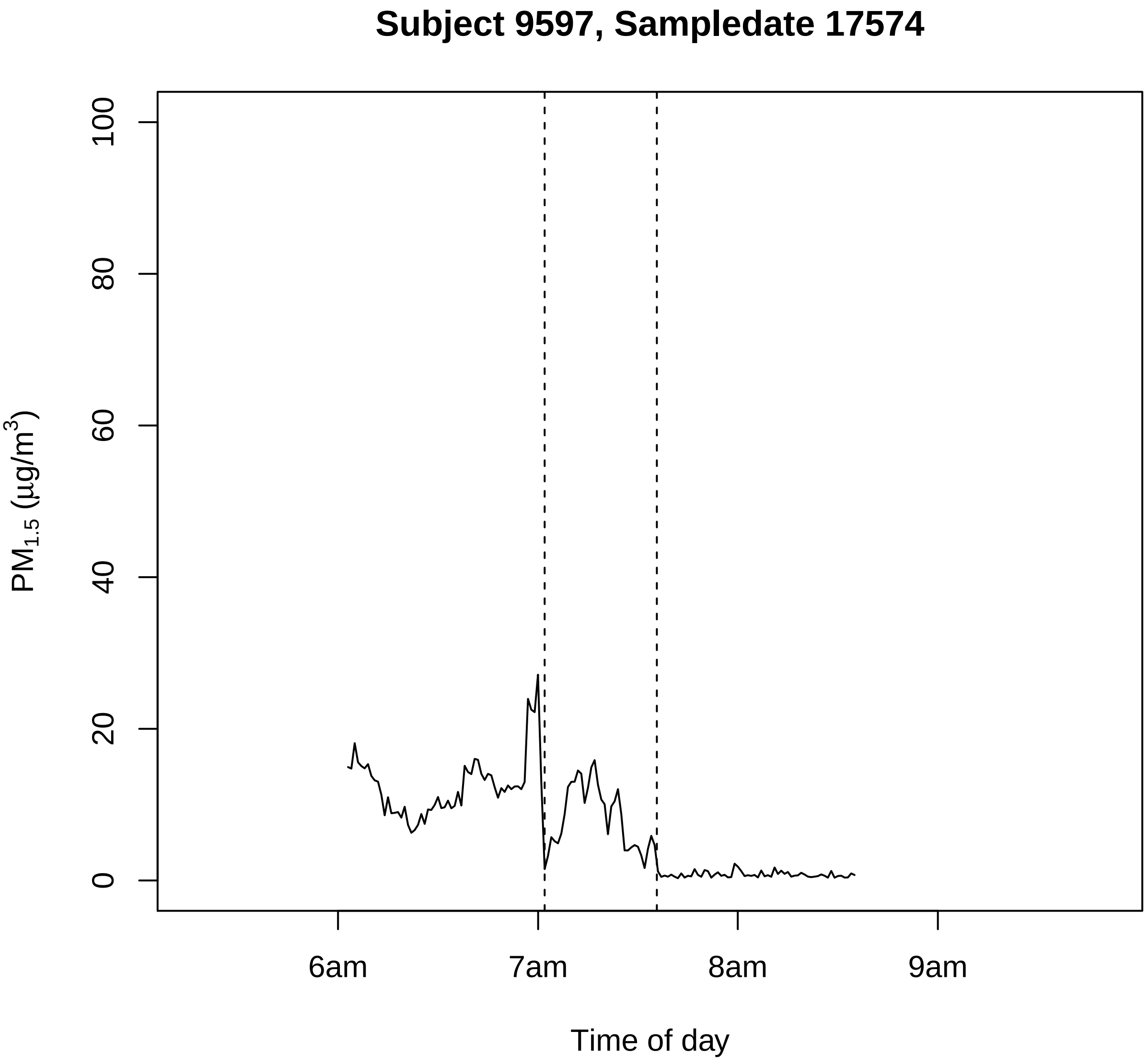

Subject 9597, Sampledate 17574
PM1.5 (µg/m3)
0
20
40
60
80
100
6am
7am
8am
9am
Time of day

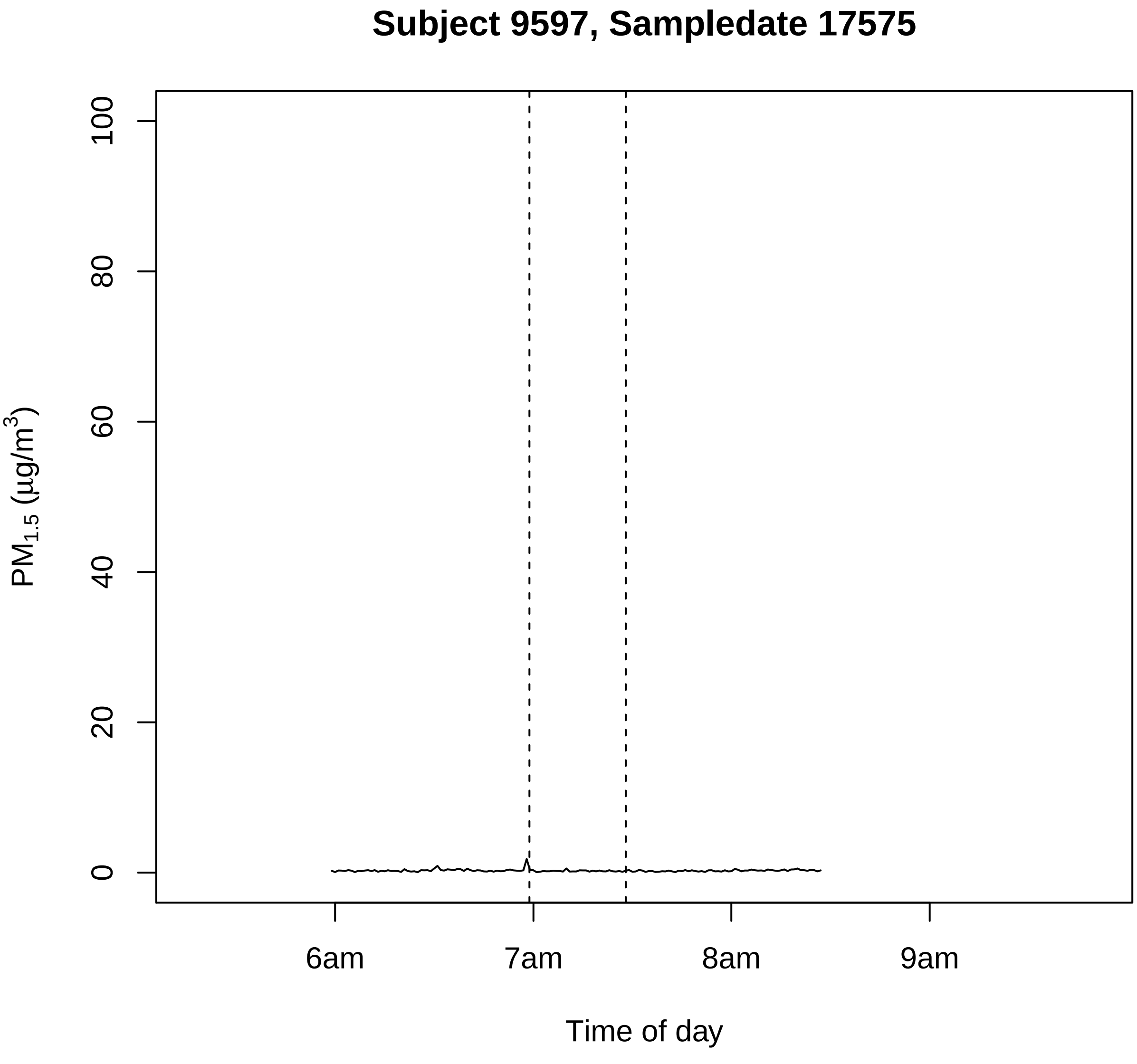

Subject 9597, Sampledate 17575
0
20
40
60
80
100
PM1.5 (µg/m3)
6am
7am
8am
9am
Time of day

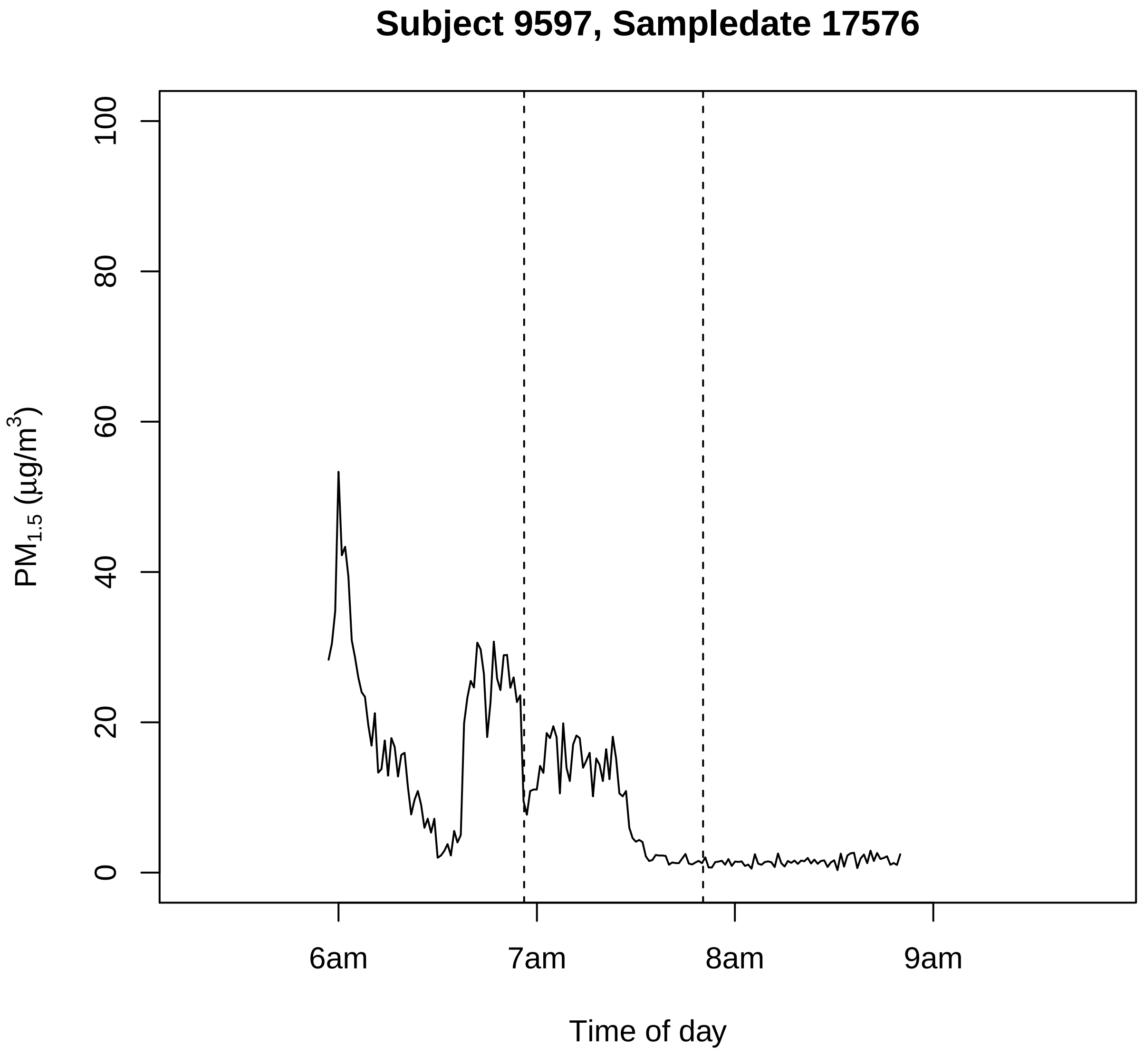
Subject 9597, Sampledate 17576
$PM_{1.5}$ (µg/m^3)
0
20
40
60
80
100
6am
7am
8am
9am
Time of day

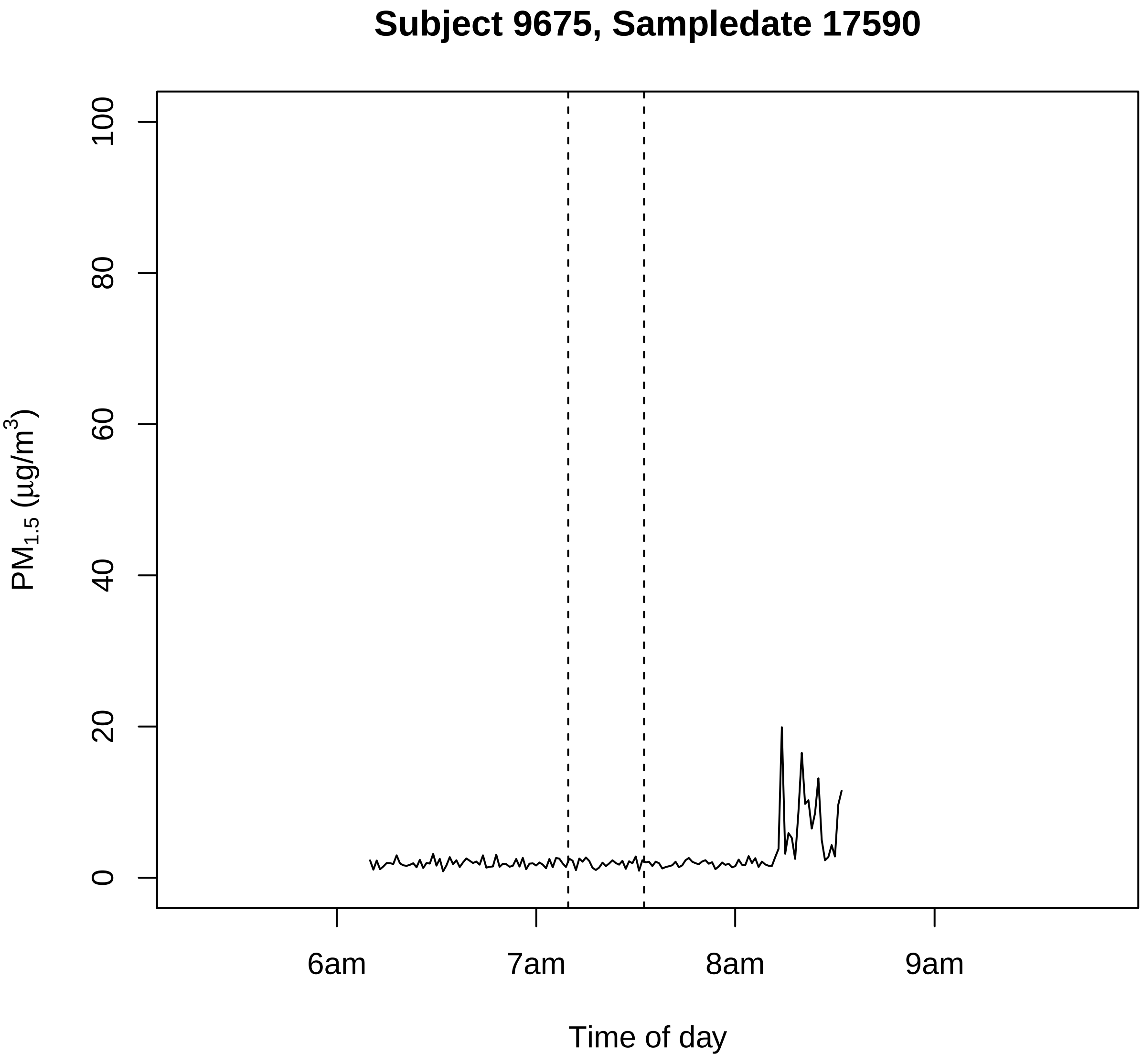

Subject 9675, Sampledate 17590
$PM_{1.5}$ (µg/m^3)
0
20
40
60
80
100
6am
7am
8am
9am
Time of day

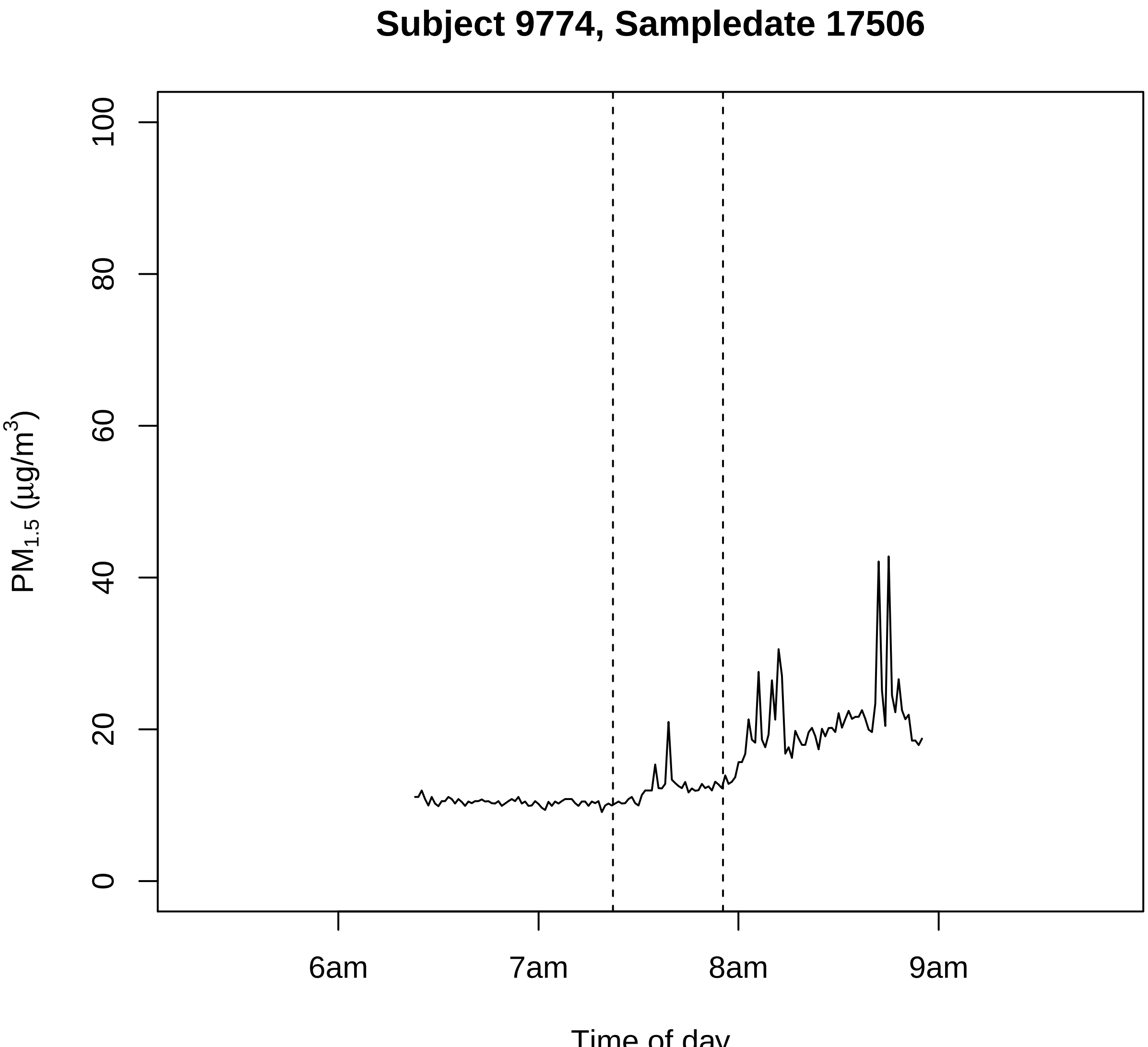

Subject 9774, Sampledate 17506
PM1.5 (µg/m3)
0
20
40
60
80
100
6am
7am
8am
9am
Time of day

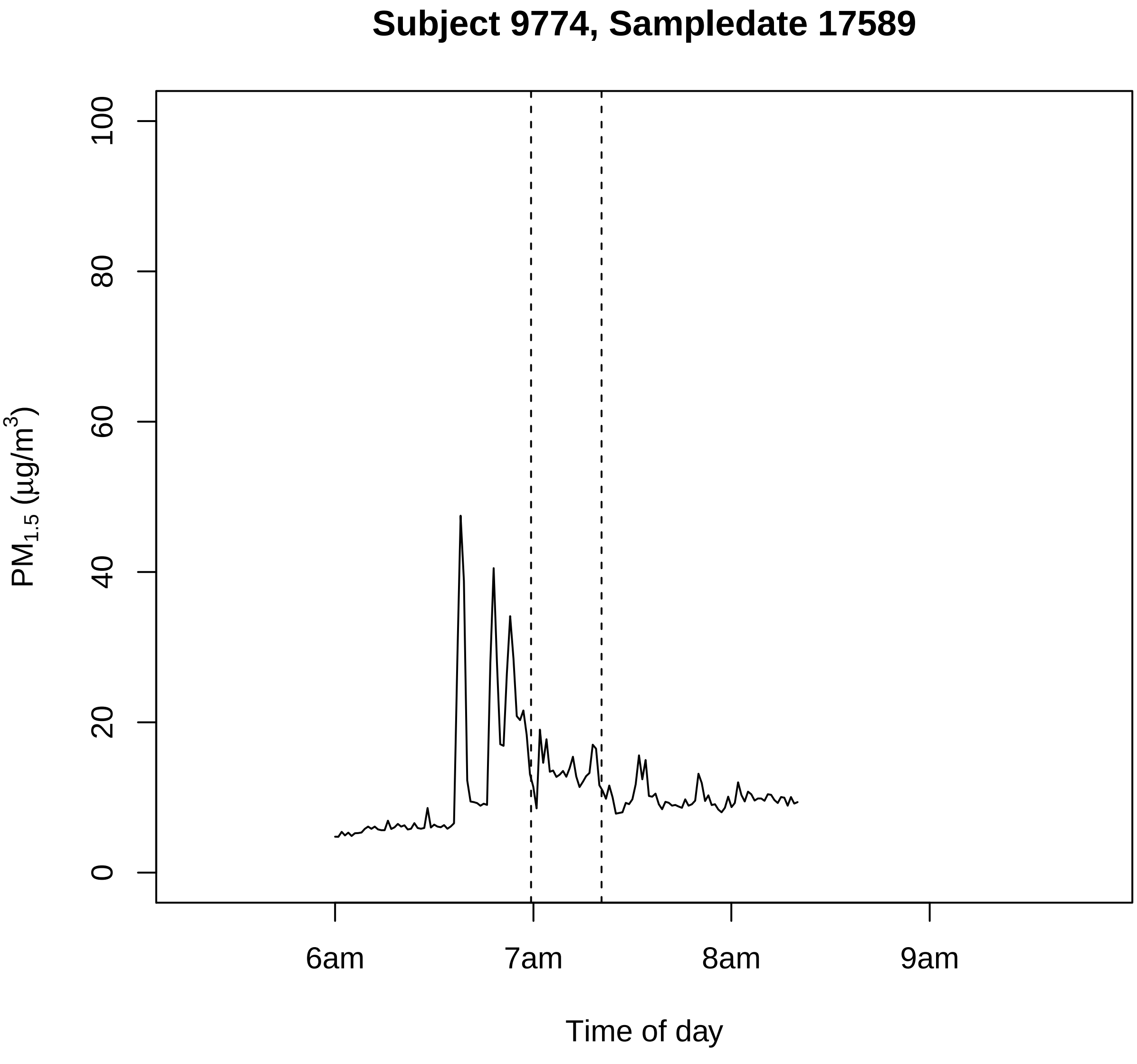
Subject 9774, Sampledate 17589
PM1.5 (μg/m3)
0
20
40
60
80
100
6am
7am
8am
9am
Time of day

Subject 9774, Sampledate 17590

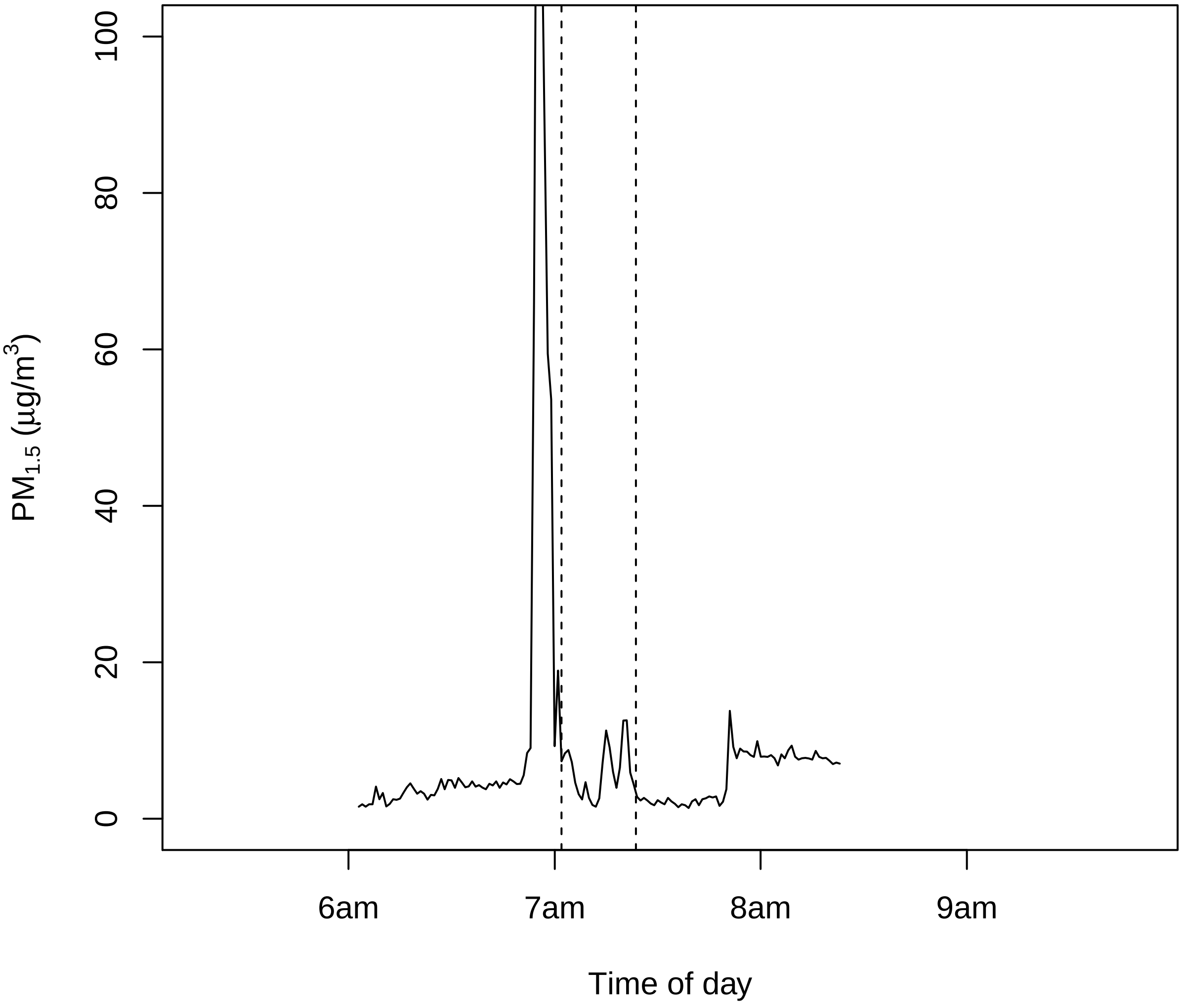

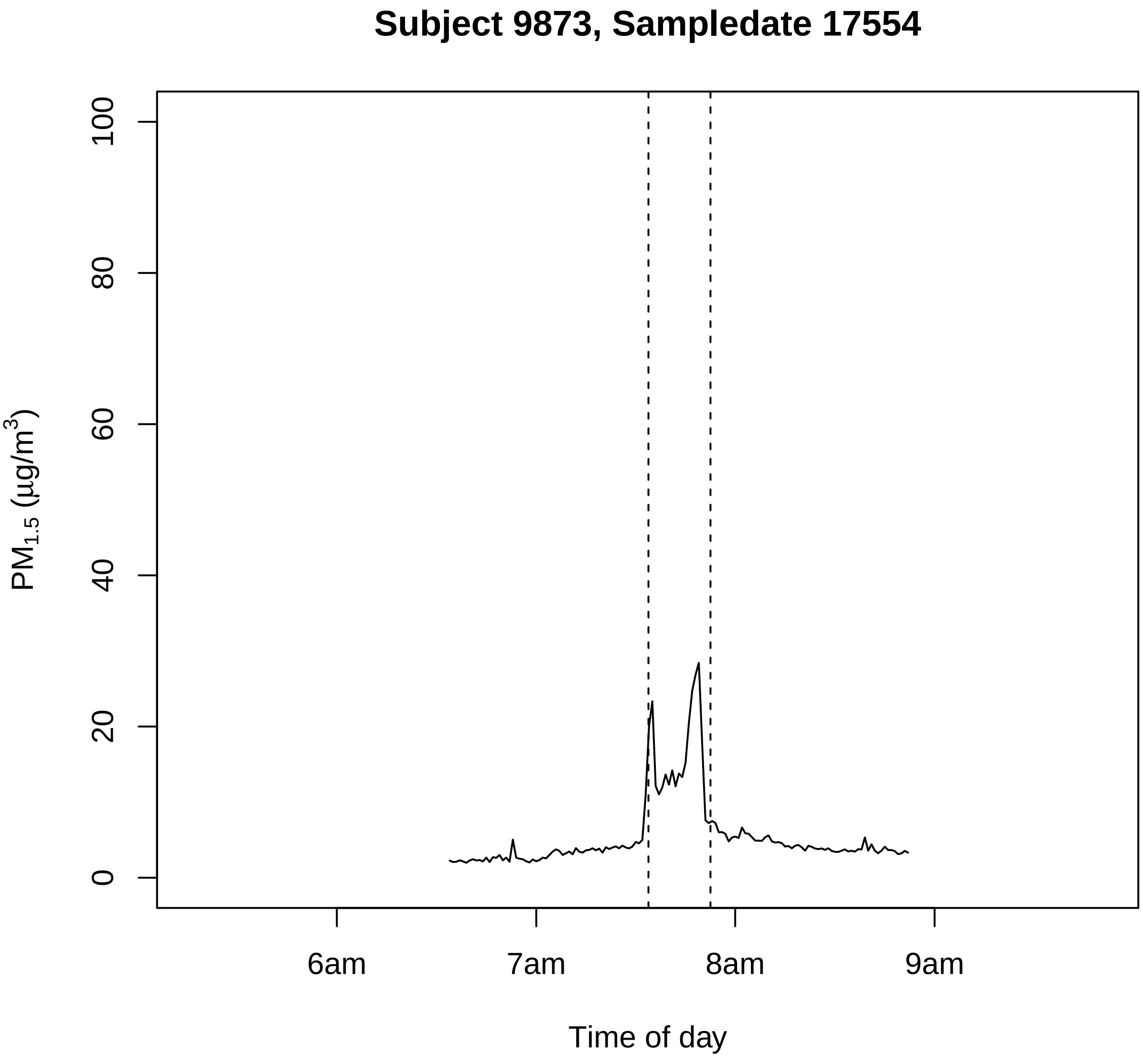

Subject 9873, Sampledate 17554
0
20
40
60
80
100
PM1.5 (µg/m3)
6am
7am
8am
9am
Time of day

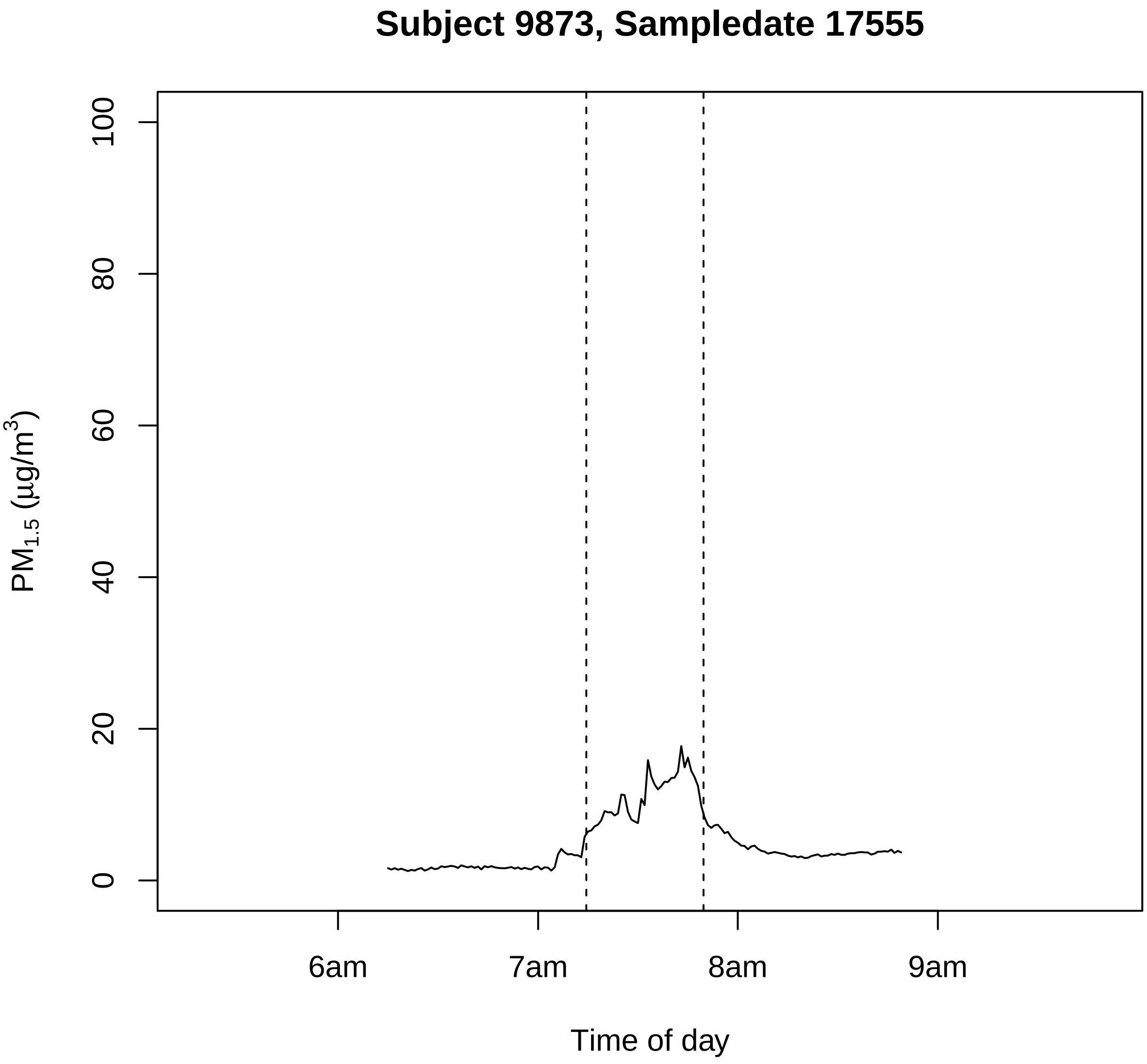
Subject 9873, Sampledate 17555
PM1.5 (µg/m3)
0
20
40
60
80
100
6am
7am
8am
9am
Time of day

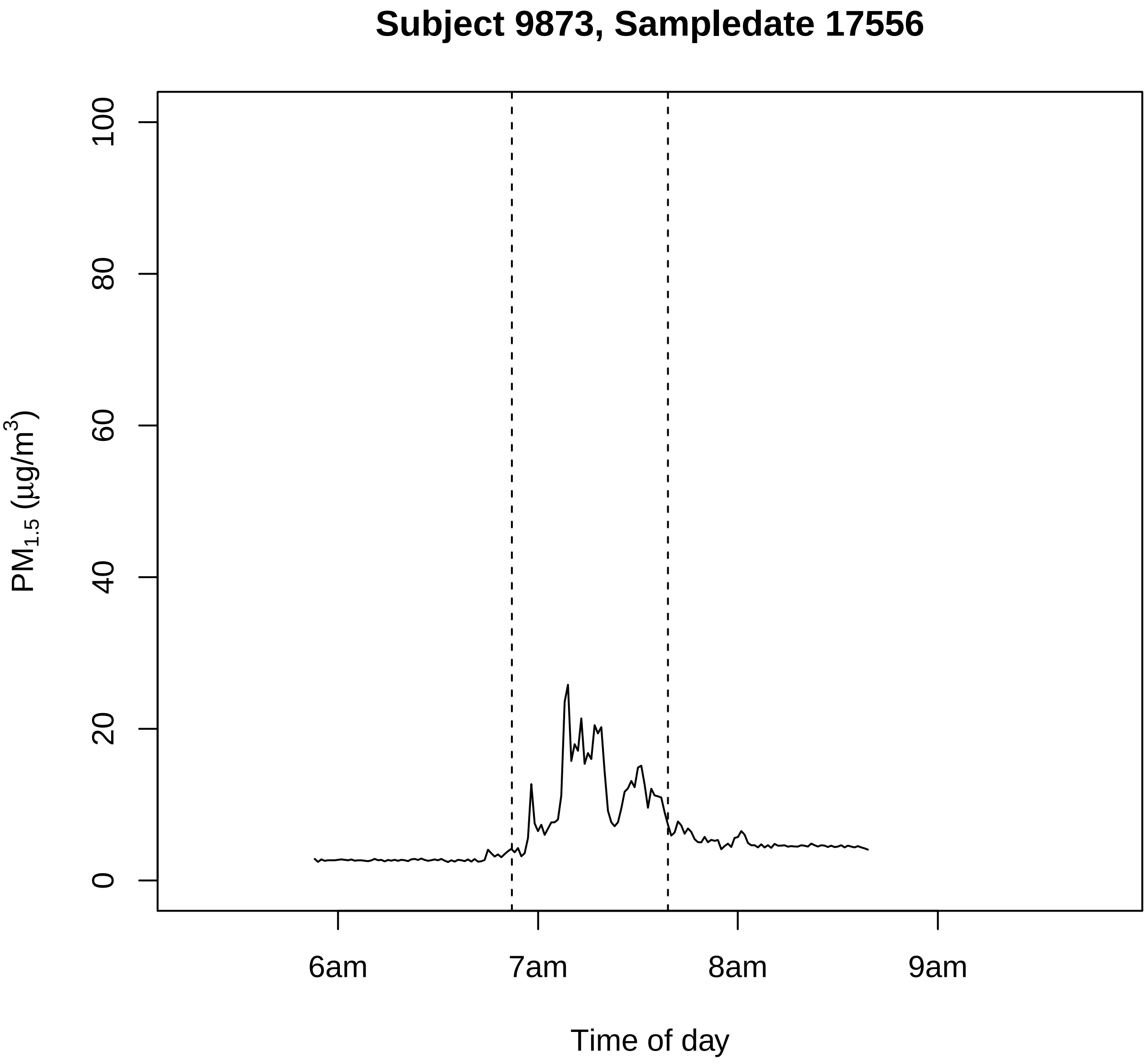

Subject 9873, Sampledate 17556
PM1.5 (µg/m3)
0
20
40
60
80
100
6am
7am
8am
9am
Time of day

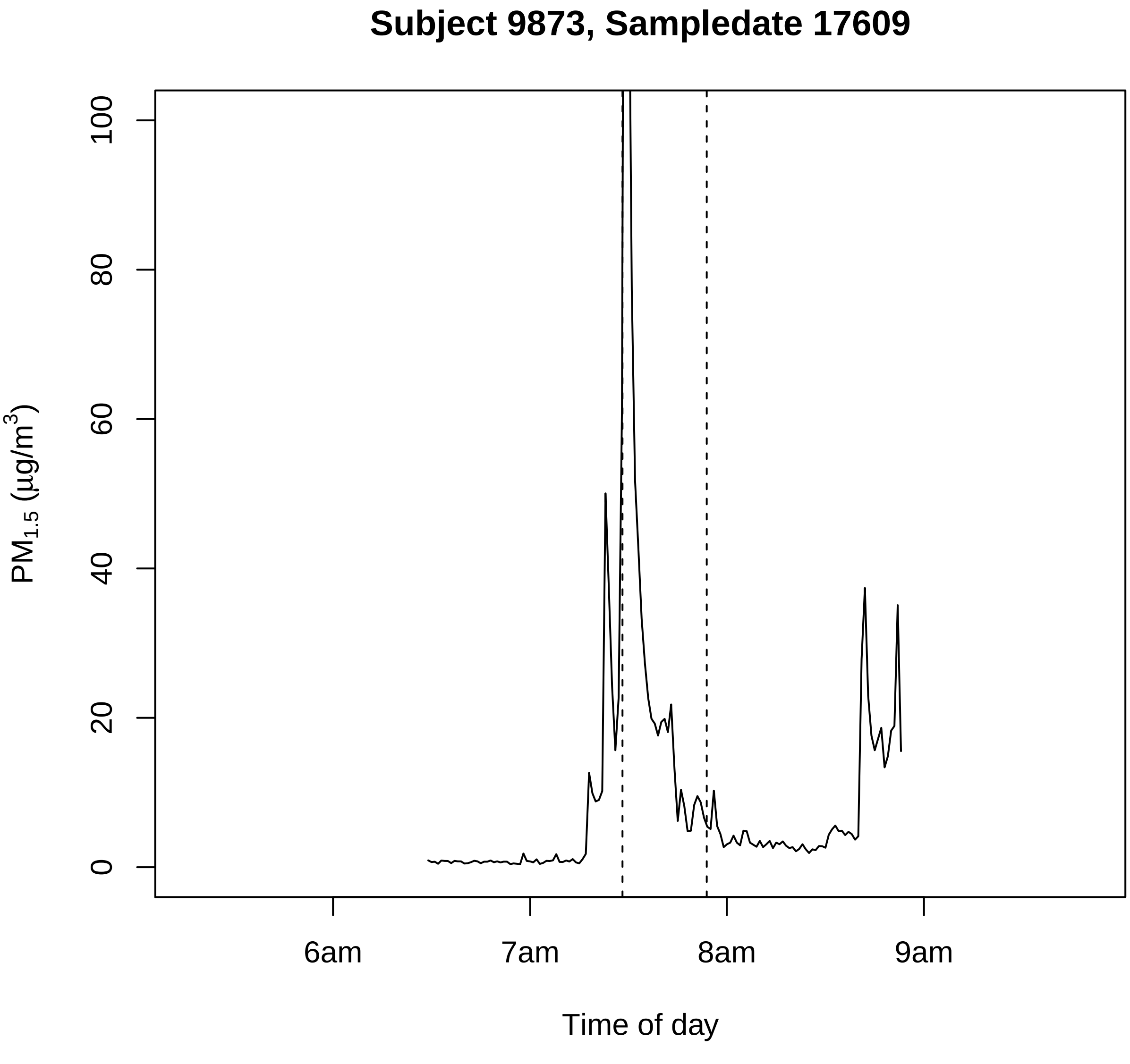

Subject 9873, Sampledate 17609
PM1.5 (µg/m3)
0
20
40
60
80
100
6am
7am
8am
9am
Time of day

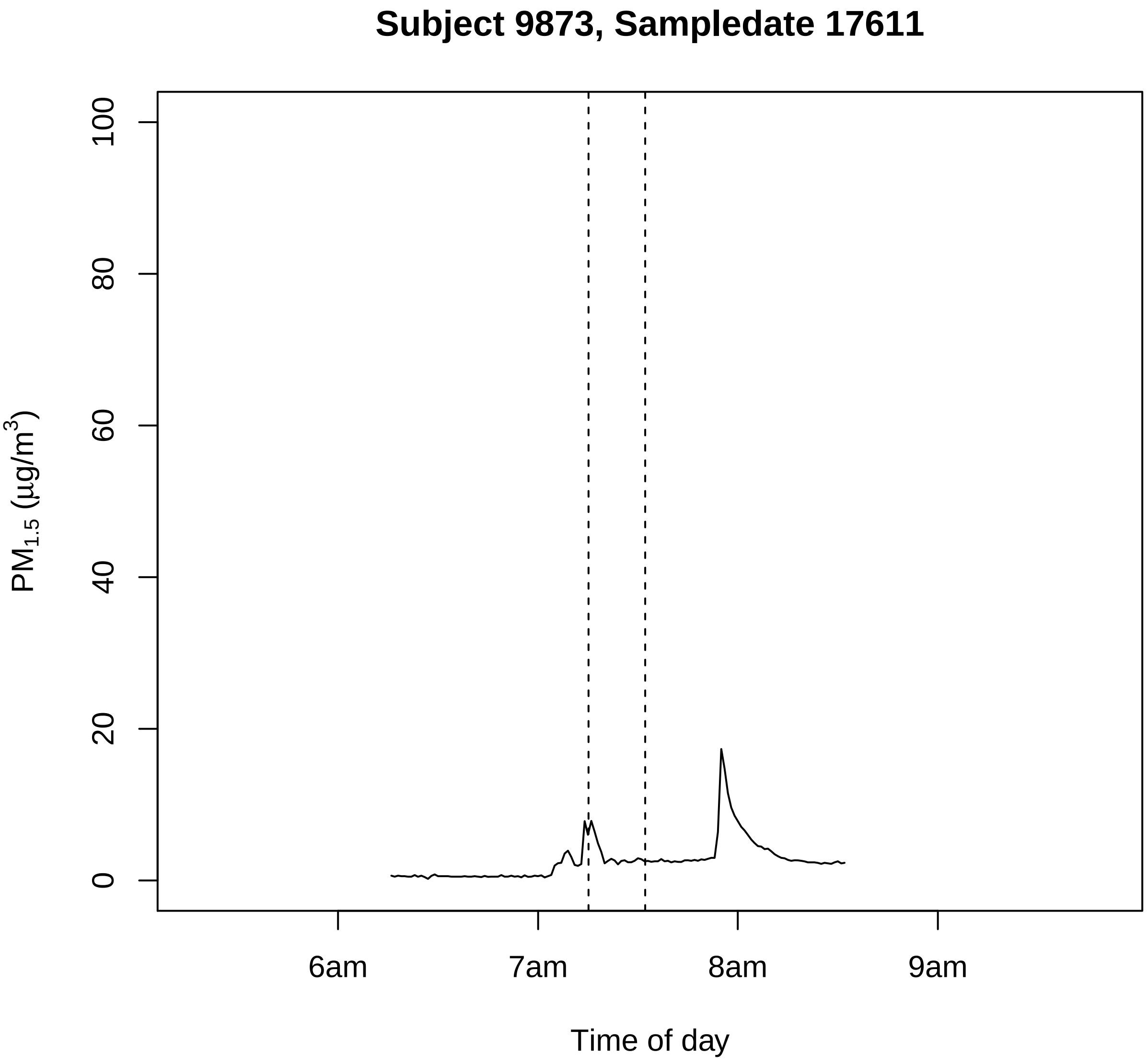

Subject 9873, Sampledate 17611
PM1.5 (µg/m3)
0
20
40
60
80
100
6am
7am
8am
9am
Time of day

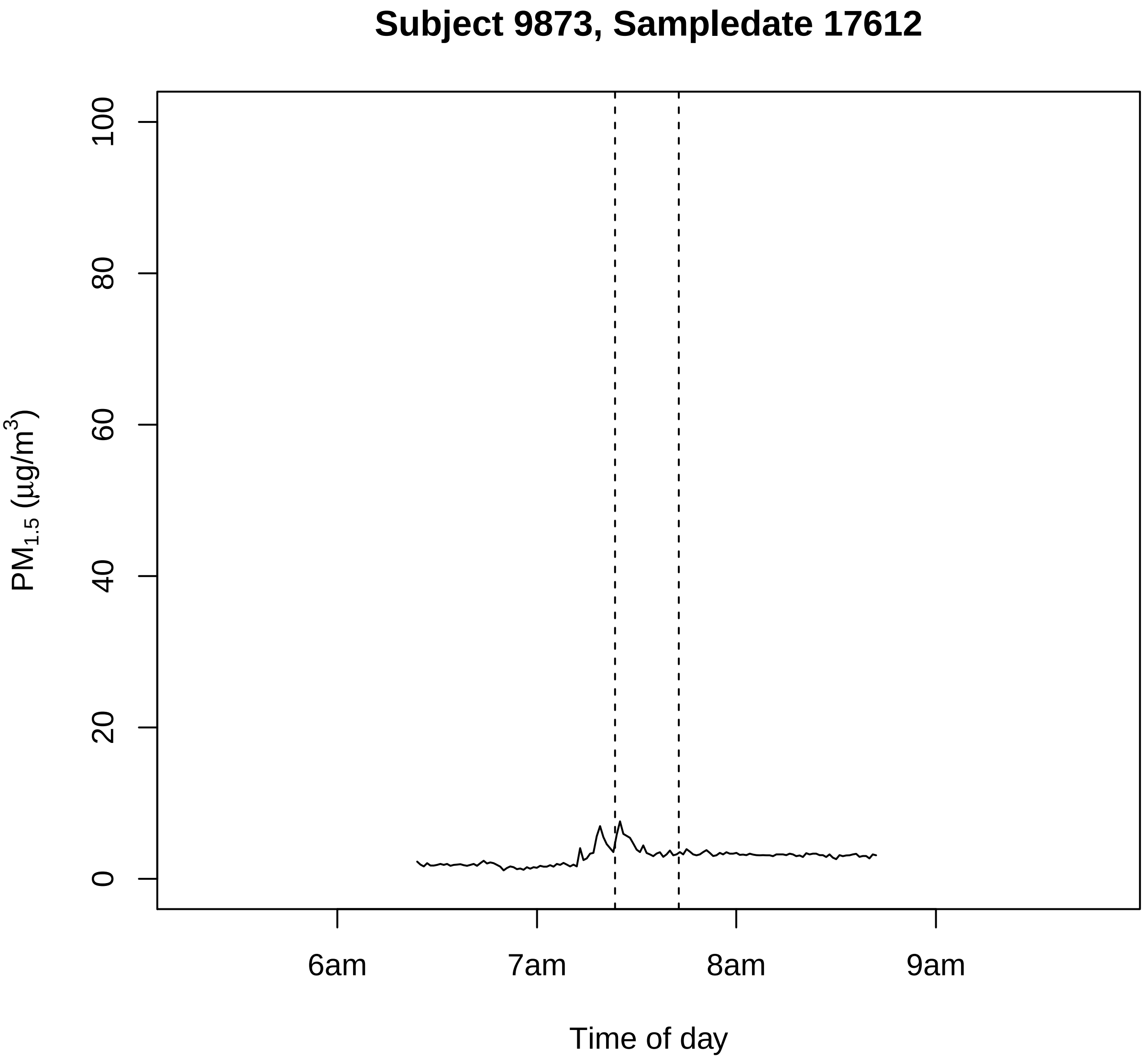

Subject 9873, Sampledate 17612
$PM_{1.5}$ (µg/m³)
0
20
40
60
80
100
6am
7am
8am
9am
Time of day

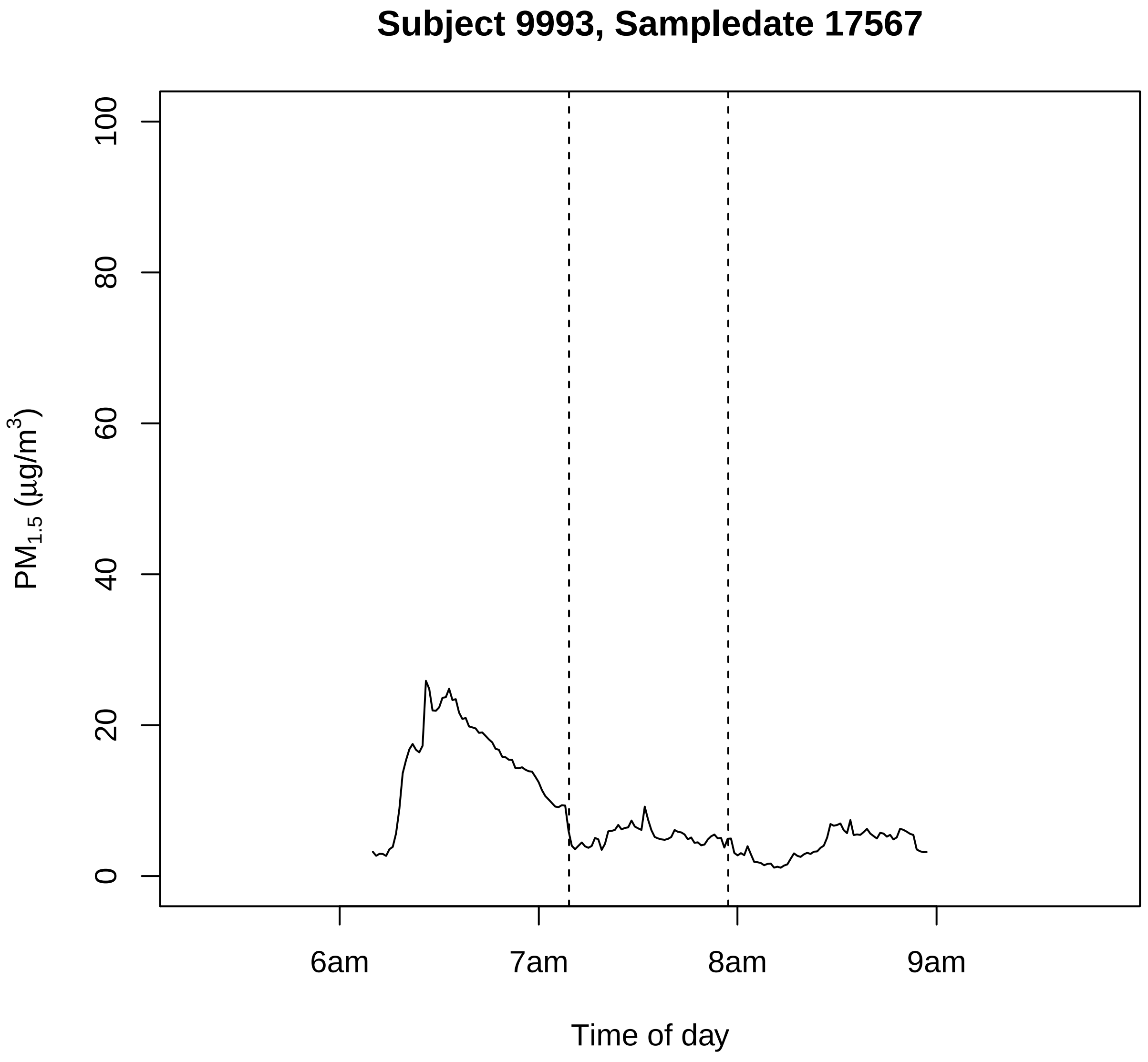

Subject 9993, Sampledate 17567
PM1.5 (µg/m3)
0
20
40
60
80
100
6am
7am
8am
9am
Time of day

Subject 9993, Sampledate 17569

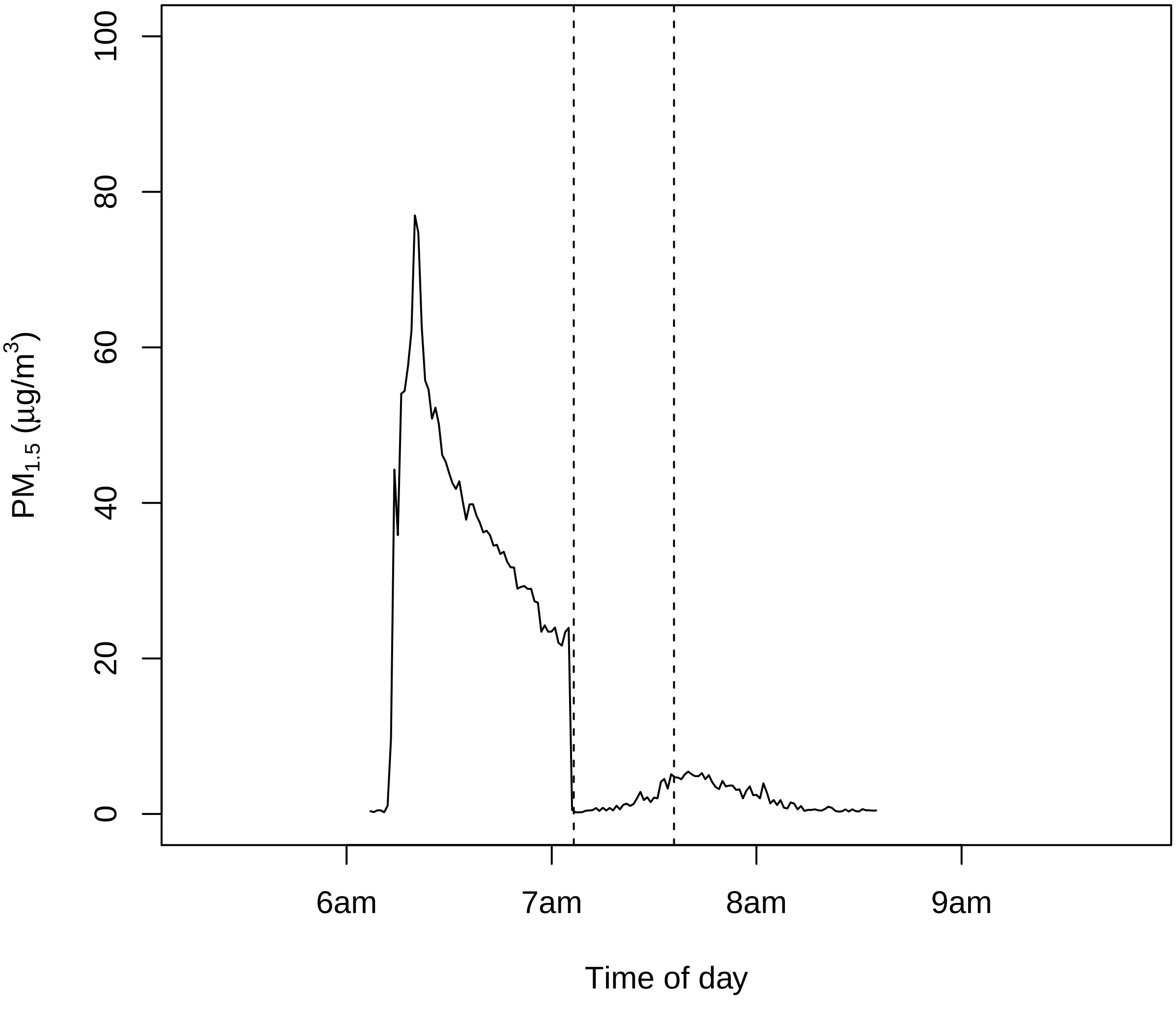

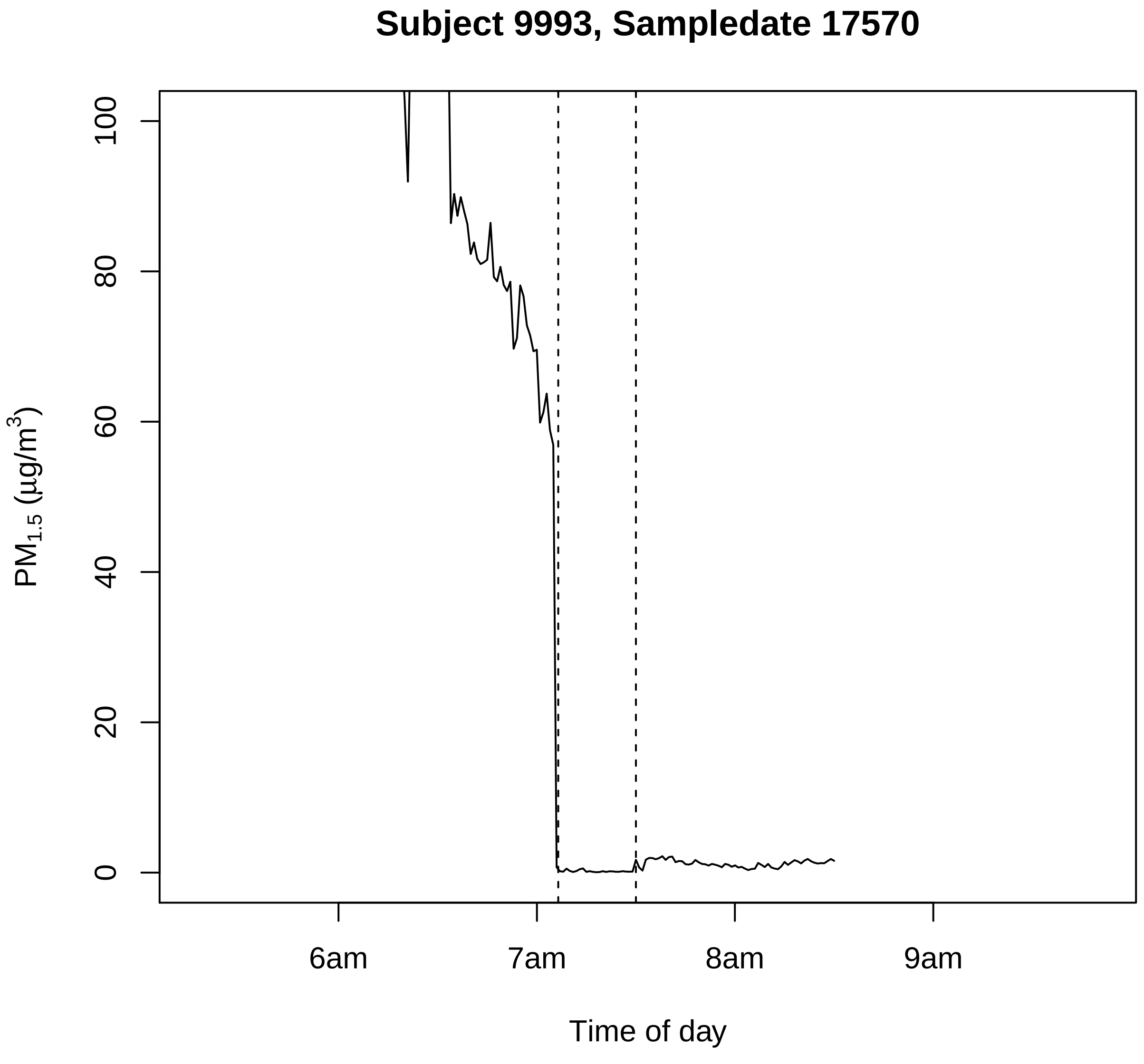
Subject 9993, Sampledate 17570
PM1.5 (µg/m3)
0
20
40
60
80
100
6am
7am
8am
9am
Time of day

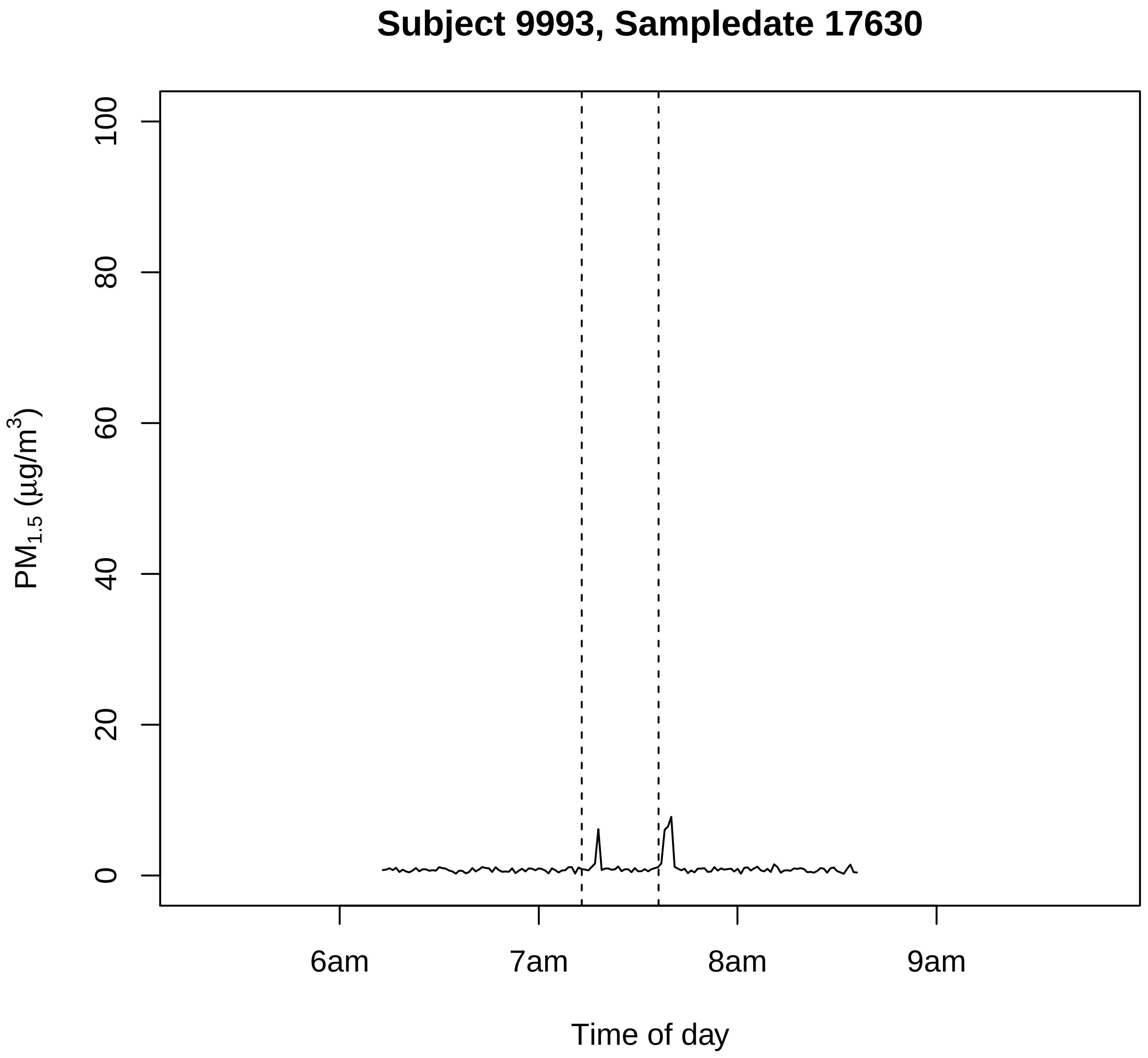
Subject 9993, Sampledate 17630
$PM_{1.5}$ (µg/m^3)
0
20
40
60
80
100
6am
7am
8am
9am
Time of day

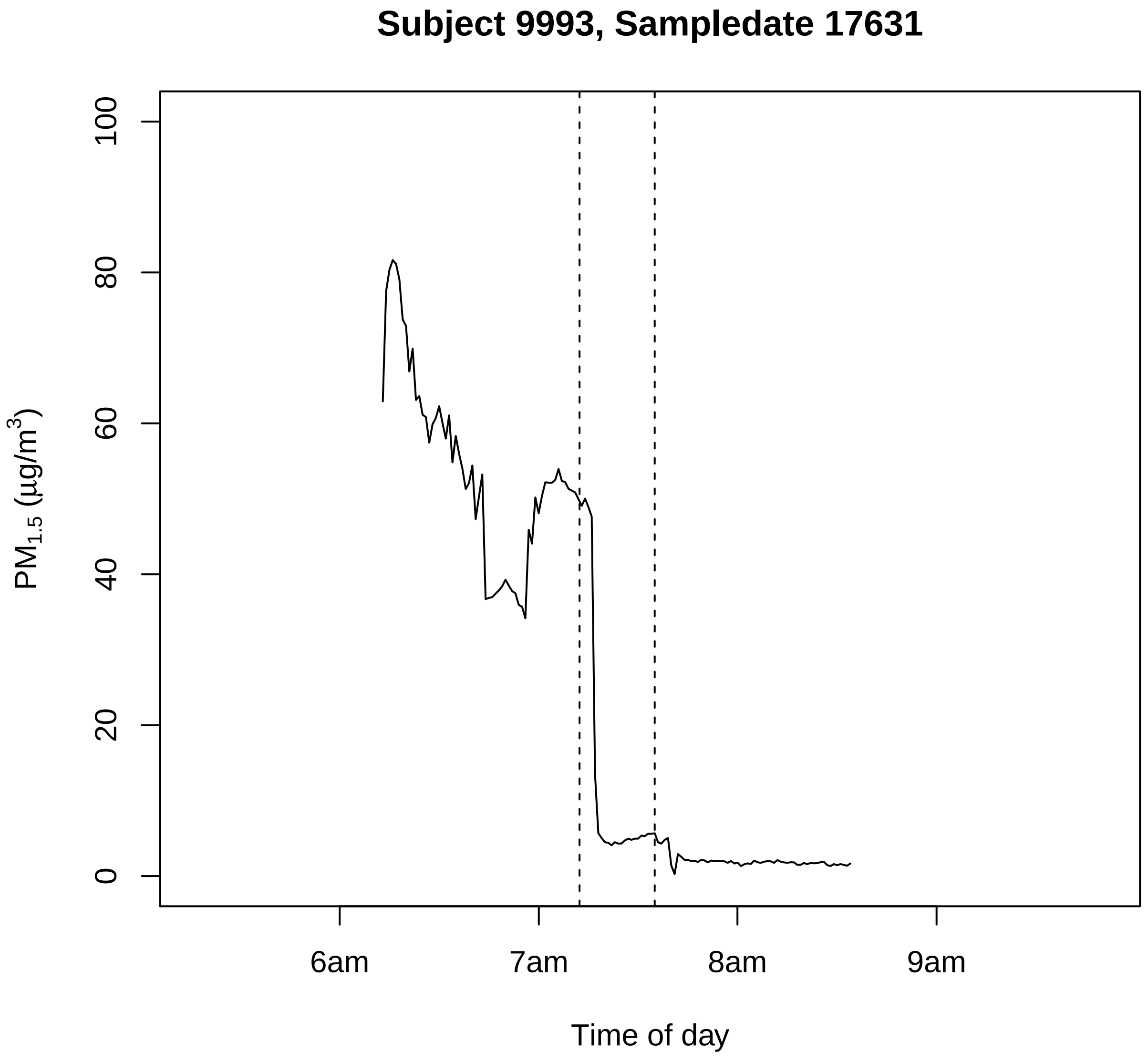

Subject 9993, Sampledate 17631
0
20
40
60
80
100
6am
7am
8am
9am
Time of day
PM1.5 (µg/m3)

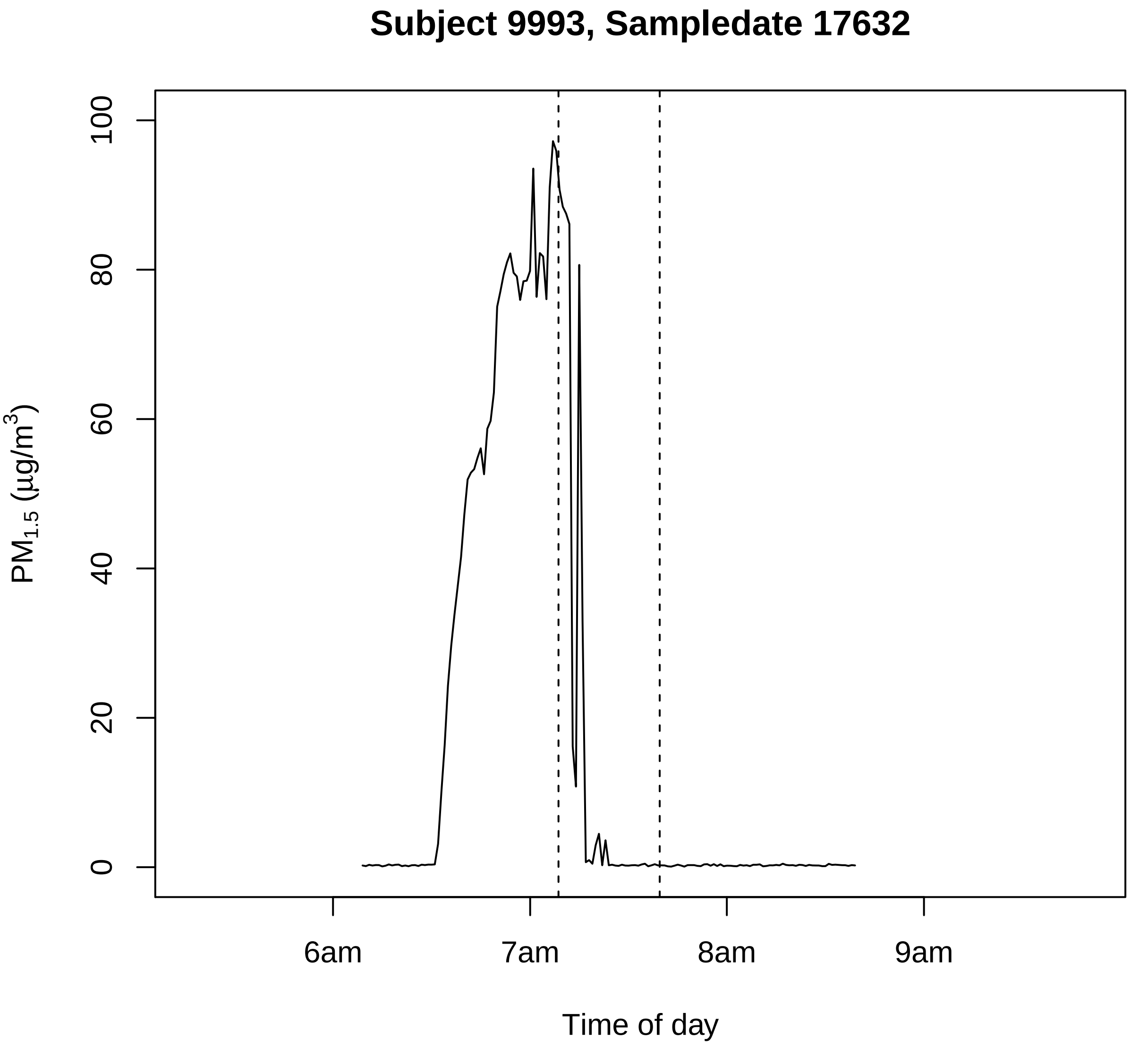

Subject 9993, Sampledate 17632
0
20
40
60
80
100
PM1.5 (µg/m3)
6am
7am
8am
9am
Time of day

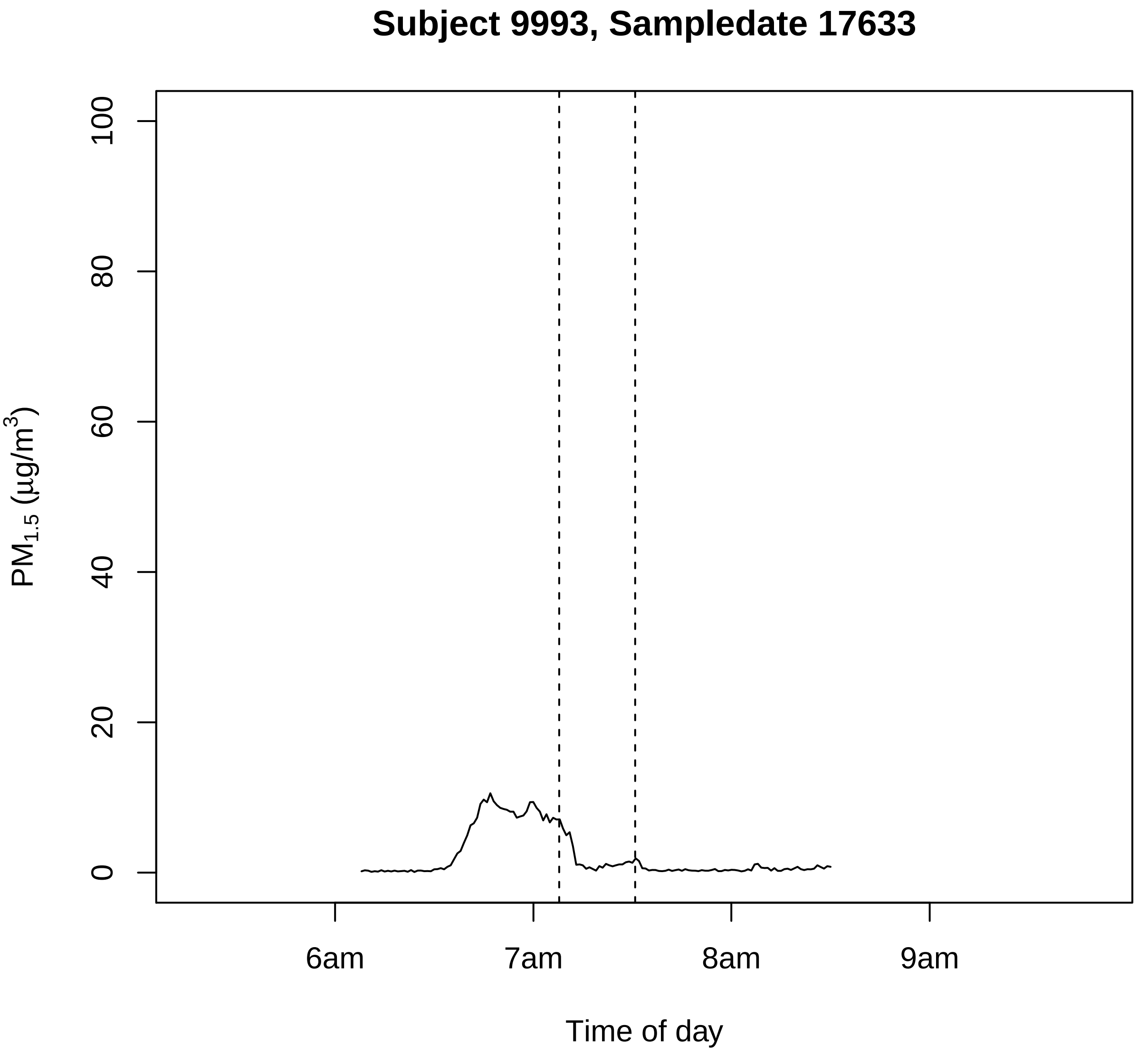
Subject 9993, Sampledate 17633
$PM_{1.5}$ (µg/m^3)
0
20
40
60
80
100
6am
7am
8am
9am
Time of day
